# New Synthetic Approach to C‑30 Ethers, Esters, and Amines of Betulin Using the Mitsunobu Reaction and Biological Evaluation of the Products

**DOI:** 10.1021/acsomega.6c00716

**Published:** 2026-04-13

**Authors:** Jan Bachořík, Ivo Frydrych, Soňa Gurská, Štěpán Dostál, Jan Pokorný, Petr Džubák, Marián Hajdúch, Milan Urban

**Affiliations:** a Department of Organic Chemistry, Faculty of Science, Palacký University Olomouc, 17. Listopadu 1192/12, 779 00 Olomouc, Czech Republic; b Laboratory of Experimental Medicine, Institute of Molecular and Translational Medicine, Faculty of Medicine and Dentistry, Palacký University and University Hospital Olomouc, Hněvotínská 1333/5, 779 00 Olomouc, Czech Republic; c Laboratory of Medicinal and Organic Chemistry, Institute of Molecular and Translational Medicine, Faculty of Medicine and Dentistry, Palacký University Olomouc, Hněvotínská 1333/5, 779 00 Olomouc, Czech Republic; d Department of Analytical Chemistry, Faculty of Science, Palacký University Olomouc, 17. Listopadu 1192/12, 779 00 Olomouc, Czech Republic

## Abstract

A novel three-step synthetic approach for modification of betulin at position C-30 was developed starting from commercially available betulin diacetate. The scope of this procedure exceeds significantly the previously used methods while providing higher yields. The final Mitsunobu reaction was the pivotal step of the synthesis, and after optimization, 39 new derivatives—ethers, esters, and amines—were synthesized in good to high yields. All the novel compounds were tested for *in vitro* cytotoxic activity against six cancer cell lines and two noncancer cell lines. Compounds **30** and **37** show potent cytotoxicity against CCRF-CEM leukemia cells (IC_50_ around 5 μM). In addition, **37** exhibits broad activity across multiple cancer cell lines, suggesting a promising multitargeted anticancer activity. Both compounds impair DNA and RNA synthesis, with **37** strongly inhibiting transcription at high doses. Analysis of apoptosis induction shows a divergent profile in both compounds; while **30** promotes robust apoptosis, derivative **37** appears to engage alternative cell death pathways. Biosynthetic disruption emerges as a promising anticancer strategy, with **37** as a top lead candidate.

## Graphical abstract

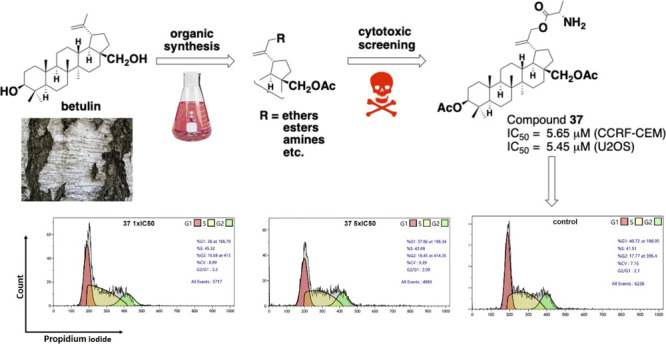

## Introduction

According to the data published in 2024, nature offers a non-negligible percentage of compounds that met the criteria of clinical trials and regulatory approval for medical usage.^1^ It also highlighted natural products and their derivatives as more viable options than synthetic compounds in medical research due to lower toxicity.^1^ These are strong and compelling findings for further development of natural products and their derivatives as potential new drugs.

Among many substances involved in contemporary research activities, pentacyclic triterpenoid betulin garnered significant attention of scientists including our research group. One of the primary reasons is its high availability in natural resources. It is commonly found and easily isolated from white parts of birch bark in large quantities, as reviewed and reported.^2–4^ Another key contribution is the wide range of biological activities of betulin derivatives including anticancer effects, which was thoroughly reviewed in 2015,^5^ as well as their antiviral^6^ and anti-inflammatory^7^ potential useful in the treatment of multiple sclerosis.^8^ Despite their abundance and numerous interesting biological effects, betulin analogues face significant challenges that need to be addressed before serious development, which include enhancement of biological effects and low solubility in water (0.08 μg/mL according to Jäger et al.).^9^

To improve these shortcomings, more modifications of the betulin chemical structure emerged, which became a powerful strategy to optimize the unfavorable properties of originally discovered lead structures. Betulin has several positions at its structures to be accessible to chemical modifications, and these were highly explored in the past, such as position C-3 and position C-28.^10–19^ Among the intriguing and less studied sites for chemical modification in the structure of betulin is the allylic position C-30. The majority of synthetic work at allylic position C-30 was achieved through the introduction of a bromine to this site via the NBS/CCl_4_ protocol^20–32^ that was subsequently substituted by a variety of nucleophiles such as carboxylates,^21,22^ amines,^27,28^ azides,^24,26,30,31^ pyridines and other nitrogen heterocycles,^25^ thioethers,^33,34^ silver nitrate,^32^ and phosphite.^35^ Consequent tests of biological activities revealed that some of these modifications at position C-30 showed a significant enhancement of anticancer activity, which motivated us to expand the numbers of such compounds and which became one of the goals of our current research interests. Specifically, a comprehensive study published by Chrobak et al. described the modification of betulin using ester functionalities.^36^ The results from the screening showed enhancement of the cytotoxic effects against cancer cells. The IC_50_ values ranged from 1.24 to 6.03 μM.

Inspired by previous work and recognizing a scarcity of progress in modification of betulin at its position C-30, we aimed to make an advancement in this area. In particular, we sought to establish a new and more robust procedure that would help to supersede the standard route dependent on the NBS/CCl_4_ protocol. Tetrachloromethane is being gradually phased out due to environmental and regulatory concerns,^37^ and the original procedure usually provided moderate to low yields. In this work, we describe optimized conditions for the introduction of the OH group at position C-30 in two steps followed by the Mitsunobu protocol for substitution with oxygen and nitrogen-based coupling partners. This reaction is versatile, high yielding, and proceeds under mild conditions, which is especially beneficial for natural products.^38^ A set of 39 new compounds was prepared and tested for cytotoxic activity. Out of these 39 new compounds, seven are esters analogous to the compounds from ref 36, differing only by a different substituent.

## Results and Discussion

### Chemistry

Our proposed three-step sequence starts from commercially available betulin diacetate. First, betulin diacetate was selectively oxidized at the allylic position (Scheme 1). Instead of the previously described conditions^39–41^ that required large quantities of selenium dioxide, we used a catalytic system (SeO_2_/TBHP). This not only improved our yields from the previously reported 56% (using the original methodology)^39^ to 84% using the new procedure but also prevented the extensive formation of colloidal elemental selenium that was always difficult to fully remove from the final product. In the second step, selective reduction of aldehyde (**1**) was performed under Luche conditions.

**Scheme 1:**
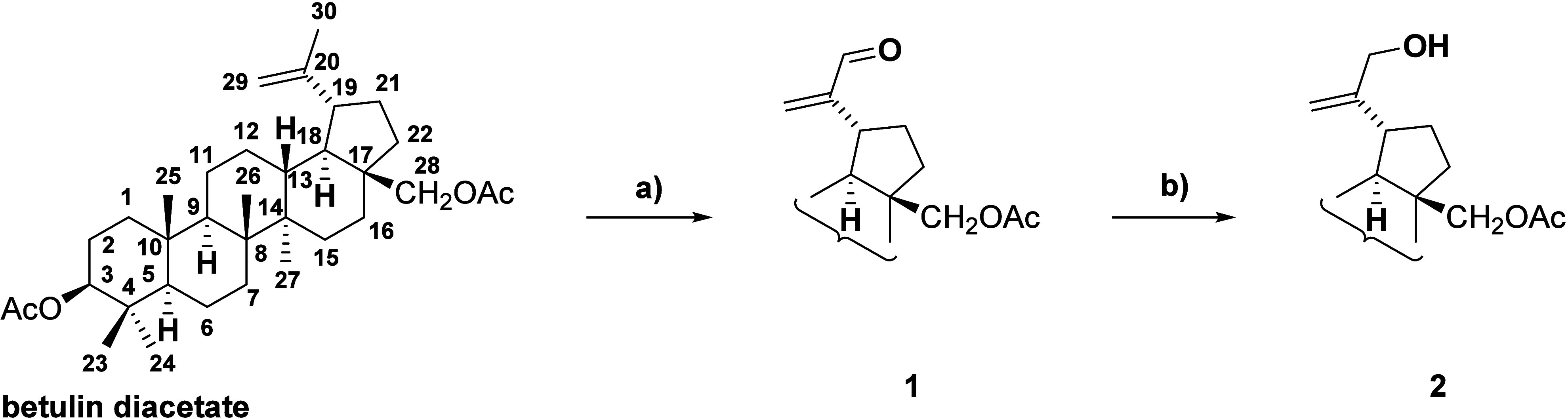
Modification of Betulin Diacetate at C-30 Using the Allylic Oxidation/Reduction Sequence^a^

a Reagents and conditions: (a) SeO_2_, TBHP, DCM, AcOH, rt, 48 h, 84%; (b) NaBH_4_, CeCl_3_·7H_2_O, THF, MeOH, 15 min, 78%.

As summarized in Table 1, our method offers a competitive alternative to previously reported protocols. Unlike the widely used NBS/CCl_4_ method, which involves toxic tetrachloromethane and delivers inconsistent yields, our two-step approach achieves comparable average efficiency and requires no toxic CCl_4_. Moreover, our protocol outperforms the existing two-step method in terms of overall yield.

**Table 1:** Comparison of Synthetic Strategies for Introduction of Leaving Group at Position C-30 (Bromine or Hydroxy Group) to the Betulin Diacetate

entry	synthetic strategy	number of steps	overall yield (%)
1	NBS/CCl_4_ ^20–32^	1	51–73 (66_avg_)
2	oxidation/hydrolysis sequence^36,42^	2	54
3	this work	2	66

The coupling reaction of derivative **2** and a suitable partner in the presence of ADDM/PBu_3_ was the last step of our sequence. This Mitsunobu reagent was chosen for its effectiveness, versatility, and convenient workup and purification procedures that were needed for universal use in triterpenoid chemistry.^43^ Concerning the coupling partners, we first focused on reactions with phenolic compounds that represent standard prenucleophiles for the Mitsunobu reaction.^38^ Our interest was to explore the effect of the presence of the alkoxy aryl ether moiety at position C-30 on the biological activity, which was motivated by their similarity to known active esters^36^ (both hydrogen bond acceptors^44^). Second, the ether derivatives proposed to be synthesized within this work have not been described yet to our knowledge in the literature. To evaluate the scope of the reaction and to expand our set of compounds for biological screening, 26 new ether derivatives (**3**–**28**) were prepared including those that contained various electron-donating and electron-withdrawing functional groups at the ortho, meta, and para positions (Scheme 2). The reaction time varied depending on the derivative prepared, being either 1 or 24 h, which is specified for each derivative in the experimental section. The reaction is highly tolerant to both electron-rich and electron-poor systems, although yields were generally higher in reactions with electron-neutral or slightly enriched rings. The scope of the reaction was also shown by the preparation of a protected amino acid **28**. All compounds (**3**–**28**) were prepared in high yields except for the pyridine derivative **27**. In addition, this method gave a complicated mixture of products with other pyridine isomers. This can be likely attributed to the slightly different acidity of pyridinols and their tautomerism to pyridone forms, which can provide alternative reaction sites within the molecular framework of prenucleophiles. On the other hand, compound **29** is a good example of a successful reaction with a BOC-protected amino acid, followed by selective deprotection that is not limited by the presence of acetyl functions.

**Scheme 2:**
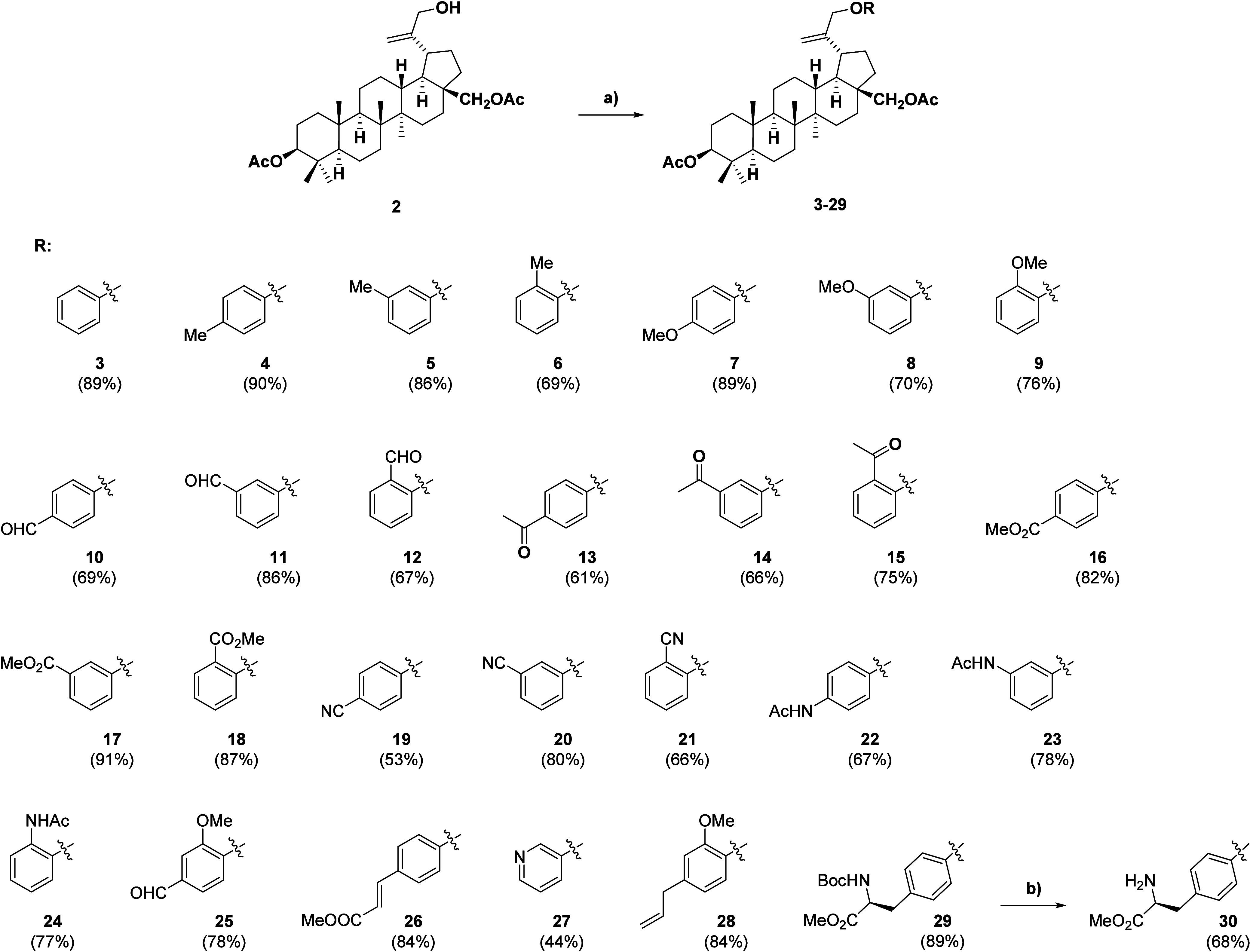
Triterpenoid C-30 Ethers Prepared by the Mitsunobu Protocol^a^

a Reagents and conditions: (a) R^1^OH, ADDM, PBu_3_, THF, rt, 1 to 24 h; (b) TFA, DCM, rt, 30 min, 68%.

Ester derivatives (**31**–**37**) were prepared (Scheme 3) in a similar manner as the ethers (**3**–**29**). The scope of the reaction was tested using a small number of selected aromatic, aliphatic, and heterocyclic carboxylic acids. The scope was intentionally limited for this specific class of compounds, as several alternative synthetic routes are well documented in the literature for the preparation of analogous compounds. These examples serve as a proof of concept to confirm that our protocol remains viable even for the type of derivatives typically prepared via the DCC protocol.^36^ Different substituents were used than in ref 36. The reaction time varied depending on the derivative prepared, being either 1 or 24 h, and is specified for each derivative in the Experimental Section. In all cases, the protocol provided derivatives in good to high yields except for compound **33** containing the electron-withdrawing nitrile functionality. Similarly to compound **30**, derivative **37** was prepared via the Mitsunobu reaction and subsequent BOC deprotection to demonstrate good compatibility with these types of modifications.

**Scheme 3:**
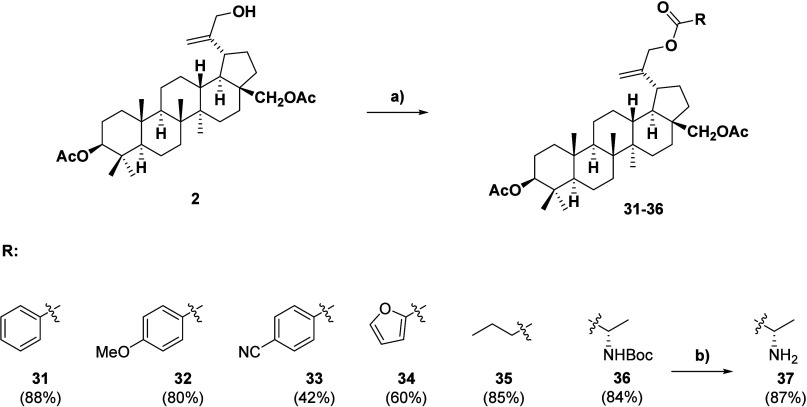
Triterpenoid C-30 Esters Prepared by the Mitsunobu Protocol^a^

a Reagents and conditions: (**a**) R_2_COOH, ADDM, PBu_3_, THF, rt, 1 to 24 h; (**b**) TFA, DCM, rt, 30 min, 87%.

To further demonstrate the synthetic versatility of the developed synthetic pathway, we tested its scope using structurally distinct nitrogen-based reagents such as phthalimide and sulfonamides (Scheme 4). The reaction time varied depending on the derivative prepared, being either 1 or 24 h, which is specified for each derivative in the Experimental Section. In every tested case, the reaction was high yielding and this clearly demonstrated the general applicability of the protocol. Also, denosylation of substance **40** was performed using standard deprotection conditions to demonstrate the usability of our synthetic pathway for the secondary amine synthesis of betulin.

**Scheme 4:**
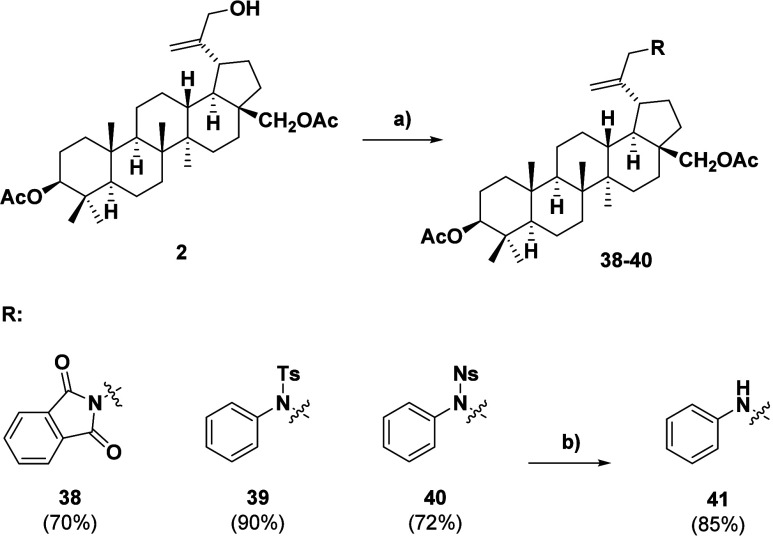
Triterpenoid C-30 Amides and Amines Prepared by the Mitsunobu Protocol^a^

a Reagents and conditions: (a) phthalimide or 4-methyl-*N*-phenylbenzenesulfonamide or 4-nitro-*N*-phenylbenzenesulfonamide, ADDM, PBu_3_, THF, rt, 1 to 24 h; (b) PhSH, DBU, MeCN, rt, 2 h, 85%.

## Biology

### Cytotoxic Activity on Cancer and Noncancer Cell Lines

The cytotoxic activity of the compounds included in this study was evaluated on a panel of human cancer cell lines (CCRF-CEM, HCT116, HCT116 p53^–/–^, K562, A549, and U2OS) and noncancerous cell lines (BJ and MRC-5) (Table 2). Compounds with IC_50_ values above 50 μM were considered inactive and are not included in Table 2. As evident from the data, the majority of the tested compounds exhibited activity in high micromolar ranges of concentrations across all cell lines. Despite this generally low activity, several compounds demonstrated moderate cytotoxicity in selected cancer cell lines. Compound **2** showed moderate cytotoxicity toward CCRF-CEM (IC_50_ = 16.68 μM) and less pronounced activity on HCT116 (34.96 μM), HCT116 p53^–/–^ (39.94 μM), and K562 (39.98 μM) cell lines. Additionally, compound **27** demonstrated selective cytotoxicity toward CCRF-CEM (IC_50_ = 16.90 μM), while being inactive in all other cell lines. Among the tested compounds, derivatives **30** and **37** stood out as the most active molecules. The best compound found in this study was **37** displaying remarkable cytotoxicity toward CCRF-CEM cells (IC_50_ = 5.65 μM) and moderate activity against HCT116 (12.30 μM), HCT116 p53^–/–^ (8.40 μM), K562 (6.65 μM), and U2OS (5.45 μM) cell lines while its activity in healthy cell lines was only moderate (BJ, IC_50_ = 26.42 μM and MRC-5, IC_50_ = 25.00 μM), showing decent selectivity index (SI) toward CCRF-CEM compared to BJ and MRC-5 cells. Consistent cytotoxicity of **37** across multiple cancer cell lines points to a potentially broad mechanism of action. Compound **30** demonstrated pronounced cytotoxicity in CCRF-CEM cells (IC_50_ = 9.61 μM) and moderate activity in HCT116 (31.02 μM), HCT116 p53^–/–^ (30.37 μM), and K562 (23.24 μM), while showing no significant activity toward noncancerous cell lines (IC_50_ > 50 μM). This suggests more favorable selectivity compared to **37**. In summary, despite the moderate to low activity of the majority of tested compounds, identification of derivatives **30** and **37** as highly active candidates, particularly toward CCRF-CEM, represents a valuable finding. In addition, moderate activity of compounds **2** and **27** suggests that even minor structural modifications may unlock selective cytotoxic potential. Further studies, including selectivity index evaluation and mechanism of action analyses, will be essential to fully characterizing these compounds and their therapeutic potential.

**Table 2:** Cytotoxicity (IC50, μmol/L) of Selected Compounds against Six Cancer Cell Lines (CCRF-CEM, HCT116, HCT116 p53^–/–^, K562, A549, and U2OS) and Two Noncancerous Cell Lines (BJ, MRC-5)

	IC_50_ (μmol/L)^a^								
comp.	CCRF-CEM	HCT116	HCT116 p53^–/–^	K562	A549	U2OS	BJ	MRC-5	SI^b^
2	16.68	34.96	39.94	39.98	38.69	26.27	>50	>50	>3.00
27	16.90	>50	>50	>50	>50	>50	>50	>50	>2.96
30	9.61	31.02	30.37	23.24	>50	28.28	40.01	>50	>4.68
37	5.65	12.30	8.40	6.65	25.91	5.45	26.42	25.00	4.55

a The IC_50_ represents the concentration of the drug required to inhibit cell growth by 50%. The standard deviation in cytotoxicity assays typically reaches up to 15% of the mean value.

b The selectivity index is calculated based on the IC_50_ for the CCRF-CEM line versus the average IC_50_ for both fibroblast lines. For IC_50_ values reported as >50 μM, SI values are expressed as lower bounds.

To gain preliminary insights into the pharmacokinetic behavior of the most potent derivatives, an in silico ADME profiling was conducted using the SwissADME web server;^45^ the results are shown in Table S2. The calculations indicate that the most promising compounds **2**, **27**, **30**, and **37** show poor solubility and deviate from the classical Lipinski’s Rule of Five, primarily due to the high molecular weight and significant lipophilicity. Despite that, if these molecules are selected for further development, there are many options to reach sufficient plasma of terpenoid compounds using prodrugs and additives within administrations.^46–48^ Toxicity profiling was also conducted using the ProTox 3.0 Web server.^49^ The results shown in the Supporting Information file as Table S3 indicate that compound **2** has the highest predicted value of LD50 (5000 mg/kg) and compound **37** the second highest (LD50 = 5000 mg/kg). The possible toxicity targets for both of these compounds are amine oxidase A (AOFA) and prostaglandin G/H synthase 1 (PGH1).

### Investigation of Cell Cycle Alterations, DNA/RNA Synthesis, and Apoptosis after Treatment with **30** and **37**

To ensure transparent interpretation of all measured cellular end points, the flow cytometry results in this study are presented in the form of representative histograms and dot plots. These graphical outputs represent the primary readouts of multiparametric flow cytometry and directly illustrate treatment-induced changes in DNA content, mitotic activity, DNA and RNA syntheses, and apoptosis without requiring additional bar graph summarization. All relevant gates, subpopulations, and positivity thresholds are clearly annotated in the displayed plots to facilitate straightforward interpretation. Using this approach, we next assessed how compounds **30** and **37** influence cell cycle progression and biosynthetic processes in CCRF-CEM leukemia cells. To gain insight into the mechanisms underlying their cytotoxicity, we analyzed cell cycle distribution, mitotic index (pH3Ser10), DNA synthesis (BrdU incorporation), RNA synthesis (BrU incorporation), and apoptosis (sub-G1) after 24 h exposure at 1× and 5× IC_5_0 concentrations (Figure 1).

**Figure 1:**
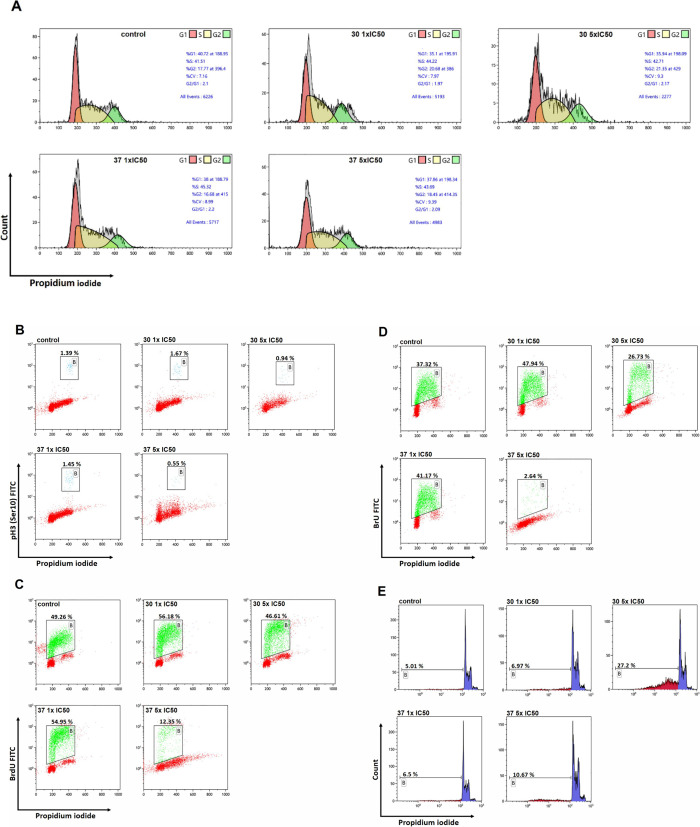
Multiparametric flow cytometry analysis of the cellular response to compounds **30** and **37** in CCRF-CEM cells. (A) Cell cycle distribution. Representative PI histograms showing G1, S, and G2/M phase profiles after 24 h treatment with compounds **30** and **37** at 1 × IC_50_ and 5 × IC_50_. PI fluorescence (*x*-axis) was used to quantify total DNA content; only live, single-cell events were included. Untreated cells served as control. (B) Mitotic index (pH3Ser10). Flow cytometric detection of phospho-histone H-3 (Ser10)–positive cells as a measure of mitotic activity. Cells were stained with the anti-pH3Ser10 antibody following identical treatment conditions. Percentages represent the proportion of mitotic cells within the PI-gated population (region B). (C) DNA synthesis activity (BrdU incorporation). Cells were pulse-labeled with BrdU prior to harvesting. BrdU-positive cells (region B) reflect actively replicating populations. Dot plots show the relative BrdU incorporation under each treatment condition. (D) RNA synthesis activity (BrU incorporation). BrU-labeled nascent RNA was detected by flow cytometry using an anti-BrdU antibody cross-reactive with BrU. Region B denotes BrU-positive cells. Data illustrates treatment-dependent modulation of transcriptional activity. (E) Apoptotic cell death (sub-G1). Representative PI histograms showing the proportion of cells with sub-G1 DNA content following 24 h exposure to the compounds. The sub-G1 fraction quantifies apoptotic DNA fragmentation. All experiments were performed in biological triplicates with similar results. Flow cytometry data were processed and quantitatively evaluated using Kaluza software (Beckman Coulter). Collectively, these multiparametric analyses provide an integrated overview of how compounds **30** and **37** affect cell cycle dynamics, biosynthetic capacity, and cell death pathways in leukemia cells.

Collectively, these assays provide a multifaceted view of how these compounds affect critical cellular processes linked to proliferation and survival. Interestingly, neither compound induced substantial alterations in cell cycle distribution compared to untreated controls (Figure 1a). For both **30** and **37**, the proportions of cells in the G0/G1, S, and G2/M phases remained remarkably stable across tested concentrations. This observation suggests that cell cycle arrest is not a dominant mechanism of action for these compounds. Instead, their cytotoxicity appears to arise from more subtle or indirect perturbations of key biosynthetic processes. This notion is supported by analysis of the mitotic index, measured via pH3Ser10 expression (Figure 1b). While minor fluctuations were observed (slightly increased at 1 × IC_50_ and decreased at 5 × IC_50_), neither compound consistently elevated the mitotic population, further indicating that direct blockade of mitotic progression is unlikely to account for the cytotoxic effects (Figure 1b). A more revealing pattern emerged from the assessment of DNA synthesis (Figure 1c). At lower concentrations (1 × IC_50_), both **30** and **37** stimulated DNA synthesis or BrDU incorporation compared to control cells (56.18 and 54.95 vs 49.26%, respectively). This transient increase could reflect a compensatory hyper-replication/replication stress or DNA repair response triggered by initial cellular damage. However, at higher concentrations (5 × IC_50_), a divergent profile emerged: **30** displayed only a modest reduction in DNA synthesis (46.61%), whereas **37** profoundly suppressed DNA synthesis (12.35%). Such concentration-dependent inhibition is consistent with induction of replication stress, which is a well-documented trigger of DNA damage signaling, cell cycle checkpoints, and ultimately apoptosis. An analogous pattern was observed in the RNA synthesis assays (Figure 1d). At 1 × IC_50_, both compounds stimulated RNA synthesis (47.94 and 41.17 vs 37.32%, respectively), perhaps as part of an early adaptive response to stress. However, at higher concentrations (5 × IC_50_), both compounds, particularly **37**, induced a marked suppression of RNA synthesis (**30**: 26.73%; **37**: 2.64%). The profound inhibition of RNA synthesis by **37** at 5 × IC_50_ is especially striking and suggests disruption of transcriptional machinery, which can rapidly compromise cell viability and trigger programmed cell death pathways. Taken together, these findings point toward a multifaceted mechanism of cytotoxicity in which **30** and **37**, rather than imposing overt cell cycle arrest, likely interfere with DNA replication and transcriptional processes, especially at higher concentrations. The initial mild stimulation of DNA/RNA synthesis at lower doses might reflect compensatory S-phase entry due to unscheduled replication in response to DNA damage rather than regular proliferation. However, at higher doses, especially for **37**, this dynamic shifts dramatically toward robust inhibition of both DNA and RNA synthesis, consistent with a collapse of essential biosynthetic pathways and activation of cell death. Mechanistically, such inhibition could arise from direct or indirect interference with DNA or transcriptional machinery. Notably, these results align with the concept that targeting fundamental biosynthetic processes can be an effective anticancer strategy, particularly in rapidly proliferating leukemia cells such as CCRF-CEM. Overall, the present data highlight **37**, in particular, as a potent modulator of essential cellular pathways, supporting its prioritization for further preclinical evaluation as a potential anticancer agent. Given the observed impact of these compounds on biosynthetic processes, we next explored whether these perturbations translate into the activation of programmed cell death, assessed as the sub-G1 apoptotic population. Apoptosis induction was evaluated as the sub-G1 population using flow cytometry, providing key insights into the cell death mechanisms triggered by **30** and **37** (Figure 1e). In untreated CCRF-CEM cells, the sub-G1 fraction accounted for 5.01% of the population, reflecting baseline levels of apoptosis. Treatment with **30** at a concentration equivalent to its IC_50_ resulted in a modest increase in the sub-G1 fraction (6.97%), indicating a limited pro-apoptotic response at lower doses. However, at 5 × IC_50_, a dramatic increase in the sub-G1 population was observed (27.20%), pointing to a concentration-dependent induction of apoptosis. This finding aligns seamlessly with the observed modest inhibition of DNA and RNA synthesis at higher concentrations of **30**, suggesting that while the compound does not induce strong cell cycle arrest or mitotic blockade, its interference with biosynthetic processes eventually triggers apoptotic cell death. In contrast, **37** demonstrated a different apoptotic profile. At 1 × IC_50_, it produced a modest increase in the sub-G1 fraction (6.5%), comparable to **30**, indicating early signs of apoptosis induction. However, even at 5 × IC_50_, the sub-G1 fraction reached only 10.67%, substantially lower than the apoptotic response observed for 30 at the same concentration. This observation is particularly intriguing given that **37** exhibited a more pronounced suppression of DNA and RNA synthesis at higher concentrations than **30**. The dissociation between the strong suppression of biosynthetic processes and the relatively modest induction of apoptosis suggests that **37** may engage additional, nonapoptotic mechanisms of cytotoxicity, such as necrosis, autophagy, or irreversible replication stress culminating in mitotic catastrophe. Alternatively, the timing of the measurement may have captured an early phase of cell death before full apoptotic commitment occurs. Taken together, these data highlight the complex interplay between biosynthetic disruption and apoptosis induction. While both compounds interfere with DNA and RNA syntheses, especially at higher concentrations, only **30** induces robust apoptosis as reflected by the sub-G1 accumulation, whereas **37** triggers a less pronounced apoptotic response despite its potent inhibition of macromolecular synthesis. This polarity underscores the importance of integrating multiple analytical end points to fully define the cytotoxic mechanisms of novel anticancer agents.

## Conclusions

The main goal of this research was to find an optimized synthetic sequence for derivatization of betulin at its position C-30 and expand the library of known semisynthetic derivatives. Our new synthetic pathway led to the synthesis of various triterpenoid ethers, esters, phthalimides, and sulfonamides. The synthesis was done through a three-step process: first, optimized allylic oxidation, followed by a selective reduction of carbonyl group, ending with Mitsunobu substitution. This synthetic strategy generated a broad range of novel compounds in good to high yields and opens many options for future syntheses of multiple-compound libraries.

Basic screening of the cytotoxic activity in a set of cancerous and healthy cells showed that compound **37** was the most active (IC_50_ values 5.65 in CCRF-CEM and 5.45 μM in U2OS). This study reveals that within this structurally diverse compound library, only a small subset exhibited meaningful cytotoxicity, highlighting the importance of structural fine tuning to achieve potent anticancer activity. Notably, **30** and **37** emerged as the most promising candidates, displaying pronounced activity against CCRF-CEM leukemia cells, with **37** demonstrating a broader cytotoxic profile across additional cancer cell lines. Although compounds **30** and **37** exhibit poor predicted biopharmaceutical properties, they serve as lead scaffolds. Future structure optimization will focus on reducing lipophilicity and exploring other strategies (e.g., lipid-based carriers) to enhance the solubility and absorption of these hydrophobic candidates. Mechanistic investigations revealed that the cytotoxicity of these compounds is not primarily mediated by classical cell cycle arrest or mitotic blockade, but rather by profound perturbations of essential biosynthetic processes, particularly DNA and RNA syntheses. The pronounced concentration-dependent inhibition of DNA and RNA synthesis observed for **37**, coupled with its modest apoptotic induction, suggests a complex mechanism involving replication stress and potential engagement of alternative cell death pathways. Conversely, **30** demonstrated robust apoptosis at higher concentrations, indicating a more direct link between biosynthetic inhibition and programmed cell death. These findings underscore the therapeutic potential of targeting biosynthetic machinery as an anticancer strategy and position **37**, in particular, as a compelling lead candidate for further preclinical development. The study further highlights the importance of integrated cytotoxicity, cell cycle, and apoptosis analyses in unravelling the multifaceted mechanisms of novel anticancer compounds, providing a strong rationale for future mechanistic and *in vivo* studies.

## Experimental Section

### Chemistry

#### General Information

All reagents were of reagent grade and were used without further purification. Starting betulin diacetate in purity 98% was purchased from the company Betulinines (www.betulinines.com). All other chemicals and solvents including dry ones were purchased from Merck (Germany). The course of the reactions was monitored by TLC on Kieselgel 60 F_254_ plates (Merck, Germany) and detected by UV light (254 nm) followed by visualization using 10% aqueous H_2_SO_4_ and heating process to 150–200 °C. Purification was performed using column chromatography on Silica gel 60 Merck 7734 (Merck, Germany). All ^1^H and ^13^C NMR experiments were recorded at 500 MHz (JEOL JNM-ECX-500) or 400 MHz (JEOL JNM-ECA400II) for ^1^H NMR, and 126 or 100 MHz for ^13^C NMR, respectively, at 25 °C in CDCl_3_. Chemical shifts δ are reported relative to the residual solvent peak (for CDCl_3_δH = 7.26 ppm, δC = 77.16 ppm). Chemical shifts δ are reported in parts per million (ppm), and coupling constants *J* are reported in Hertz (Hz). HRMS analyses were performed on a SELECT SERIES Cyclic IMS QTOF (Waters Corp., Wilmslow, U.K.) equipped with an Atmospheric Solid Analysis Probe (ASAP) source operated in positive mode. Sodium iodide 2 μg/μL in propan-2-ol/water (1:1, v/v) was used for the mass calibration (*m*/*z* 50–2000). Instrumental parameters were set as follows: corona current 2 μA and desolvation temperature of 400 °C under standard conditions and 500 °C for less efficiently ionizing analytes, respectively. Data were acquired with a lockmass correction using leucine enkephalin at a concentration of 50 pg/μL in water/acetonitrile (1:1, v/v) containing 0.1% formic acid, monitoring the [M + H]^+^ ion at *m*/*z* 556.2771. Samples were dissolved in acetone and analyzed at a concentration of 10 μg/mL, with an increased concentration of 50 μg/mL applied for compounds exhibiting poorer ionization efficiency. For ASAP introduction, a quartz capillary was immersed directly into the sample solution and subsequently positioned in the ASAP source for direct analysis. IR samples were analyzed by Fourier transform infrared spectroscopy in attenuated total reflectance (ATR) mode using a Nicolet iS50 FTIR spectrometer (Thermo Fisher Scientific).

##### Synthesis of 3β,28-Diacetoxy-30-oxolup-20(29)-ene (**1**)

Betulin diacetate (98%, 10.2 g, 0.019 mol) was dissolved in a mixture of DCM (132 mL) and AcOH (78 mL). Both 70% aqueous solution of *tert*-butyl hydroperoxide (7.8 mL, 0.057 mol) and selenium dioxide (632 mg, 0.0057 mol) were then added into the reaction mixture. The reaction mixture was stirred at room temperature for 48 h. The reaction was determined to be complete by TLC analysis using mobile phase hexane/EtOAc (4:1). The reaction mixture was diluted with DCM (200 mL). The organic phase was washed three times with 1 M solution of FeSO_4_·7H_2_O (250 mL), two times with saturated solution of NaHCO_3_ (250 mL), and once with brine (200 mL). The organic phase was dried over anhydrous MgSO_4_, and the organic solvent was subsequently removed under reduced pressure using a rotary evaporator, followed by column chromatography purification on SiO_2_ eluting with a gradient mobile phase of hexane/EtOAc (7:1 to 4:1). After the purification process, aldehyde **1** was obtained in 84% yield as a white solid. ^1^H NMR (500 MHz, CDCl_3_): δ 9.50 (s, 1H, CHO), 6.27 (s, 1H, H-29), 5.92 (s, 1H, H-29), 4.47 – 4.42 (m, 1H, H-3), 4.27 (d, *J* = 11.2 Hz, 1H, H-28), 3.86 (d, *J* = 11.1 Hz, 1H, H-28), 2.89 – 2.69 (m, 1H, H-19), 2.06 (s, 3H, CH_3_C=O), 2.03 (s, 3H, CH_3_C=O), 1.01 (s, 3H, CH_3_), 0.93 (s, 3H, CH_3_), 0.84 – 0.81 (m, 9H, 3 × CH_3_). ^13^C NMR (126 MHz, CDCl_3_): δ 194.83, 171.59, 171.14, 156.70, 133.37, 81.02, 62.56, 55.50, 50.22, 46.72, 42.71, 40.97, 38.51, 37.92, 37.37, 37.29, 37.17, 34.62, 34.25, 32.16, 29.87, 28.07, 27.59, 27.11, 23.80, 21.44, 21.16, 20.93, 18.28, 16.62, 16.24, 16.13, 14.73, 14.34. IR (DRIFT): 2938.56, 2868.40, 1736.71, 1722.09, 1687.35, 1460.32, 1389.86, 1365.05, 1235.03, 1028.61, 1014.09. HRMS (ASAP, TOF ES^+^): calcd. for C_34_H_53_O_5_^+^ [M + H]^+^ 541.3888; found 541.3896.

##### Synthesis of 3β,28-Diacetoxy-30-hydroxylup-20(29)-ene (**2**)

Aldehyde **1** (6.44 g, 0.012 mol) and CeCl_3_·7H_2_O (8 g, 0.0216 mol) were dissolved in a mixture of THF (120 mL) and MeOH (40 mL). Sodium borohydride (817 mg, 0.0216 mol) was then added into the reaction mixture in small doses. The reaction mixture was stirred at room temperature for 15 min, after the complete addition of the sodium borohydride. The reaction was determined to be complete by TLC analysis using mobile phase hexane/EtOAc (3:1). The reaction mixture of diluted EtOAc (180 mL) and 1 M solution of HCl (90 mL) was added. After phase separation, the aqueous layer was re-extracted with an additional portion of EtOAc (60 mL). The combined organic extracts were then washed once with H_2_O (90 mL) and after that once with brine (90 mL). The organic phase was dried over anhydrous MgSO_4_, and the organic solvent was subsequently removed under reduced pressure using a rotary evaporator, followed by column chromatography purification on SiO_2_ eluting with a gradient mobile phase of hexane/EtOAc (4:1 to 2:1). After the purification process, hydroxy derivative **2** was obtained in 78% yield as a white solid. ^1^H NMR (500 MHz, CDCl_3_): δ 4.96 (d, *J* = 1.1 Hz, 1H, H-29), 4.90 (s, 1H, H-29), 4.48 – 4.44 (m, 1H, H-3), 4.23 (dd, *J* = 11.2, 1.4 Hz, 1H, H-28), 4.15 – 4.07 (m, 2H, H-30), 3.84 (d, *J* = 11.0 Hz, 1H, H-28), 2.34 (td, *J* = 11.0, 5.5 Hz, 1H, H-19), 2.06 (s, 3H, CH_3_C=O), 2.03 (s, 3H, CH_3_C=O), 1.03 (s, 3H, CH_3_), 0.97 (s, 3H, CH_3_), 0.85 – 0.83 (m, 6H, 2 × CH_3_), 0.83 (s, 3H). ^13^C NMR (126 MHz, CDCl_3_): δ 171.71, 171.13, 154.36, 107.43, 81.04, 65.13, 62.62, 55.52, 50.40, 49.61, 46.48, 43.59, 42.81, 41.07, 38.53, 37.95, 37.65, 37.21, 34.53, 34.30, 31.70, 29.90, 28.09, 27.19, 26.84, 23.83, 21.45, 21.16, 21.05, 18.31, 16.63, 16.30, 16.19, 14.87. IR (DRIFT): 3552.05, 2939.03, 2870.45, 1731.97, 1712.75, 1456.62, 1363.27, 1242.00, 1030.27, 1013.20. HRMS (ASAP, TOF ES^+^): calcd. for C_34_H_55_O_5_^+^ [M + H]^+^ 543.4044; found 543.4021.

#### General Procedure for the Mitsunobu Reaction

The Mitsunobu reaction was performed under inert conditions. Alcohol **2** (326 mg, 0.6 mmol), tributyl phosphine (600 μL, 2.4 mmol), and prenucleophile (2.4 mmol) were dissolved in anhydrous THF (12 mL). Azodicarboxylic bismorpholide (ADDM, 615 mg, 2.4 mmol) was then added into the reaction mixture. The reaction mixture was stirred at room temperature for 1 or 24 h (depended on prenucleophile). The reaction was determined to be complete by TLC analysis using mobile phase hexane/EtOAc (3:1). The reaction mixture was diluted with diethyl ether (50 mL). The organic phase was washed once with H_2_O (20 mL), three times with 1 M solution of NaOH (20 mL), and once with brine (20 mL). The organic phase was dried over anhydrous MgSO_4_, and the organic solvent was subsequently removed under reduced pressure using a rotary evaporator, followed by column chromatography purification on SiO_2_ eluting with organic solvents (details will be specified for each derivative).

#### Synthesis of Ether Compounds **3**–**29**

##### 3β,28-Diacetoxy-30-phenoxylup-20­(29)-ene (**3**)

Compound **3** was prepared according to the general procedure for the Mitsunobu reaction. The reaction time was 1 h. After the column chromatography purification using hexane/EtOAc (8:1), derivative **3** was obtained in 70% yield as a white solid. ^1^H NMR (500 MHz, CDCl_3_): δ 7.31 – 7.27 (m, 2H, aryl), 6.97 – 6.91 (m, 3H, aryl), 5.09 (d, *J* = 1.1 Hz, 1H, H-29), 5.02 (s, 1H, H-29), 4.49 – 4.45 (m, 3H, H-3, H-30), 4.26 (dd, *J* = 11.2, 1.1 Hz, 1H, H-28), 3.85 (d, *J* = 11.0 Hz, 1H, H-28), 2.44 (td, *J* = 11.3, 5.5 Hz, 1H, H-19), 2.07 (s, 3H, CH_3_C=O), 2.04 (s, 3H, CH_3_C=O), 1.04 (s, 3H, CH_3_), 0.97 (s, 3H, CH_3_), 0.85 (s, 3H, CH_3_), 0.85 (s, 3H, CH_3_), 0.84 (s, 3H, CH_3_). ^13^C NMR (126 MHz, CDCl_3_): δ 171.72, 171.14, 158.98, 149.87, 129.57, 120.95, 114.84, 110.68, 81.06, 70.36, 62.68, 55.54, 50.43, 49.65, 46.50, 43.68, 42.85, 41.10, 38.55, 37.96, 37.70, 37.23, 34.53, 34.33, 31.55, 29.94, 29.85, 28.10, 27.21, 26.91, 23.85, 21.46, 21.18, 21.09, 18.32, 16.64, 16.32, 16.22, 14.93. IR (DRIFT): 2936.17, 2866.35, 1731.66, 1598.77, 1241.33, 1029.74. HRMS (ASAP, TOF ES^+^): calcd. for C_40_H_59_O_5_^+^ [M + H]^+^ 619.4357; found 619.4318.

##### 3β,28-Diacetoxy-30-(4-methylphenoxy)­lup-20­(29)-ene (**4**)

Compound **4** was prepared according to the general procedure for the Mitsunobu reaction. The reaction time was 1 h. After the column chromatography purification using hexane/EtOAc (6:1), derivative **4** was obtained in 90% yield as a white solid. ^1^H NMR (500 MHz, CDCl_3_): δ 7.09 – 7.06 (m, 2H, aryl), 6.83 – 6.80 (m, 2H, aryl), 5.07 (d, *J* = 1.1 Hz, 1H, H-29), 5.00 (s, 1H, H-29), 4.49 – 4.44 (m, 3H, H-3, H-30), 4.26 (dd, *J* = 11.1, 1.0 Hz, 1H, H-28), 3.85 (d, *J* = 11.0 Hz, 1H, H-28), 2.43 (td, *J* = 11.2, 5.3 Hz, 1H, H-19), 2.29 (s, 3H, benzylic), 2.07 (s, 3H, CH_3_C=O), 2.04 (s, 3H, CH_3_C=O), 1.04 (s, 3H, CH_3_), 0.97 (s, 3H, CH_3_), 0.85 (s, 3H, CH_3_), 0.85 (s, 3H, CH_3_), 0.84 (s, 3H, CH_3_). ^13^C NMR (126 MHz, CDCl_3_) δ 171.71, 171.12, 156.89, 150.00, 130.14, 129.99, 114.69, 110.54, 81.05, 70.49, 62.68, 55.53, 50.42, 49.61, 46.49, 43.70, 42.84, 41.09, 38.54, 37.95, 37.69, 37.22, 34.52, 34.32, 31.52, 29.93, 28.10, 27.21, 26.86, 23.84, 21.45, 21.17, 21.08, 20.62, 18.32, 16.64, 16.31, 16.21, 14.92. IR (DRIFT): 2942.84, 2870.45, 1732.19, 1509.45, 1236.67, 1028.72. HRMS (ASAP, TOF ES^+^): calcd. for C_41_H_61_O_5_^+^ [M + H]^+^ 633.4514; found 633.4470.

##### 3β,28-Diacetoxy-30-(3-methylphenoxy)­lup-20­(29)-ene (**5**)

Compound **5** was prepared according to the general procedure for the Mitsunobu reaction. The reaction time was 1 h. After the column chromatography purification using hexane/EtOAc (6:1), derivative **5** was obtained in 86% yield as a white solid. ^1^H NMR (400 MHz, CDCl_3_): δ 7.16 (t, *J* = 7.8 Hz, 1H, aryl), 6.78 – 6.71 (m, 3H, aryl), 5.08 (d, *J* = 1.2 Hz, 1H, H-29), 5.00 (s, 1H, H-29), 4.50 – 4.45 (m, 3H, H-3, H-30), 4.26 (dd, *J* = 11.2, 1.0 Hz, 1H, H-28), 3.85 (d, *J* = 11.1 Hz, 1H, H-28), 2.43 (td, *J* = 11.2, 5.4 Hz, 1H), 2.33 (s, 3H, benzylic), 2.07 (s, 3H, CH_3_C=O), 2.04 (s, 3H, CH_3_C=O), 1.04 (s, 3H, CH_3_), 0.98 (s, 3H, CH_3_), 0.85 (s, 3H, CH_3_), 0.85 (s, 3H, CH_3_), 0.84 (s, 3H, CH_3_).^13^C NMR (101 MHz, CDCl_3_): δ 171.72, 171.13, 159.00, 149.94, 139.56, 129.28, 121.77, 115.67, 111.71, 110.47, 81.04, 70.31, 62.67, 55.52, 50.41, 49.62, 46.48, 43.63, 42.83, 41.08, 38.52, 37.95, 37.68, 37.21, 34.51, 34.31, 31.54, 29.92, 28.09, 27.20, 26.91, 23.84, 21.69, 21.45, 21.17, 21.07, 18.31, 16.63, 16.31, 16.21, 14.91. IR (DRIFT): 2941.34, 2868.40, 1731.61, 1585.16, 1238.76, 1028.45. HRMS (ASAP, TOF ES^+^): calcd. for C_41_H_61_O_5_^+^ [M + H]^+^ 633.4514; found 633.4485.

##### 3β,28-Diacetoxy-30-(2-methylphenoxy)­lup-20­(29)-ene (**6**)

Compound **6** was prepared according to the general procedure for the Mitsunobu reaction. The reaction time was 1 h. After the column chromatography purification using hexane/EtOAc (6:1), derivative **6** was obtained in 85% yield as a white solid. ^1^H NMR (400 MHz, CDCl_3_): δ 7.17 – 7.12 (m, 2H, aryl), 6.88 – 6.81 (m, 2H, aryl), 5.13 (d, *J* = 1.2 Hz, 1H, H-29), 5.02 (s, 1H, H-29), 4.49 – 4.45 (m, 3H, H-3, H-30), 4.27 (dd, *J* = 11.1, 1.2 Hz, 1H, H-28), 3.85 (d, *J* = 11.1 Hz, 1H, H-28), 2.44 (td, *J* = 11.1, 5.4 Hz, 1H, H-19), 2.26 (s, 3H, benzylic), 2.07 (s, 3H, CH_3_C=O), 2.04 (s, 3H, CH_3_C=O), 1.04 (s, 3H, CH_3_), 0.98 (s, 3H, CH_3_), 0.85 (s, 3H, CH_3_), 0.85 (s, 3H, CH_3_), 0.84 (s, 3H, CH_3_). ^13^C NMR (101 MHz, CDCl_3_): δ 171.69, 171.12, 157.10, 150.22, 130.84, 126.97, 126.85, 120.54, 111.04, 109.99, 81.03, 70.53, 62.64, 55.52, 50.41, 49.79, 46.48, 43.29, 42.82, 41.08, 38.53, 37.95, 37.69, 37.21, 34.52, 34.31, 31.82, 29.93, 28.09, 27.21, 27.00, 23.83, 21.45, 21.17, 21.07, 18.31, 16.64, 16.57, 16.30, 16.20, 14.92. IR (DRIFT): 2942.82, 2866.35, 1732.07, 1493.89, 1239.28, 1028.41. HRMS (ASAP, TOF ES^+^): calcd. for C_41_H_61_O_5_^+^ [M + H]^+^ 633.4514; found 633.4477.

##### 3β,28-Diacetoxy-30-(4-methoxyphenoxy)­lup-20­(29)-ene (**7**)

Compound **7** was prepared according to the general procedure for the Mitsunobu reaction. The reaction time was 1 h. After the column chromatography purification using hexan/EtOAc (6:1), derivative **7** was obtained in 89% yield as a white solid. ^1^H NMR (500 MHz, CDCl_3_): δ 6.87 – 6.85 (m, 2H, aryl), 6.84 – 6.82 (m, 2H, aryl), 5.08 (d, *J* = 1.4 Hz, 1H, H-29), 5.00 (s, 1H, H-29), 4.49 – 4.45 (m, 1H, H-3), 4.42 (s, 2H, H-30), 4.26 (dd, *J* = 11.1, 1.2 Hz, 1H, H-28), 3.85 (d, *J* = 11.0 Hz, 1H, H-28), 3.77 (s, 3H, CH_3_O), 2.43 (td, *J* = 11.2, 5.4 Hz, 1H, H-19), 2.07 (s, 3H, CH_3_C=O), 2.04 (s, 3H, CH_3_C=O), 1.04 (s, 3H, CH_3_), 0.97 (s, 3H, CH_3_), 0.85 (s, 3H, CH_3_), 0.84 (s, 3H, CH_3_), 0.84 (s, 3H, CH_3_). ^13^C NMR (126 MHz, CDCl_3_) δ 171.73, 171.15, 154.03, 153.20, 150.13, 115.75, 114.77, 110.53, 81.06, 71.11, 62.69, 55.89, 55.54, 50.44, 49.63, 46.49, 43.66, 42.85, 41.10, 38.55, 37.96, 37.70, 37.23, 34.53, 34.33, 31.54, 29.94, 28.10, 27.21, 26.88, 23.85, 21.46, 21.18, 21.09, 18.32, 16.64, 16.32, 16.22, 14.93. IR (DRIFT): 2939.65, 2868.40, 1731.40, 1506.88, 1228.97, 1029.62. HRMS (ASAP, TOF ES^+^): calcd. for C_41_H_61_O_6_^+^ [M + H]^+^ 649.4463; found 649.4421.

##### 3β,28-Diacetoxy-30-(3-methoxyphenoxy)­lup-20­(29)-ene (**8**)

Compound **8** was prepared according to the general procedure for the Mitsunobu reaction. The reaction time was 1 h. After the column chromatography purification using hexane/EtOAc (6:1), derivative **8** was obtained in 70% yield as a white solid. ^1^H NMR (500 MHz, CDCl_3_): δ 7.18 (t, *J* = 8.2 Hz, 1H, aryl), 6.54 – 6.48 (m, 3H, aryl), 5.08 (d, *J* = 0.9 Hz, 1H, H-29), 5.01 (s, 1H, H-29), 4.49 – 4.43 (m, 3H, H-3, H-30), 4.26 (d, *J* = 11.1 Hz, 1H, H-28), 3.85 (d, *J* = 11.1 Hz, 1H, H-28), 3.79 (s, 3H, CH_3_O), 2.43 (td, *J* = 11.2, 5.4 Hz, 1H, H-19), 2.07 (s, 3H, CH_3_C=O), 2.04 (s, 3H, CH_3_C=O), 1.04 (s, 3H, CH_3_), 0.97 (s, 3H, CH_3_), 0.85 (s, 3H, CH_3_), 0.85 (s, 3H, CH_3_), 0.84 (s, 3H, CH_3_). ^13^C NMR (126 MHz, CDCl_3_): δ 171.72, 171.14, 160.97, 160.25, 149.78, 129.98, 110.66, 107.14, 106.51, 101.35, 81.05, 70.43, 62.67, 55.53, 55.43, 50.42, 49.65, 46.49, 43.68, 42.85, 41.10, 38.54, 37.96, 37.69, 37.23, 34.53, 34.32, 31.52, 29.93, 28.10, 27.21, 26.90, 23.85, 21.45, 21.17, 21.07, 18.32, 16.64, 16.31, 16.22, 14.93. IR (DRIFT): 2942.55, 2868.40, 1731.53, 1592.49, 1243.38, 1149.33, 1029.23. HRMS (ASAP, TOF ES^+^): calcd. for C_41_H_61_O_6_^+^ [M + H]^+^ 649.4463; found 649.4459.

##### 3β,28-Diacetoxy-30-(2-methoxyphenoxy)­lup-20­(29)-ene (**9**)

Compound **9** was prepared according to the general procedure for the Mitsunobu reaction. The reaction time was 1 h. After the column chromatography purification using hexane/EtOAc (6:1), derivative **9** was obtained in 76% yield as a white solid. ^1^H NMR (500 MHz, CDCl_3_): δ 6.94 – 6.87 (m, 4H, aryl), 5.09 (d, *J* = 1.0 Hz, 1H, H-29), 5.00 (s, 1H, H-29), 4.56 (d, *J* = 13.5 Hz, 1H, H-30), 4.53 (d, *J* = 13.5 Hz, 1H, H-30), 4.50 – 4.44 (m, 1H, H-3), 4.26 (dd, *J* = 11.1, 0.9 Hz, 1H, H-28), 3.89 – 3.83 (m, 4H, H-28, CH_3_O), 2.43 (td, *J* = 11.2, 5.4 Hz, 1H, H-19), 2.07 (s, 3H, CH_3_C=O), 2.04 (s, 3H, CH_3_C=O), 1.04 (s, 3H, CH_3_), 0.96 (s, 3H, CH_3_), 0.85 (s, 3H, CH_3_), 0.84 (s, 3H, CH_3_), 0.84 (s, 3H, CH_3_). ^13^C NMR (126 MHz, CDCl_3_): δ 171.70, 171.13, 149.76, 149.65, 148.59, 121.38, 120.88, 113.83, 112.07, 110.11, 81.06, 71.33, 62.69, 56.06, 55.54, 50.42, 49.65, 46.48, 43.44, 42.82, 41.09, 38.54, 37.96, 37.67, 37.22, 34.50, 34.31, 31.50, 29.92, 28.10, 27.21, 26.87, 23.84, 21.46, 21.17, 21.07, 18.32, 16.64, 16.30, 16.21, 14.86. IR (DRIFT): 2941.55, 2870.45, 1731.73, 1504.59, 1241.33, 1028.13. HRMS (ASAP, TOF ES^+^): calcd. for C_41_H_61_O_6_^+^ [M + H]^+^ 649.4463; found 649.4426.

##### 3β,28-Diacetoxy-30-(4-formylphenoxy)­lup-20­(29)-ene (**10**)

Compound **10** was prepared according to the general procedure for the Mitsunobu reaction. The reaction time was 1 h. After the column chromatography purification using hexane/EtOAc (6:1), derivative **10** was obtained in 69% yield as a white solid. ^1^H NMR (500 MHz, CDCl_3_): δ 9.89 (s, 1H, CHO), 7.85 – 7.82 (m, 2H, aryl), 7.03 – 7.00 (m, 2H, aryl), 5.09 (d, *J* = 0.5 Hz, 1H, H-29), 5.06 (s, 1H, H-29), 4.56 (s, 2H, H-30), 4.49 – 4.44 (m, 1H, H-3), 4.25 (dd, *J* = 11.1, 1.0 Hz, 1H, H-28), 3.86 (d, *J* = 11.1 Hz, 1H, H-28), 2.44 (td, *J* = 11.3, 5.5 Hz, 1H, H-19), 2.07 (s, 3H, CH_3_C=O), 2.04 (s, 3H, CH_3_C=O), 1.04 (s, 3H, CH_3_), 0.97 (s, 3H, CH_3_), 0.85 (s, 3H, CH_3_), 0.84 (s, 3H, CH_3_), 0.84 (s, 3H, CH_3_). ^13^C NMR (126 MHz, CDCl_3_) δ 190.88, 171.71, 171.13, 163.94, 149.04, 132.11, 130.24, 115.13, 111.37, 81.01, 70.95, 62.58, 55.54, 50.42, 49.78, 46.51, 43.37, 42.84, 41.10, 38.56, 37.95, 37.66, 37.22, 34.50, 34.32, 31.68, 29.93, 28.09, 27.17, 27.09, 23.83, 21.45, 21.16, 21.08, 18.31, 16.63, 16.32, 16.21, 14.91. IR (DRIFT): 2943.41, 2870.45, 1731.85, 1693.48, 1599.25, 1240.81, 1157.89, 1028.95. HRMS (ASAP, TOF ES^+^): calcd. for C_41_H_59_O_6_^+^ [M + H]^+^ 647.4306; found 647.4312.

##### 3β,28-Diacetoxy-30-(3-formylphenoxy)­lup-20­(29)-ene (**11**)

Compound **11** was prepared according to the general procedure for the Mitsunobu reaction. The reaction time was 1 h. After the column chromatography purification using hexane/EtOAc (6:1), derivative **11** was obtained as in 86% yield a white solid. ^1^H NMR (500 MHz, CDCl_3_): δ 9.98 (s, 1H, CHO), 7.47 – 7.43 (m, 2H, aryl), 7.42 – 7.39 (m, 1H, aryl), 7.20 (dt, *J* = 6.6, 2.6 Hz, 1H, aryl), 5.09 (d, *J* = 0.9 Hz, 1H, H-29), 5.05 (s, 1H, H-29), 4.54 (s, 2H, H-30), 4.49 – 4.44 (m, 1H, H-3), 4.26 (dd, *J* = 11.1, 1.0 Hz, 1H, H-28), 3.85 (d, *J* = 11.0 Hz, 1H, H-28), 2.44 (td, *J* = 11.3, 5.4 Hz, 1H, H-19), 2.07 (s, 3H, CH_3_C=O), 2.04 (s, 3H, CH_3_C=O), 1.04 (s, 3H, CH_3_), 0.97 (s, 3H, CH_3_), 0.85 (s, 3H, CH_3_), 0.84 (s, 3H, CH_3_), 0.84 (s, 3H, CH_3_). ^13^C NMR (126 MHz, CDCl_3_): δ 192.18, 171.72, 171.13, 159.52, 149.35, 137.98, 130.21, 123.78, 122.25, 113.14, 111.09, 81.04, 70.82, 62.62, 55.53, 50.41, 49.76, 46.52, 43.48, 42.85, 41.10, 38.54, 37.96, 37.68, 37.23, 34.52, 34.33, 31.64, 29.93, 28.10, 27.19, 27.06, 23.84, 21.45, 21.17, 21.08, 18.32, 16.64, 16.32, 16.22, 14.93. IR (DRIFT): 2941.9, 2868.2, 1730.8, 1698.3, 1595.6, 1240.0, 1166.70, 1028.9. HRMS (ASAP, TOF ES^+^): calcd. for C_41_H_59_O_6_^+^ [M + H]^+^ 647.4306; found 647.4315.

##### 3β,28-Diacetoxy-30-(2-formylphenoxy)­lup-20­(29)-ene (**12**)

Compound **12** was prepared according to the general procedure for the Mitsunobu reaction. The reaction time was 1 h. After the column chromatography purification using hexane/EtOAc (6:1), derivative **12** was obtained in 67% yield as a white solid. ^1^H NMR (500 MHz, CDCl_3_): δ 10.53 (d, *J* = 0.7 Hz, 1H, CHO), 7.85 (dd, *J* = 7.7, 1.8 Hz, 1H, aryl), 7.54 (ddd, *J* = 8.4, 7.3, 1.9 Hz, 1H, aryl), 7.04 (t, *J* = 7.5 Hz, 1H, aryl), 6.99 (d, *J* = 8.3 Hz, 1H, aryl), 5.13 (s, 1H, H-29), 5.08 (s, 1H, H-29), 4.61 – 4.55 (m, 2H, H-30), 4.49 – 4.44 (m, 1H, H-3), 4.26 (dd, *J* = 11.2, 1.1 Hz, 1H, H-28), 3.84 (d, *J* = 11.0 Hz, 1H, H-28), 2.43 (td, *J* = 11.2, 5.4 Hz, 1H, H-19), 2.07 (s, 3H, CH_3_C=O), 2.04 (s, 3H, CH_3_C=O), 1.04 (s, 3H, CH_3_), 0.98 (s, 3H, CH_3_), 0.85 (s, 3H, CH_3_), 0.84 (s, 3H, CH_3_), 0.84 (s, 3H, CH_3_). ^13^C NMR (126 MHz, CDCl_3_): δ 189.74, 171.68, 171.14, 161.27, 149.18, 136.02, 128.66, 125.22, 121.03, 112.74, 110.97, 81.02, 71.31, 62.54, 55.54, 50.42, 49.94, 46.51, 43.24, 42.84, 41.10, 38.55, 37.96, 37.65, 37.23, 34.52, 34.32, 31.95, 29.95, 28.10, 27.25, 27.18, 23.83, 21.45, 21.16, 21.08, 18.31, 16.64, 16.32, 16.21, 14.91. IR (DRIFT): 2940.03, 2866.35, 1732.08, 1688.59, 1598.48, 1238.99, 1028.88. HRMS (ASAP, TOF ES^+^): calcd. for C_41_H_59_O_6_^+^ [M + H]^+^ 647.4306; found 647.4304.

##### 3β,28-Diacetoxy-30-(4-acetylphenoxy)­lup-20­(29)-ene (**13**)

Compound **13** was prepared according to the general procedure for the Mitsunobu reaction. The reaction time was 1 h. After the column chromatography purification using hexane/EtOAc (4:1) derivative **13** was obtained in 61% yield as a white solid. ^1^H NMR (500 MHz, CDCl_3_): δ 7.94 – 7.91 (m, 2H, aryl), 6.95 – 6.92 (m, 2H, aryl), 5.07 (d, *J* = 0.8 Hz, 1H, H-29), 5.04 (s, 1H, H-29), 4.53 (s, 2H, H-30), 4.48 – 4.43 (m, 1H), 4.24 (dd, *J* = 11.1, 0.9 Hz, 1H, H-28), 3.84 (d, *J* = 11.1 Hz, 1H, H-28), 2.54 (s, 3H, CH_3_C = O), 2.47 – 2.38 (m, 1H, H-19), 2.06 (s, 3H, CH_3_C=O), 2.02 (s, 3H, CH_3_C=O), 1.03 (s, 3H, CH_3_), 0.96 (s, 3H, CH_3_), 0.84 (s, 3H, CH_3_), 0.83 (s, 3H, CH_3_), 0.82 (s, 3H, CH_3_). ^13^C NMR (126 MHz, CDCl_3_) δ 196.79, 171.65, 171.07, 162.79, 149.16, 130.68, 130.58, 114.47, 111.20, 80.97, 70.72, 62.54, 55.49, 50.38, 49.70, 46.46, 43.40, 42.80, 41.05, 38.52, 37.90, 37.63, 37.18, 34.46, 34.28, 31.61, 29.88, 28.05, 27.14, 27.00, 26.44, 23.79, 21.40, 21.12, 21.05, 18.27, 16.59, 16.28, 16.17, 14.87. IR (DRIFT): 2942.08, 2868.40, 1731.66, 1677.62, 1598.92, 1239.40, 1169.40, 1028.87. HRMS (ASAP, TOF ES^+^): calcd. for C_42_H_61_O_6_^+^ [M + H]^+^ 661.4463; found 661.4459.

##### 3β,28-Diacetoxy-30-(3-acetylphenoxy)­lup-20­(29)-ene (**14**)

Compound **14** was prepared according to the general procedure for the Mitsunobu reaction. The reaction time was 1 h. After the column chromatography purification using hexane/EtOAc (6:1), derivative **14** was obtained in 66% yield as a white solid. ^1^H NMR (500 MHz, CDCl_3_): δ 7.55 – 7.52 (m, 1H, aryl), 7.50 (dd, *J* = 2.7, 1.5 Hz, 1H, aryl), 7.37 (t, *J* = 7.9 Hz, 1H, aryl), 7.13 (ddd, *J* = 8.2, 2.6, 1.0 Hz, 1H, aryl), 5.09 (d, *J* = 0.9 Hz, 1H, H-29), 5.04 (s, 1H, H-29), 4.53 (s, 2H, H-30), 4.50 – 4.44 (m, 1H, H-3), 4.26 (dd, *J* = 11.1, 1.1 Hz, 1H, H-28), 3.85 (d, *J* = 11.0 Hz, 1H, H-28), 2.59 (s, 3H, CH_3_C = O), 2.44 (td, *J* = 10.8, 4.9 Hz, 1H, H-19), 2.07 (s, 3H, CH_3_C=O), 2.03 (s, 3H, CH_3_C=O), 1.04 (s, 3H, CH_3_), 0.97 (s, 3H, CH_3_), 0.85 (s, 3H, CH_3_), 0.84 (s, 3H, CH_3_), 0.83 (s, 3H, CH_3_). ^13^C NMR (126 MHz, CDCl_3_) δ 197.99, 171.71, 171.12, 159.17, 149.53, 138.66, 129.71, 121.38, 120.41, 113.39, 110.93, 81.04, 70.74, 62.63, 55.52, 50.41, 49.73, 46.50, 43.48, 42.84, 41.09, 38.53, 37.95, 37.68, 37.22, 34.51, 34.32, 31.63, 29.93, 28.09, 27.19, 27.03, 26.87, 23.84, 21.45, 21.17, 21.08, 18.31, 16.63, 16.31, 16.21, 14.92. IR (DRIFT): 2942.96, 2872.49, 1732.03, 1685.76, 1581.07, 1241.41, 1029.21. HRMS (ASAP, TOF ES^+^): calcd. for C_42_H_61_O_6_^+^ [M + H]^+^ 661.4463; found 661.4427.

##### 3β,28-Diacetoxy-30-(2-acetylphenoxy)­lup-20­(29)-ene (**15**)

Compound **15** was prepared according to the general procedure for the Mitsunobu reaction. The reaction time was 1 h. After the column chromatography purification using hexane/EtOAc (3:1), derivative **15** was obtained in 75% yield as a white solid. ^1^H NMR (500 MHz, CDCl_3_): δ 7.70 (dd, *J* = 7.7, 1.8 Hz, 1H, aryl), 7.43 (ddd, *J* = 8.3, 7.3, 1.9 Hz, 1H, aryl), 7.00 (td, *J* = 7.5, 0.9 Hz, 1H, aryl), 6.97 (d, *J* = 8.3 Hz, 1H, aryl), 5.13 (s, 1H, H-29), 5.07 (s, 1H, H-29), 4.60 – 4.54 (m, 2H, H-30), 4.49 – 4.44 (m, 1H, H-3), 4.26 (dd, *J* = 11.1, 1.1 Hz, 1H, H-28), 3.83 (d, *J* = 11.1 Hz, 1H, H-28), 2.64 (s, 3H, CH_3_C = O), 2.41 (td, *J* = 11.2, 5.5 Hz, 1H, H-19), 2.07 (s, 3H, CH_3_C=O), 2.04 (s, 3H, CH_3_C=O), 1.04 (s, 3H, CH_3_), 0.99 (s, 3H, CH_3_), 0.85 (s, 3H, CH_3_), 0.85 (s, 3H, CH_3_), 0.84 (s, 3H, CH_3_). ^13^C NMR (126 MHz, CDCl_3_): δ 200.21, 171.67, 171.15, 158.15, 149.31, 133.55, 130.49, 129.05, 120.98, 112.98, 110.64, 81.01, 71.34, 62.51, 55.52, 50.38, 49.96, 46.52, 43.23, 42.83, 41.08, 38.54, 37.95, 37.61, 37.21, 34.48, 34.30, 32.00, 31.78, 29.92, 28.09, 27.24, 27.17, 23.82, 21.46, 21.17, 21.05, 18.30, 16.64, 16.31, 16.19, 14.91. IR (DRIFT): 2942.65, 2874.54, 1731.82, 1674.77, 1596.84, 1232.03, 1028.31. HRMS (ASAP, TOF ES^+^): calcd. for C_42_H_61_O_6_^+^ [M + H]^+^ 661.4463; found 661.4459.

##### 3β,28-Diacetoxy-30-(4-methoxycarbonylphenoxy)­lup-20­(29)-ene (**16**)

Compound **16** was prepared according to the general procedure for the Mitsunobu reaction. The reaction time was 1 h. After the column chromatography purification using hexane/EtOAc (6:1), derivative **16** was obtained in 82% yield as a white solid. ^1^H NMR (500 MHz, CDCl_3_): δ 8.01 – 7.97 (m, 2H, aryl), 6.95 – 6.91 (m, 2H, aryl), 5.08 (d, *J* = 0.8 Hz, 1H, H-29), 5.04 (s, 1H, H-29), 4.53 (s, 2H, H-30), 4.49 – 4.44 (m, 1H, H-3), 4.25 (dd, *J* = 11.1, 1.0 Hz, 1H, H-28), 3.88 (s, 3H, CH_3_O), 3.85 (d, *J* = 11.1 Hz, 1H, H-28), 2.43 (td, *J* = 11.2, 5.4 Hz, 1H, H-19), 2.07 (s, 3H, CH_3_C=O), 2.04 (s, 3H, CH_3_C=O), 1.04 (s, 3H, CH_3_), 0.97 (s, 3H, CH_3_), 0.85 (s, 3H, CH_3_), 0.85 (s, 3H, CH_3_), 0.84 (s, 3H, CH_3_). ^13^C NMR (126 MHz, CDCl_3_) δ 171.72, 171.15, 166.99, 162.68, 149.27, 131.73, 122.90, 114.46, 111.17, 81.04, 70.73, 62.62, 55.54, 52.00, 50.42, 49.74, 46.51, 43.43, 42.85, 41.10, 38.56, 37.96, 37.68, 37.23, 34.51, 34.33, 31.66, 29.93, 28.10, 27.19, 27.04, 23.84, 21.46, 21.17, 21.09, 18.32, 16.64, 16.32, 16.22, 14.91. IR (DRIFT): 2944.18, 2870.45, 1719.95, 1604.96, 1509.22, 1240.56, 1166.65, 1104.39, 1028.98. HRMS (ASAP, TOF ES^+^): calcd. for C_42_H_61_O_7_^+^ [M + H]^+^ 677.4412; found 677.4384.

##### 3β,28-Diacetoxy-30-(3-methoxycarbonylphenoxy)­lup-20­(29)-ene (**17**)

Compound **17** was prepared according to the general procedure for the Mitsunobu reaction. The reaction time was 1 h. After the column chromatography purification using hexane/EtOAc (6:1), derivative **17** was obtained in 91% yield as a white solid. ^1^H NMR (500 MHz, CDCl_3_): δ 7.63 (d, *J* = 7.7 Hz, 1H, aryl), 7.57 (s, 1H, aryl), 7.34 (t, *J* = 7.9 Hz, 1H, aryl), 7.15 – 7.08 (m, 1H, aryl), 5.09 (s, 1H, H-29), 5.03 (s, 1H, H-29), 4.52 (s, 2H, H-30), 4.49 – 4.44 (m, 1H, H-3), 4.26 (d, *J* = 11.1 Hz, 1H, H-28), 3.91 (s, 3H, CH_3_O), 3.85 (d, *J* = 11.3 Hz, 1H, H-28), 2.44 (td, *J* = 10.8, 5.2 Hz, 1H, H-19), 2.07 (s, 3H, CH_3_C=O), 2.04 (s, 3H, CH_3_C=O), 1.04 (s, 3H, CH_3_), 0.97 (s, 3H, CH_3_), 0.85 (s, 3H, CH_3_), 0.84 (s, 3H, CH_3_), 0.84 (s, 3H, CH_3_). ^13^C NMR (126 MHz, CDCl_3_): δ 171.72, 171.13, 167.09, 158.91, 149.53, 131.59, 129.54, 122.27, 120.28, 115.03, 110.89, 81.05, 70.76, 62.65, 55.53, 52.32, 50.41, 49.72, 46.50, 43.48, 42.85, 41.10, 38.54, 37.95, 37.68, 37.22, 34.51, 34.32, 31.63, 29.94, 28.10, 27.20, 27.02, 23.84, 21.45, 21.17, 21.08, 18.32, 16.64, 16.32, 16.22, 14.92. IR (DRIFT): 2944.46, 2872.49, 1724.98, 1585.16, 1241.05, 1029.46. HRMS (ASAP, TOF ES^+^): calcd. for C_42_H_61_O_7_^+^ [M + H]^+^ 677.4412; found 677.4413.

##### 3β,28-Diacetoxy-30-(2-methoxycarbonylphenoxy)­lup-20­(29)-ene (**18**)

Compound **18** was prepared according to the general procedure for the Mitsunobu reaction. The reaction time was 1 h. After the column chromatography purification using hexane/EtOAc (6:1), derivative **18** was obtained in 87% yield as a white solid. ^1^H NMR (500 MHz, CDCl_3_): δ 7.80 (dd, *J* = 7.7, 1.6 Hz, 1H, aryl), 7.47 – 7.42 (m, 1H, aryl), 7.00 – 6.95 (m, 2H, aryl), 5.25 (s, 1H, H-29), 5.04 (s, 1H, H-29), 4.54 (s, 2H, H-30), 4.50 – 4.43 (m, 1H, H-3), 4.26 (d, *J* = 11.0 Hz, 1H, H-28), 3.89 (s, 3H, CH_3_O), 3.85 (d, *J* = 11.2 Hz, 1H, H-28), 2.43 (td, *J* = 11.0, 5.4 Hz, 1H, H-19), 2.07 (s, 3H, CH_3_C=O), 2.04 (s, 3H, CH_3_C=O), 1.04 (s, 3H, CH_3_), 0.98 (s, 3H, CH_3_), 0.85 (s, 3H, CH_3_), 0.84 (s, 3H, CH_3_), 0.83 (s, 3H, CH_3_). ^13^C NMR (126 MHz, CDCl_3_) δ 171.69, 171.13, 167.05, 158.26, 149.16, 133.46, 131.93, 120.72, 120.49, 113.33, 110.09, 81.04, 71.01, 62.62, 55.54, 52.14, 50.41, 49.68, 46.51, 42.84, 41.10, 38.53, 37.96, 37.65, 37.22, 34.49, 34.32, 31.59, 29.94, 29.85, 28.10, 27.20, 26.99, 23.84, 21.45, 21.17, 21.07, 18.32, 16.64, 16.31, 16.21, 14.89. IR (DRIFT): 2940.55, 2868.40, 1725.84, 1599.49, 1237.50, 1082.77, 1029.78. HRMS (ASAP, TOF ES^+^): calcd. for C_42_H_61_O_7_^+^ [M + H]^+^ 677.4412; found 677.4404.

##### 3β,28-Diacetoxy-30-(4-cyanophenoxy)­lup-20­(29)-ene (**19**)

Compound **19** was prepared according to the general procedure for the Mitsunobu reaction. The reaction time was 1 h. After the column chromatography purification using hexane/EtOAc (6:1), derivative **19** was obtained in 53% yield as a white solid. ^1^H NMR (500 MHz, CDCl_3_): δ 7.60 – 7.57 (m, 2H, aryl), 6.99 – 6.94 (m, 2H, aryl), 5.07 (s, 1H, H-29), 5.06 (s, 1H, H-29), 4.52 (s, 2H, H-30), 4.49 – 4.44 (m, 1H, H-3), 4.24 (d, *J* = 10.9 Hz, 1H, H-28), 3.85 (d, *J* = 11.0 Hz, 1H, H-28), 2.42 (td, *J* = 11.0, 5.3 Hz, 1H, H-19), 2.07 (s, 3H, CH_3_C=O), 2.04 (s, 3H, CH_3_C=O), 1.04 (s, 3H, CH_3_), 0.97 (s, 3H, CH_3_), 0.85 (s, 3H, CH_3_), 0.85 (s, 3H, CH_3_), 0.84 (s, 3H, CH_3_). ^13^C NMR (126 MHz, CDCl_3_): δ 171.73, 171.16, 162.18, 148.87, 134.14, 119.32, 115.57, 111.50, 104.31, 81.02, 71.00, 62.56, 55.54, 50.42, 49.81, 46.51, 43.28, 42.84, 41.10, 38.57, 37.96, 37.65, 37.22, 34.49, 34.33, 31.69, 29.92, 29.85, 28.09, 27.16, 27.13, 23.83, 21.45, 21.16, 21.08, 18.31, 16.63, 16.32, 16.21, 14.91, 14.26. IR (DRIFT): 2939.50, 2866.35, 2223.71, 1731.91, 1604.14, 1237.93, 1029.09. HRMS (ASAP, TOF ES^+^): calcd. for C_41_H_58_NO_5_^+^ [M + H]^+^ 644.4310; found 644.4272.

##### 3β,28-Diacetoxy-30-(3-cyanophenoxy)­lup-20­(29)-ene (**20**)

Compound **20** was prepared according to the general procedure for the Mitsunobu reaction. The reaction time was 1 h. After the column chromatography purification using hexane/EtOAc (6:1), derivative **20** was obtained in 80% yield as a white solid. ^1^H NMR (500 MHz, CDCl_3_): δ 7.39 – 7.35 (m, 1H, aryl), 7.26 – 7.22 (m, 1H, aryl), 7.18 – 7.13 (m, 2H, aryl), 5.07 (s, 1H, H-29), 5.06 (s, 1H, H-29), 4.51 – 4.44 (m, 3H, H-3, H-30), 4.25 (d, *J* = 11.1 Hz, 1H, H-28), 3.85 (d, *J* = 11.1 Hz, 1H, H-28), 2.42 (td, *J* = 11.0, 5.5 Hz, 1H, H-19), 2.07 (s, 3H, CH_3_C=O), 2.04 (s, 3H, CH_3_C=O), 1.04 (s, 3H, CH_3_), 0.97 (s, 3H, CH_3_), 0.85 (s, 3H, CH_3_), 0.85 (s, 3H, CH_3_), 0.84 (s, 3H, CH_3_). ^13^C NMR (126 MHz, CDCl_3_): δ 171.72, 171.14, 159.00, 149.00, 130.48, 124.81, 120.19, 118.85, 117.76, 113.39, 111.31, 81.03, 71.03, 62.57, 55.53, 50.41, 49.81, 46.51, 43.32, 42.84, 41.10, 38.54, 37.95, 37.65, 37.22, 34.51, 34.32, 31.68, 29.93, 29.84, 28.10, 27.17, 23.84, 21.45, 21.17, 21.07, 18.31, 16.64, 16.32, 16.21, 14.92. IR (DRIFT): 2942.68, 2872.49, 2229.85, 1730.24, 1579.02, 1240.12, 1028.64. HRMS (ASAP, TOF ES^+^): calcd. for C_41_H_58_NO_5_^+^ [M + H]^+^ 644.4310; found 644.4279.

##### 3β,28-Diacetoxy-30-(2-cyanophenoxy)­lup-20­(29)-ene (**21**)

Compound **21** was prepared according to the general procedure for the Mitsunobu reaction. The reaction time was 24 h. After the column chromatography purification using hexane/EtOAc (6:1), derivative **21** was obtained in 66% yield as a white solid. ^1^H NMR (500 MHz, CDCl_3_): δ 7.57 (dd, *J* = 7.7, 1.7 Hz, 1H, aryl), 7.51 (ddd, *J* = 8.5, 7.5, 1.7 Hz, 1H, aryl), 7.01 (td, *J* = 7.6, 0.9 Hz, 1H, aryl), 6.96 (d, *J* = 8.5 Hz, 1H, aryl), 5.16 (s, 1H, H-29), 5.08 (s, 1H, H-29), 4.61 – 4.55 (m, 2H, H-30), 4.50 – 4.43 (m, 1H, H-3), 4.27 (dd, *J* = 11.2, 1.2 Hz, 1H, H-28), 3.84 (d, *J* = 11.0 Hz, 1H, H-28), 2.43 (td, *J* = 11.2, 5.4 Hz, 1H, H-19), 2.07 (s, 3H, CH_3_C=O), 2.03 (s, 3H, CH_3_C=O), 1.04 (s, 3H, CH_3_), 0.97 (s, 3H, CH_3_), 0.85 (s, 3H, CH_3_), 0.84 (s, 3H, CH_3_), 0.83 (s, 3H, CH_3_). ^13^C NMR (126 MHz, CDCl_3_): δ 171.70, 171.15, 160.63, 148.51, 134.33, 134.01, 121.03, 116.52, 112.50, 111.37, 102.42, 81.04, 71.54, 62.59, 55.51, 50.37, 49.88, 46.49, 43.18, 42.82, 41.07, 38.51, 37.95, 37.59, 37.21, 34.54, 34.30, 31.81, 29.94, 28.09, 27.17, 27.11, 23.83, 21.46, 21.18, 21.03, 18.30, 16.64, 16.30, 16.19, 14.87. IR (DRIFT): 2943.32, 2870.45, 2227.81, 1731.16, 1597.44, 1239.42, 1028.15. HRMS (ASAP, TOF ES^+^): calcd. for C_41_H_58_NO_5_^+^ [M + H]^+^ 644.4310; found 644.4305.

##### 30-(4-Acetamidophenoxy)-3β,28-diacetoxylup-20­(29)-ene (**22**)

Compound **22** was prepared according to the general procedure for the Mitsunobu reaction. The reaction time was 1 h. After the column chromatography purification using hexane/EtOAc (1:1), derivative **22** was obtained in 67% yield as a white solid. ^1^H NMR (500 MHz, CDCl_3_): δ 7.38 (d, *J* = 8.9 Hz, 2H, aryl), 7.10 (s, 1H, Ac–N**H**-), 6.87 (d, *J* = 8.9 Hz, 2H, aryl), 5.06 (s, 1H, H-29), 5.01 (s, 1H, H-29), 4.48 – 4.44 (m, 3H, H-3, H-30), 4.25 (d, *J* = 11.0 Hz, 1H, H-28), 3.84 (d, *J* = 11.0 Hz, 1H, H-28), 2.42 (td, *J* = 10.9, 5.4 Hz, 1H, H-19), 2.15 (s, 3H, CH_3_C=O), 2.07 (s, 3H, CH_3_C=O), 2.04 (s, 3H, CH_3_C=O), 1.04 (s, 3H, CH_3_), 0.97 (s, 3H, CH_3_), 0.85 (s, 3H, CH_3_), 0.84 (s, 3H, CH_3_), 0.83 (s, 3H, CH_3_). ^13^C NMR (126 MHz, CDCl_3_) δ 171.74, 171.16, 168.20, 155.87, 149.78, 131.23, 121.92, 115.15, 110.75, 81.07, 70.76, 62.67, 55.53, 50.42, 49.65, 46.49, 43.64, 42.85, 41.09, 38.55, 37.96, 37.69, 37.23, 34.52, 34.32, 31.55, 29.93, 28.10, 27.20, 26.91, 24.54, 23.84, 21.46, 21.18, 21.08, 18.32, 16.64, 16.32, 16.21, 14.93. IR (DRIFT): 2943.56, 2870.45, 1732.37, 1667.86, 1539.54, 1508.46, 1234.88, 1029.09. HRMS (ASAP, TOF ES^+^): calcd. for C_42_H_62_NO_6_^+^ [M + H]^+^ 676.4572; found 676.4578.

##### 30-(3-Acetamidophenoxy)-3β,28-diacetoxylup-20­(29)-ene (**23**)

Compound **23** was prepared according to the general procedure for the Mitsunobu reaction. The reaction time was 1 h. After the column chromatography purification using hexane/EtOAc (1:1), derivative **23** was obtained in 78% yield as a white solid. ^1^H NMR (500 MHz, CDCl_3_): δ 7.29 (s, 1H, Ac–N**H**−), 7.19 (t, *J* = 8.2 Hz, 1H, aryl), 6.97 (d, *J* = 8.1 Hz, 1H, aryl), 6.67 (d, *J* = 7.8 Hz, 1H, aryl), 5.07 (s, 1H, H-29), 5.01 (s, 1H, H-29), 4.52 – 4.43 (m, 3H, H-3, H-30), 4.26 (d, *J* = 11.0 Hz, 1H, H-28), 3.84 (d, *J* = 11.0 Hz, 1H, H-28), 2.42 (td, *J* = 11.2, 5.4 Hz, 1H, H-19), 2.16 (s, 3H, CH_3_C=O), 2.07 (s, 3H, CH_3_C=O), 2.04 (s, 3H, CH_3_C=O), 1.03 (s, 3H, CH_3_), 0.97 (s, 3H, CH_3_), 0.85 (s, 3H, CH_3_), 0.84 (s, 3H, CH_3_), 0.83 (s, 3H, CH_3_). ^13^C NMR (126 MHz, CDCl_3_): δ 171.74, 171.17, 168.33, 159.55, 149.73, 139.27, 129.77, 112.15, 110.88, 106.51, 81.08, 70.62, 62.68, 55.52, 50.41, 49.63, 46.48, 43.54, 42.84, 41.08, 38.53, 37.95, 37.68, 37.22, 34.50, 34.31, 31.57, 29.92, 29.84, 28.09, 27.20, 26.93, 24.85, 23.84, 21.45, 21.18, 21.08, 18.31, 16.63, 16.31, 16.21, 14.92. IR (DRIFT): 2942.62, 2870.45, 1732.43, 1598.95, 1242.07, 1155.34, 1029.58. HRMS (ASAP, TOF ES^+^): calcd. for C_42_H_62_NO_6_^+^ [M + H]^+^ 676.4572; found 676.4570.

##### 30-(2-Acetamidophenoxy)-3β,28-diacetoxylup-20­(29)-ene (**24**)

Compound **24** was prepared according to the general procedure for the Mitsunobu reaction. The reaction time was 1 h. After the column chromatography purification using hexane/EtOAc (3:1) derivative **24** was obtained in 77% yield as a white solid. ^1^H NMR (500 MHz, CDCl_3_): δ 8.35 (d, *J* = 7.6 Hz, 1H, aryl), 7.76 (s, 1H, Ac–N**H**−), 7.03 – 6.94 (m, 2H, aryl), 6.87 (d, *J* = 8.0 Hz, 1H, aryl), 5.07 (s, 1H, H-29), 5.06 (s, 1H, H-29), 4.57 – 4.50 (m, 2H, H-30), 4.49 – 4.43 (m, 1H, H-3), 4.24 (d, *J* = 11.0 Hz, 1H, H-28), 3.86 (d, *J* = 11.1 Hz, 1H, H-28), 2.42 (td, *J* = 11.3, 5.3 Hz, 1H, H-19), 2.18 (s, 3H, CH_3_C=O), 2.07 (s, 3H, CH_3_C=O), 2.04 (s, 3H, CH_3_C=O), 1.04 (s, 3H, CH_3_), 0.98 (s, 3H, CH_3_), 0.85 (s, 3H, CH_3_), 0.84 (s, 3H, CH_3_), 0.83 (s, 3H, CH_3_). ^13^C NMR (126 MHz, CDCl_3_): δ 171.64, 171.11, 168.11, 149.57, 147.06, 128.04, 123.69, 121.51, 120.07, 111.28, 110.76, 80.97, 71.53, 62.44, 55.52, 50.37, 49.83, 46.53, 43.30, 42.83, 41.08, 38.55, 37.94, 37.64, 37.21, 34.49, 34.29, 31.99, 29.91, 28.08, 27.22, 27.15, 25.07, 23.81, 21.43, 21.14, 21.11, 18.29, 16.63, 16.30, 16.19, 14.88. IR (DRIFT): 2943.93, 2866.35, 1731.75, 1694.79, 1601.54, 1241.40, 1028.97. HRMS (ASAP, TOF ES^+^): calcd. for C_42_H_62_NO_6_^+^ [M + H]^+^ 676.4572; found 676.4569.

##### 3β,28-Diacetoxy-30-(4-formyl-2-methoxyphenoxy)­lup-20­(29)-ene (**25**)

Compound **25** was prepared according to the general procedure for the Mitsunobu reaction. The reaction time was 1 h. After the column chromatography purification using hexane/EtOAc (3:1), derivative **25** was obtained in 78% yield as a white solid. ^1^H NMR (500 MHz, CDCl_3_): δ 9.85 (s, 1H, CHO), 7.43 – 7.40 (m, 2H, aryl), 6.97 – 6.94 (m, 1H, aryl), 5.08 (s, 1H, H-29), 5.05 (s, 1H, H-29), 4.66 – 4.59 (m, 2H, H-30), 4.49 – 4.44 (m, 1H, H-3), 4.24 (dd, *J* = 11.2, 1.1 Hz, 1H, H-28), 3.93 (s, 3H, CH_3_O), 3.85 (d, *J* = 11.0 Hz, 1H, H-28), 2.42 (td, *J* = 11.1, 5.2 Hz, 1H, H-19), 2.06 (s, 3H, CH_3_C=O), 2.03 (s, 3H, CH_3_C=O), 1.03 (s, 3H, CH_3_), 0.96 (s, 3H, CH_3_), 0.85 (s, 3H, CH_3_), 0.84 (s, 3H, CH_3_), 0.83 (s, 3H, CH_3_). ^13^C NMR (126 MHz, CDCl_3_): δ 191.01, 171.68, 171.12, 153.96, 150.12, 148.78, 130.36, 126.67, 112.04, 110.96, 109.50, 81.01, 71.49, 62.58, 56.15, 55.54, 50.41, 49.81, 46.49, 43.20, 42.81, 41.08, 38.55, 37.95, 37.64, 37.21, 34.46, 34.30, 31.64, 29.90, 28.09, 27.17, 27.02, 23.82, 21.44, 21.15, 21.07, 18.30, 16.63, 16.30, 16.19, 14.83. IR (DRIFT): 2941.56, 2868.40, 1731.39, 1682.94, 1585.65, 1232.79, 1133.54, 1027.40. HRMS (ASAP, TOF ES^+^): calcd. for C_42_H_61_O_7_^+^ [M + H]^+^ 677.4412; found 677.4416.

##### 3β,28-Diacetoxy-30-{4-[(2*E*)-(methoxycarbonyl)­vinyl]­phenoxy}­lup-20­(29)-ene (**26**)

Compound **26** was prepared according to the general procedure for the Mitsunobu reaction. The reaction time was 1 h. After the column chromatography purification using hexane/EtOAc (4:1), derivative **26** was obtained in 84% yield as a white solid. ^1^H NMR (500 MHz, CDCl_3_): δ 7.64 (d, *J* = 16.0 Hz, 1H, vinyl), 7.48 – 7.45 (m, 2H, aryl), 6.93 – 6.89 (m, 2H, aryl), 6.30 (d, *J* = 15.9 Hz, 1H, vinyl), 5.07 (d, *J* = 0.8 Hz, 1H, H-29), 5.03 (s, 1H, H-29), 4.50 (s, 2H, H-30), 4.48 – 4.44 (m, 1H, H-3), 4.25 (d, *J* = 11.1 Hz, 1H, H-28), 3.85 (d, *J* = 11.1 Hz, 1H, H-28), 3.78 (s, 3H, CH_3_O), 2.42 (td, *J* = 11.2, 5.3 Hz, 1H, H-19), 2.06 (s, 3H, CH_3_C=O), 2.03 (s, 3H, CH_3_C=O), 1.03 (s, 3H, CH_3_), 0.96 (s, 3H, CH_3_), 0.84 (s, 3H, CH_3_), 0.84 (s, 3H, CH_3_), 0.83 (s, 3H, CH_3_). ^13^C NMR (126 MHz, CDCl_3_) δ 171.68, 171.10, 167.86, 160.73, 149.37, 144.62, 129.82, 127.39, 115.48, 115.19, 111.06, 81.00, 70.64, 62.58, 55.50, 51.68, 50.39, 49.67, 46.47, 43.46, 42.81, 41.06, 38.53, 37.92, 37.64, 37.19, 34.48, 34.29, 31.58, 29.90, 29.81, 28.07, 27.16, 26.97, 23.80, 21.42, 21.14, 21.06, 18.28, 16.61, 16.29, 16.18, 14.89. IR (DRIFT): 2943.97, 2870.45, 1730.15, 1602.14, 1509.27, 1240.01, 1163.99, 1028.88. HRMS (ASAP, TOF ES^+^): calcd. for C_44_H_63_O_7_^+^ [M + H]^+^ 703.4568; found 703.4550.

##### 3β,28-Diacetoxy-30-(3-pyridyloxy)­lup-20­(29)-ene (**27**)

Compound **27** was prepared according to the general procedure for the Mitsunobu reaction. The reaction time was 1 h. After the column chromatography purification using hexane/EtOAc (3:1), derivative **27** was obtained in 44% yield as a white solid. ^1^H NMR (500 MHz, CDCl_3_): δ 8.33 (s, 1H, aryl), 8.24 – 8.21 (m, 1H, aryl), 7.23 – 7.21 (m, 2H, aryl), 5.09 (d, *J* = 0.8 Hz, 1H, H-29), 5.05 (s, 1H, H-29), 4.52 (s, 2H, H-30), 4.48 – 4.44 (m, 1H, H-3), 4.25 (dd, *J* = 11.1, 0.9 Hz, 1H, H-28), 3.85 (d, *J* = 11.1 Hz, 1H, H-28), 2.43 (td, *J* = 11.2, 5.4 Hz, 1H, H-19), 2.07 (s, 3H, CH_3_C=O), 2.03 (s, 3H, CH_3_C=O), 1.04 (s, 3H, CH_3_), 0.97 (s, 3H, CH_3_), 0.85 (s, 3H, CH_3_), 0.84 (s, 3H, CH_3_), 0.83 (s, 3H, CH_3_). ^13^C NMR (126 MHz, CDCl_3_): δ 171.70, 171.12, 155.14, 149.19, 142.28, 138.13, 123.98, 121.66, 111.20, 81.02, 70.94, 62.59, 55.52, 50.41, 49.77, 46.51, 42.84, 41.09, 38.55, 37.95, 37.65, 37.22, 34.50, 34.32, 31.65, 29.93, 29.84, 28.09, 27.17, 27.06, 23.83, 21.44, 21.16, 21.07, 18.31, 16.63, 16.31, 16.21, 14.90. IR (DRIFT): 2940.39, 2872.49, 1731.08, 1574.93, 1230.98, 1027.85. HRMS (ASAP, TOF ES^+^): calcd. for C_39_H_58_NO_5_^+^ [M + H]^+^ 620.4310; found 620.4305.

##### 3β,28-Diacetoxy-30-(4-allyl-2-methoxyphenoxy)­lup-20­(29)-ene (**28**)

Compound **28** was prepared according to the general procedure for the Mitsunobu reaction. The reaction time was 1 h. After the column chromatography purification using hexane/EtOAc (8:1), derivative **28** was obtained in 84% yield as a white solid. ^1^H NMR (500 MHz, CDCl_3_): δ 6.80 (d, *J* = 8.1 Hz, 1H, aryl), 6.72 – 6.67 (m, 2H, aryl), 5.96 (ddt, *J* = 16.7, 10.0, 6.7 Hz, 1H, vinyl), 5.10 – 5.04 (m, 3H, vinyl, H-29), 4.99 (s, 1H, H-29), 4.56 – 4.44 (m, 3H, H-3, H-30), 4.26 (d, *J* = 11.2 Hz, 1H, H-28), 3.88 – 3.83 (m, 4H, H-28, CH_3_O), 3.33 (d, *J* = 6.7 Hz, 2H, benzylic), 2.42 (td, *J* = 11.2, 5.3 Hz, 1H, H-19), 2.07 (s, 3H, CH_3_C=O), 2.04 (s, 3H, CH_3_C=O), 1.03 (s, 3H, CH_3_), 0.96 (s, 3H, CH_3_), 0.85 (s, 3H, CH_3_), 0.84 (s, 3H, CH_3_), 0.84 (s, 3H, CH_3_). ^13^C NMR (126 MHz, CDCl_3_): δ 171.70, 171.13, 149.75, 149.67, 146.91, 137.83, 133.26, 120.51, 115.74, 113.90, 112.57, 110.03, 81.07, 71.53, 62.69, 56.06, 55.54, 50.43, 49.61, 43.43, 42.82, 41.09, 39.98, 38.55, 37.96, 37.68, 37.22, 34.50, 34.32, 31.48, 29.92, 29.85, 28.10, 27.21, 26.85, 23.85, 21.46, 21.18, 21.08, 18.33, 16.65, 16.31, 16.21, 14.87.IR (DRIFT): 2940.42, 2868.40, 1731.77, 1509.98, 1231.69, 1028.76. HRMS (ASAP, TOF ES^+^): calcd. for C_44_H_65_O_6_^+^ [M + H]^+^ 689.4776; found 689.4749.

##### 3β,28-Diacetoxy-30-{4-[(2′S)-2′-((*tert*-butoxycarbonyl)­amino)-3-methoxy-3-oxopropyl]­phenoxy}­lup-20­(29)-ene (**29**)

Compound **29** was prepared according to the general procedure for the Mitsunobu reaction. The reaction time was 1 h. After the column chromatography purification using a gradient of hexane/EtOAc (4:1 to 3:1), derivative **29** was obtained in 89% yield as a white solid. ^1^H NMR (500 MHz, CDCl_3_): δ 7.05 – 7.01 (m, 2H, aryl), 6.86 – 6.82 (m, 2H, aryl), 5.07 (d, *J* = 0.9 Hz, 1H, H-29), 5.01 (s, 1H, H-29), 4.95 (d, *J* = 8.4 Hz, 1H), 4.59 – 4.50 (m, 1H), 4.49 – 4.42 (m, 3H, H-3, H-30), 4.26 (dd, *J* = 11.2, 0.9 Hz, 1H, H-28), 3.85 (d, *J* = 11.1 Hz, 1H, H-28), 3.71 (s, 3H, CH_3_O), 3.13 – 2.93 (m, 2H), 2.43 (td, *J* = 11.2, 5.3 Hz, 1H, H-19), 2.07 (s, 3H, CH_3_C=O), 2.04 (s, 3H, CH_3_C=O), 1.42 (s, 9H, 3 × CH_3_), 1.04 (s, 3H, CH_3_), 0.97 (s, 3H, CH_3_), 0.85 (s, 3H, CH_3_), 0.84 (s, 3H, CH_3_), 0.84 (s, 3H, CH_3_). ^13^C NMR (126 MHz, CDCl_3_) δ 172.58, 171.73, 171.15, 158.11, 155.25, 149.82, 130.42, 128.26, 114.90, 110.67, 81.06, 80.05, 70.44, 62.67, 55.54, 54.69, 52.32, 50.43, 49.68, 46.50, 43.66, 42.85, 41.10, 38.55, 37.96, 37.69, 37.23, 34.52, 34.33, 31.56, 29.92, 28.47, 28.10, 27.21, 26.90, 23.84, 21.45, 21.18, 21.08, 18.32, 16.64, 16.32, 16.22, 14.93. IR (DRIFT): 2944.34, 2866.35, 1729.29, 1510.19, 1237.29, 1164.44, 1027.47. HRMS (ASAP, TOF ES^+^): calcd. for C_49_H_74_NO_9_^+^ [M + H]^+^ 820.5358; found 820.5367.

##### Synthesis of 3β,28-Diacetoxy-30-[4-((2S)-2-amino-3-methoxy-3-oxopropyl)­phenoxy]­lup-20(29)-ene (**30**)

The reaction was performed under inert conditions. Compound **29** (200 mg, 0.244 mmol) was dissolved in TFA (500 μL) and DCM (500 μL). The reaction mixture was stirred at room temperature for 0.5 h. The reaction was determined to be complete by TLC analysis using mobile phase hexane/EtOAc (3:1). The reaction mixture was diluted with DCM (30 mL). The organic phase was washed three times with saturated solution of NaHCO_3_ (10 mL), once with H_2_O (10 mL), and once with brine (10 mL). The organic phase was dried over anhydrous MgSO_4_, and the organic solvent was subsequently removed under reduced pressure using a rotary evaporator, followed by column chromatography purification on SiO_2_ eluting with a gradient mobile phase of hexane/EtOAc (3:1 to 100% EtOAc). After the purification process derivative **30** was obtained in 68% yield as a white solid. ^1^H NMR (500 MHz, CDCl_3_): δ 7.09 (d, *J* = 8.1 Hz, 2H, aryl), 6.85 (d, *J* = 8.3 Hz, 2H, aryl), 5.06 (s, 1H, H-29), 5.00 (s, 1H, H-29), 4.50 – 4.40 (m, 3H, H-3, H-30), 4.25 (d, *J* = 10.9 Hz, 1H, H-28), 3.84 (d, *J* = 11.0 Hz, 1H, H-28), 3.75 – 3.65 (m, 5H), 3.01 (dd, *J* = 13.7, 5.1 Hz, 1H), 2.81 (dd, *J* = 13.7, 7.7 Hz, 1H), 2.47 – 2.38 (m, 1H, H-19), 2.06 (s, 3H, CH_3_C=O), 2.03 (s, 3H, CH_3_C=O), 1.03 (s, 3H, CH_3_), 0.96 (s, 3H, CH_3_), 0.84 (s, 3H, CH_3_), 0.84 (s, 3H, CH_3_), 0.83 (s, 3H, CH_3_). ^13^C NMR (126 MHz, CDCl_3_) δ 171.73, 171.14, 151.78, 148.42, 129.33, 117.52, 112.94, 108.42, 81.05, 62.70, 55.53, 50.42, 49.66, 47.98, 46.50, 44.62, 42.84, 41.10, 38.55, 37.96, 37.67, 37.23, 34.60, 34.32, 31.52, 29.93, 29.85, 28.10, 27.21, 27.07, 26.94, 23.84, 21.46, 21.18, 21.09, 18.32, 16.64, 16.32, 16.21, 14.91. IR (DRIFT): 2943.7, 2871.0, 1731.6, 1610.9, 1509.3, 1236.1, 1028.3. HRMS (ASAP, TOF ES^+^): calcd. for C_44_H_66_NO_7_^+^ [M + H]^+^ 720.4834; found 720.4825.

#### Synthesis of Ester Compounds **31**–**36**

##### 3β,28-Diacetoxy-30-(benzoyloxy)­lup-20­(29)-ene (**31**)

Compound **31** was prepared according to the general procedure for the Mitsunobu reaction. The reaction time was 1 h. After the column chromatography purification using hexane/EtOAc (8:1), derivative **31** was obtained in 88% yield as a white solid. ^1^H NMR (400 MHz, CDCl_3_): δ 8.08 – 8.05 (m, 2H, aryl), 7.59 – 7.54 (m, 1H, aryl), 7.47 – 7.42 (m, 2H, aryl), 5.06 (d, *J* = 0.9 Hz, 1H, H-29), 5.02 (s, 1H, H-29), 4.82 (d, *J* = 13.7 Hz, 1H, H-30), 4.78 (d, *J* = 13.5 Hz, 1H, H-30), 4.51 – 4.43 (m, 1H, H-3), 4.26 (dd, *J* = 11.1, 0.9 Hz, 1H, H-28), 3.83 (d, *J* = 11.0 Hz, 1H, H-28), 2.44 (td, *J* = 10.9, 5.3 Hz, 1H, H-19), 2.06 (s, 3H, CH_3_C=O), 2.04 (s, 3H, CH_3_C=O), 1.03 (s, 3H, CH_3_), 0.97 (s, 3H, CH_3_), 0.86 – 0.84 (m, 6H, 2 × CH_3_), 0.83 (s, 3H, CH_3_). ^13^C NMR (101 MHz, CDCl_3_) δ 171.68, 171.12, 166.42, 149.05, 133.17, 130.32, 129.77, 128.55, 110.83, 81.02, 66.76, 62.63, 55.49, 50.36, 49.78, 46.49, 44.00, 42.82, 41.06, 38.53, 37.94, 37.67, 37.20, 34.54, 34.27, 31.48, 29.91, 28.09, 27.17, 26.81, 23.83, 21.45, 21.15, 21.03, 18.30, 16.64, 16.29, 16.19, 14.90. IR (DRIFT): 2940.91, 2870.45, 1723.81, 1450.99, 1239.21, 1026.41. HRMS (ASAP, TOF ES^+^): calcd. for C_41_H_59_O_6_^+^ [M + H]^+^ 647.4306; found 647.4307.

##### 3β,28-Diacetoxy-30-(4-methoxybenzoyloxy)­lup-20­(29)-ene (**32**)

Compound **32** was prepared according to the general procedure for the Mitsunobu reaction. The reaction time was 1 h. After the column chromatography purification using hexane/EtOAc (5:1), derivative **32** was obtained in 80% yield as a white solid. ^1^H NMR (500 MHz, CDCl_3_): δ 8.04 – 7.99 (m, 2H, aryl), 6.95 – 6.91 (m, 2H, aryl), 5.05 (s, 1H, H-29), 5.01 (s, 1H, H-29), 4.79 (d, *J* = 13.6 Hz, 1H, H-30), 4.75 (d, *J* = 13.5 Hz, 1H, H-30), 4.50 – 4.42 (m, 1H, H-3), 4.26 (d, *J* = 10.9 Hz, 1H, H-28), 3.88 – 3.81 (m, 4H, H-28, CH_3_O), 2.43 (td, *J* = 10.6, 5.0 Hz, 1H, H-19), 2.06 (s, 3H, CH_3_C=O), 2.04 (s, 3H, CH_3_C=O), 1.03 (s, 3H, CH_3_), 0.97 (s, 3H, CH_3_), 0.85 (s, 6H, 2 × CH_3_), 0.83 (s, 3H, CH_3_). ^13^C NMR (126 MHz, CDCl_3_) δ 171.71, 171.13, 166.18, 163.60, 149.26, 131.81, 122.77, 113.83, 110.66, 81.06, 66.46, 62.68, 55.60, 55.52, 50.38, 49.76, 46.50, 44.04, 42.84, 41.08, 38.56, 37.96, 37.69, 37.22, 34.55, 34.29, 31.49, 29.93, 28.10, 27.20, 26.80, 23.85, 21.45, 21.17, 21.05, 18.32, 16.65, 16.30, 16.21, 14.93. IR (DRIFT): 2942.28, 2868.40, 1731.46, 1606.03, 1511.24, 1243.34, 1166.32, 1100.44, 1028.15. HRMS (ASAP, TOF ES^+^): calcd. for C_42_H_61_O_7_^+^ [M + H]^+^ 677.4412; found 677.4419.

##### 3β,28-Diacetoxy-30-(4-cyanobenzoyloxy)­lup-20­(29)-ene (**33**)

Compound **33** was prepared according to the general procedure for the Mitsunobu reaction. The reaction time was 24 h. After the column chromatography purification using hexane/EtOAc (3:1), derivative **33** was obtained in 42% yield as a white solid. ^1^H NMR (500 MHz, CDCl_3_): δ 8.17 – 8.14 (m, 2H, aryl), 7.78 – 7.74 (m, 2H, aryl), 5.05 (s, 2H, H-29), 4.85 (d, *J* = 13.6 Hz, 1H, H-30), 4.81 (d, *J* = 13.6 Hz, 1H, H-30), 4.49 – 4.44 (m, 1H, H-3), 4.24 (dd, *J* = 11.1, 1.0 Hz, 1H, H-28), 3.83 (d, *J* = 11.2 Hz, 1H, H-28), 2.41 (td, *J* = 10.9, 5.4 Hz, 1H, H-19), 2.06 (s, 3H, CH_3_C=O), 2.04 (s, 3H, CH_3_C=O), 1.03 (s, 3H, CH_3_), 0.98 (s, 3H, CH_3_), 0.85 (s, 3H, CH_3_), 0.85 (s, 3H, CH_3_), 0.84 (s, 3H, CH_3_). ^13^C NMR (126 MHz, CDCl_3_) δ 171.70, 171.14, 164.77, 148.63, 134.13, 132.45, 130.28, 118.08, 116.71, 111.09, 81.00, 67.74, 62.55, 55.53, 50.39, 49.91, 46.51, 43.75, 42.83, 41.09, 38.57, 37.96, 37.66, 37.22, 34.52, 34.30, 31.62, 29.91, 28.10, 27.16, 27.00, 23.83, 21.45, 21.16, 21.06, 18.31, 16.65, 16.32, 16.21, 14.91. IR (DRIFT): 2942.95, 2870.45, 2364.93, 1727.18, 1454.18, 1240.92, 1105.23, 1029.76. HRMS (ASAP, TOF ES^+^): calcd. for C_42_H_58_NO_6_^+^ [M + H]^+^ 672.4259; found 672.4255.

##### 3β,28-Diacetoxy-30-(2-furoyloxy)­lup-20­(29)-ene (**34**)

Compound **34** was prepared according to the general procedure for the Mitsunobu reaction. The reaction time was 1 h. After the column chromatography purification using hexane/EtOAc (3:1), derivative **34** was obtained in 60% yield as a white solid. ^1^H NMR (500 MHz, CDCl_3_): δ 7.59 (dd, *J* = 1.7, 0.9 Hz, 1H, aryl), 7.19 (dd, *J* = 3.5, 0.9 Hz, 1H, aryl), 6.52 (dd, *J* = 3.5, 1.7 Hz, 1H, aryl), 5.04 (d, *J* = 0.9 Hz, 1H, H-29), 5.01 (s, 1H, H-29), 4.80 (d, *J* = 13.5 Hz, 1H, H-30), 4.76 (d, *J* = 13.5 Hz, 1H, H-30), 4.49 – 4.45 (m, 1H, H-3), 4.25 (dd, *J* = 11.1, 1.1 Hz, 1H, H-28), 3.83 (d, *J* = 11.1 Hz, 1H, H-28), 2.42 (td, *J* = 10.8, 5.2 Hz, 1H, H-19), 2.06 (s, 3H, CH_3_C = O), 2.04 (s, 3H, CH_3_C = O), 1.03 (s, 3H, CH_3_), 0.96 (s, 3H, CH_3_), 0.85 (s, 3H, CH_3_), 0.84 (s, 3H, CH_3_), 0.83 (s, 3H, CH_3_). ^13^C NMR (126 MHz, CDCl_3_): δ 171.69, 171.12, 158.52, 148.65, 146.58, 144.78, 118.15, 112.02, 111.33, 81.03, 66.49, 62.64, 55.51, 50.38, 49.75, 46.50, 44.01, 42.82, 41.07, 38.55, 37.96, 37.68, 37.22, 34.52, 34.29, 31.36, 29.91, 28.10, 27.18, 26.75, 23.84, 21.45, 21.16, 21.05, 18.31, 16.65, 16.30, 16.20, 14.87. IR (DRIFT): 2944.27, 2870.45, 1728.78, 1581.07, 1472.66, 1230.61, 1176.99, 1114.27, 1029.12. HRMS (ASAP, TOF ES^+^): calcd. for C_39_H_57_O_7_^+^ [M + H]^+^ 637.4099; found 637.4103.

##### 3β,28-Diacetoxy-30-(butanoyloxy)­lup-20­(29)-ene (**35**)

Compound **35** was prepared according to the general procedure for the Mitsunobu reaction. The reaction time was 1 h. After the column chromatography purification using hexane/EtOAc (7:1), derivative **35** was obtained in 85% yield as a white solid. ^1^H NMR (500 MHz, CDCl_3_): δ 4.95 (s, 1H, H-29), 4.94 (d, *J* = 1.0 Hz, 1H, H-29), 4.58 (d, *J* = 13.6 Hz, 1H, H-30), 4.53 (d, *J* = 13.6 Hz, 1H, H-30), 4.50 – 4.44 (m, 1H, H-3), 4.24 (d, *J* = 11.1 Hz, 1H, H-28), 3.82 (d, *J* = 11.1 Hz, 1H, H-28), 2.41 – 2.30 (m, 3H, H-19, CH_2_), 2.07 (s, 3H, CH_3_C=O), 2.04 (s, 3H, CH_3_C=O), 1.03 (s, 3H, CH_3_), 0.98 – 0.95 (m, 6H, 2 × CH_3_), 0.87 – 0.84 (m, 6H, 2 × CH_3_), 0.83 (s, 3H, CH_3_). ^13^C NMR (126 MHz, CDCl_3_): δ 173.48, 171.72, 171.14, 149.09, 110.67, 81.04, 65.93, 62.62, 55.52, 50.40, 49.63, 46.48, 44.05, 42.82, 41.08, 38.55, 37.96, 37.67, 37.22, 36.42, 34.52, 34.30, 31.28, 29.91, 28.09, 27.18, 26.71, 23.84, 21.45, 21.17, 21.05, 18.57, 18.31, 16.64, 16.31, 16.20, 14.91, 13.89. IR (DRIFT): 2941.67, 2872.49, 1732.40, 1455.38, 1238.08, 1169.39, 1027.74 HRMS (ASAP, TOF ES^+^): calcd. for C_38_H_61_O_6_^+^ [M + H]^+^ 613.4463; found 613.4464.

##### 3β,28-Diacetoxy-30-{(2′S)-2′-[(*tert*-butoxycarbonyl)­amino]­propanoyloxy}­lup-20­(29)-ene (**36**)

Compound **36** was prepared according to the general procedure for the Mitsunobu reaction. The reaction time was 1 h. After the column chromatography purification using hexane/EtOAc (6:1), derivative **36** was obtained in 84% yield as a white solid. ^1^H NMR (500 MHz, CDCl_3_): δ 5.04 (d, *J* = 5.1 Hz, 1H, NH), 4.96 (s, 1H, H-29), 4.94 (s, 1H, H-29), 4.67 – 4.54 (m, 2H, H-30), 4.50 – 4.43 (m, 1H, H-3), 4.40 – 4.29 (m, 1H), 4.25 (d, *J* = 10.9 Hz, 1H, H-28), 3.81 (d, *J* = 11.1 Hz, 1H, H-28), 2.35 (td, *J* = 10.4, 5.2 Hz, 1H, H-19), 2.06 (s, 3H, CH_3_C=O), 2.03 (s, 3H, CH_3_C=O), 1.44 (s, 9H, 3 × CH_3_), 1.03 (s, 3H, CH_3_), 0.97 (s, 3H, CH_3_), 0.86 – 0.84 (m, 6H, 2 × CH_3_), 0.83 (s, 3H, CH_3_). ^13^C NMR (126 MHz, CDCl_3_): δ 173.18, 171.67, 171.13, 155.20, 148.51, 148.46, 110.75, 81.03, 80.00, 66.96, 62.56, 55.51, 50.37, 49.70, 49.46, 46.49, 43.83, 42.82, 41.07, 38.54, 37.95, 37.62, 37.22, 34.48, 34.29, 31.35, 31.29, 29.89, 28.48, 28.09, 27.17, 26.81, 23.83, 21.45, 21.16, 21.03, 18.91, 18.31, 16.64, 16.30, 16.19, 14.89. IR (DRIFT): 2940.00, 2868.40, 1732.58, 1507.39, 1239.49, 1159.59, 1028.02. HRMS (ASAP, TOF ES^+^): calcd. for C_42_H_68_NO_8_^+^ [M + H]^+^ 714.4939; found 714.4946.

##### Synthesis of 3β,28-Diacetoxy-30-[(2*S*)-2-aminopropanoyloxy]­lup-20­(29)-ene (**37**)

The reaction was performed under inert conditions. Compound **36** (174 mg, 0.244 mmol) was dissolved in TFA (500 μL) and DCM (500 μL). The reaction mixture was stirred at room temperature for 0.5 h. The reaction was determined to be complete by TLC analysis using mobile phase hexane/EtOAc (6:1). The reaction mixture was diluted with DCM (30 mL). The organic phase was washed three times with saturated solution of NaHCO_3_ (10 mL), once with H_2_O (10 mL) and once with brine (10 mL). The organic phase was dried over anhydrous MgSO_4_, and the organic solvent was subsequently removed under reduced pressure using a rotary evaporator, followed by column chromatography purification on SiO_2_ eluting with EtOAc. After the purification process, derivative **37** was obtained in 87% yield as a white solid. ^1^H NMR (500 MHz, CDCl_3_): δ 4.97 (s, 1H, H-29), 4.94 (d, *J* = 0.8 Hz, 1H, H-29), 4.63 – 4.55 (m, 2H, H-30), 4.49 – 4.44 (m, 1H, H-3), 4.24 (dd, *J* = 11.1, 1.0 Hz, 1H, H-28), 3.82 (d, *J* = 11.1 Hz, 1H, H-28), 3.59 (q, *J* = 7.1 Hz, 1H, R-C**H**-NH_2_), 2.39 – 2.31 (m, 1H, H-19), 2.07 (s, 3H, CH_3_C= O), 2.03 (s, 3H, CH_3_C=O), 1.36 (d, *J* = 7.0 Hz, 3H, CH_3_), 1.03 (s, 3H, CH_3_), 0.97 (s, 3H, CH_3_), 0.86 – 0.84 (m, 6H, 2 × CH_3_), 0.83 (s, 3H, CH_3_). ^13^C NMR (126 MHz, CDCl_3_): δ 176.43, 171.70, 171.13, 148.89, 110.58, 81.02, 66.65, 66.56, 62.57, 55.52, 50.39, 50.36, 49.74, 46.49, 42.82, 41.08, 38.55, 37.95, 37.64, 37.22, 34.50, 34.30, 31.42, 29.91, 28.09, 27.17, 26.86, 23.83, 21.45, 21.16, 21.05, 20.83, 18.31, 16.64, 16.31, 16.20, 14.90. IR (DRIFT): 2941.26, 2870.45, 1731.42, 1456.23, 1238.62, 1928.74. HRMS (ASAP, TOF ES^+^): calcd. for C_37_H_60_NO_6_^+^ [M + H]^+^ 614.4415; found 614.4418.

##### Synthesis of 3β,28-Diacetoxy-30-(phthalimido)­lup-20(29)-ene (**38**)

Compound **38** was prepared according to the general procedure for the Mitsunobu reaction. The reaction time was 24 h. After the column chromatography purification using hexane/EtOAc (3:1), derivative **38** was obtained in 70% yield as a white solid. ^1^H NMR (500 MHz, CDCl_3_): δ 7.88 – 7.84 (m, 2H, aryl), 7.75 – 7.70 (m, 2H, aryl), 4.88 (s, 1H, H-29), 4.63 (s, 1H, H-29), 4.51 – 4.45 (m, 1H, H-3), 4.32 – 4.24 (m, 2H, H-28, H-30), 4.17 (d, *J* = 16.2 Hz, 1H, H-30), 3.80 (d, *J* = 11.1 Hz, 1H, H-28), 2.40 (td, *J* = 10.6, 5.1 Hz, 1H, H-19), 2.06 (s, 3H, CH_3_C=O), 2.04 (s, 3H, CH_3_C=O), 1.03 (s, 3H, CH_3_), 0.98 (s, 3H, CH_3_), 0.85 (s, 3H, CH_3_), 0.84 (s, 3H, CH_3_), 0.83 (s, 3H, CH_3_). ^13^C NMR (126 MHz, CDCl_3_) δ 171.67, 171.11, 168.14, 148.31, 134.16, 132.22, 123.50, 108.76, 81.05, 62.70, 55.50, 50.36, 49.65, 46.51, 44.58, 42.85, 41.46, 41.07, 38.56, 37.96, 37.62, 37.24, 34.64, 34.28, 31.04, 29.96, 28.10, 27.19, 26.59, 23.86, 21.45, 21.16, 21.10, 18.32, 16.65, 16.29, 16.21, 14.89. IR (DRIFT): 2943.21, 2870.45, 1715.16, 1239.63, 1028.59. HRMS (ASAP, TOF ES^+^): calcd. for C_42_H_58_NO_6_^+^ [M + H]^+^ 672.4259; found 672.4260.

#### Synthesis of Sulfonamide Compounds **39** and **40**

##### 3β,28-Diacetoxy-30-[phenyl­(tosyl)­amino]­lup-20­(29)-ene (**39**)

Compound **39** was prepared according to the general procedure for the Mitsunobu reaction. The reaction time was 1 h. After the column chromatography purification using hexane/EtOAc (4:1), derivative **39** was obtained in 90% yield as a white solid. ^1^H NMR (500 MHz, CDCl_3_): δ 7.45 (d, *J* = 8.2 Hz, 2H, aryl), 7.29 – 7.21 (m, 6H, aryl), 7.08 – 7.04 (m, 2H, aryl), 4.85 (s, 1H, H-29), 4.82 (s, 1H, H-29), 4.48 – 4.43 (m, 1H, H-3), 4.26 (d, *J* = 11.0 Hz, 1H, H-28), 4.18 (d, *J* = 15.0 Hz, 1H, H-30), 4.08 (d, *J* = 15.0 Hz, 1H, H-30), 3.77 (d, *J* = 11.1 Hz, 1H, H-28), 2.42 (s, 3H, benzylic), 2.33 (dt, *J* = 11.4, 5.8 Hz, 1H, H-19), 2.07 (s, 3H, CH_3_C=O), 2.04 (s, 3H, CH_3_C=O), 1.00 (s, 3H, CH_3_), 0.89 (s, 3H, CH_3_), 0.83 (s, 9H, 3 × CH_3_). ^13^C NMR (126 MHz, CDCl_3_) δ 171.62, 171.14, 148.76, 143.55, 139.29, 135.29, 129.54, 128.74, 128.68, 127.89, 127.60, 112.07, 81.05, 62.80, 55.51, 50.32, 49.89, 46.46, 42.78, 41.01, 38.54, 37.94, 37.46, 37.16, 34.38, 34.28, 31.35, 30.01, 29.83, 28.08, 27.16, 26.88, 23.83, 21.68, 21.45, 21.18, 21.07, 18.28, 16.62, 16.29, 16.18, 14.82. IR (DRIFT): 2942.92, 2872.49, 1731.82, 1456.23, 1240.44, 1163.96, 1028.67. HRMS (ASAP, TOF ES^+^): calcd. for C_47_H_66_NO_6_S^+^ [M + H]^+^ 772.4605; found 772.4590.

##### 3β,28-Diacetoxy-30-[(4-nitrobenzenesulfonyl)­(phenyl)­amino]­lup-20­(29)-ene (**40**)

Compound **40** was prepared according to the general procedure for the Mitsunobu reaction. The reaction time was 1 h. After the column chromatography purification using hexane/EtOAc (4:1) derivative **40** was obtained in 72% yield as a white solid. ^1^H NMR (500 MHz, CDCl_3_): δ 8.32 – 8.27 (m, 2H, aryl), 7.75 – 7.71 (m, 2H, aryl), 7.33 – 7.27 (m, 3H, aryl), 7.07 – 7.03 (m, 2H, aryl), 4.88 (s, 2H, H-29), 4.50 – 4.41 (m, 1H, H-3), 4.28 – 4.20 (m, 2H, H-28, H-30), 4.14 (d, *J* = 15.0 Hz, 1H, H-30), 3.79 (d, *J* = 11.1 Hz, 1H, H-28), 2.39 – 2.29 (m, 1H, H-19), 2.07 (s, 3H, CH_3_C=O), 2.04 (s, 3H, CH_3_C=O), 1.00 (s, 3H, CH_3_), 0.89 (s, 3H, CH_3_), 0.83 (s, 6H, 2 × CH_3_), 0.83 (s, 3H, CH_3_). ^13^C NMR (126 MHz, CDCl_3_) δ 171.63, 171.15, 150.25, 148.17, 143.95, 138.37, 129.19, 128.97, 128.58, 128.31, 124.21, 112.56, 81.02, 62.72, 55.52, 50.32, 49.99, 46.50, 42.80, 41.02, 38.55, 37.94, 37.46, 37.16, 34.39, 34.28, 31.37, 30.02, 28.08, 27.13, 27.00, 23.83, 21.45, 21.17, 21.07, 18.28, 16.63, 16.30, 16.18, 14.84. IR (DRIFT): 2943.46, 2870.45, 1731.28, 1530.87, 1240.31, 1166.70, 1028.81. HRMS (ASAP, TOF ES^+^): calcd. for C_46_H_63_N_2_O_8_S^+^ [M + H]^+^ 803.4300; found 803.4308.

##### Synthesis of 3β,28-Diacetoxy-30-(phenylamino)­lup-20(29)-ene (**41**)

The reaction was performed under inert conditions. Compound **40** (270 mg, 0.336 mmol) was dissolved in anhydrous MeCN (5 mL). Thiophenol (86 μL, 0.84 mmol) and DBU (125 μL, 0.84 mmol) were then added into the reaction mixture. The reaction mixture was stirred at room temperature for 2 h. The reaction was determined to be complete by TLC analysis using mobile phase hexane/EtOAc (4:1). The reaction mixture was diluted with H_2_O (50 mL) and extracted three times with EtOAc (20 mL). The collected organic phase was washed once with brine (20 mL). The organic phase was dried over anhydrous MgSO_4_, and the organic solvent was subsequently removed under reduced pressure using a rotary evaporator, followed by column chromatography purification on SiO_2_ eluting with organic hexane/EtOAc (5:1). After the purification process, derivative **41** was obtained as in 85% yield a white solid. ^1^H NMR (500 MHz, CDCl_3_): δ 7.19 – 7.14 (m, 2H, aryl), 6.70 (tt, *J* = 7.3, 1.1 Hz, 1H, aryl), 6.62 – 6.58 (m, 2H, aryl), 4.93 (d, *J* = 1.3 Hz, 1H, H-29), 4.91 (s, 1H, H-29), 4.50 – 4.45 (m, 1H, H-3), 4.25 (dd, 1H, H-28), 3.90 – 3.78 (m, 2H, H-28, NH), 3.74 (d, *J* = 16.3 Hz, 1H, H-30), 3.68 (d, *J* = 16.0 Hz, 1H, H-30), 2.38 (td, *J* = 11.1, 5.4 Hz, 1H, H-19), 2.07 (s, 3H, CH_3_C=O), 2.04 (s, 3H, CH_3_C=O), 1.04 (s, 3H, CH_3_), 0.98 (s, 3H, CH_3_), 0.86 (s, 3H, CH_3_), 0.85 (s, 3H, CH_3_), 0.84 (s, 3H, CH_3_). ^13^C NMR (126 MHz, CDCl_3_) δ 171.73, 171.14, 151.79, 148.42, 129.33, 117.52, 112.94, 108.42, 81.05, 62.70, 55.53, 50.42, 49.67, 47.99, 46.50, 44.62, 42.84, 41.10, 38.55, 37.96, 37.68, 37.23, 34.60, 34.32, 31.52, 29.93, 28.10, 27.21, 26.95, 23.84, 21.46, 21.18, 21.09, 18.32, 16.64, 16.32, 16.21, 14.92. IR (DRIFT): 3410.5, 2942.7, 2869.9, 1731.0, 1602.4, 1241.0, 1028.8. HRMS (ASAP, TOF ES^+^): calcd. for C_40_H_60_NO_4_^+^ [M + H]^+^ 618.4517; found 618.4510.

### Biological Evaluation

#### Cell Culture and MTS Cytotoxicity Assay

Cytotoxicity screening was performed according to a standardized protocol routinely used and validated in our laboratory.^48,50^ Unless otherwise stated, all cell lines were obtained from the American Type Culture Collection (ATCC). The CCRF-CEM cell line (T lymphoblastic leukemia) served as a model of high chemoselectivity. K562 cells represent chronic myeloid leukemia with the BCR-ABL translocation, U2OS cells are derived from osteosarcoma, HCT116 cells originate from colorectal carcinoma, and their p53 knockdown derivative HCT116 p53^–/–^ (Horizon Discovery Ltd., UK) models p53-deficient tumors, which are commonly associated with poor prognosis. The A549 cell line represents lung adenocarcinoma. BJ and MRC-5 human fibroblasts were included as nontumor controls. All cells were cultured in appropriate media as recommended by ATCC or Horizon Discovery (DMEM or RPMI 1640 supplemented with 5 g/L glucose, 2 mM glutamine, 100 U/mL penicillin, 100 μg/mL streptomycin, 10% fetal calf serum, and NaHCO_3_) in 75 cm^2^ tissue culture flasks.^50^

MTS cytotoxicity assays were carried out in triplicate independent experiments following our established protocol, with cell viability quantified according to standard procedures.

#### Cell Cycle and Apoptosis Analysis

Cell cycle distribution and apoptosis (sub-G1 fraction) were assessed by flow cytometry. CCRF-CEM cells were seeded at 5 × 10^5^ cells/mL in six-well plates and treated for 24 h with either 1× or 5× IC_5_0 concentrations of the tested compounds. Cells were cultured in RPMI 1640 medium supplemented with 10% fetal calf serum, 100 U/mL penicillin, and 100 μg/mL streptomycin under standard conditions (37 °C, 5% CO_2_). A vehicle-treated control was included in parallel. Following treatment, cells were harvested, washed with ice-cold PBS, and fixed in 70% ethanol at −20 °C overnight. Fixed cells were washed with hypotonic citrate buffer, incubated with RNase A (50 μg/mL), and stained with propidium iodide for total DNA content analysis. Flow cytometric acquisition was performed on a FACSCalibur (Becton Dickinson) with a 488 nm excitation laser. Data were analyzed using Kaluza software (Beckman Coulter) to determine cell cycle phase distribution and quantify apoptotic sub-G1 populations. Half of the sample was processed for pH3Ser10 (Sigma) antibody staining and subsequent flow cytometric analysis of mitotic cells.^51^

#### Analysis of DNA Synthesis by BrdU Incorporation

Cells were cultured and treated under the same conditions as for cell cycle analysis. To assess DNA synthesis, cells were pulse-labeled with 10 μM 5-bromo-2′-deoxyuridine (BrdU) for 30 min before harvesting. Following labeling, cells were collected by trypsinization, fixed in ice-cold 70% ethanol, and incubated on ice for 30 min. DNA was denatured by incubation in 2 M HCl for 30 min at room temperature and neutralized with 0.1 M sodium tetraborate (Na_2_B_4_O_7_). After washing with PBS containing 0.5% Tween-20 and 1% BSA, BrdU incorporation was detected using a primary anti-BrdU antibody (EXBIO) followed by an FITC-conjugated secondary antimouse antibody (Sigma), with all incubations performed at room temperature in the dark. Finally, cells were counterstained with propidium iodide (0.1 mg/mL) and treated with RNase A (0.5 mg/mL) for 1 h. Flow cytometric analysis was conducted on a FACSCalibur cytometer (Becton Dickinson) equipped with a 488 nm laser.

#### Analysis of RNA Synthesis by BrU Incorporation

Cells were cultured and treated under the same conditions as for cell cycle analysis. To assess RNA synthesis, cells were pulse-labeled with 1 mM 5-bromouridine (BrU) for 30 min before harvesting. After labeling, cells were fixed in 1% paraformaldehyde (PBS-buffered) containing 0.05% NP-40 for 15 min at room temperature and stored at 4 °C overnight to enhance fixation. Residual aldehyde groups were quenched with 1% glycine in PBS, followed by PBS washing. BrU incorporation was detected using a primary anti-BrdU antibody (EXBIO; cross-reactive with BrU) and an FITC-conjugated secondary antimouse antibody (Sigma), with both incubations performed for 30 min at room temperature in the dark. To stabilize fluorescence, cells underwent a secondary fixation in 1% paraformaldehyde with 0.05% NP-40. Finally, cells were stained with propidium iodide (0.1 mg/mL) and treated with RNase A (0.5 mg/mL) for 1 h at room temperature in the dark. Flow cytometry was performed using a FACSCalibur cytometer (Becton Dickinson) equipped with a 488 nm laser.

## Supplementary Material



## Notes

Full characterization data, specifically comprising ^1^H and ^13^C NMR, as well as HRMS data for all synthesized compounds (Figures S1–S123); all measured cytotoxic activities, including error bars and *in silico* data from predicted ADMET studies (Tables S1–S3) (PDF)
