# Supplementary material for: New Synthetic Approach to C‑30 Ethers, Esters, and Amines of Betulin Using the Mitsunobu Reaction and Biological Evaluation of the Products
Source: ACS Omega. 2026 Apr 13;11(16):24564–79. doi: 10.1021/acsomega.6c00716 (PMC13129828; doi:10.1021/acsomega.6c00716)
Supplement: Supplementary file 1 [file ao6c00716_si_001.pdf]

New Synthetic Approach to C-30 Ethers, Esters, and Amines of Betulin using Mitsunobu Reaction and Biological Evaluation of the Products

Jan Bachořík,[a] Ivo Frydrych,[b] Soňa Gurská,[b] Štěpán Dostál,[d] Jan Pokorný,[a] Petr Džubák,[b] Marián Hajdúch,[b] Milan Urban[c]*

[a]Department of Organic Chemistry, Faculty of Science, Palacký University Olomouc, 17. Listopadu 1192/12, 779 00 Olomouc, Czech Republic.

[b]Laboratory of Experimental Medicine, Institute of Molecular and Translational Medicine, Faculty of Medicine and Dentistry, Palacký University and University Hospital Olomouc, Hněvotínská 1333/5, 779 00 Olomouc, Czech Republic.

[c]Laboratory of Medicinal and Organic Chemistry, Institute of Molecular and Translational Medicine, Faculty of Medicine and Dentistry, Palacký University Olomouc, Hněvotínská 1333/5, 779 00 Olomouc, Czech Republic.

[d]Department of Analytical Chemistry, Faculty of Science, Palacký University Olomouc, 17. Listopadu 1192/12, 779 00 Olomouc, Czech Republic.

Corresponding Author

Milan Urban - orcid.org/0000-0002-8249-028X; Phone: +420 585 632 197; Email: milan.urban@upol.cz

Contents

Figure S1. ^{1}H NMR spectrum of the compound **1** ($CDCl_3$, 500 MHz)....6
Figure S2. ^{13}C NMR spectrum of the compound **1** ($CDCl_3$, 126 MHz). 6
Figure S3. HRMS spectrum of the compound **1**....7
Figure S4. ^{1}H NMR spectrum of the compound **2** ($CDCl_3$, 500 MHz)....8
Figure S5. ^{13}C NMR spectrum of the compound **2** ($CDCl_3$, 126 MHz). 8
Figure S6. HRMS spectrum of the compound **2**....9
Figure S7. ^{1}H NMR spectrum of the compound **3** ($CDCl_3$, 500 MHz)....9
Figure S8. ^{13}C NMR spectrum of the compound **3** ($CDCl_3$, 126 MHz). 10
Figure S9. HRMS spectrum of the compound **3**....10
Figure S10. ^{1}H NMR spectrum of the compound **4** ($CDCl_3$, 500 MHz)....11
Figure S11. ^{13}C NMR spectrum of the compound **4** ($CDCl_3$, 126 MHz). 11
Figure S12. HRMS spectrum of the compound **4**....12
Figure S13. ^{1}H NMR spectrum of the compound **5** ($CDCl_3$, 400 MHz)....12
Figure S14. ^{13}C NMR spectrum of the compound **5** ($CDCl_3$, 101 MHz). 13
Figure S15. HRMS spectrum of the compound **5**....13
Figure S16. ^{1}H NMR spectrum of the compound **6** ($CDCl_3$, 400 MHz)....14
Figure S17. ^{13}C NMR spectrum of the compound **6** ($CDCl_3$, 101 MHz). 14
Figure S18. HRMS spectrum of the compound **6**....15
Figure S19. ^{1}H NMR spectrum of the compound **7** ($CDCl_3$, 500 MHz)....15
Figure S20. ^{13}C NMR spectrum of the compound **7** ($CDCl_3$, 126 MHz). 16
Figure S21. HRMS spectrum of the compound **7**....16
Figure S22. ^{1}H NMR spectrum of the compound **8** ($CDCl_3$, 500 MHz)....17
Figure S23. ^{13}C NMR spectrum of the compound **8** ($CDCl_3$, 126 MHz). 17
Figure S24. HRMS spectrum of the compound **8**....18
Figure S25. ^{1}H NMR spectrum of the compound **9** ($CDCl_3$, 500 MHz)....18
Figure S26. ^{13}C NMR spectrum of the compound **9** ($CDCl_3$, 126 MHz). 19
Figure S27. HRMS spectrum of the compound **9**....19
Figure S28. ^{1}H NMR spectrum of the compound **10** ($CDCl_3$, 500 MHz)....20
Figure S29. ^{13}C NMR spectrum of the compound **10** ($CDCl_3$, 126 MHz). 20
Figure S30. HRMS spectrum of the compound **10**....21
Figure S31. ^{1}H NMR spectrum of the compound **11** ($CDCl_3$, 500 MHz)....21
Figure S32. ^{13}C NMR spectrum of the compound **11** ($CDCl_3$, 126 MHz). 22
Figure S33. HRMS spectrum of the compound **11**....22
Figure S34. ^{1}H NMR spectrum of the compound **12** ($CDCl_3$, 500 MHz)....23
Figure S35. ^{13}C NMR spectrum of the compound **12** ($CDCl_3$, 126 MHz). 23
Figure S36. HRMS spectrum of the compound **12**....24
Figure S37. ^{1}H NMR spectrum of the compound **13** ($CDCl_3$, 500 MHz)....24
Figure S38. ^{13}C NMR spectrum of the compound **13** ($CDCl_3$, 126 MHz). 25

Figure S39. HRMS spectrum of the compound **13**......25
Figure S40. ^{1}H NMR spectrum of the compound **14** ($CDCl_3$, 500 MHz)......26
Figure S41. ^{13}C NMR spectrum of the compound **14** ($CDCl_3$, 126 MHz). 26
Figure S42. HRMS spectrum of the compound **14**......27
Figure S43. ^{1}H NMR spectrum of the compound **15** ($CDCl_3$, 500 MHz)......27
Figure S44. ^{13}C NMR spectrum of the compound **15** ($CDCl_3$, 126 MHz). 28
Figure S45. HRMS spectrum of the compound **15**......28
Figure S46. ^{1}H NMR spectrum of the compound **16** ($CDCl_3$, 500 MHz)......29
Figure S47. ^{13}C NMR spectrum of the compound **16** ($CDCl_3$, 126 MHz). 29
Figure S48. HRMS spectrum of the compound **16**......30
Figure S49. ^{1}H NMR spectrum of the compound **17** ($CDCl_3$, 500 MHz)......30
Figure S50. ^{13}C NMR spectrum of the compound **17** ($CDCl_3$, 126 MHz). 31
Figure S51. HRMS spectrum of the compound **17**......31
Figure S52. ^{1}H NMR spectrum of the compound **18** ($CDCl_3$, 500 MHz)......32
Figure S53. ^{13}C NMR spectrum of the compound **18** ($CDCl_3$, 126 MHz). 32
Figure S54. HRMS spectrum of the compound **18**......33
Figure S55. ^{1}H NMR spectrum of the compound **19** ($CDCl_3$, 500 MHz, contains also residual TMS). 33
Figure S56. ^{13}C NMR spectrum of the compound **19** ($CDCl_3$, 126 MHz). 34
Figure S57. HRMS spectrum of the compound **19**......34
Figure S58. ^{1}H NMR spectrum of the compound **20** ($CDCl_3$, 500 MHz)......35
Figure S59. ^{13}C NMR spectrum of the compound **20** ($CDCl_3$, 126 MHz). 35
Figure S60. HRMS spectrum of the compound **20**......36
Figure S61. ^{1}H NMR spectrum of the compound **21** ($CDCl_3$, 500 MHz)......36
Figure S62. ^{13}C NMR spectrum of the compound **21** ($CDCl_3$, 126 MHz). 37
Figure S63. HRMS spectrum of the compound **21**......37
Figure S64. ^{1}H NMR spectrum of the compound **22** ($CDCl_3$, 500 MHz)......38
Figure S65. ^{13}C NMR spectrum of the compound **22** ($CDCl_3$, 126 MHz). 38
Figure S66. HRMS spectrum of the compound **22**......39
Figure S67. ^{1}H NMR spectrum of the compound **23** ($CDCl_3$, 500 MHz)......39
Figure S68. ^{13}C NMR spectrum of the compound **23** ($CDCl_3$, 126 MHz). 40
Figure S69. HRMS spectrum of the compound **23**......40
Figure S70. ^{1}H NMR spectrum of the compound **24** ($CDCl_3$, 500 MHz)......41
Figure S71. ^{13}C NMR spectrum of the compound **24** ($CDCl_3$, 126 MHz). 41
Figure S72. HRMS spectrum of the compound **24**......42
Figure S73. ^{1}H NMR spectrum of the compound **25** ($CDCl_3$, 500 MHz)......42
Figure S74. ^{13}C NMR spectrum of the compound **25** ($CDCl_3$, 126 MHz). 43
Figure S75. HRMS spectrum of the compound **25**......43
Figure S76. ^{1}H NMR spectrum of the compound **26** ($CDCl_3$, 500 MHz)......44
Figure S77. ^{13}C NMR spectrum of the compound **26** ($CDCl_3$, 126 MHz). 44
Figure S78. HRMS spectrum of the compound **26**......45

Figure S79. ^{1}H NMR spectrum of the compound **27** ($CDCl_3$, 500 MHz)....45
Figure S80. ^{13}C NMR spectrum of the compound **27** ($CDCl_3$, 126 MHz). 46
Figure S81. HRMS spectrum of the compound **27**....46
Figure S82. ^{1}H NMR spectrum of the compound **28** ($CDCl_3$, 500 MHz)....47
Figure S83. ^{13}C NMR spectrum of the compound **28** ($CDCl_3$, 126 MHz). 47
Figure S84. HRMS spectrum of the compound **28**....48
Figure S85. ^{1}H NMR spectrum of the compound **29** ($CDCl_3$, 500 MHz)....48
Figure S86. ^{13}C NMR spectrum of the compound **29** ($CDCl_3$, 126 MHz). 49
Figure S87. HRMS spectrum of the compound **29**....49
Figure S88. ^{1}H NMR spectrum of the compound **30** ($CDCl_3$, 500 MHz)....50
Figure S89. ^{13}C NMR spectrum of the compound **30** ($CDCl_3$, 126 MHz). 50
Figure S90. HRMS spectrum of the compound **30**....51
Figure S91. ^{1}H NMR spectrum of the compound **31** ($CDCl_3$, 400 MHz)....51
Figure S92. ^{13}C NMR spectrum of the compound **31** ($CDCl_3$, 101 MHz). 52
Figure S93. HRMS spectrum of the compound **31**....52
Figure S94. ^{1}H NMR spectrum of the compound **32** ($CDCl_3$, 500 MHz)....53
Figure S95. ^{13}C NMR spectrum of the compound **32** ($CDCl_3$, 126 MHz). 53
Figure S96. HRMS spectrum of the compound **32**....54
Figure S97. ^{1}H NMR spectrum of the compound **33** ($CDCl_3$, 500 MHz)....54
Figure S98. ^{13}C NMR spectrum of the compound **33** ($CDCl_3$, 126 MHz). 55
Figure S99. HRMS spectrum of the compound **33**....55
Figure S100. ^{1}H NMR spectrum of the compound **34** ($CDCl_3$, 500 MHz)....56
Figure S101. ^{13}C NMR spectrum of the compound **34** ($CDCl_3$, 126 MHz). 56
Figure S102. HRMS spectrum of the compound **34**....57
Figure S103. ^{1}H NMR spectrum of the compound **35** ($CDCl_3$, 500 MHz)....57
Figure S104. ^{13}C NMR spectrum of the compound **35** ($CDCl_3$, 126 MHz). 58
Figure S105. HRMS spectrum of the compound **35**....58
Figure S106. ^{1}H NMR spectrum of the compound **36** ($CDCl_3$, 500 MHz)....59
Figure S107. ^{13}C NMR spectrum of the compound **36** ($CDCl_3$, 126 MHz). 59
Figure S108. HRMS spectrum of the compound **36**....60
Figure S109. ^{1}H NMR spectrum of the compound **37** ($CDCl_3$, 500 MHz)....60
Figure S110. ^{13}C NMR spectrum of the compound **37** ($CDCl_3$, 126 MHz). 61
Figure S111. HRMS spectrum of the compound **37**....61
Figure S112. ^{1}H NMR spectrum of the compound **38** ($CDCl_3$, 500 MHz)....62
Figure S113. ^{13}C NMR spectrum of the compound **38** ($CDCl_3$, 126 MHz). 62
Figure S114. HRMS spectrum of the compound **38**....63
Figure S115. ^{1}H NMR spectrum of the compound **39** ($CDCl_3$, 500 MHz)....63
Figure S116. ^{13}C NMR spectrum of the compound **39** ($CDCl_3$, 126 MHz). 64
Figure S117. HRMS spectrum of the compound **39**....64
Figure S118. ^{1}H NMR spectrum of the compound **40** ($CDCl_3$, 500 MHz)....65
Figure S119. ^{13}C NMR spectrum of the compound **40** ($CDCl_3$, 126 MHz). 65

Figure S120. HRMS spectrum of the compound **40**....66
Figure S121. ^{1}H NMR spectrum of the compound **41** ($CDCl_3$, 500 MHz)....66
Figure S122. ^{13}C NMR spectrum of the compound **41** ($CDCl_3$, 126 MHz). 67
Figure S123. HRMS spectrum of the compound **41**....67
Table S1. Full table of all measured cytotoxic activities in all used cancer cells. 68
Table S2. Predicted ADME and Physicochemical Properties of selected compounds using the SwissADME webserver[45]. 69
Table S3. Predicted Toxicity Properties of selected compounds using the ProTox 3.0 webserver[49]. ...69

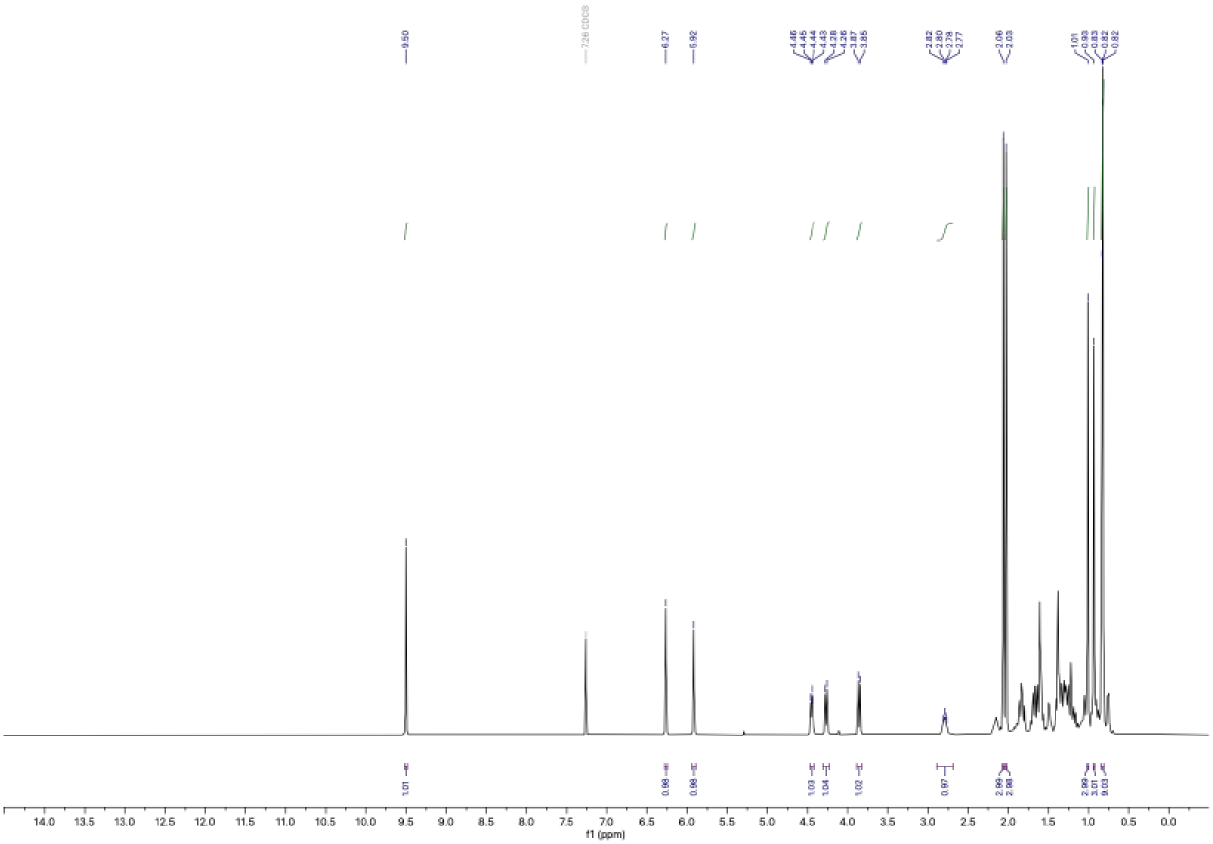

Figure S1. ^{1}H NMR spectrum of the compound **1** ($CDCl_3$, 500 MHz).

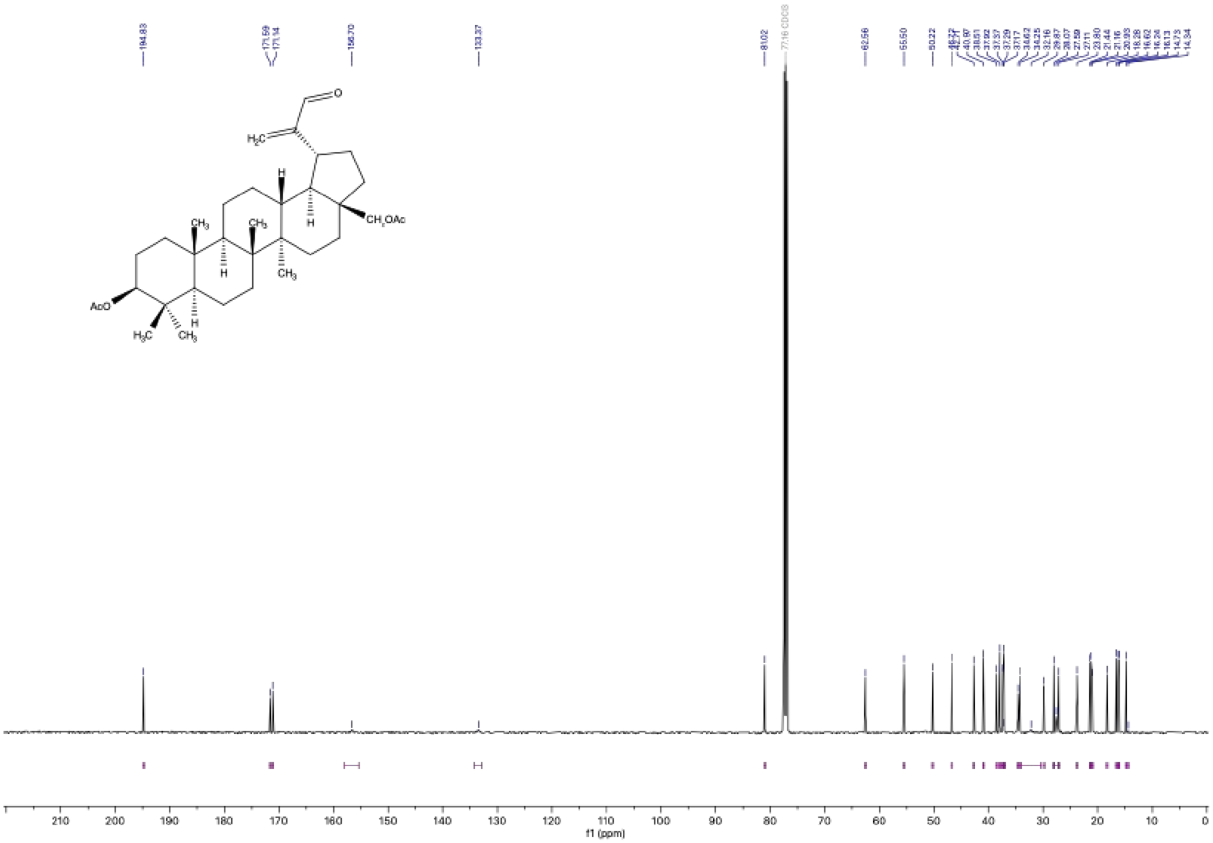

Figure S2. ^{13}C NMR spectrum of the compound **1** ($CDCl_3$, 126 MHz).

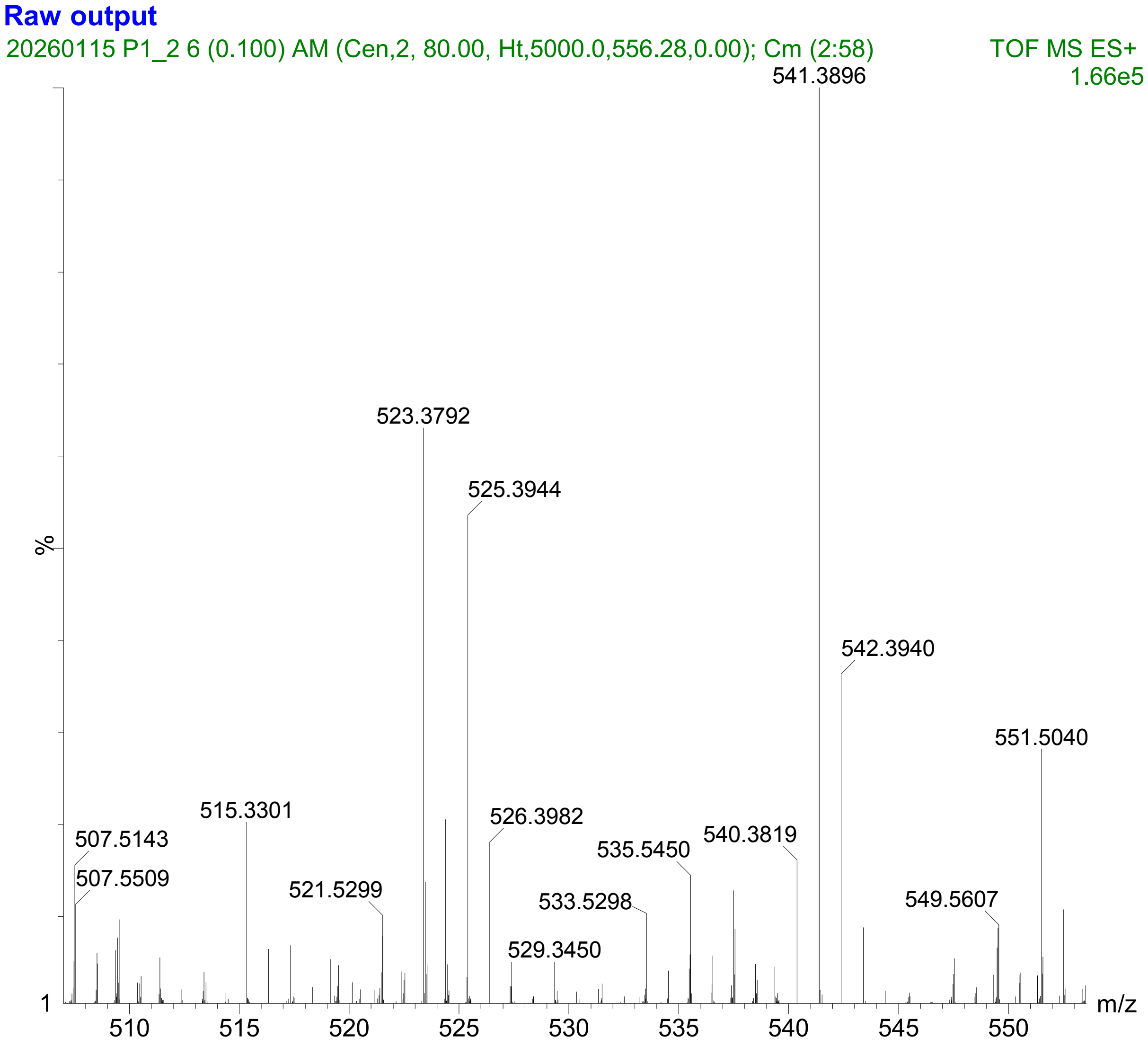

Figure S3. HRMS spectrum of the compound **1**.

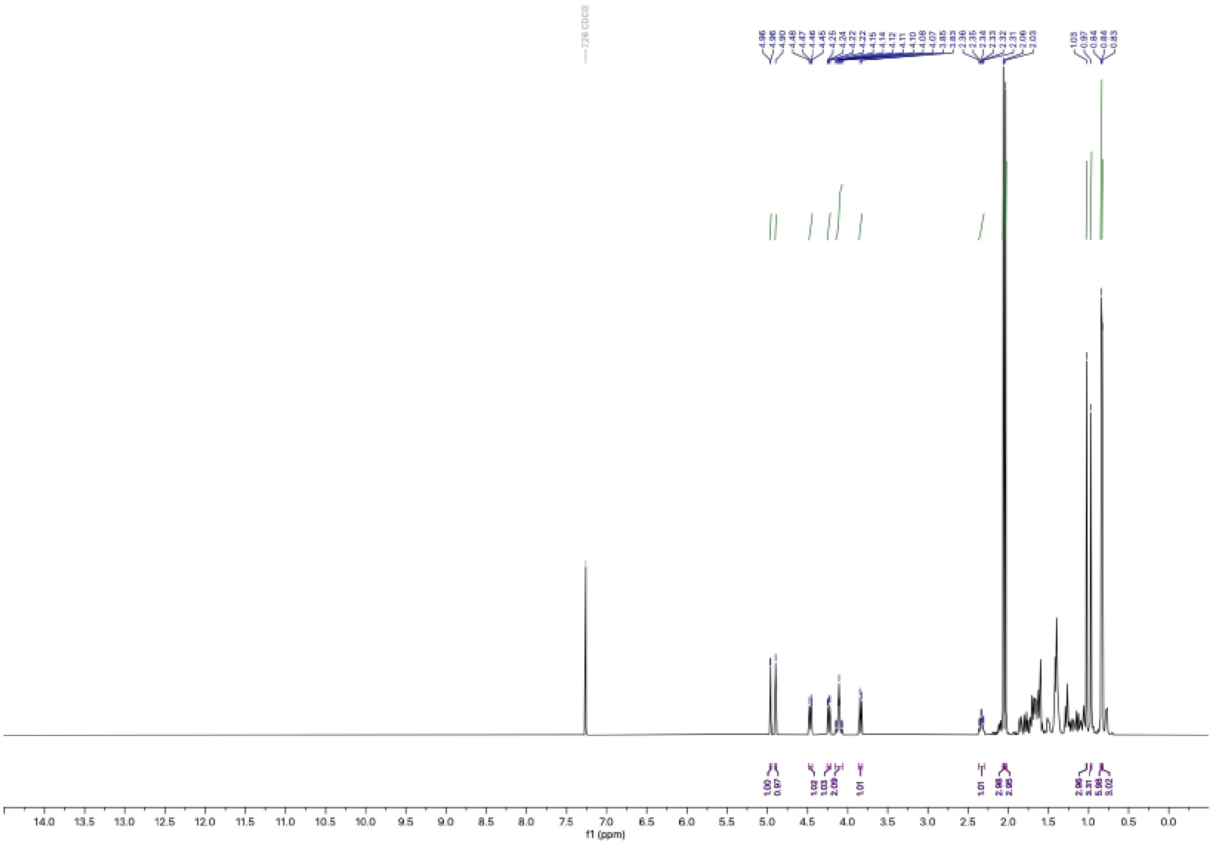

Figure S4. ^{1}H NMR spectrum of the compound **2** ($CDCl_3$, 500 MHz).

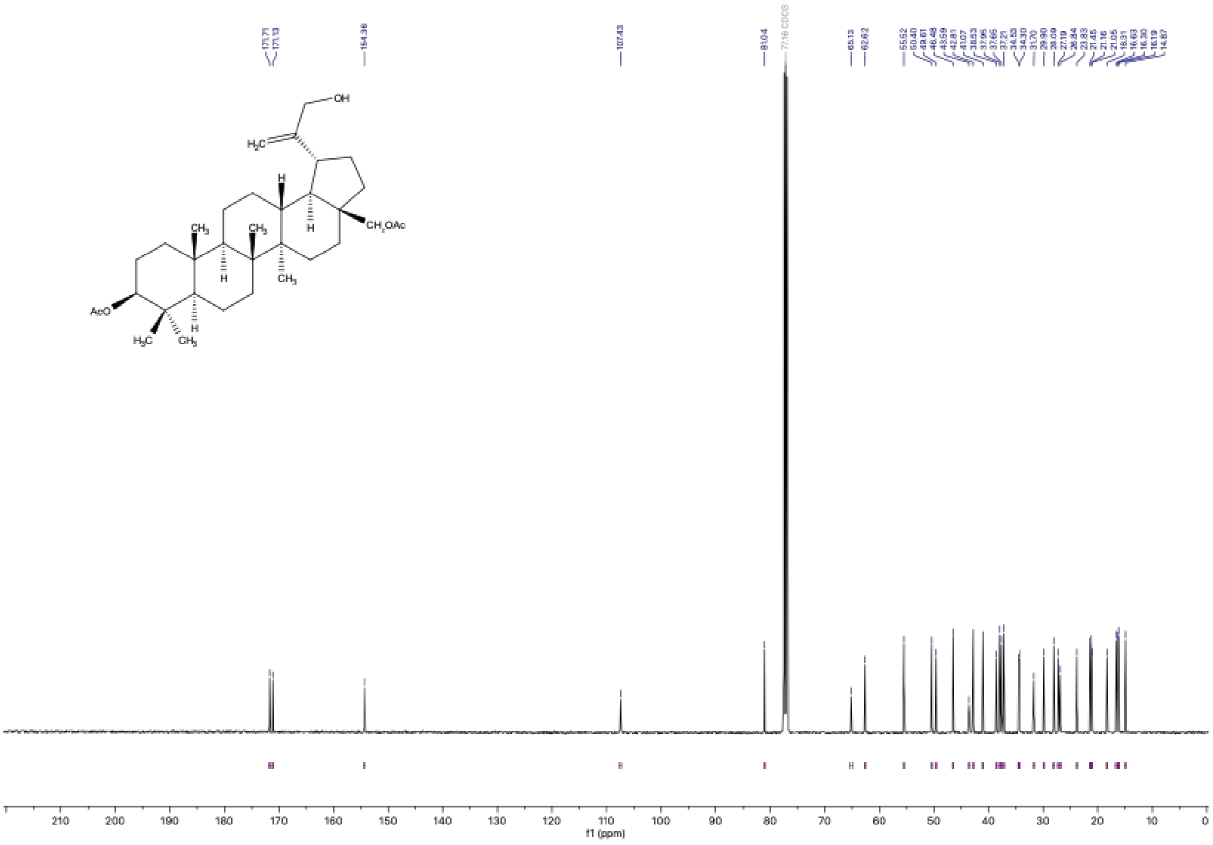

Figure S5. ^{13}C NMR spectrum of the compound **2** ($CDCl_3$, 126 MHz).

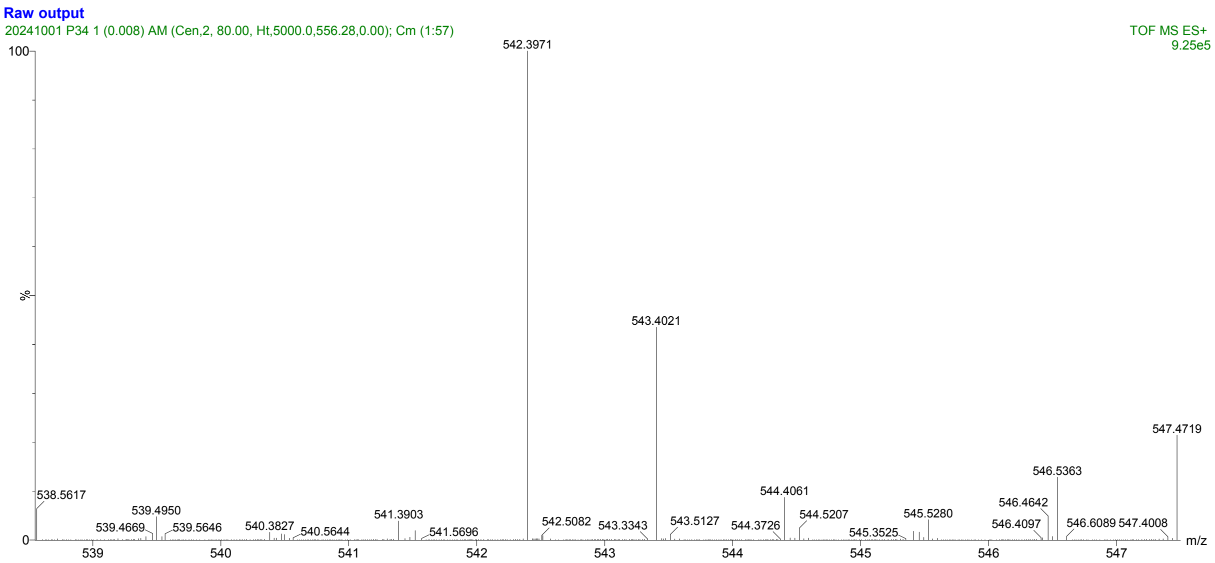

Figure S6. HRMS spectrum of the compound **2**.

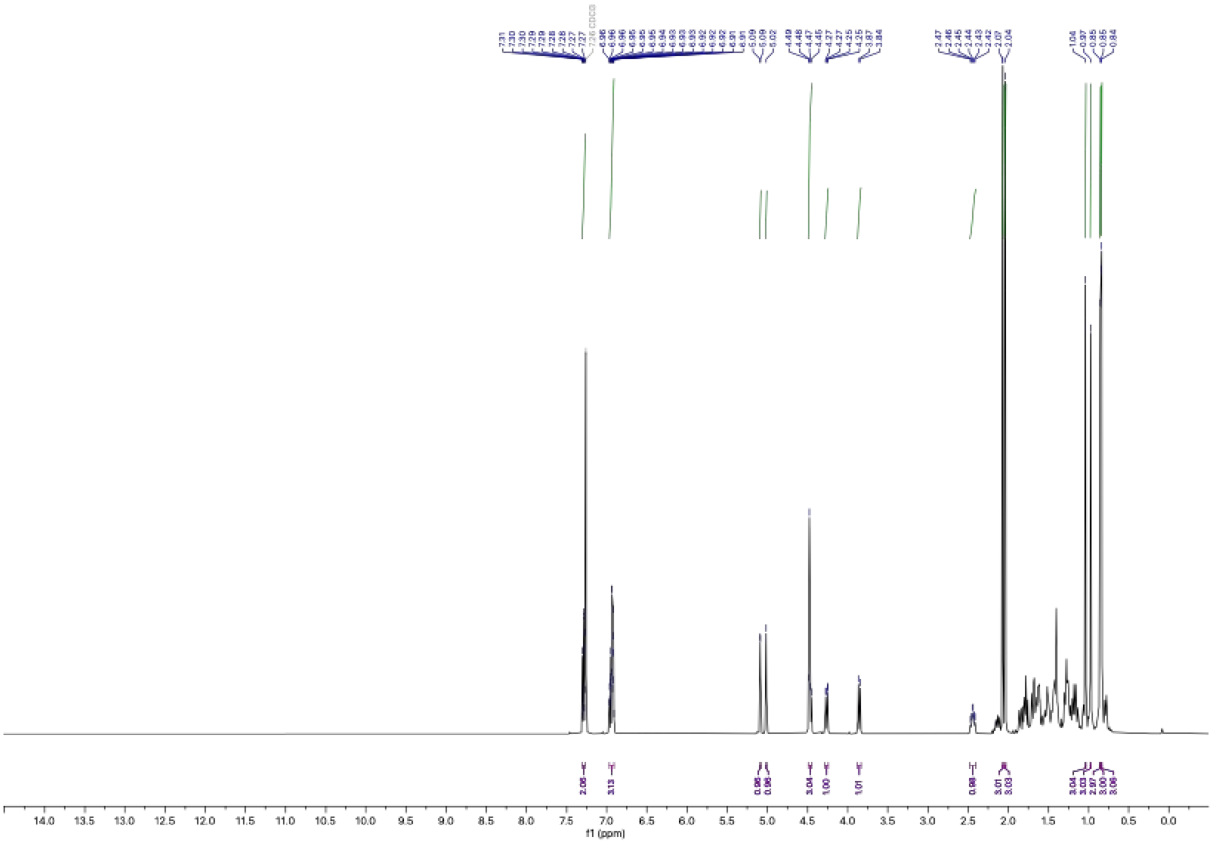

Figure S7. ^{1}H NMR spectrum of the compound **3** ($CDCl_3$, 500 MHz).

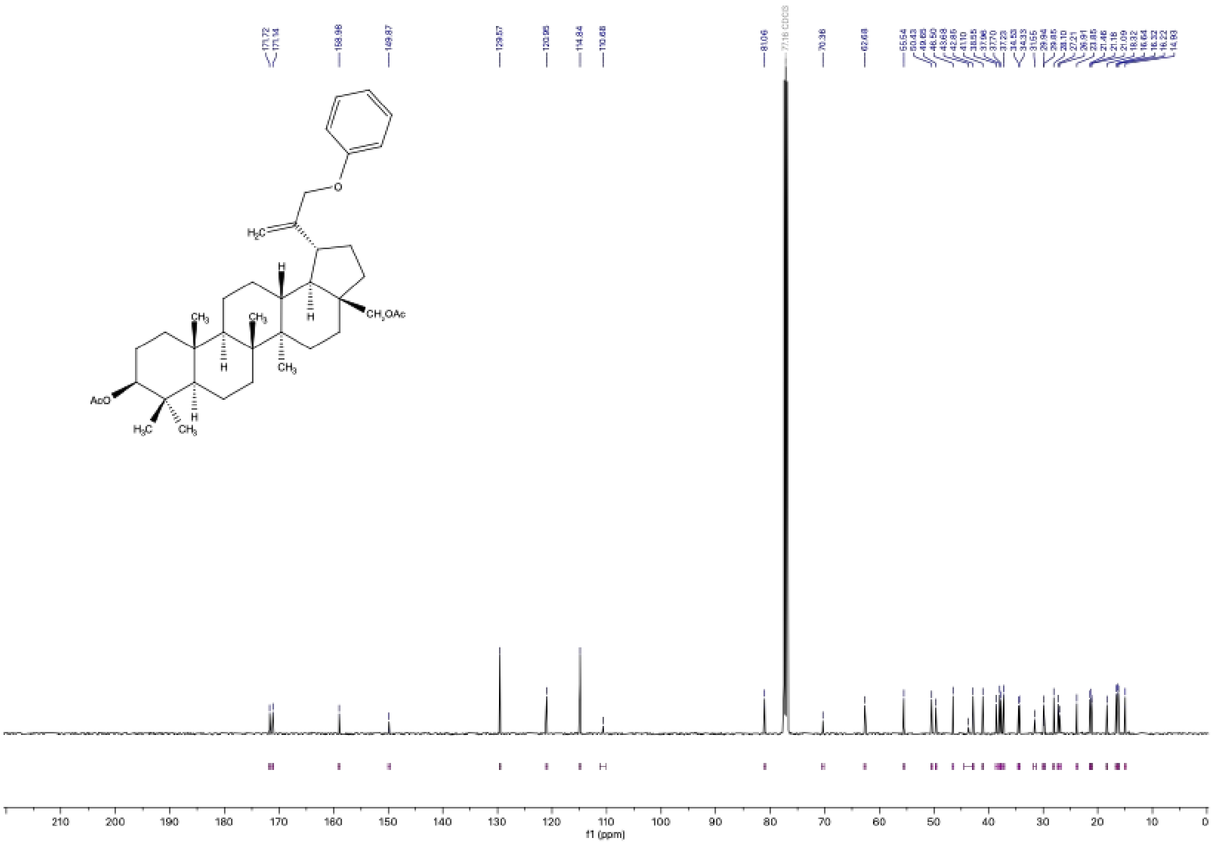

Figure S8. ^{13}C NMR spectrum of the compound **3** ($CDCl_3$, 126 MHz).

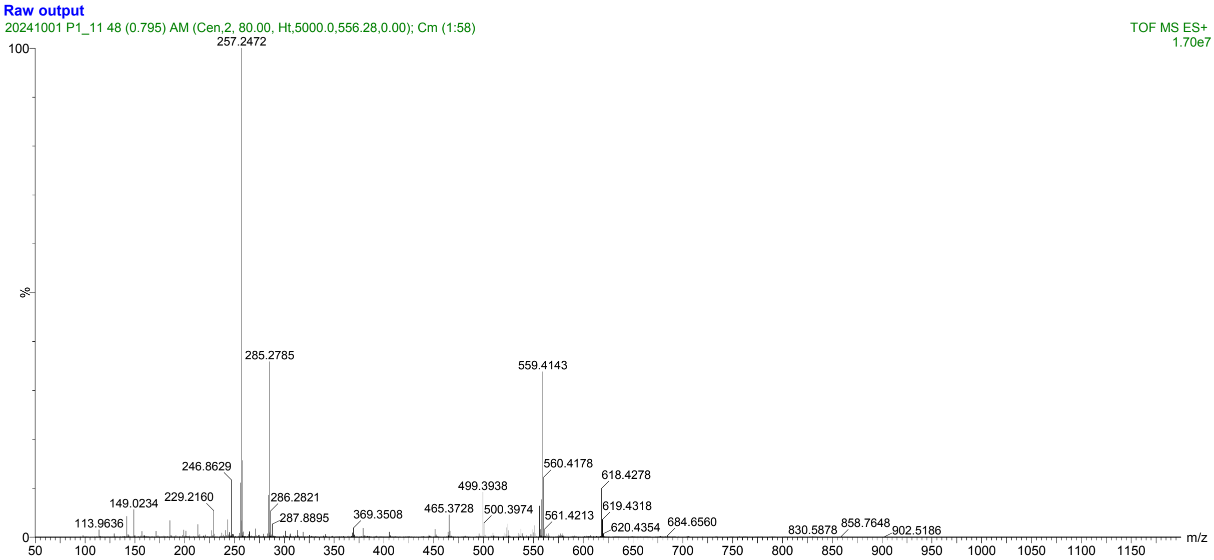

Figure S9. HRMS spectrum of the compound **3**.

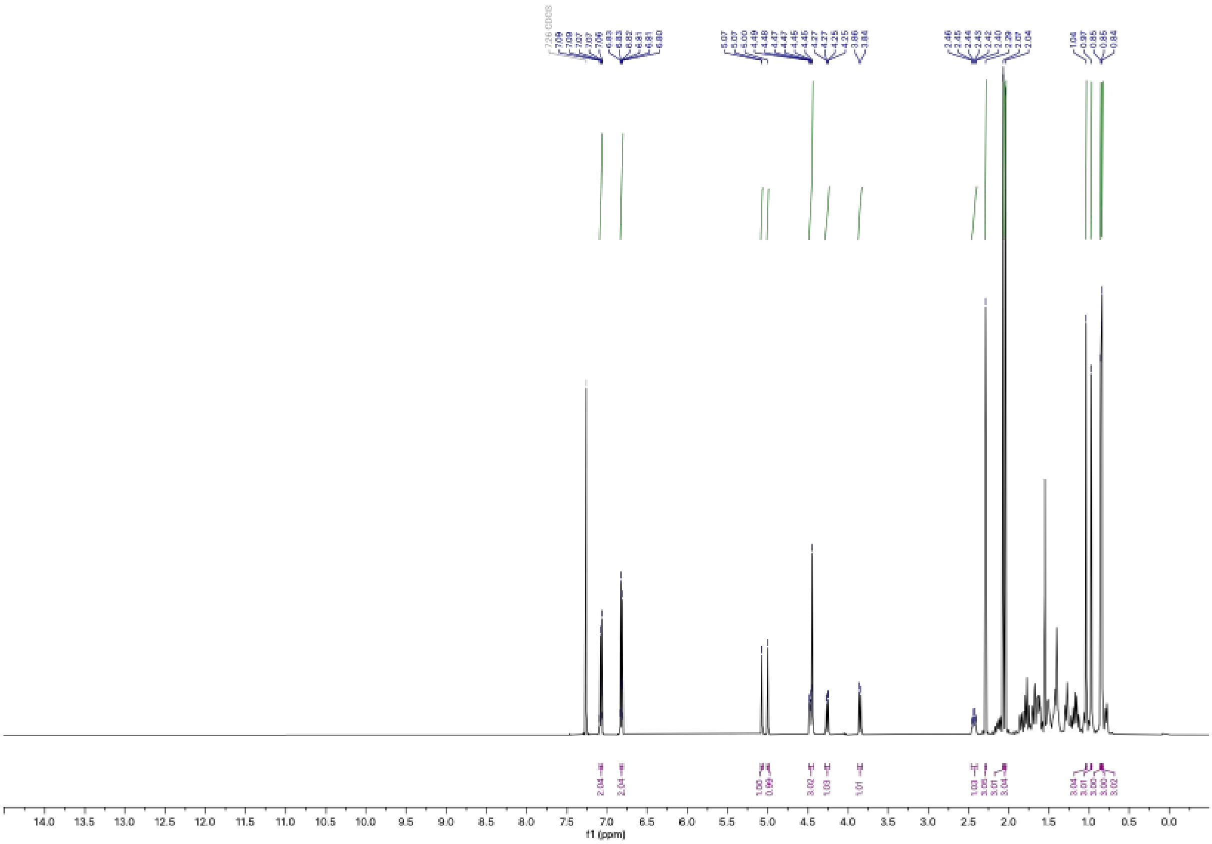

Figure S10. ^{1}H NMR spectrum of the compound **4** ($CDCl_3$, 500 MHz).

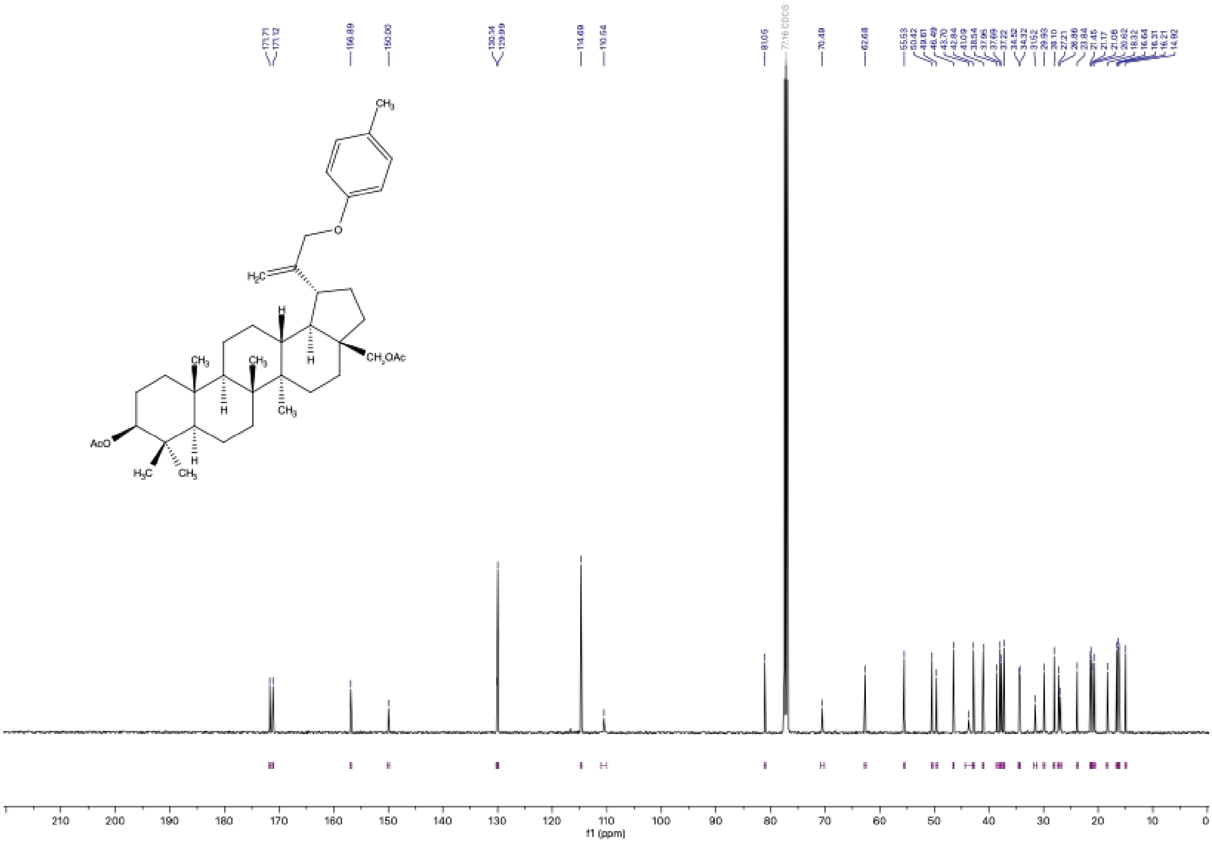

Figure S11. ^{13}C NMR spectrum of the compound **4** ($CDCl_3$, 126 MHz).

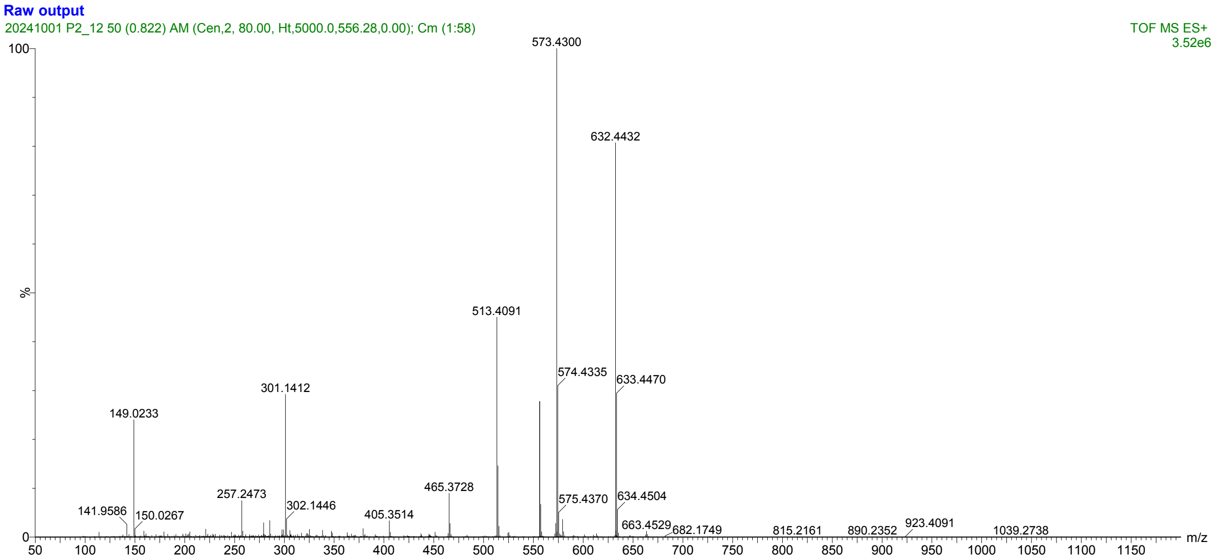

Figure S12. HRMS spectrum of the compound **4**.

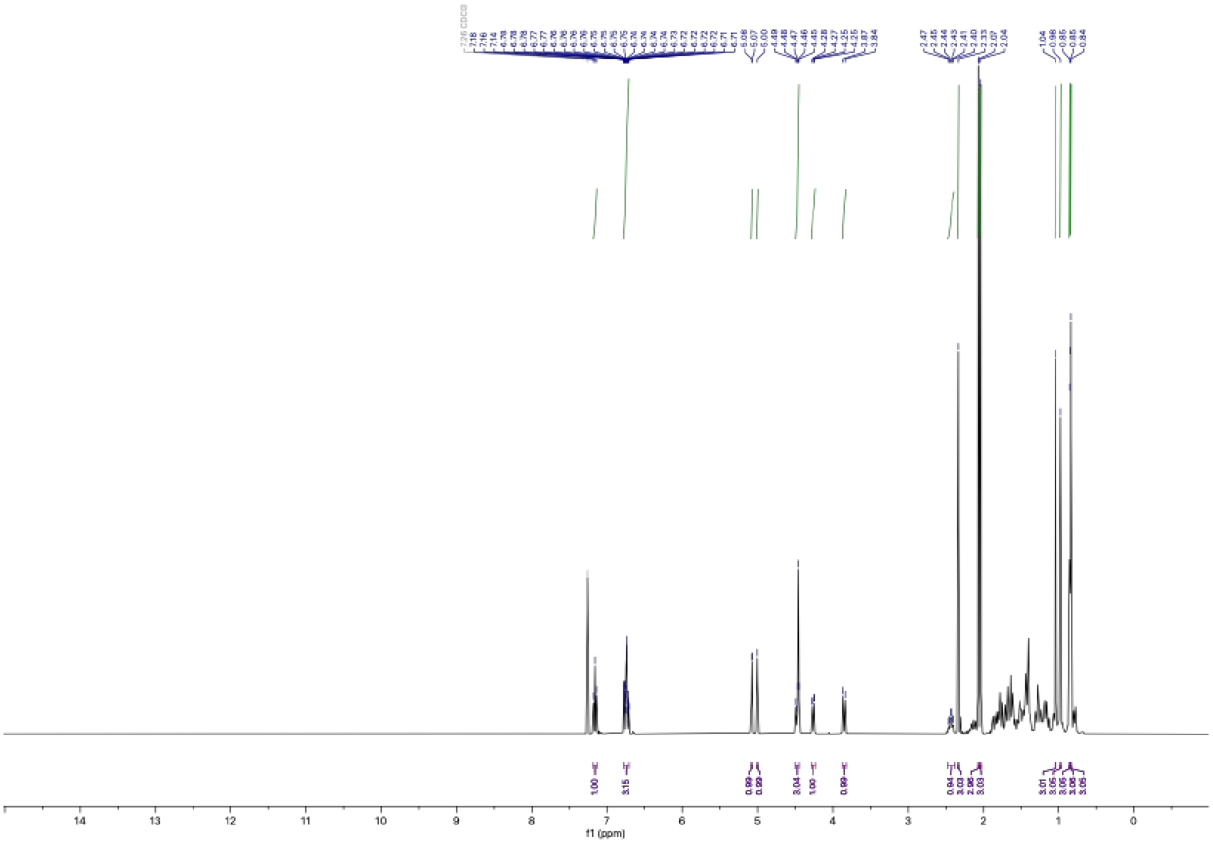

Figure S13. ^{1}H NMR spectrum of the compound **5** ($CDCl_3$, 400 MHz).

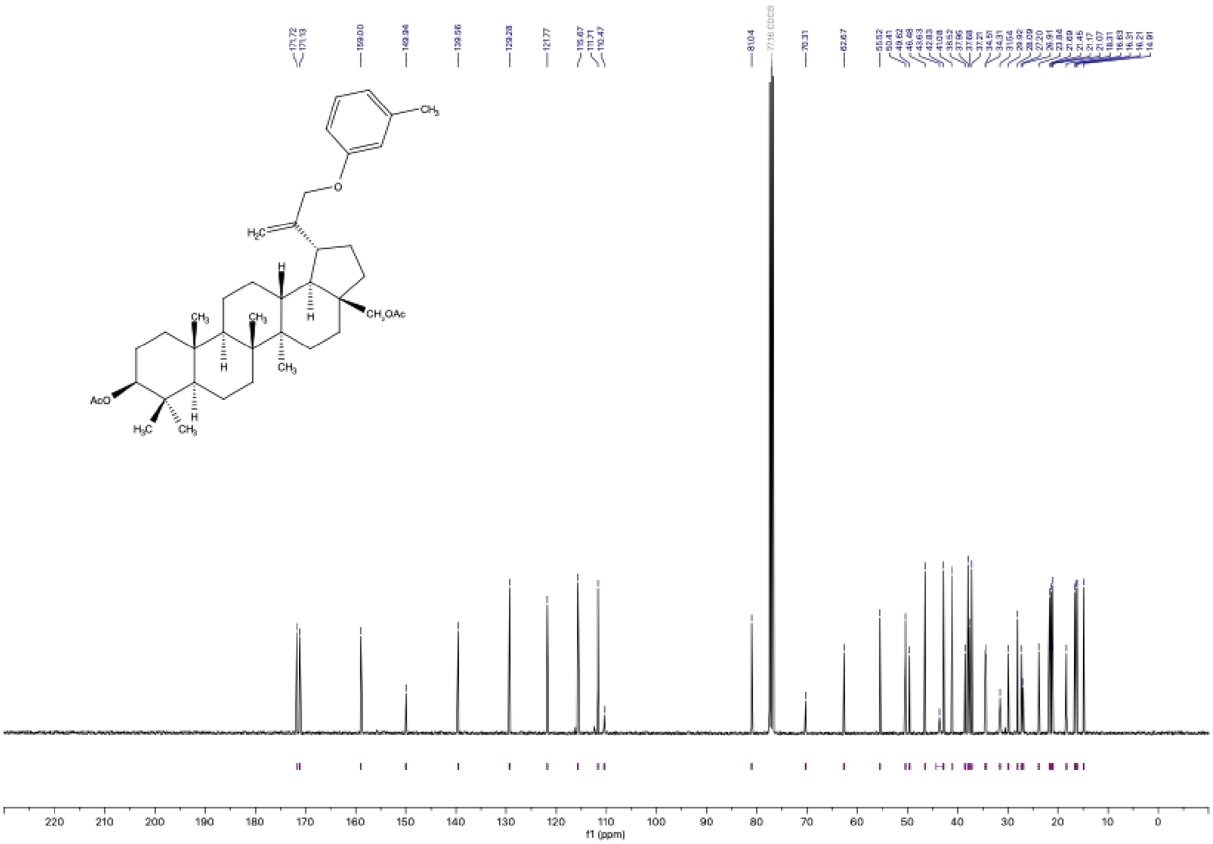

Figure S14. ^{13}C NMR spectrum of the compound **5** ($CDCl_3$, 101 MHz).

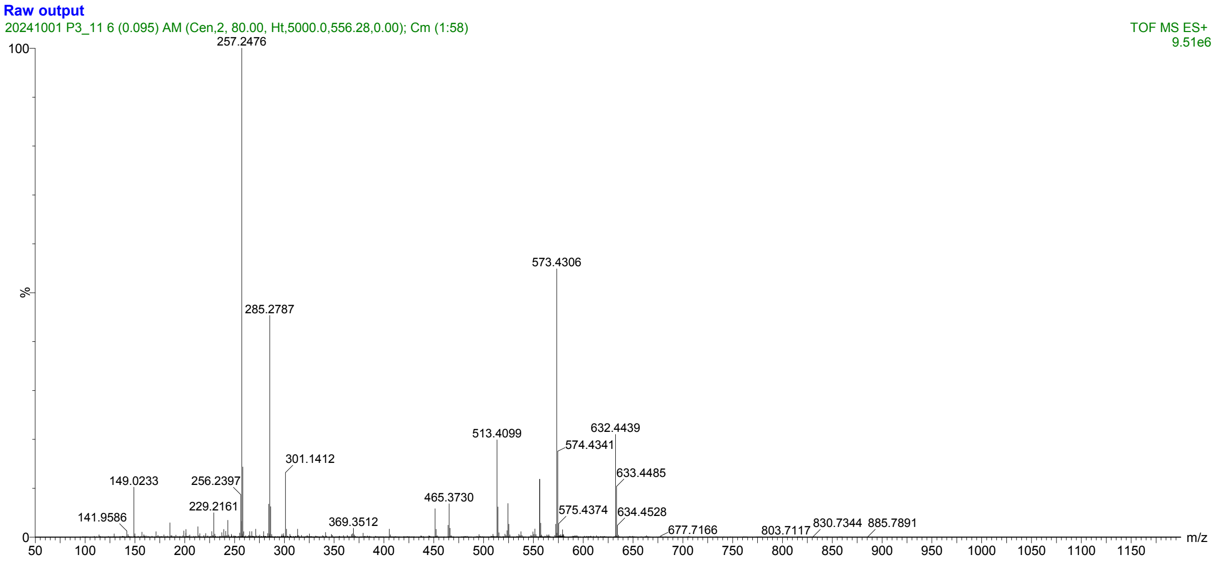

Figure S15. HRMS spectrum of the compound **5**.

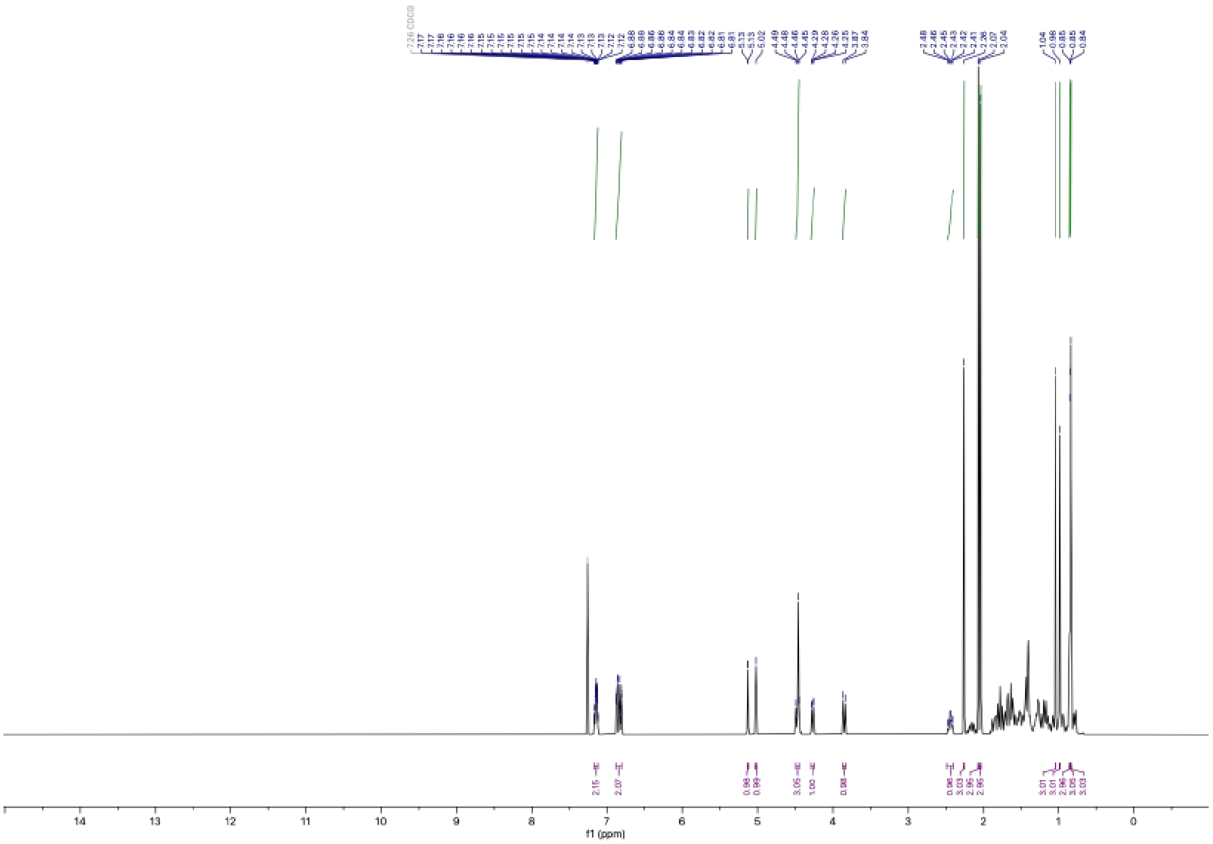

Figure S16. ^{1}H NMR spectrum of the compound **6** ($CDCl_3$, 400 MHz).

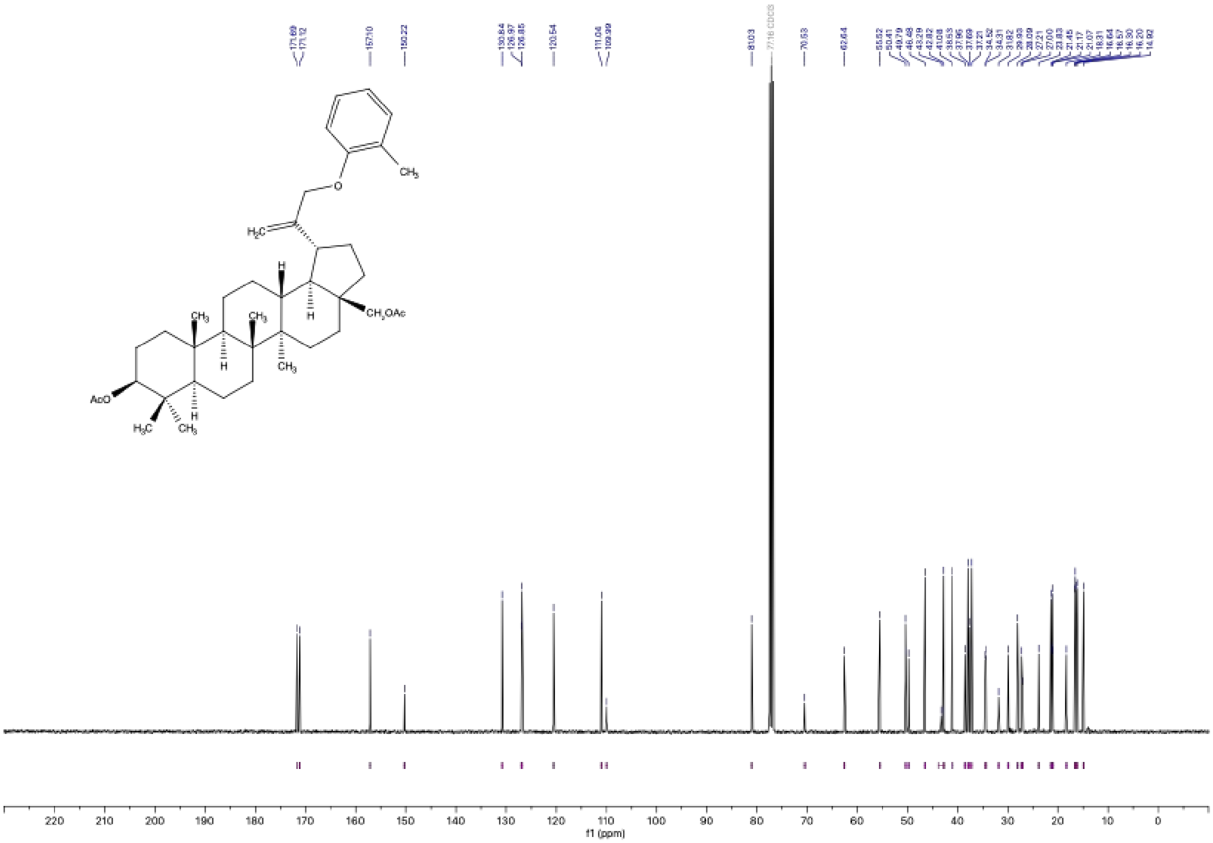

Figure S17. ^{13}C NMR spectrum of the compound **6** ($CDCl_3$, 101 MHz).

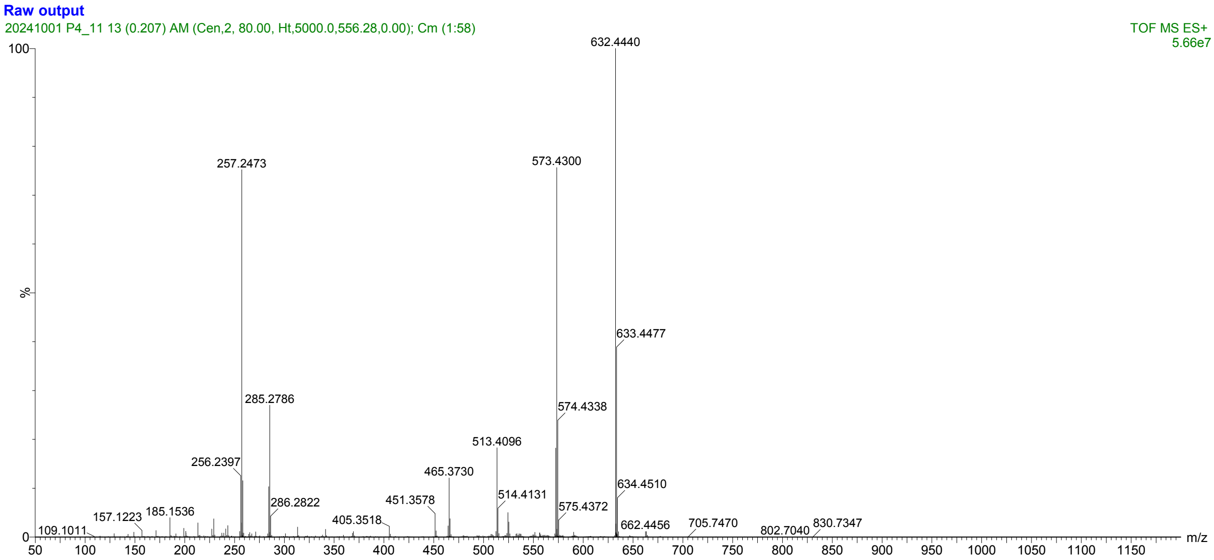

Figure S18. HRMS spectrum of the compound **6**.

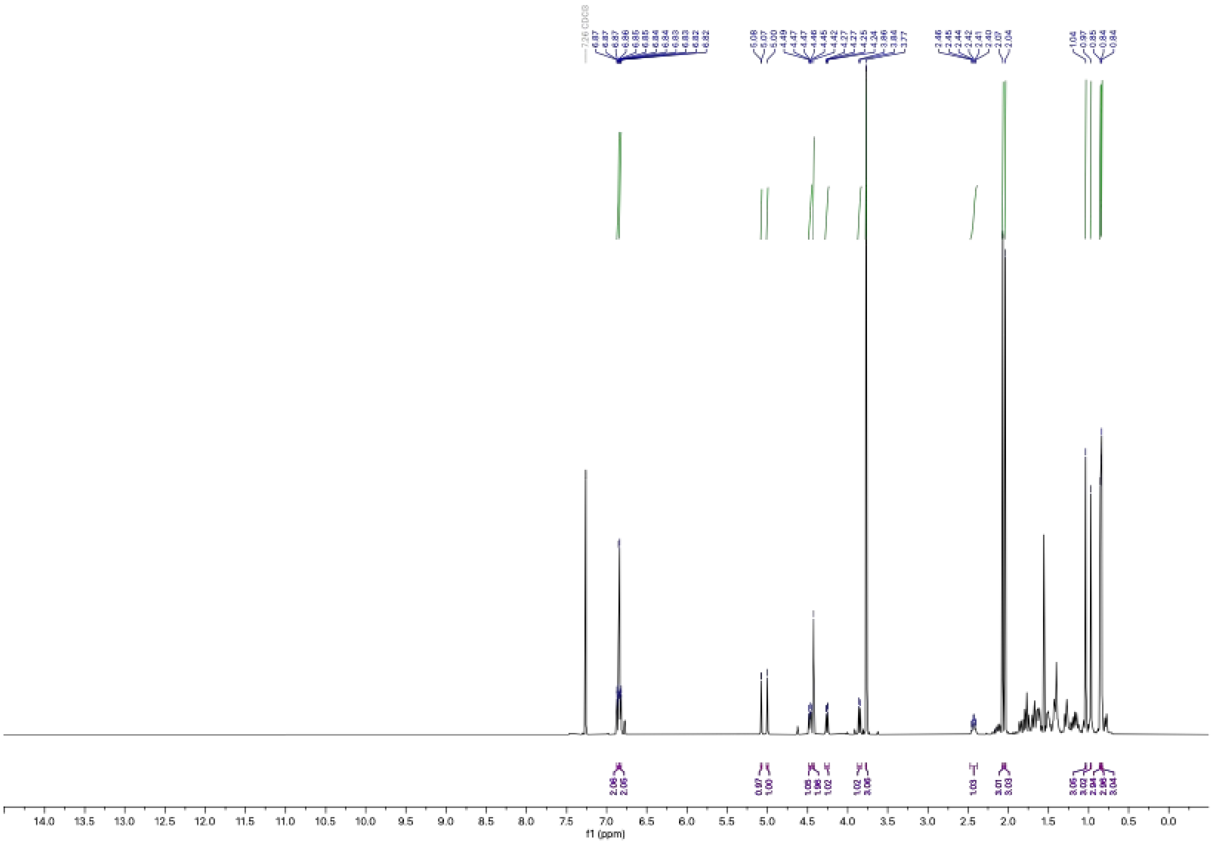

Figure S19. ^{1}H NMR spectrum of the compound **7** ($CDCl_3$, 500 MHz).

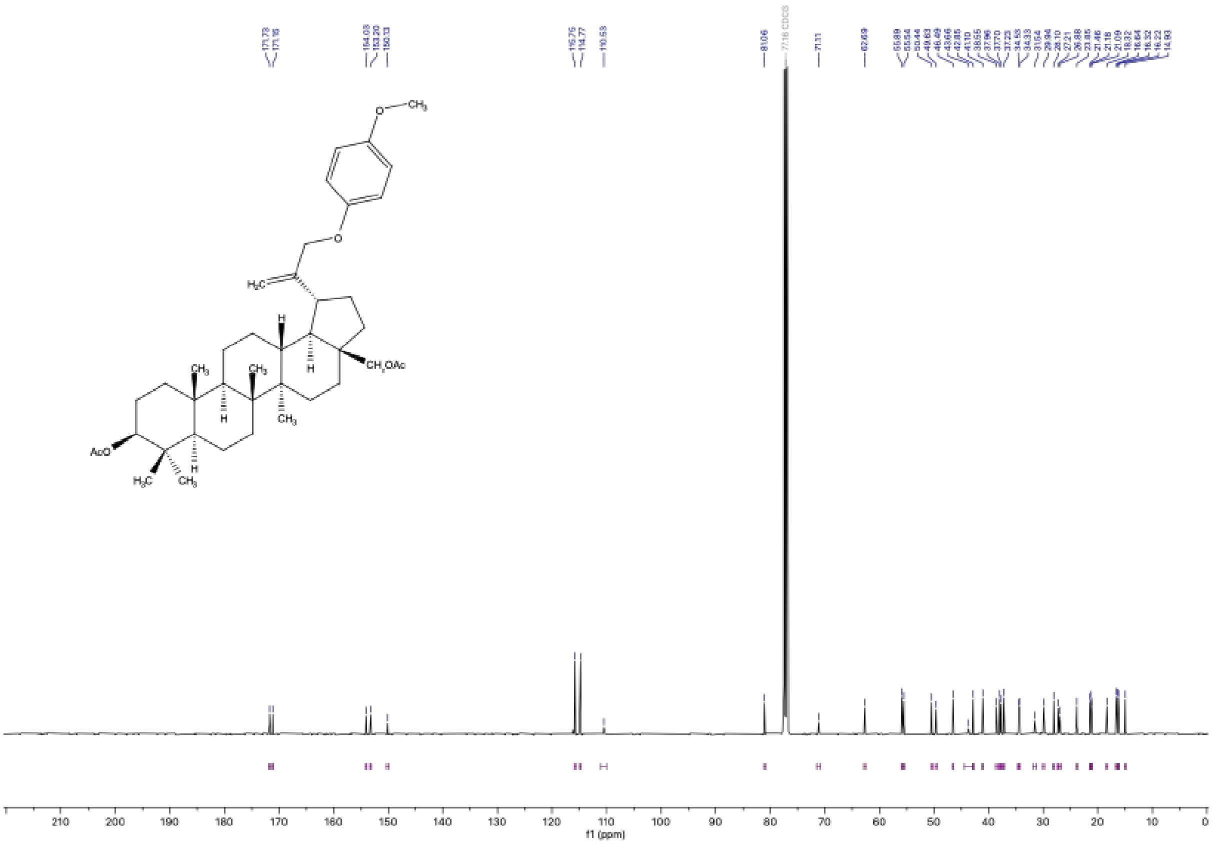

Figure S20. ^{13}C NMR spectrum of the compound **7** ($CDCl_3$, 126 MHz).

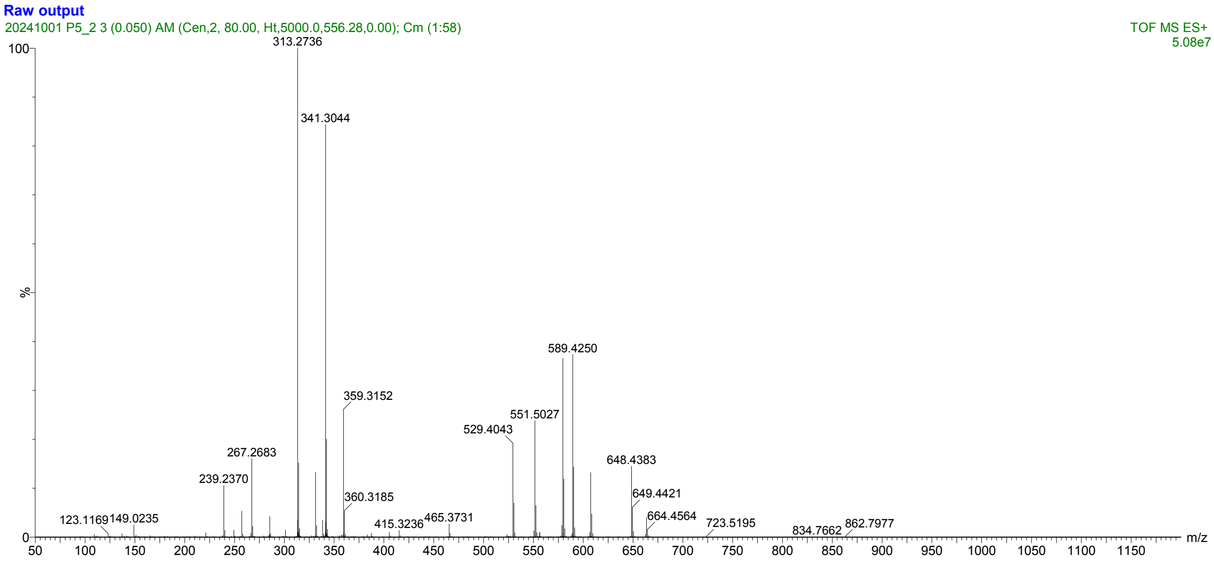

Figure S21. HRMS spectrum of the compound **7**.

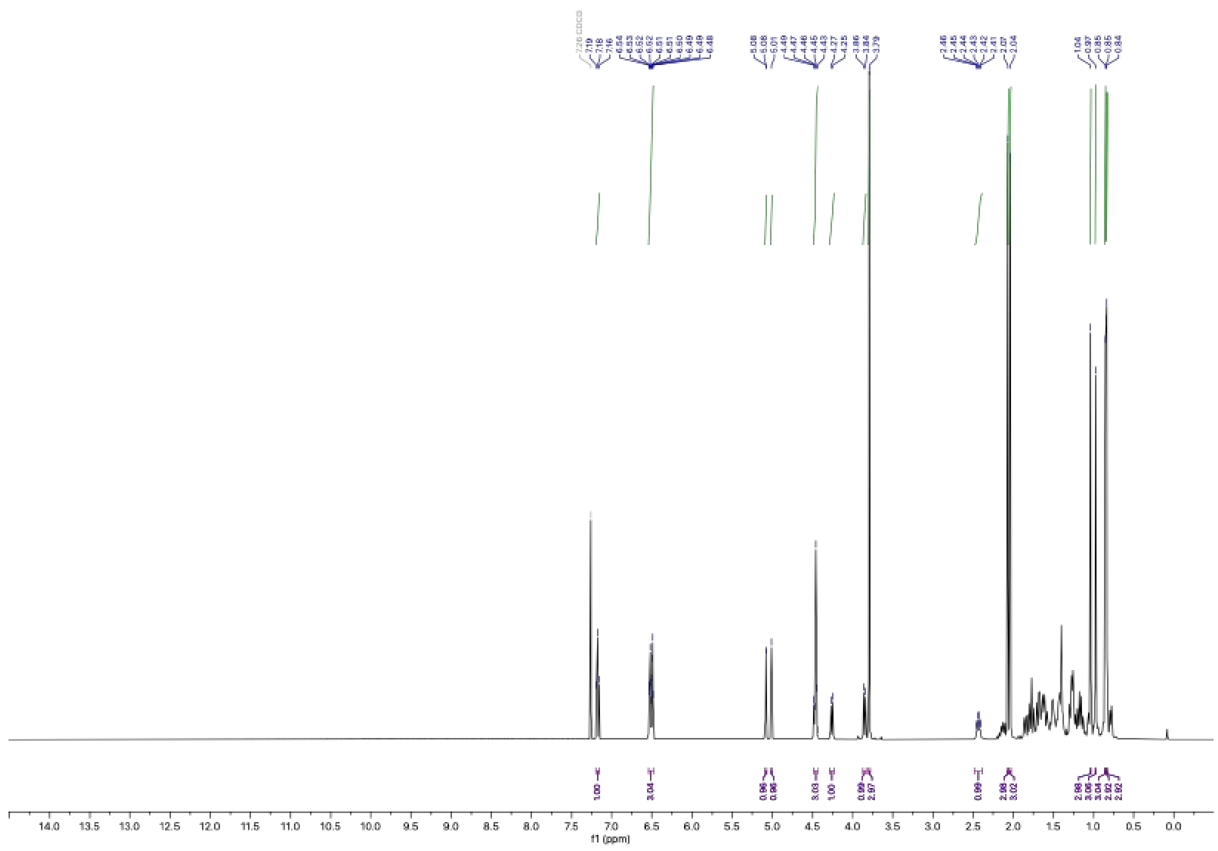

Figure S22. ^{1}H NMR spectrum of the compound **8** ($CDCl_3$, 500 MHz).

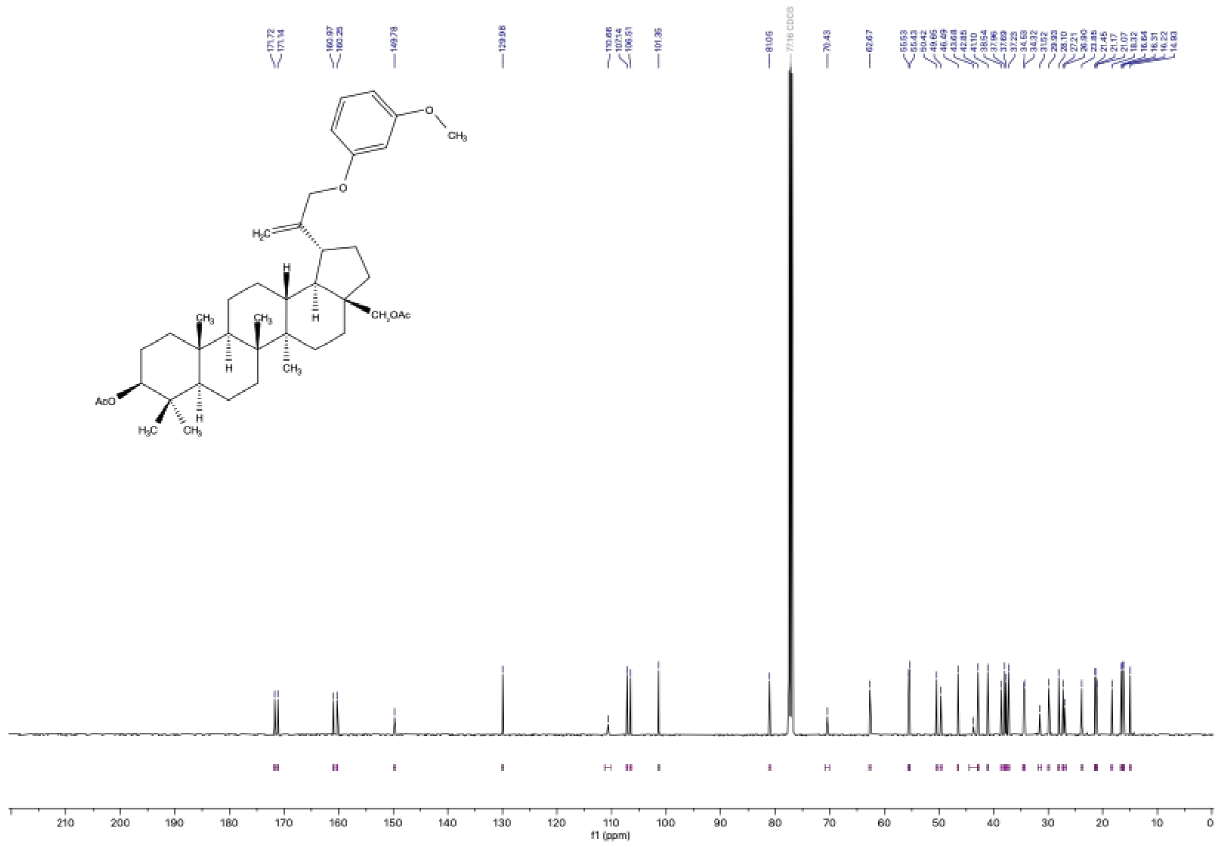

Figure S23. ^{13}C NMR spectrum of the compound **8** ($CDCl_3$, 126 MHz).

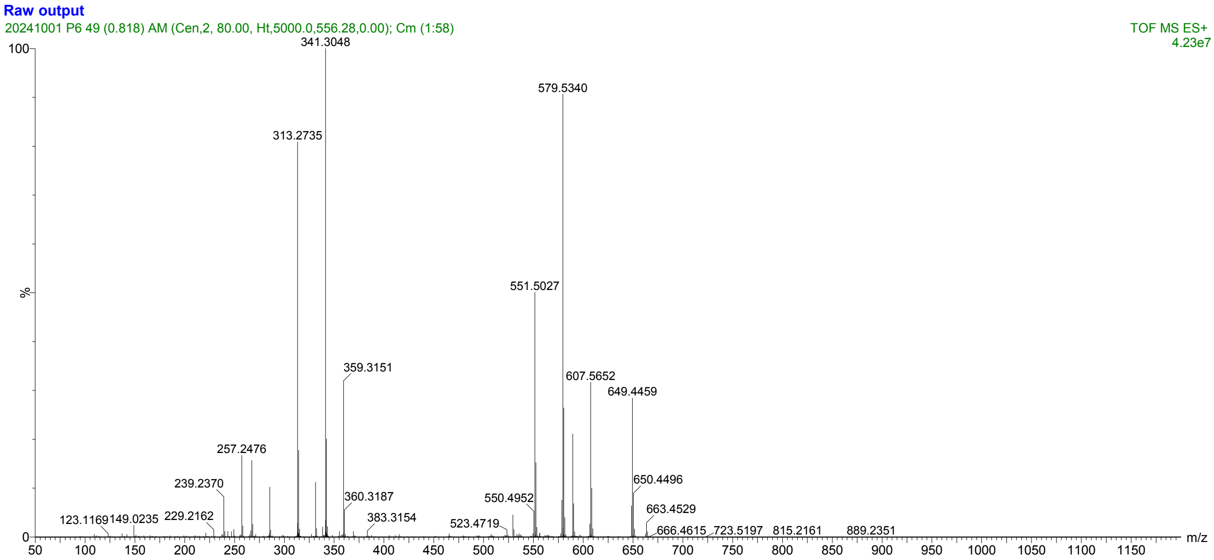

Figure S24. HRMS spectrum of the compound **8**.

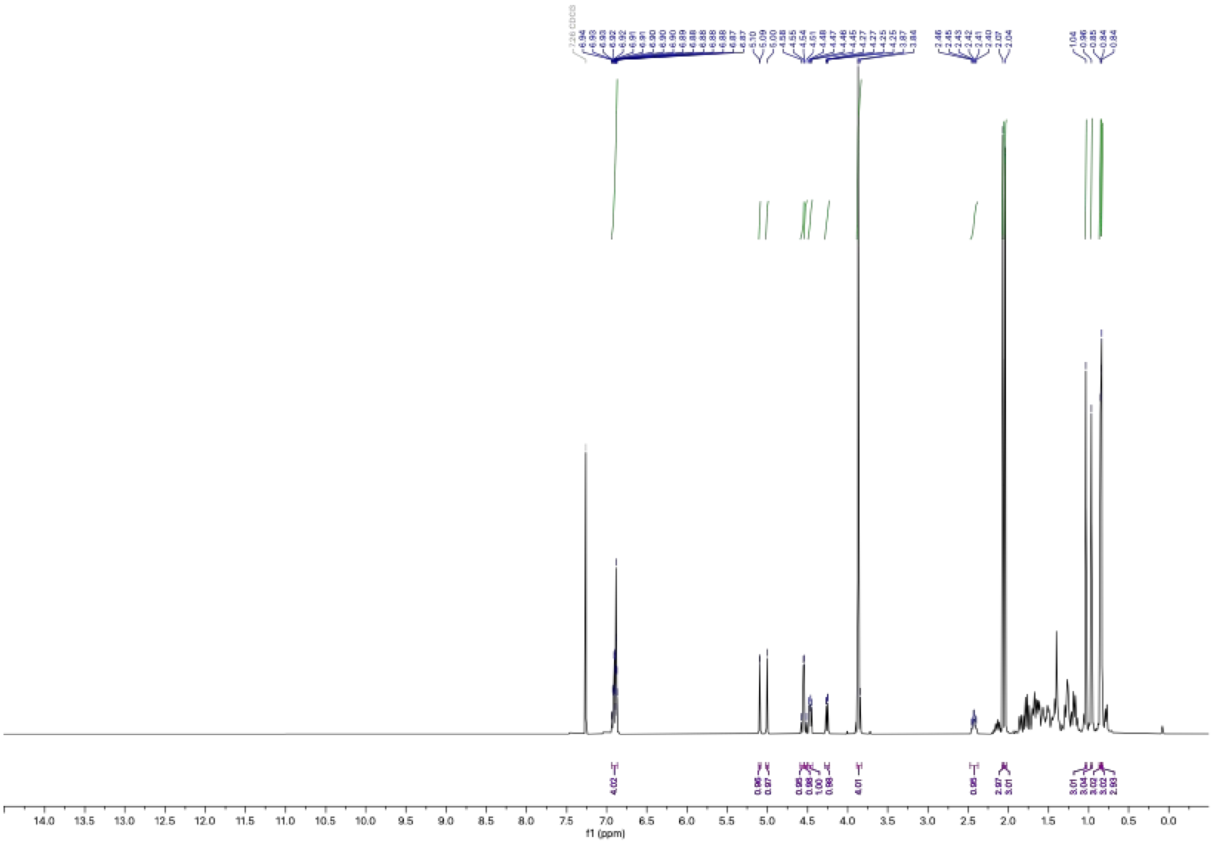

Figure S25. ^{1}H NMR spectrum of the compound **9** ($CDCl_3$, 500 MHz).

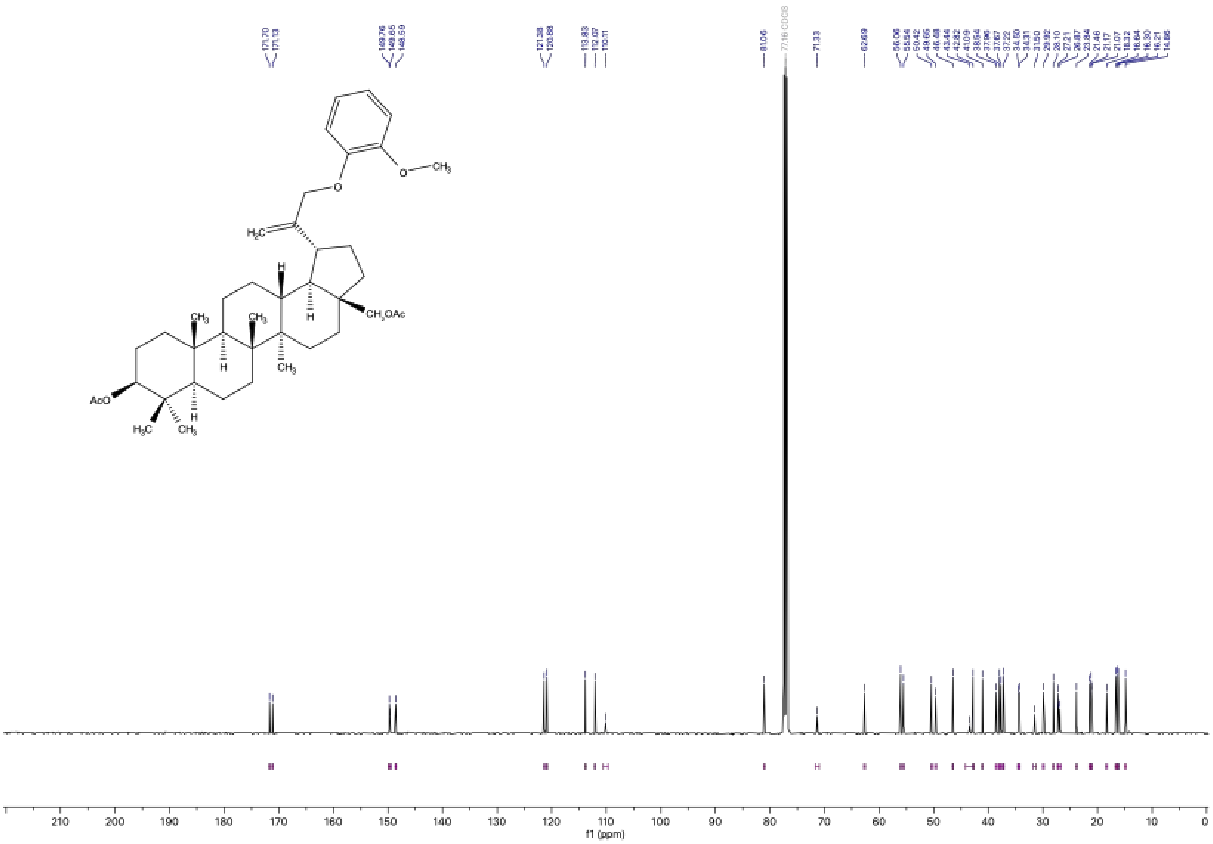

Figure S26. ^{13}C NMR spectrum of the compound **9** ($CDCl_3$, 126 MHz).

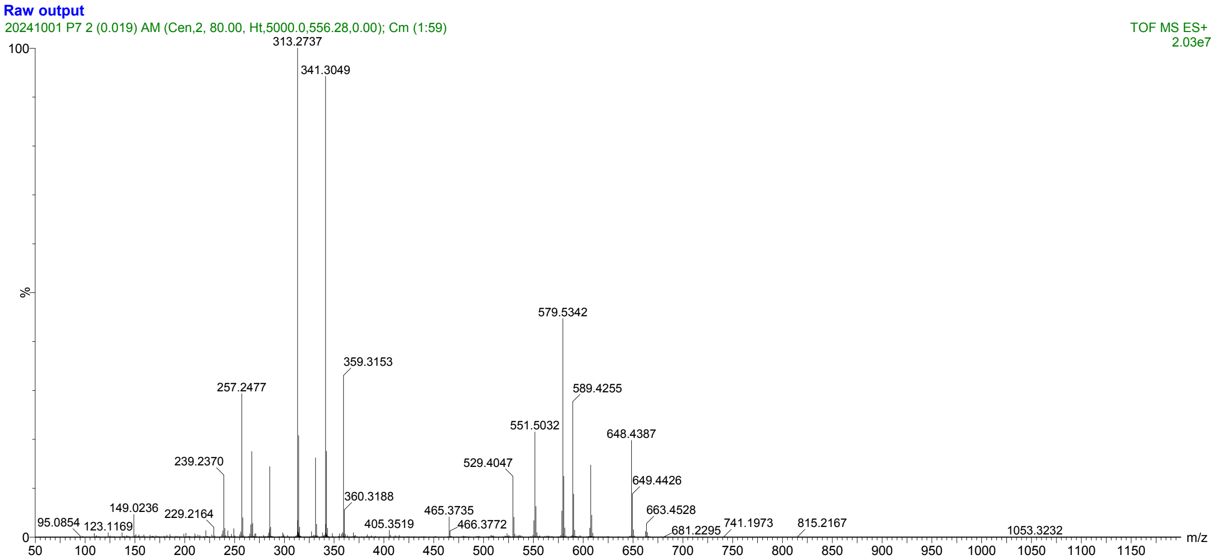

Figure S27. HRMS spectrum of the compound **9**.

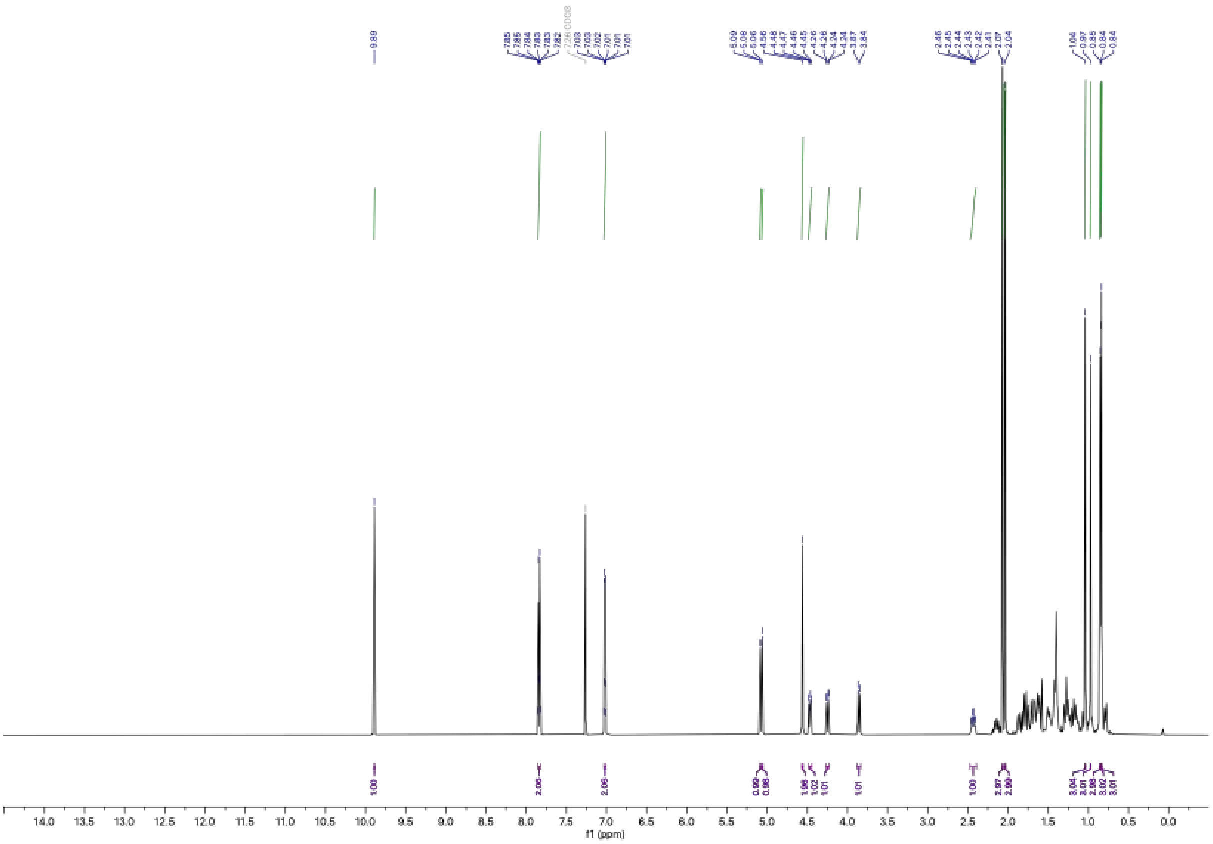

Figure S28. ^{1}H NMR spectrum of the compound **10** ($CDCl_3$, 500 MHz).

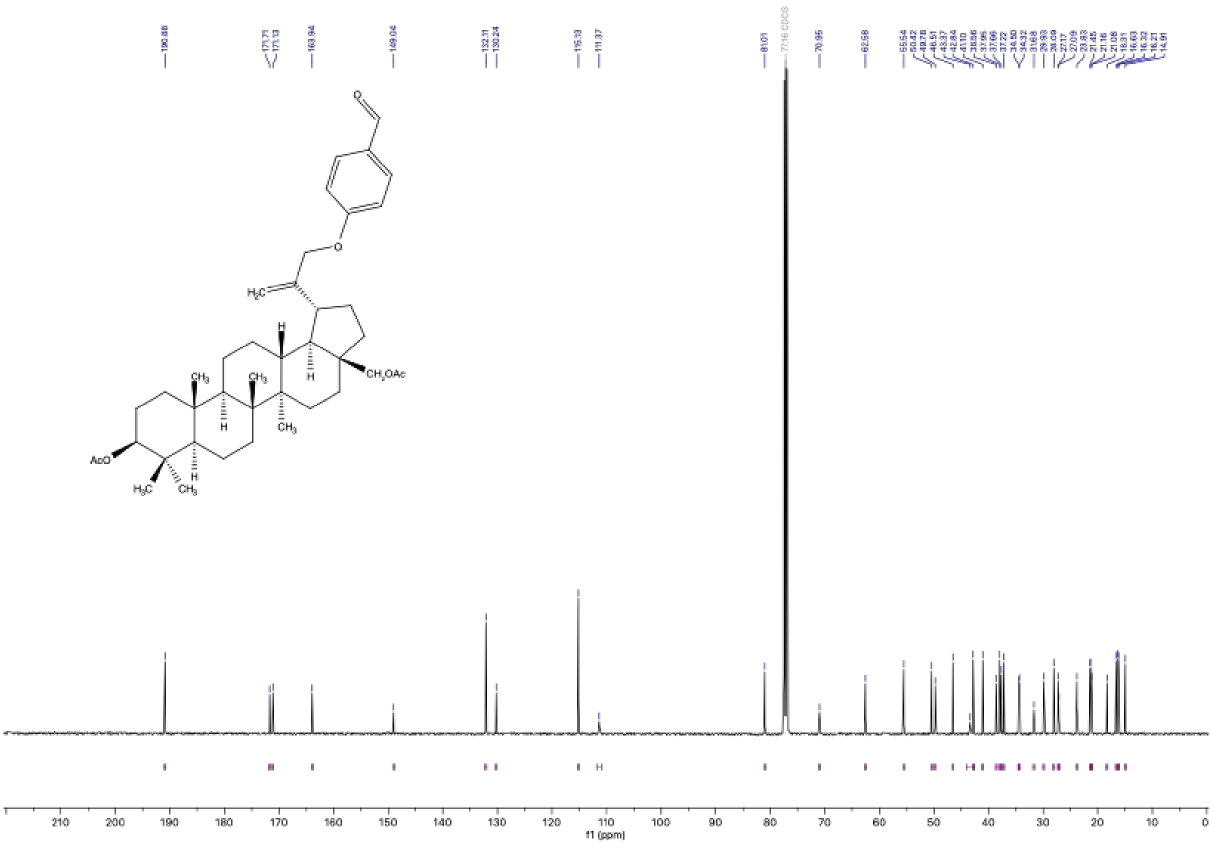

Figure S29. ^{13}C NMR spectrum of the compound **10** ($CDCl_3$, 126 MHz).

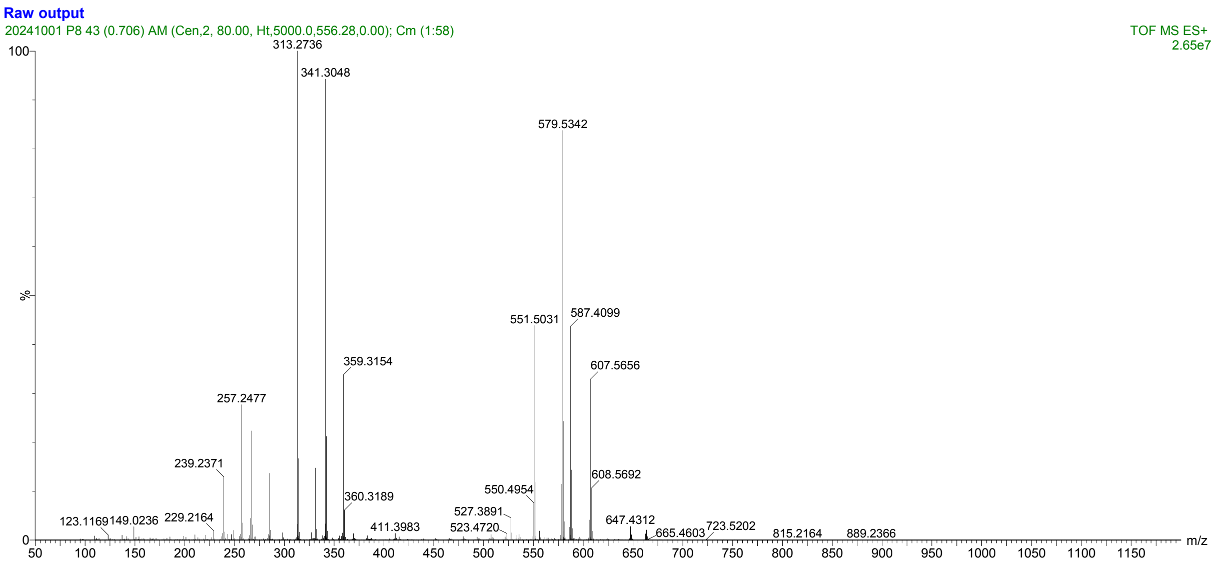

Figure S30. HRMS spectrum of the compound **10**.

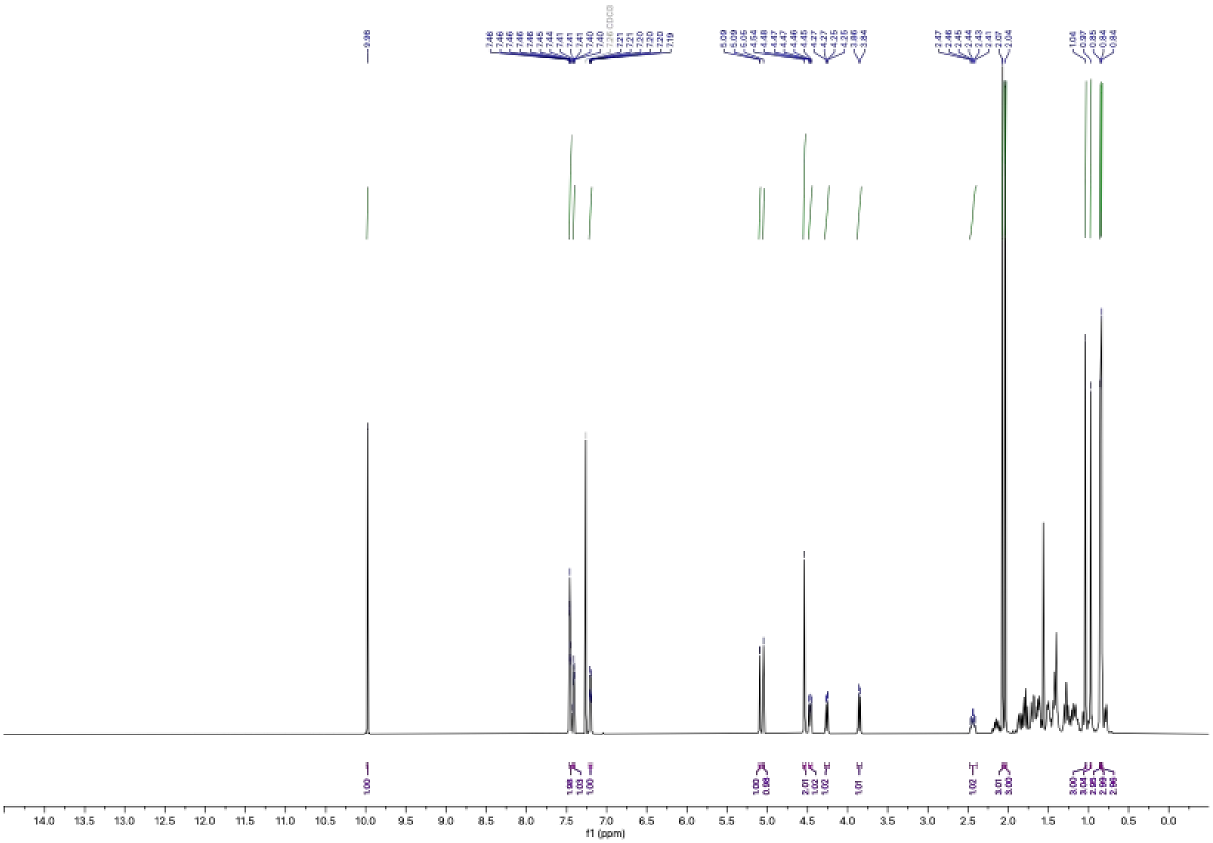

Figure S31. ^{1}H NMR spectrum of the compound **11** ($CDCl_3$, 500 MHz).

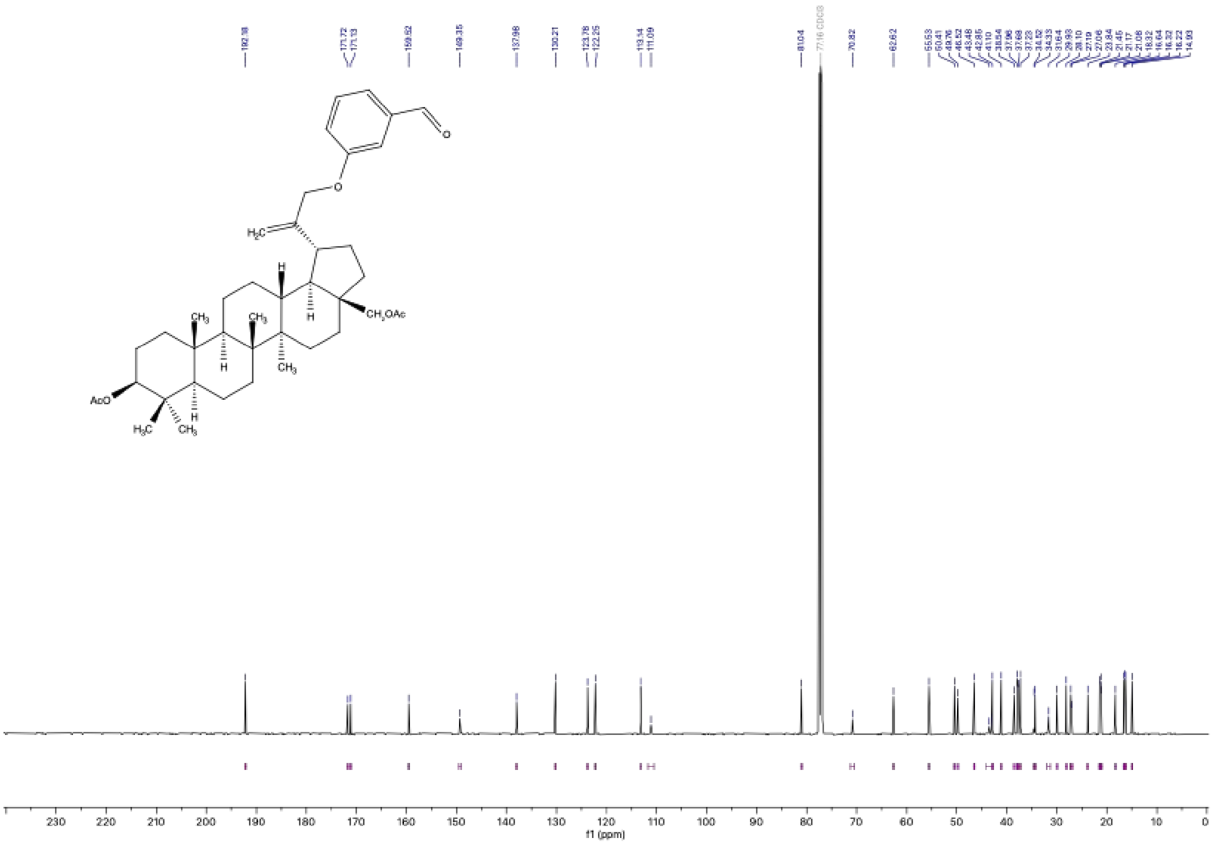

Figure S32. ^{13}C NMR spectrum of the compound **11** ($CDCl_3$, 126 MHz).

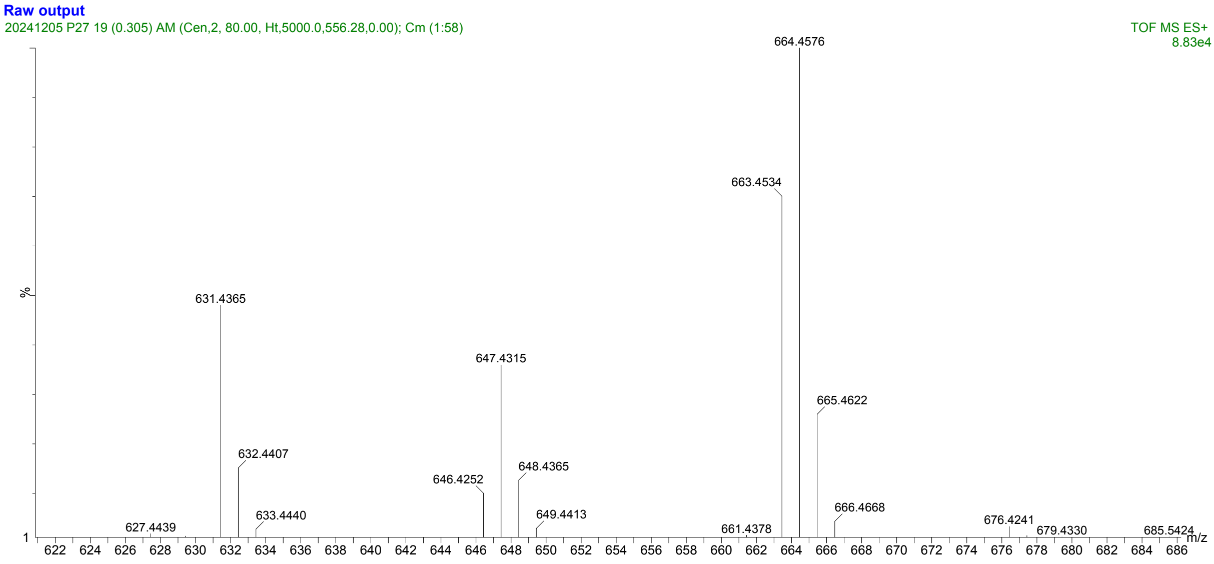

Figure S33. HRMS spectrum of the compound **11**.

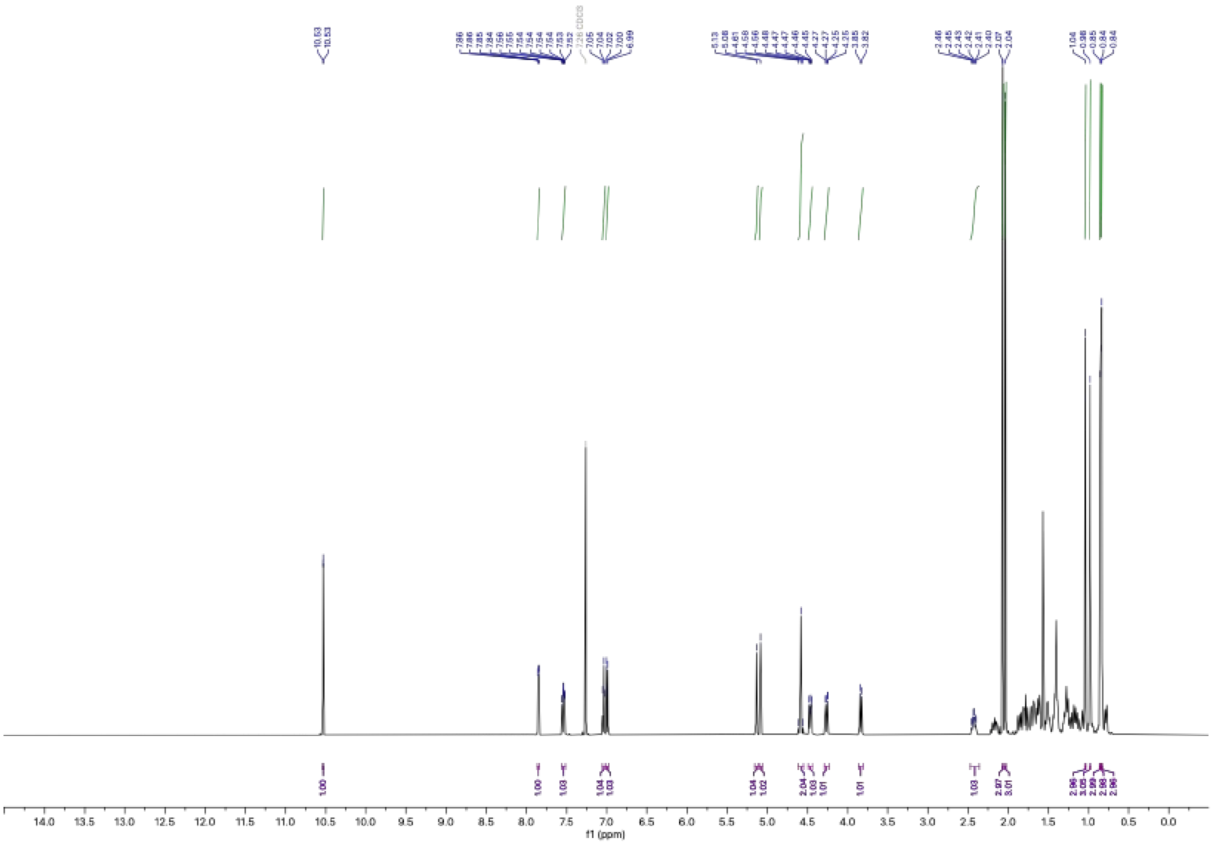

Figure S34. ^{1}H NMR spectrum of the compound **12** ($CDCl_3$, 500 MHz).

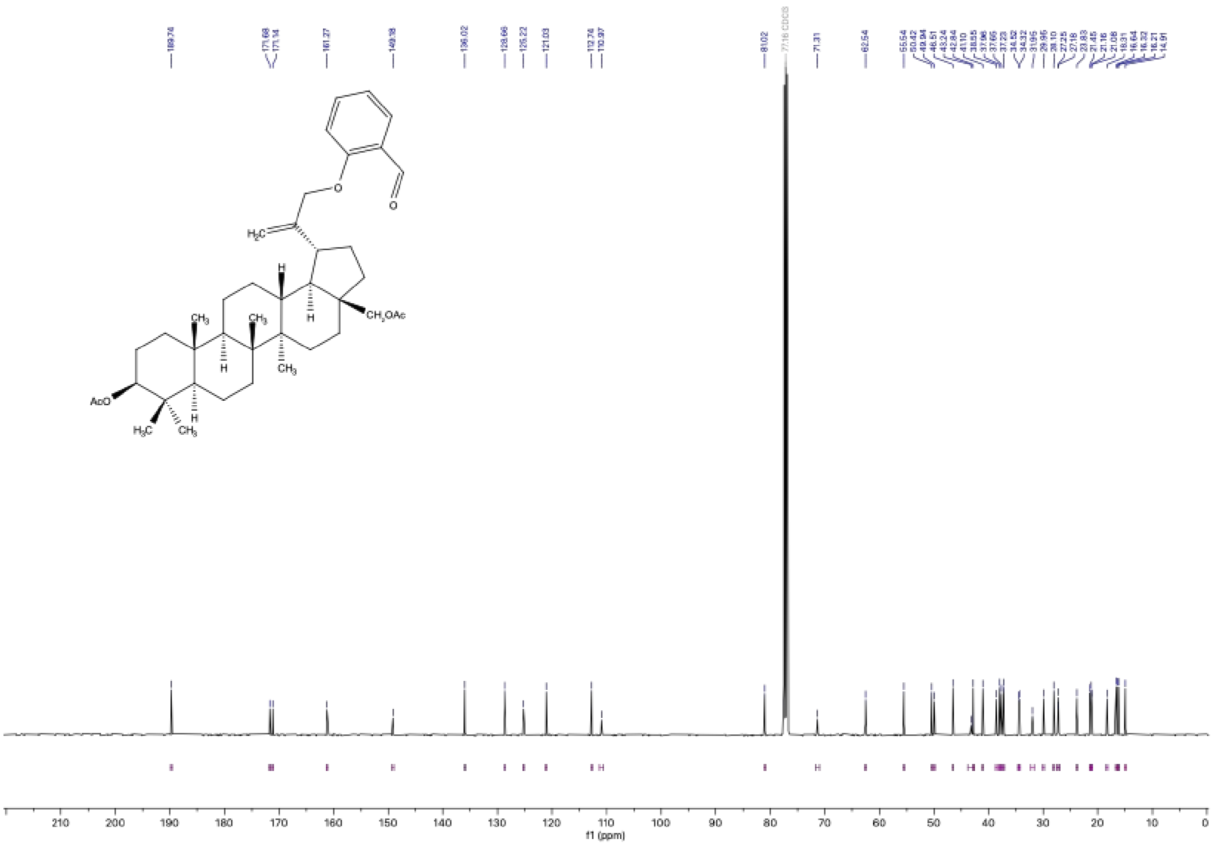

Figure S35. ^{13}C NMR spectrum of the compound **12** ($CDCl_3$, 126 MHz).

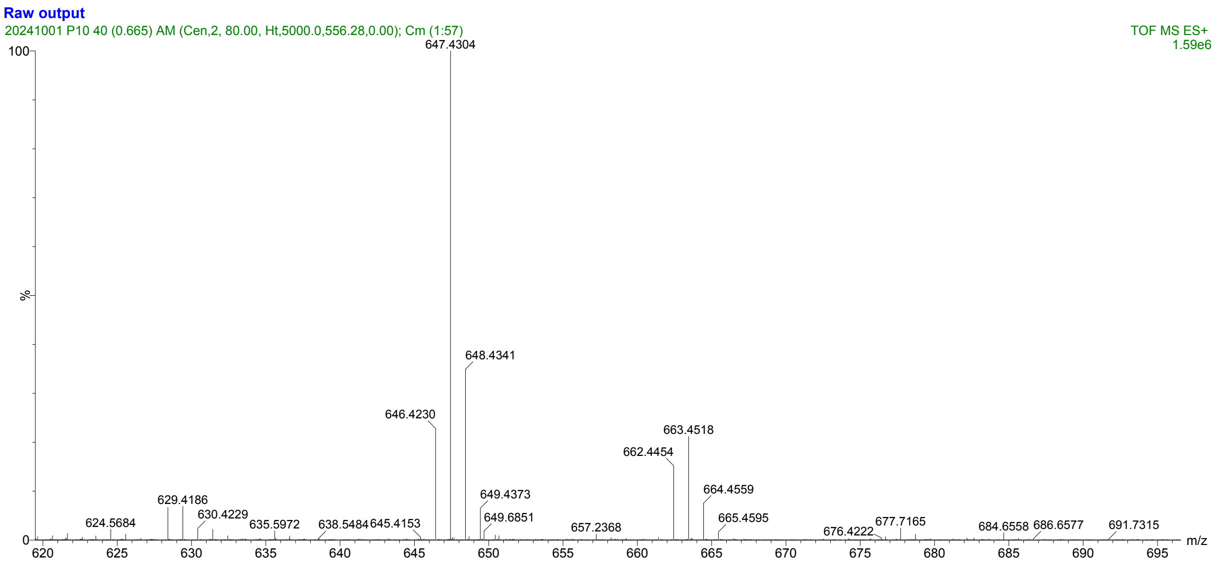

Figure S36. HRMS spectrum of the compound **12**.

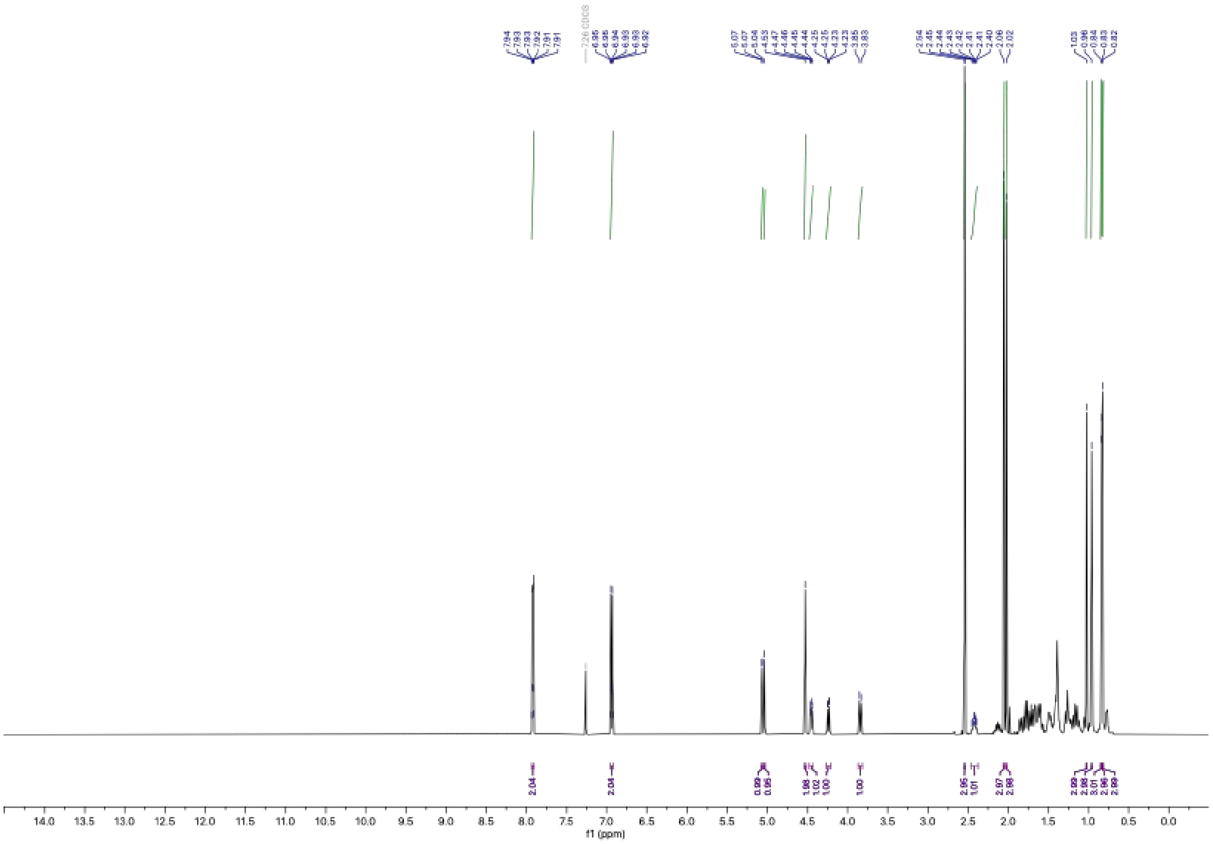

Figure S37. ^{1}H NMR spectrum of the compound **13** ($CDCl_3$, 500 MHz).

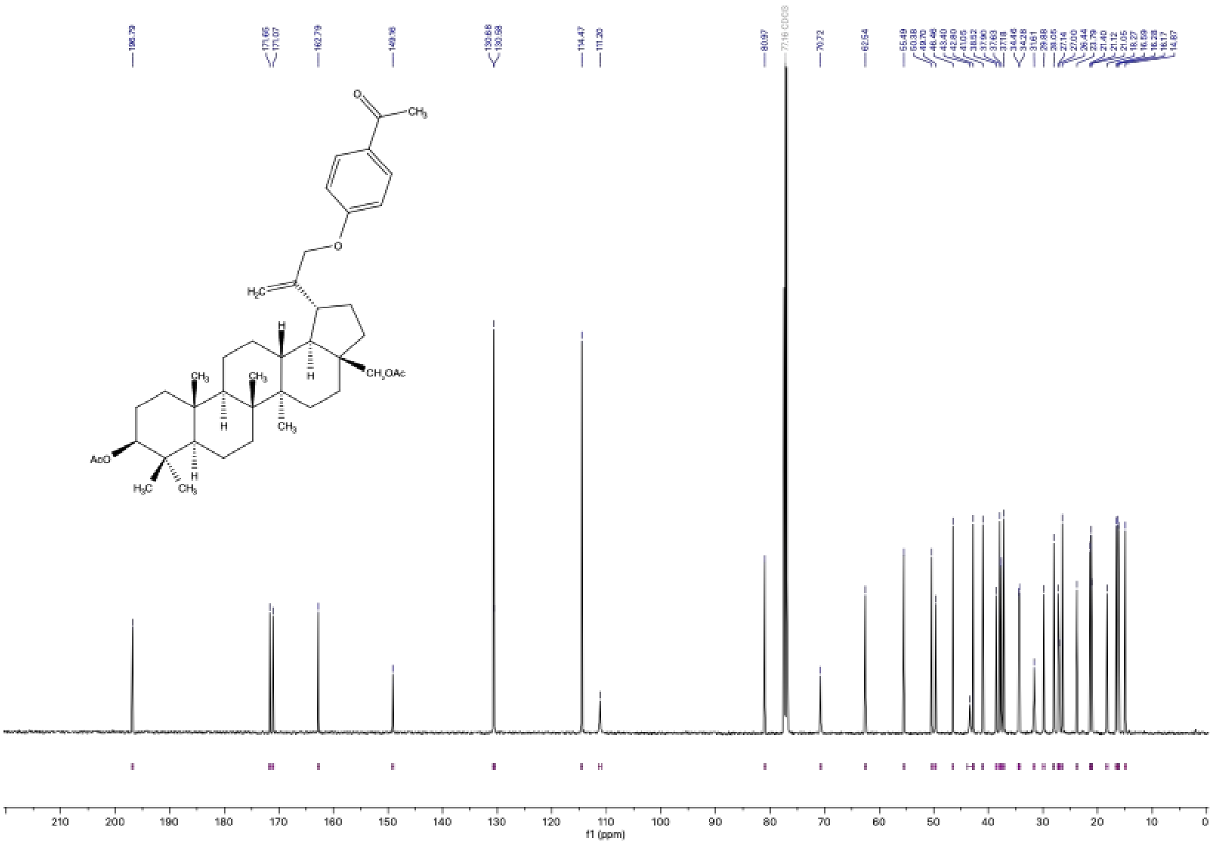

Figure S38. ^{13}C NMR spectrum of the compound **13** ($CDCl_3$, 126 MHz).

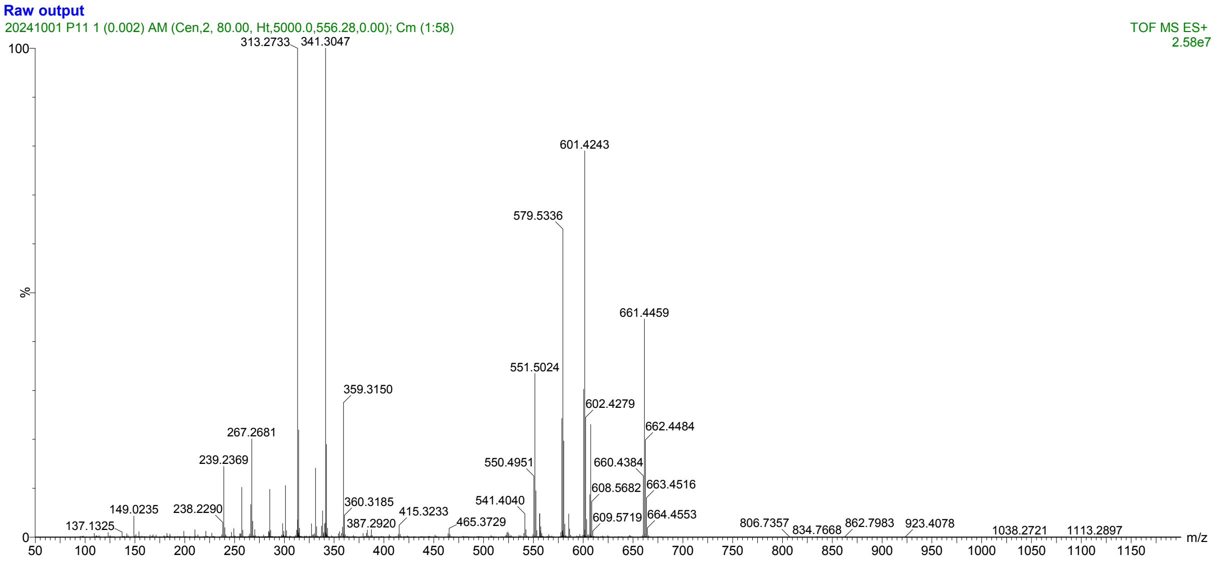

Figure S39. HRMS spectrum of the compound **13**.

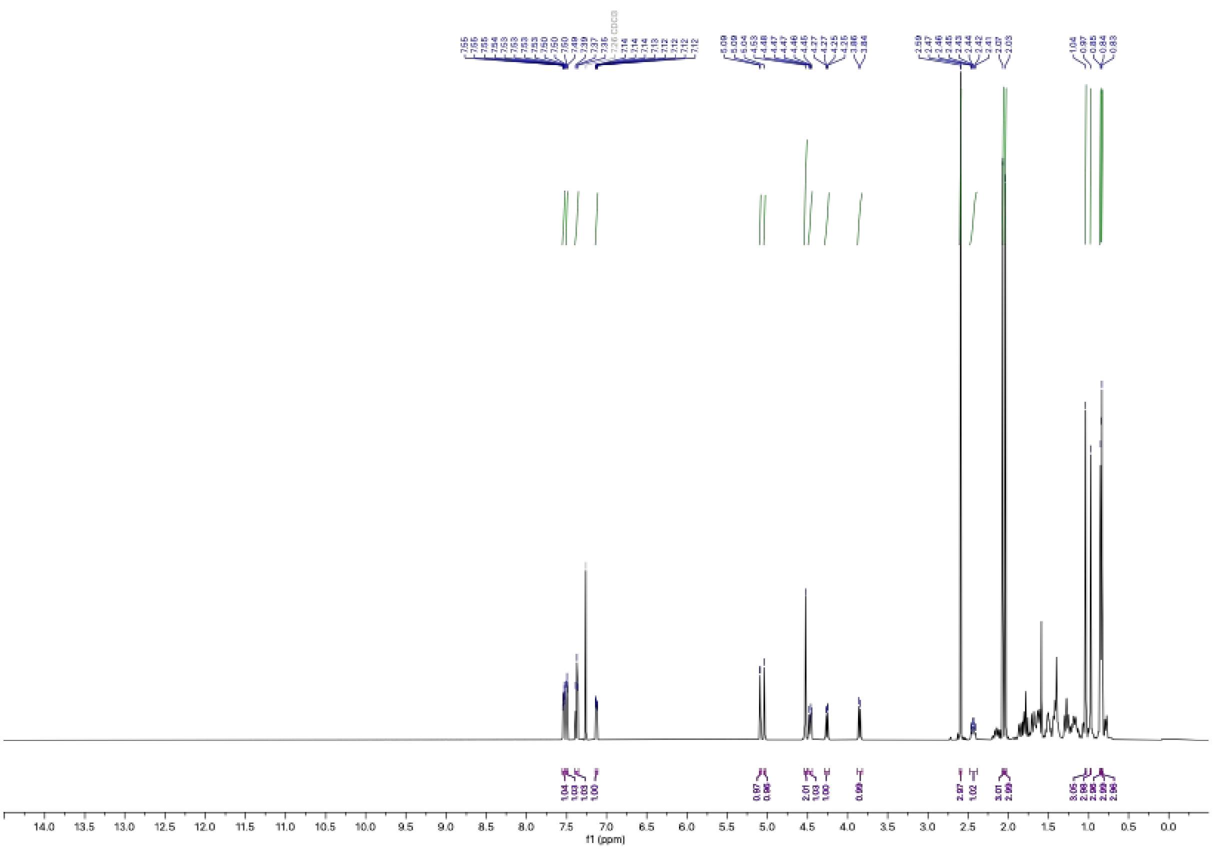

Figure S40. ^{1}H NMR spectrum of the compound **14** ($CDCl_3$, 500 MHz).

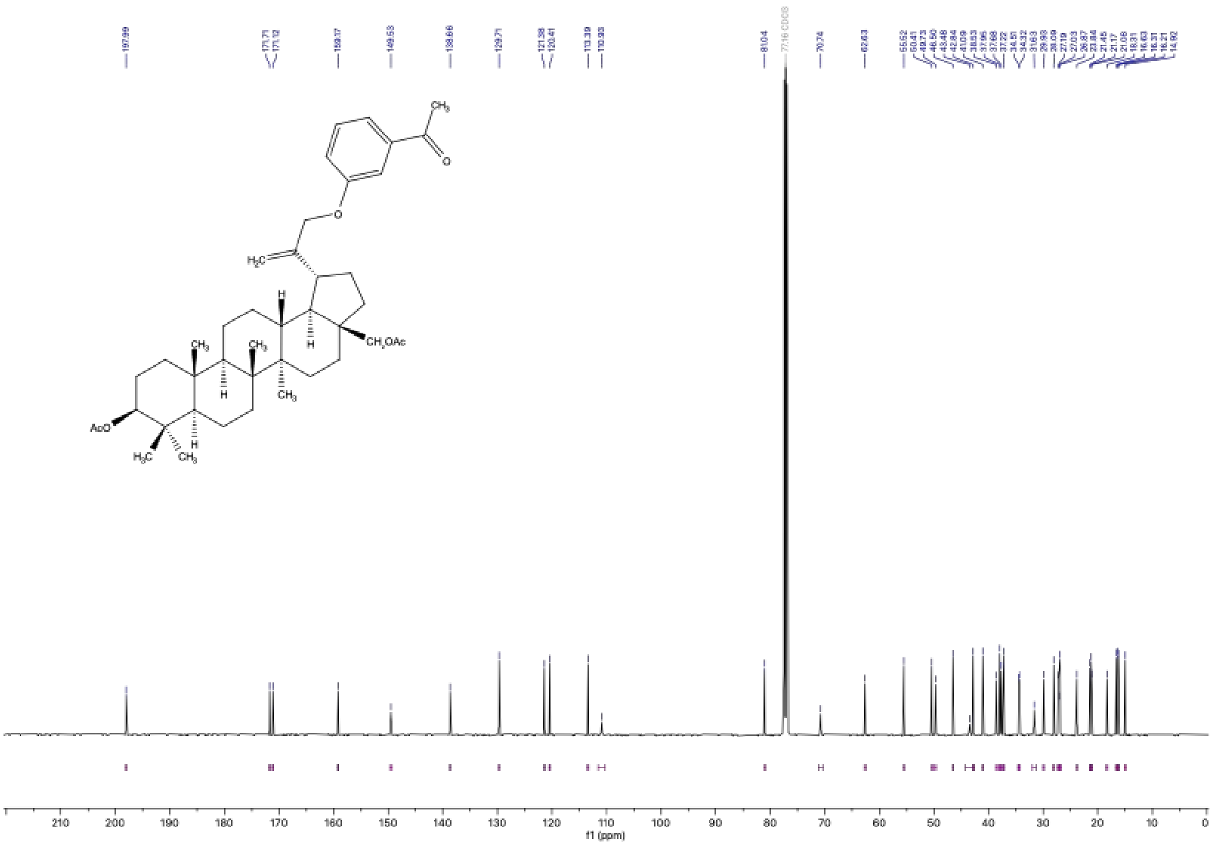

Figure S41. ^{13}C NMR spectrum of the compound **14** ($CDCl_3$, 126 MHz).

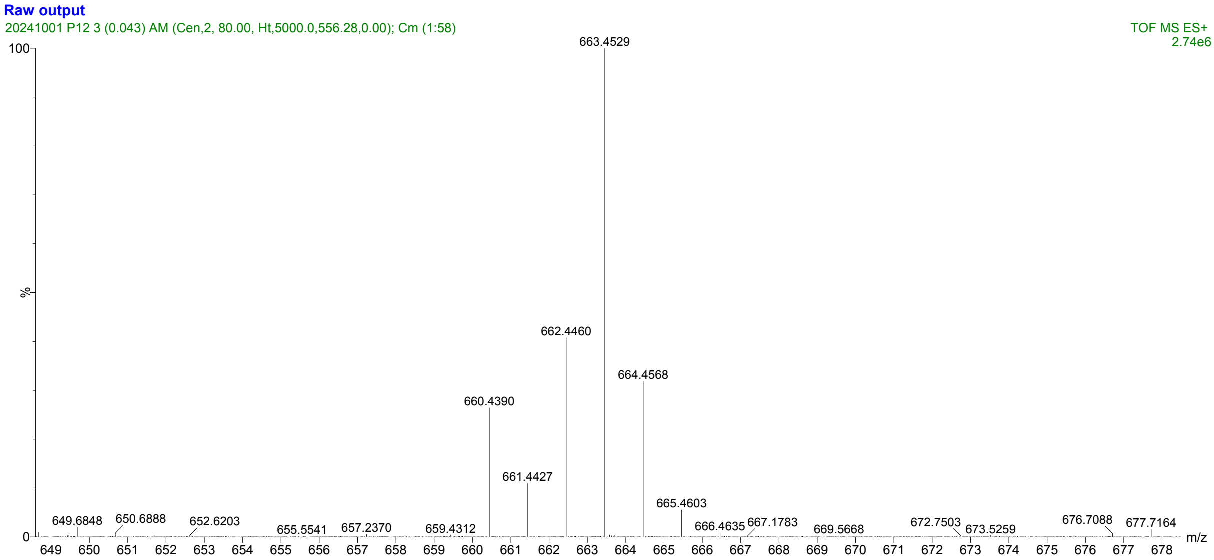

Figure S42. HRMS spectrum of the compound **14**.

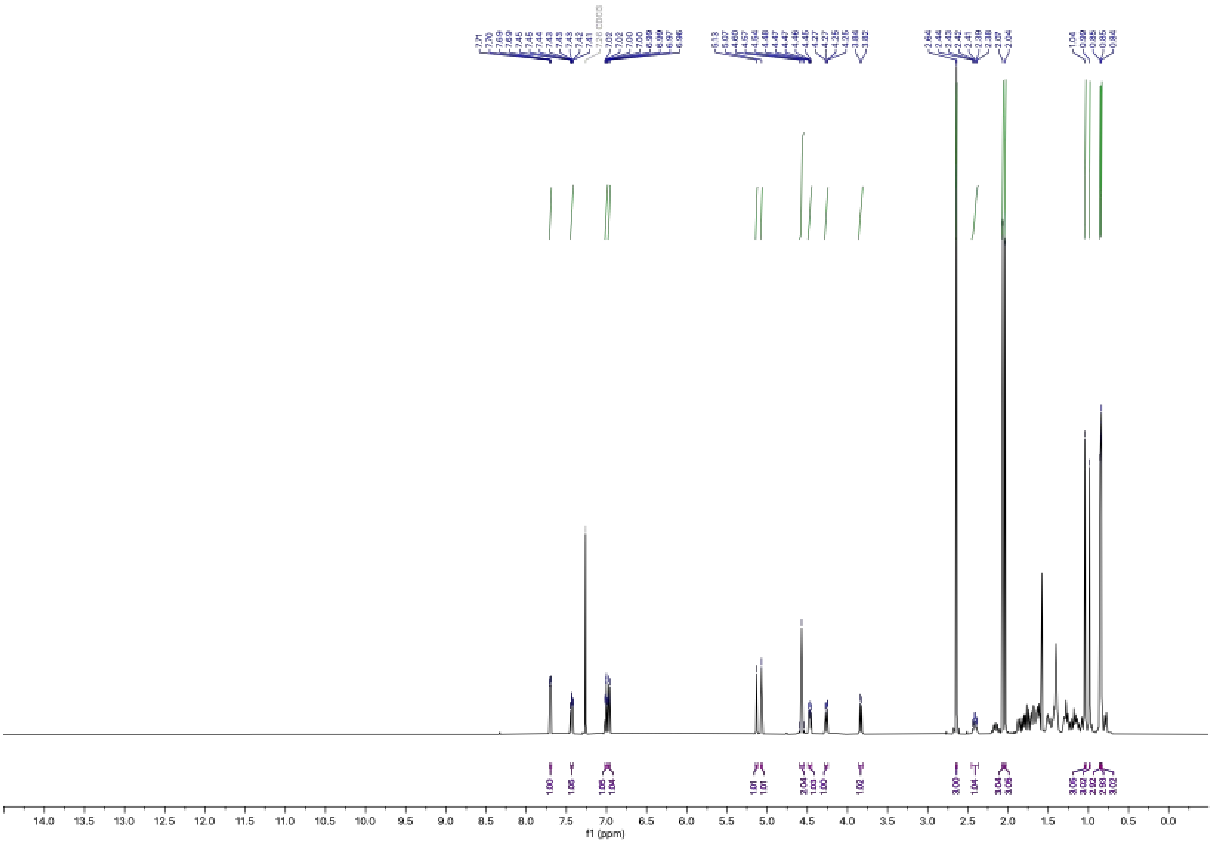

Figure S43. ^{1}H NMR spectrum of the compound **15** ($CDCl_3$, 500 MHz).

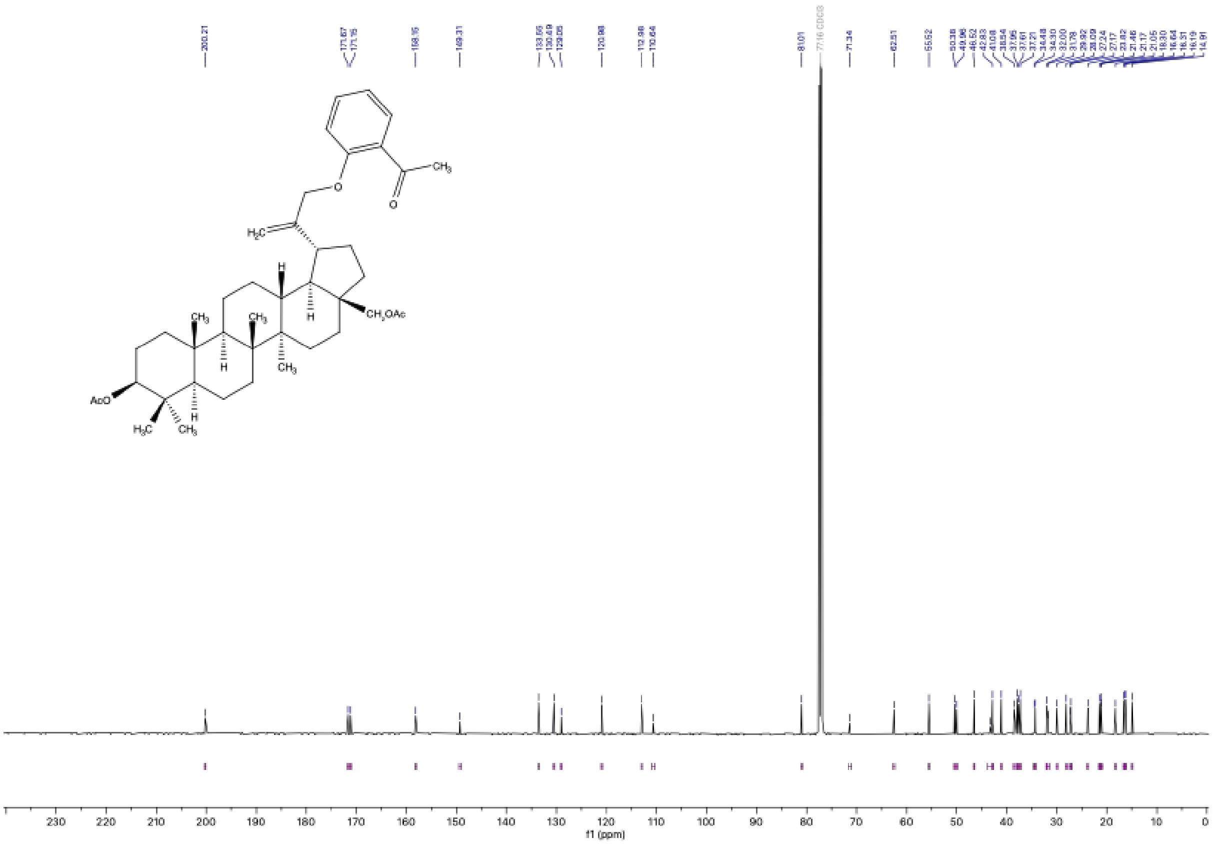

Figure S44. ^{13}C NMR spectrum of the compound **15** ($CDCl_3$, 126 MHz).

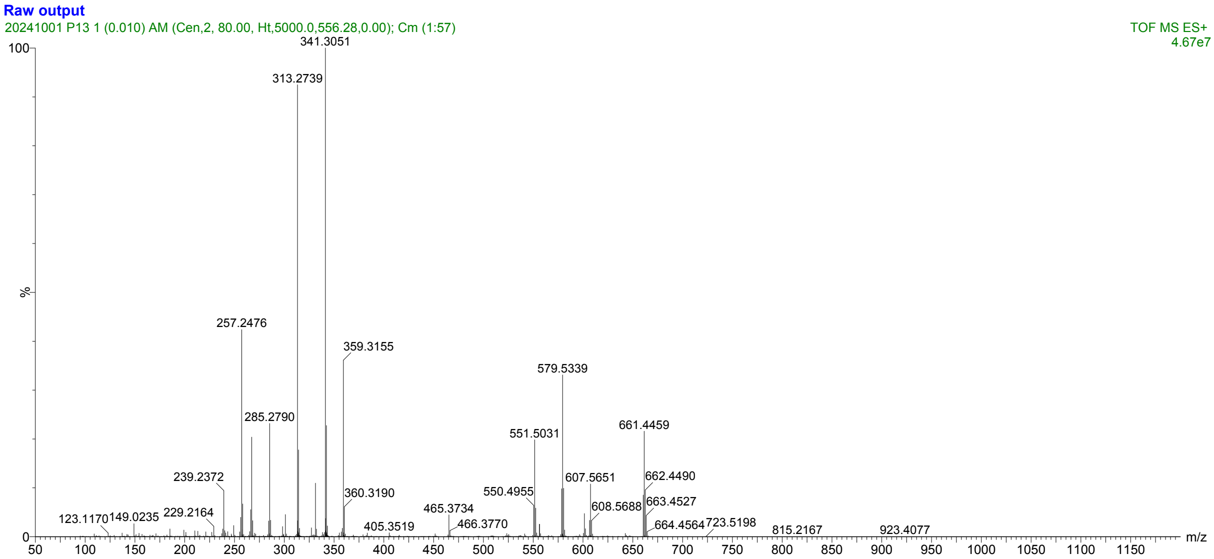

Figure S45. HRMS spectrum of the compound **15**.

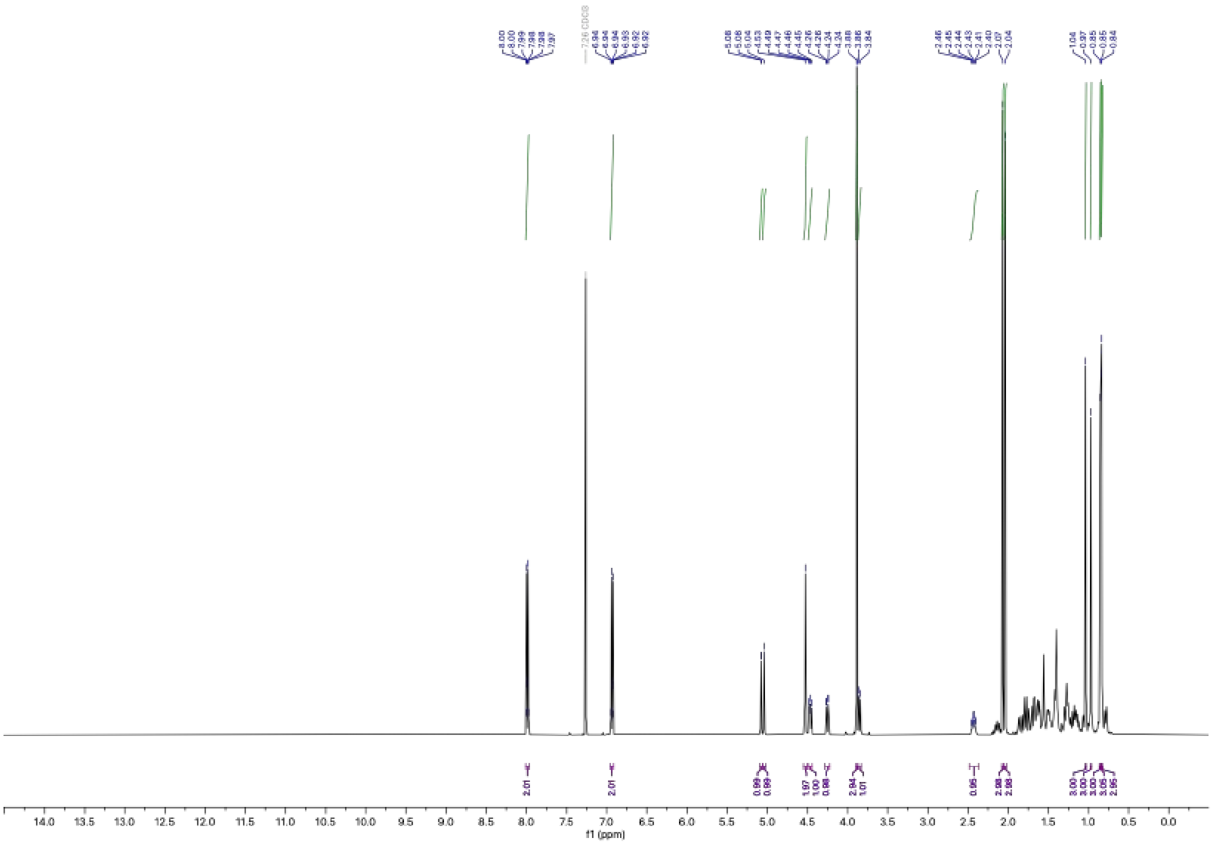

Figure S46. ^{1}H NMR spectrum of the compound **16** ($CDCl_3$, 500 MHz).

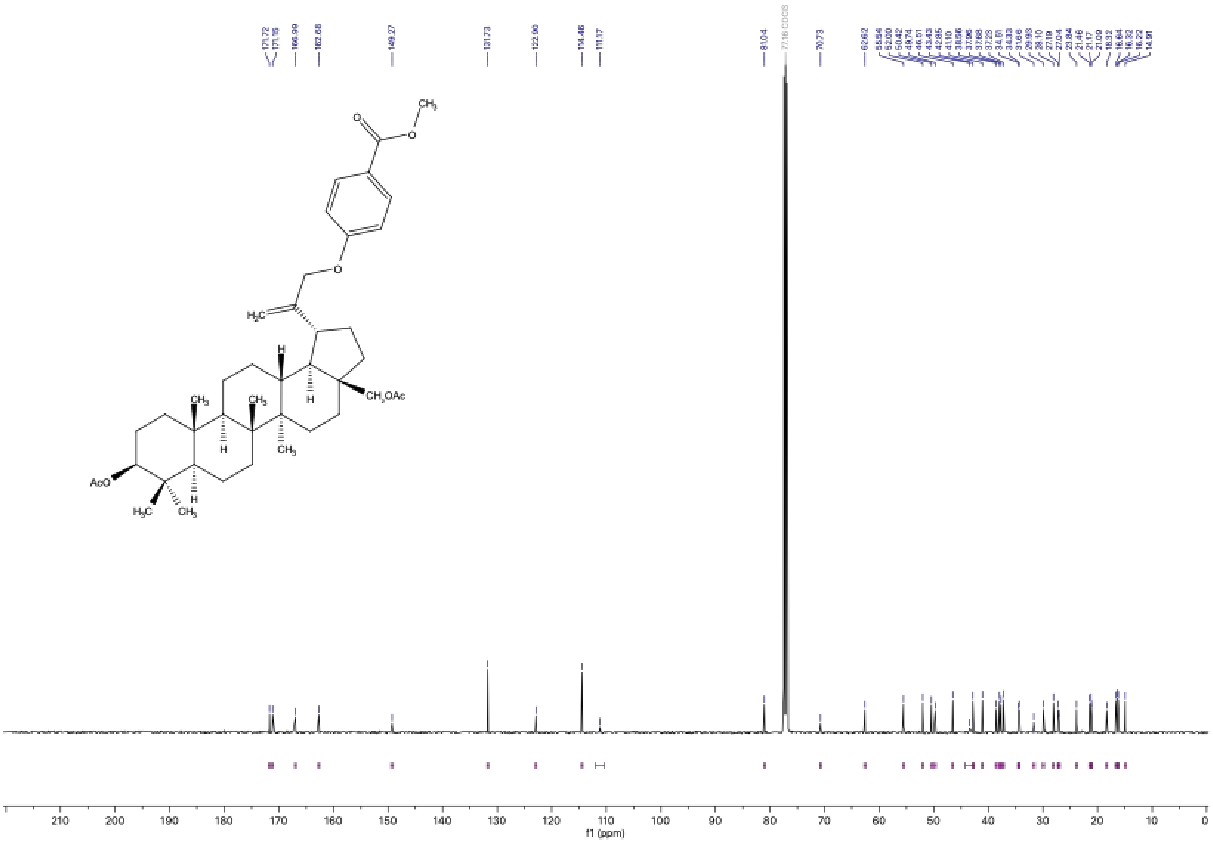

Figure S47. ^{13}C NMR spectrum of the compound **16** ($CDCl_3$, 126 MHz).

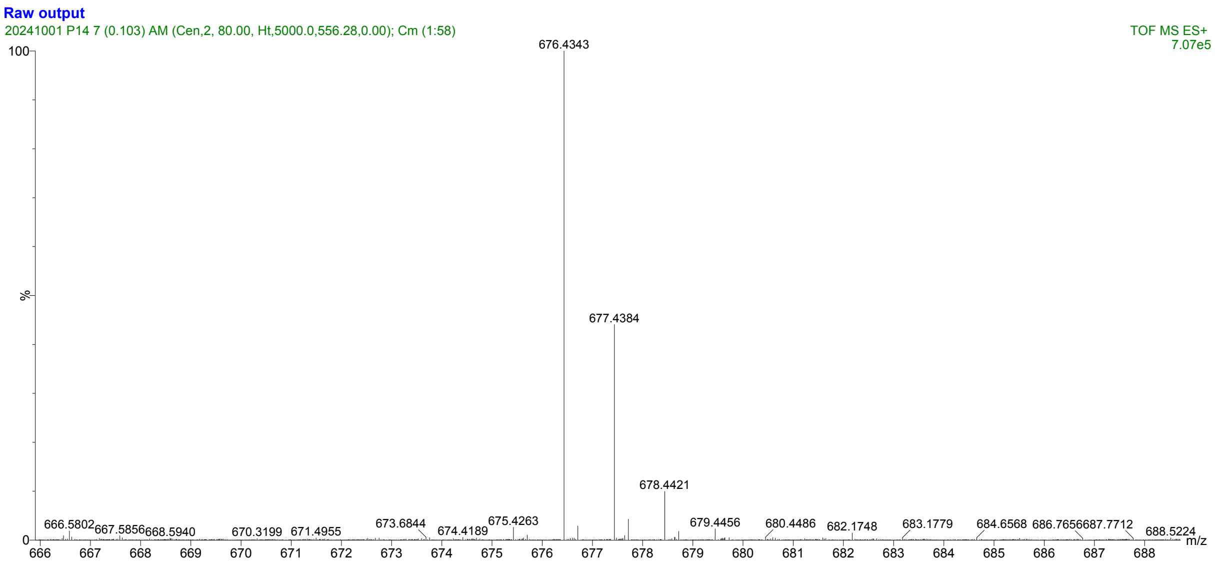

Figure S48. HRMS spectrum of the compound **16**.

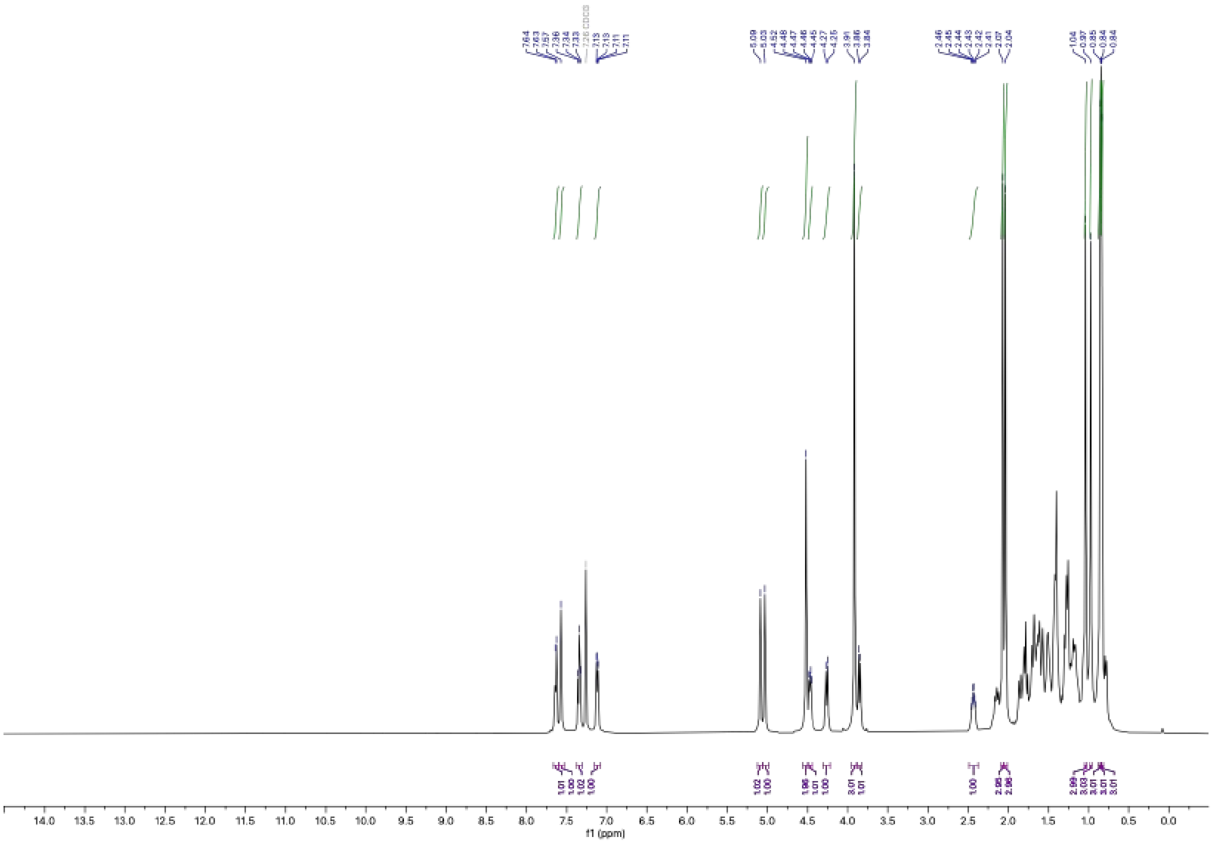

Figure S49. ^{1}H NMR spectrum of the compound **17** ($CDCl_3$, 500 MHz).

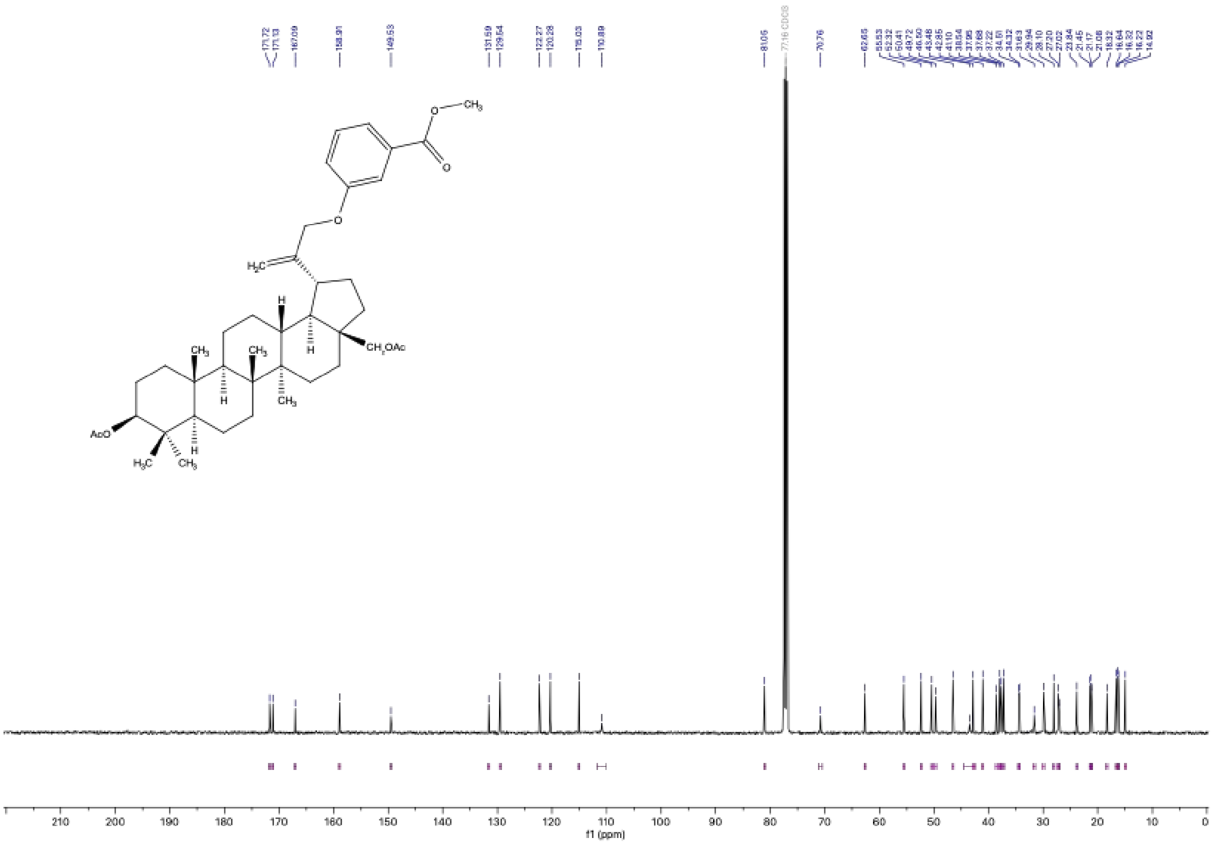

Figure S50. ^{13}C NMR spectrum of the compound **17** ($CDCl_3$, 126 MHz).

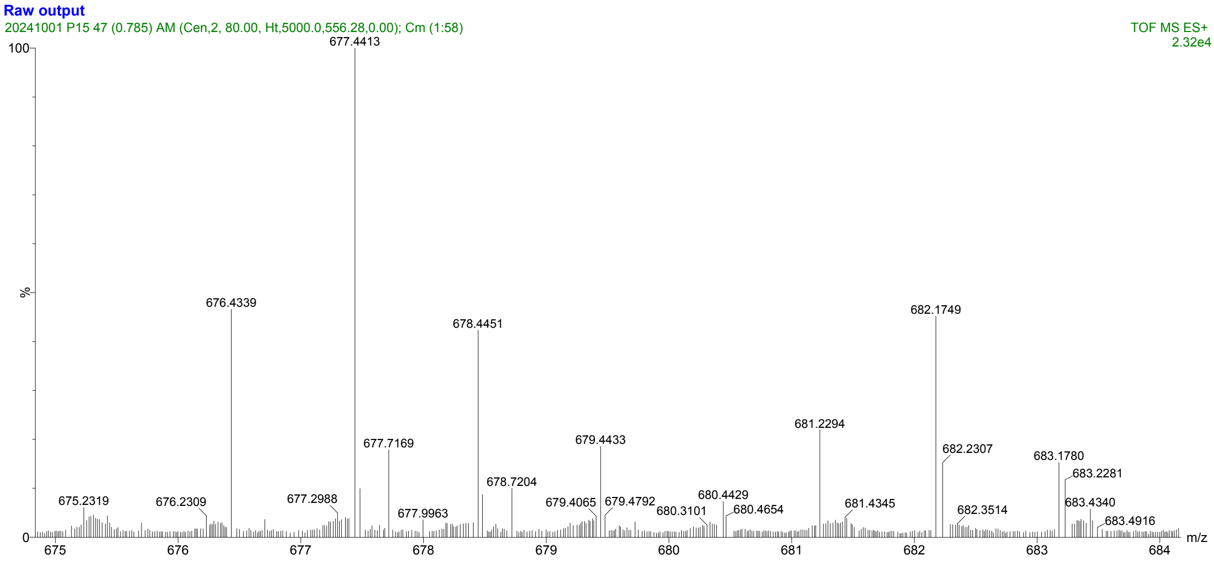

Figure S51. HRMS spectrum of the compound **17**.

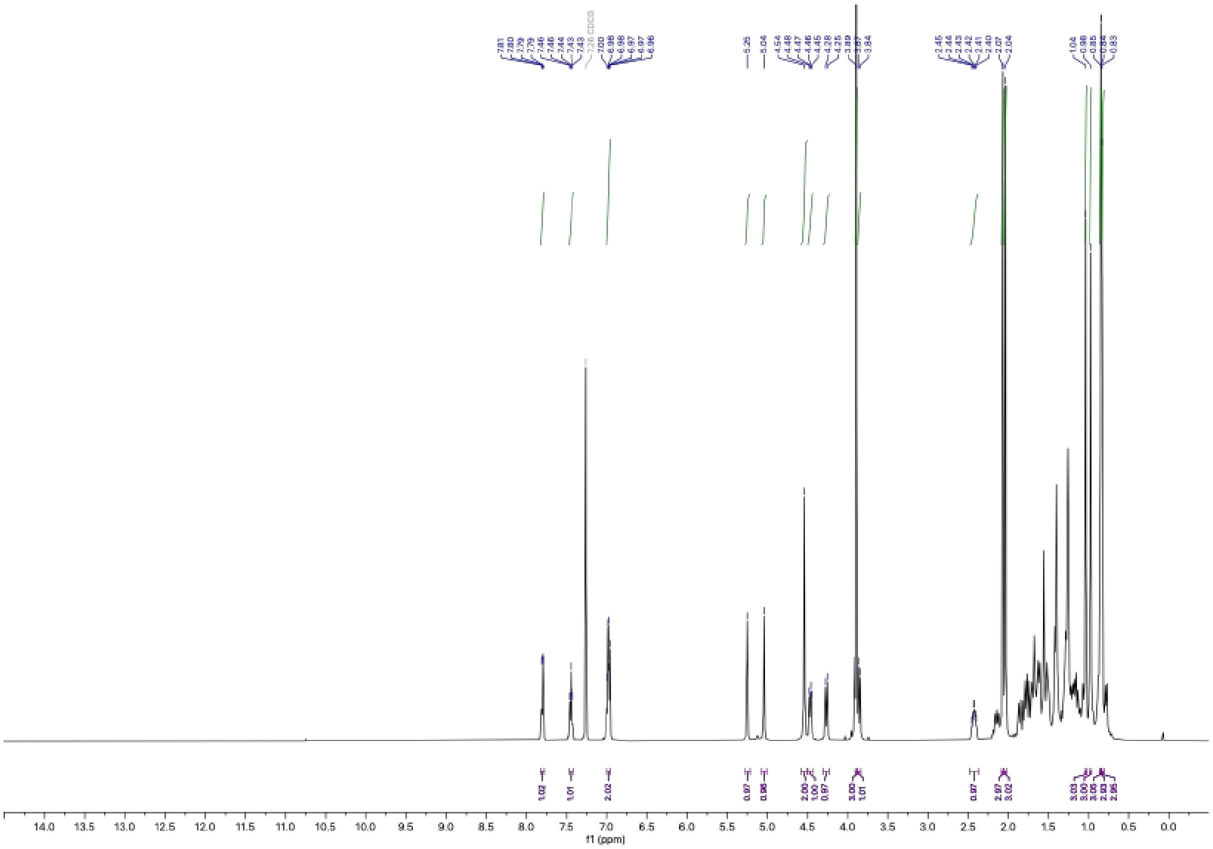

Figure S52. ^{1}H NMR spectrum of the compound **18** ($CDCl_3$, 500 MHz).

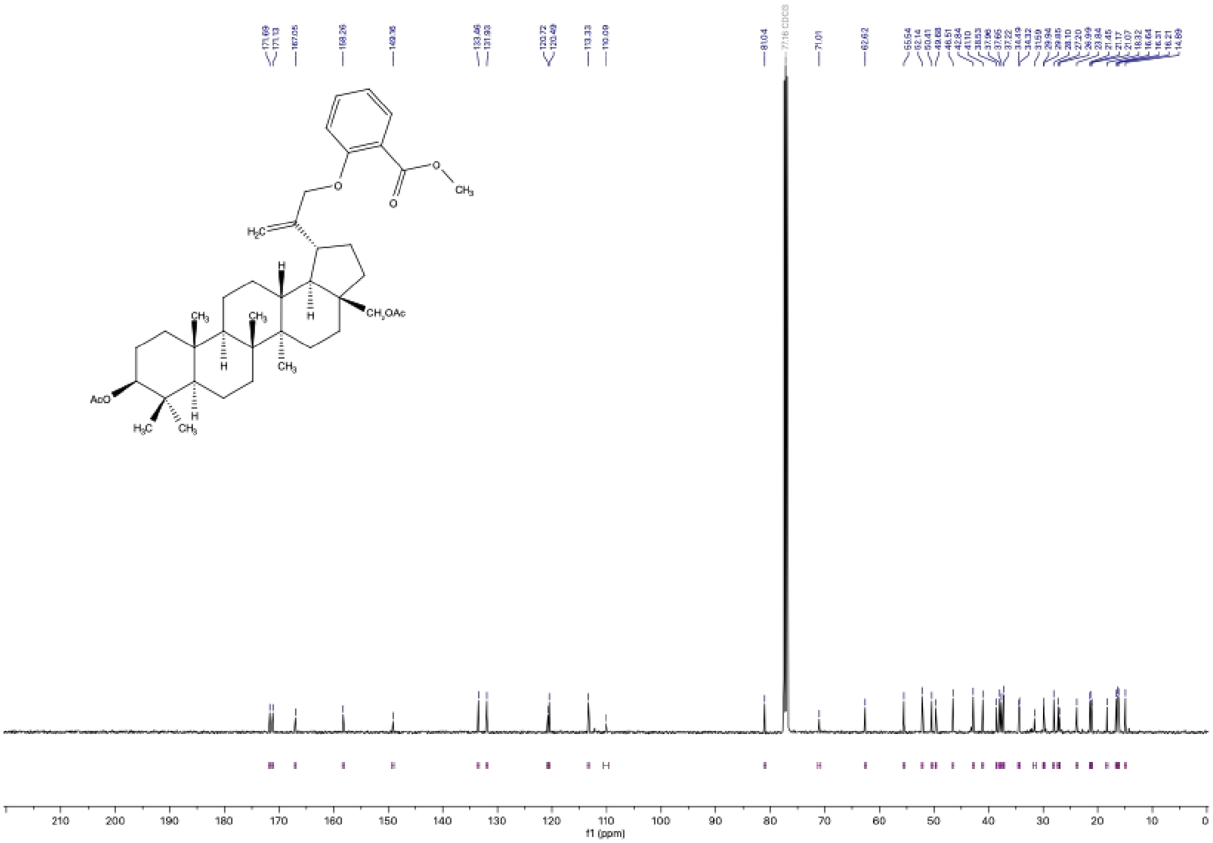

Figure S53. ^{13}C NMR spectrum of the compound **18** ($CDCl_3$, 126 MHz).

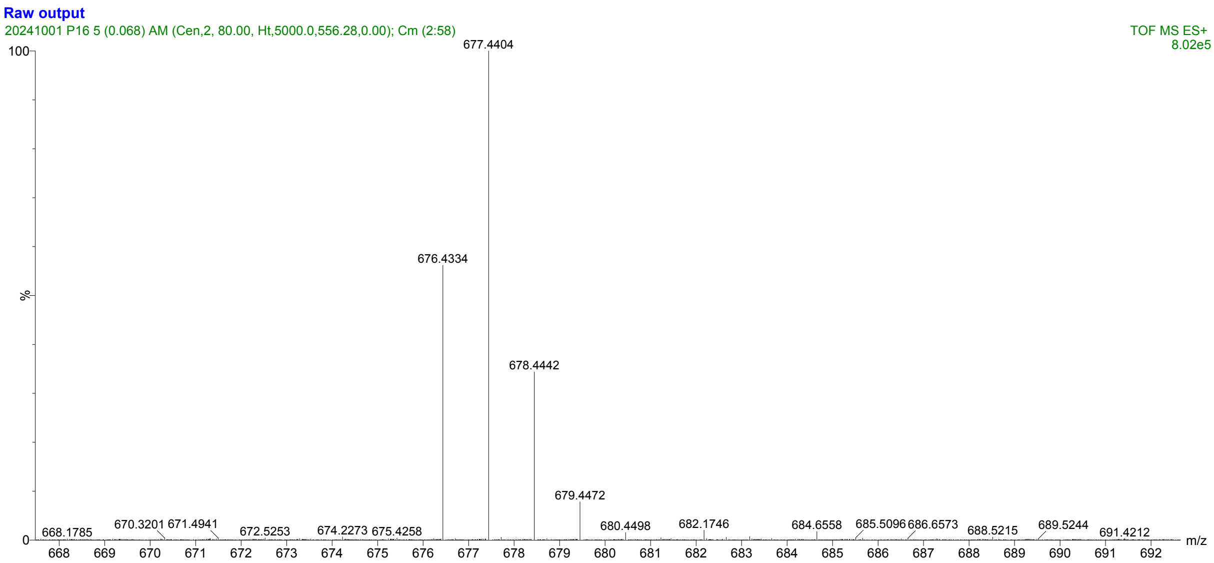

Figure S54. HRMS spectrum of the compound **18**.

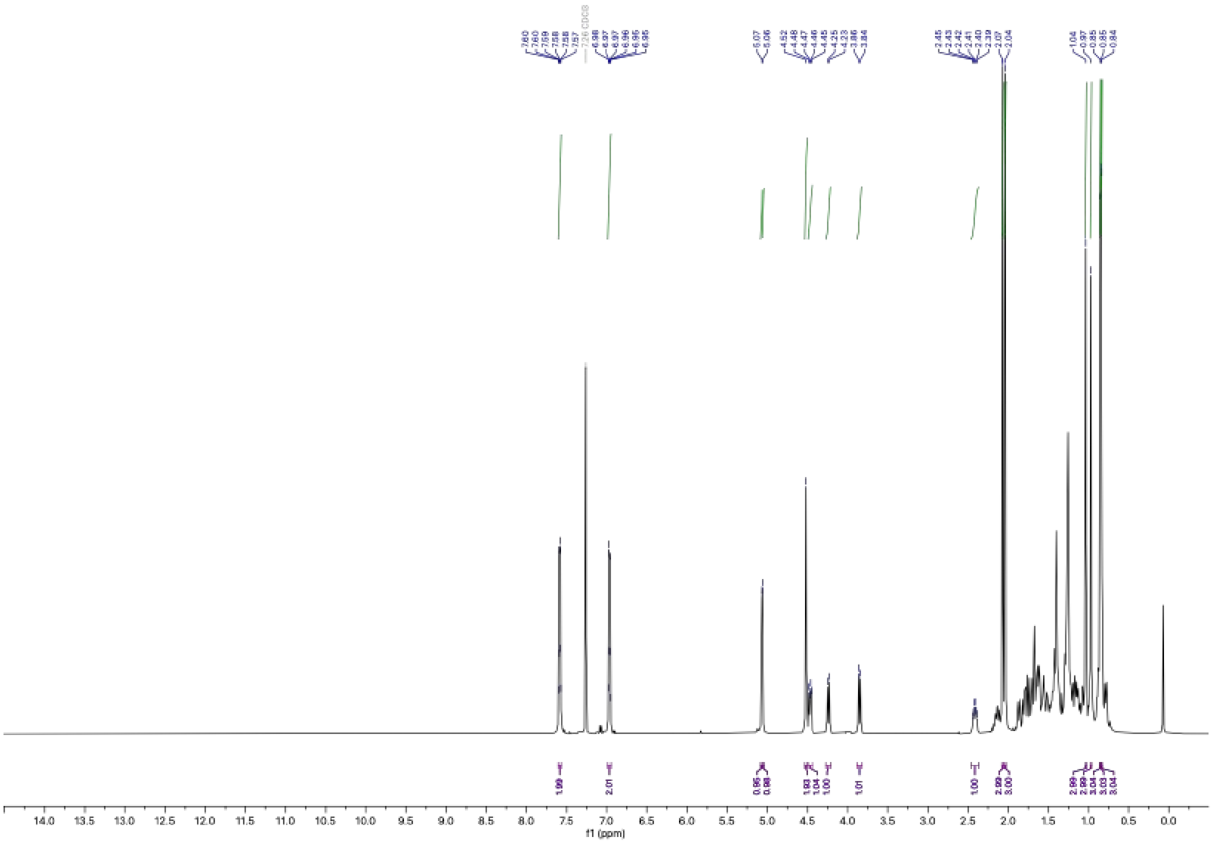

Figure S55. ^{1}H NMR spectrum of the compound **19** ($CDCl_3$, 500 MHz, contains also residual TMS).

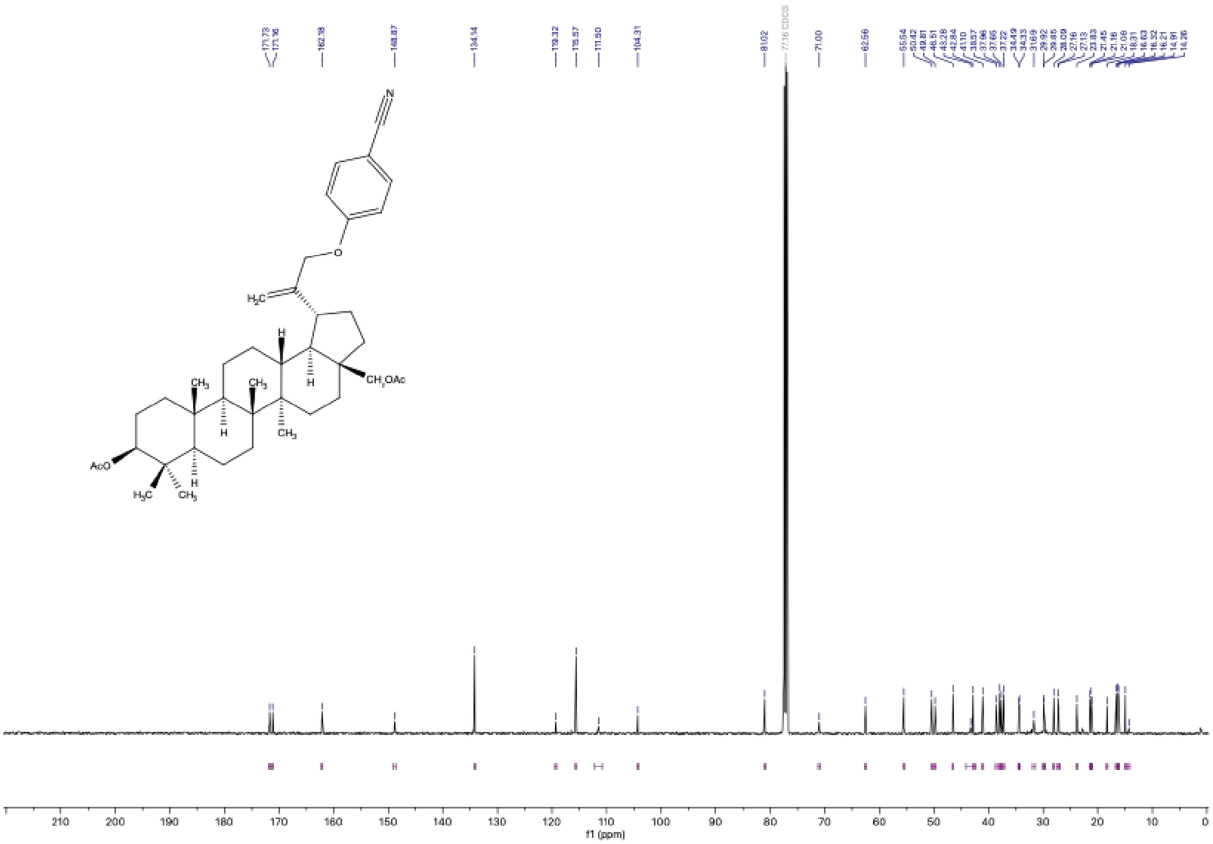

Figure S56. ^{13}C NMR spectrum of the compound **19** ($CDCl_3$, 126 MHz).

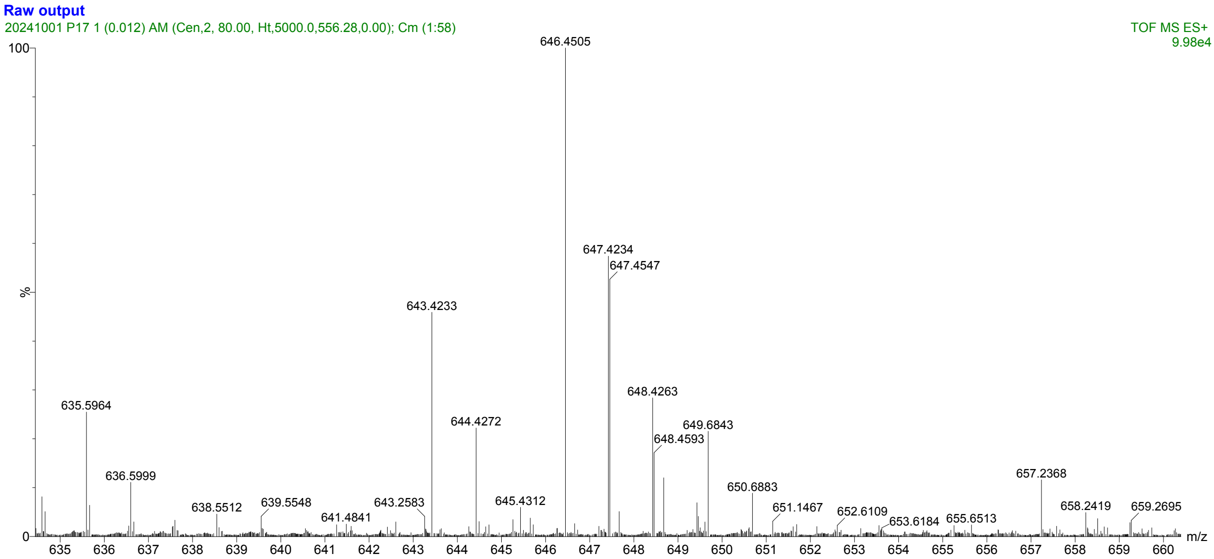

Figure S57. HRMS spectrum of the compound **19**.

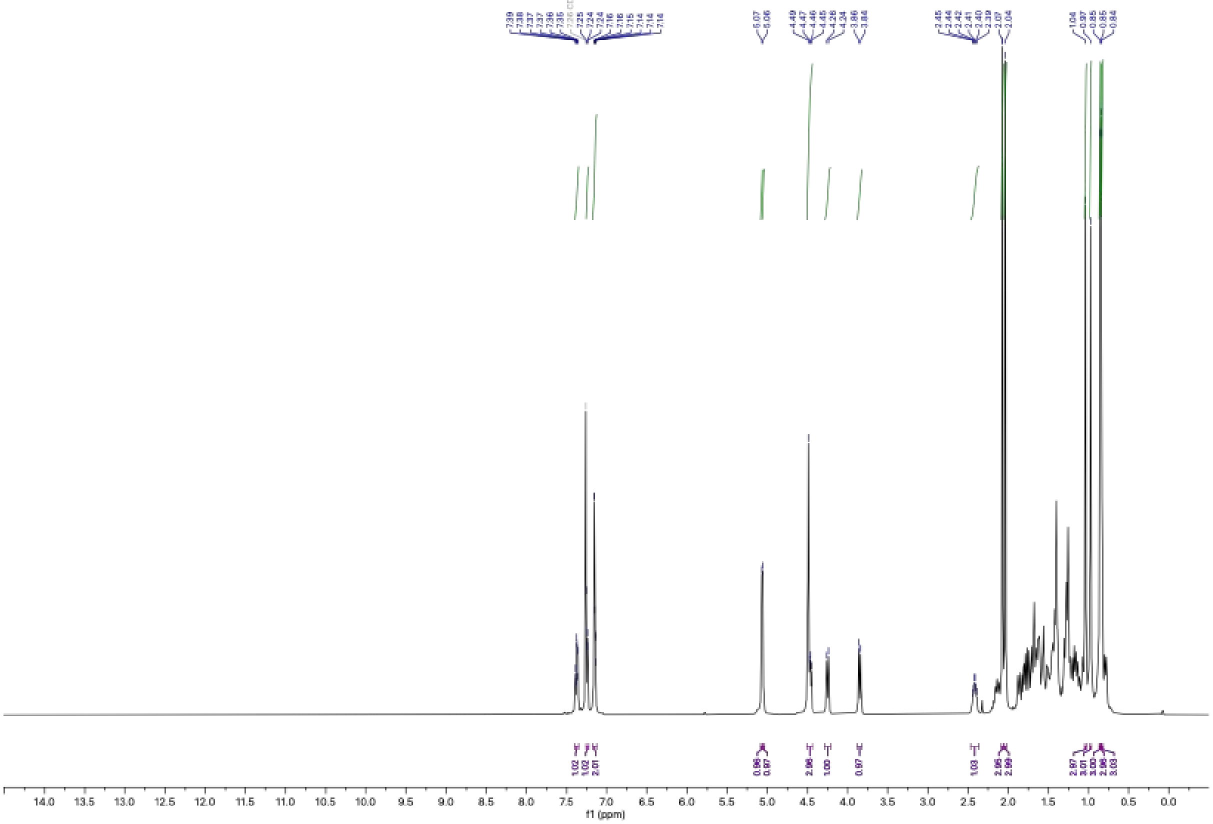

Figure S58. ^{1}H NMR spectrum of the compound **20** ($CDCl_3$, 500 MHz).

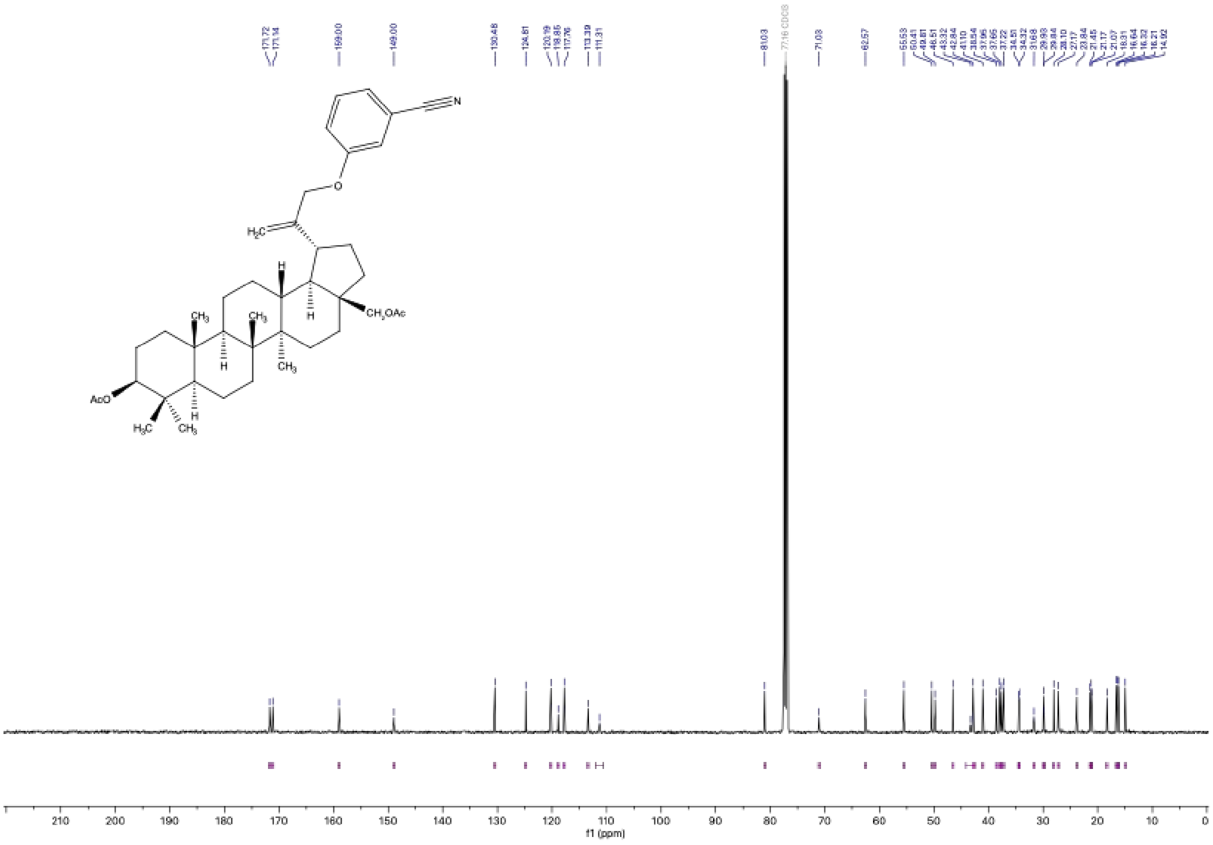

Figure S59. ^{13}C NMR spectrum of the compound **20** ($CDCl_3$, 126 MHz).

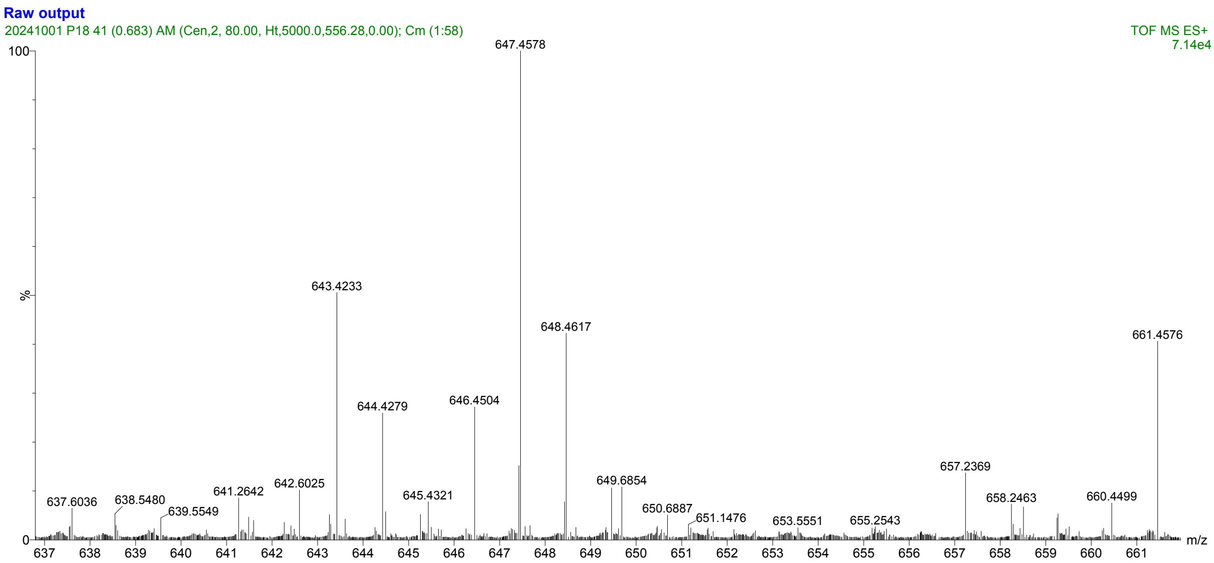

Figure S60. HRMS spectrum of the compound **20**.

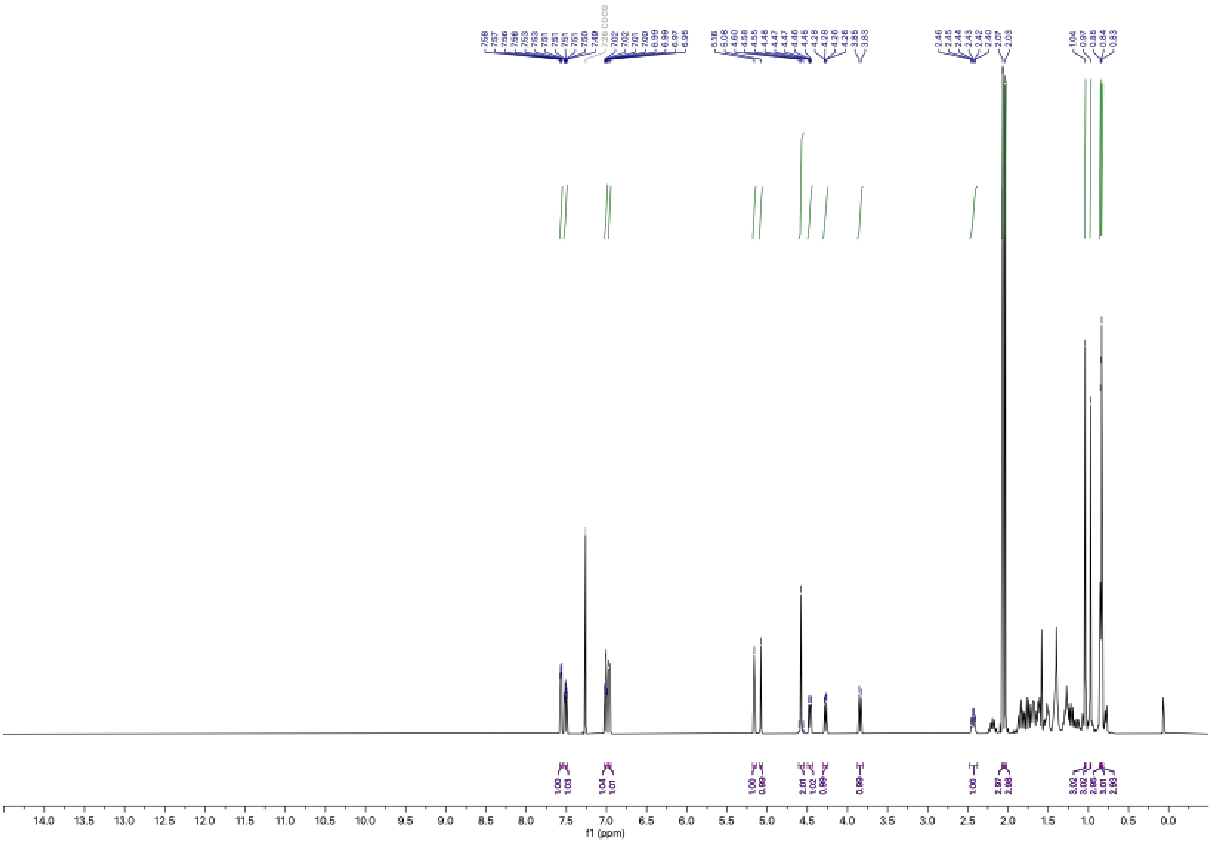

Figure S61. ^{1}H NMR spectrum of the compound **21** ($CDCl_3$, 500 MHz).

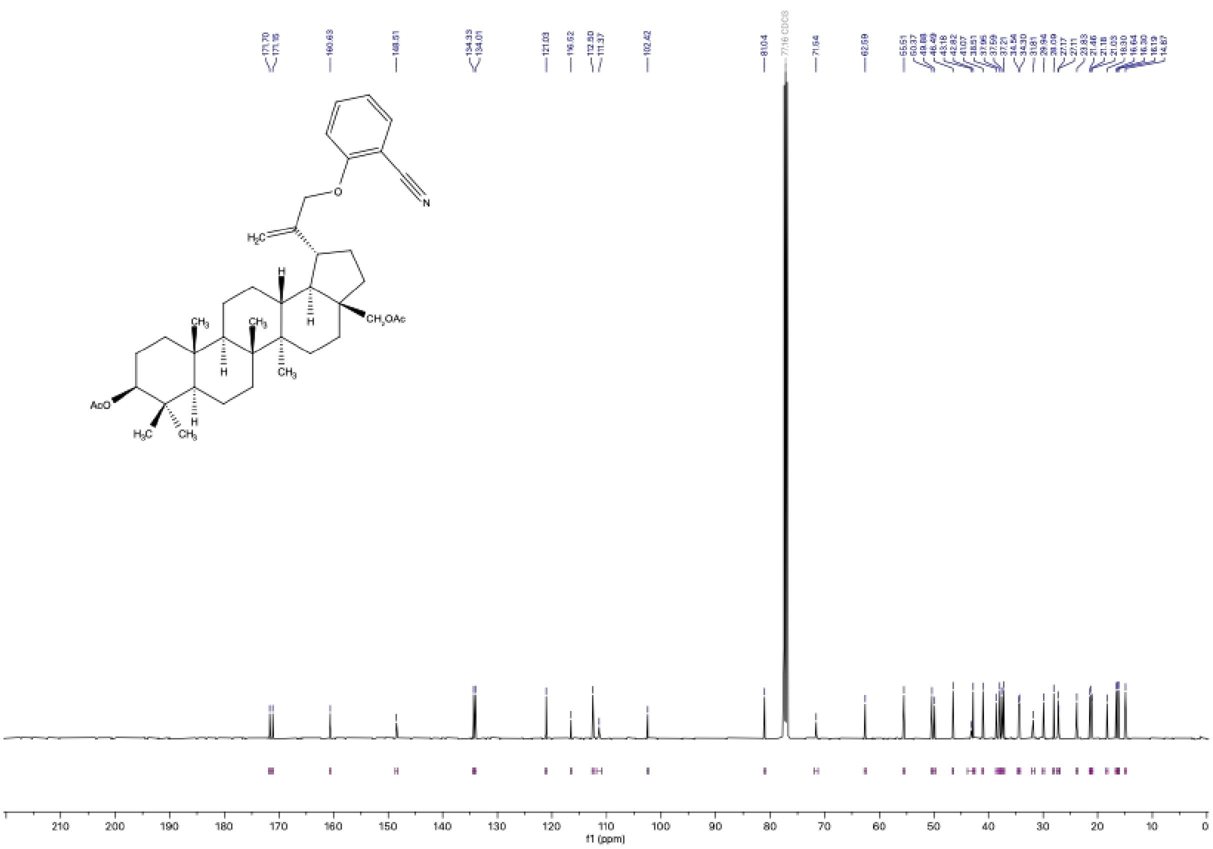

Figure S62. ^{13}C NMR spectrum of the compound **21** ($CDCl_3$, 126 MHz).

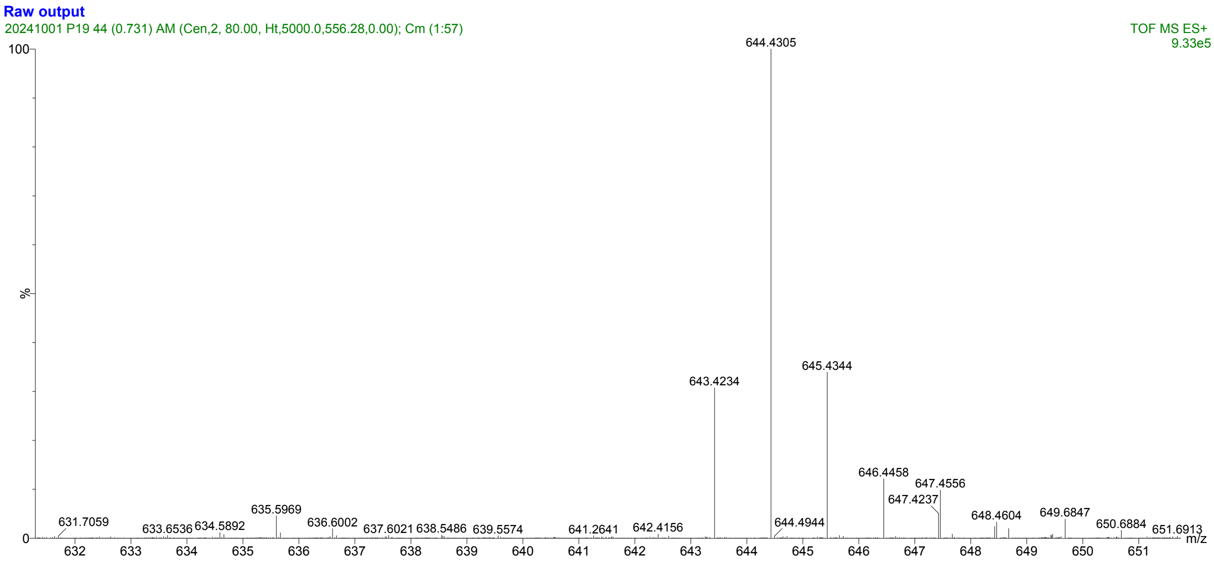

Figure S63. HRMS spectrum of the compound **21**.

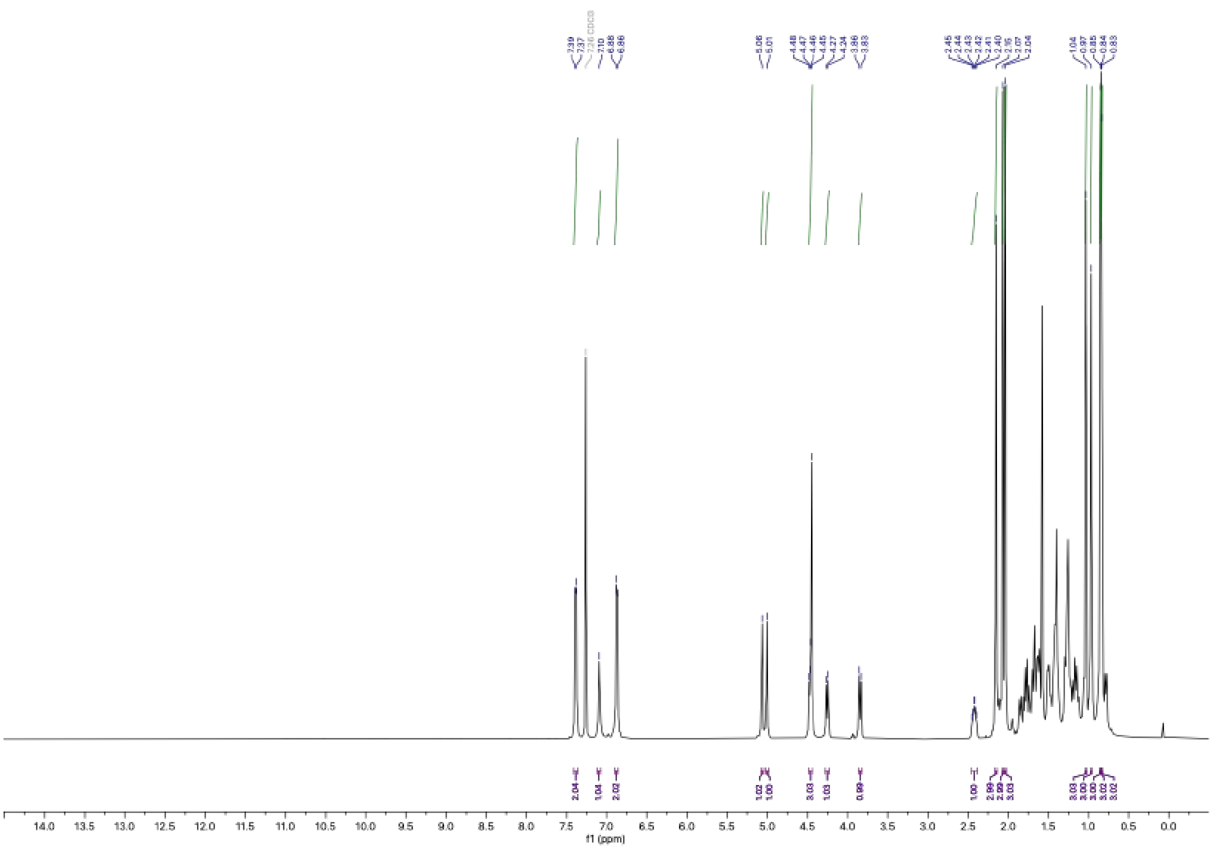

Figure S64. ^{1}H NMR spectrum of the compound **22** ($CDCl_3$, 500 MHz).

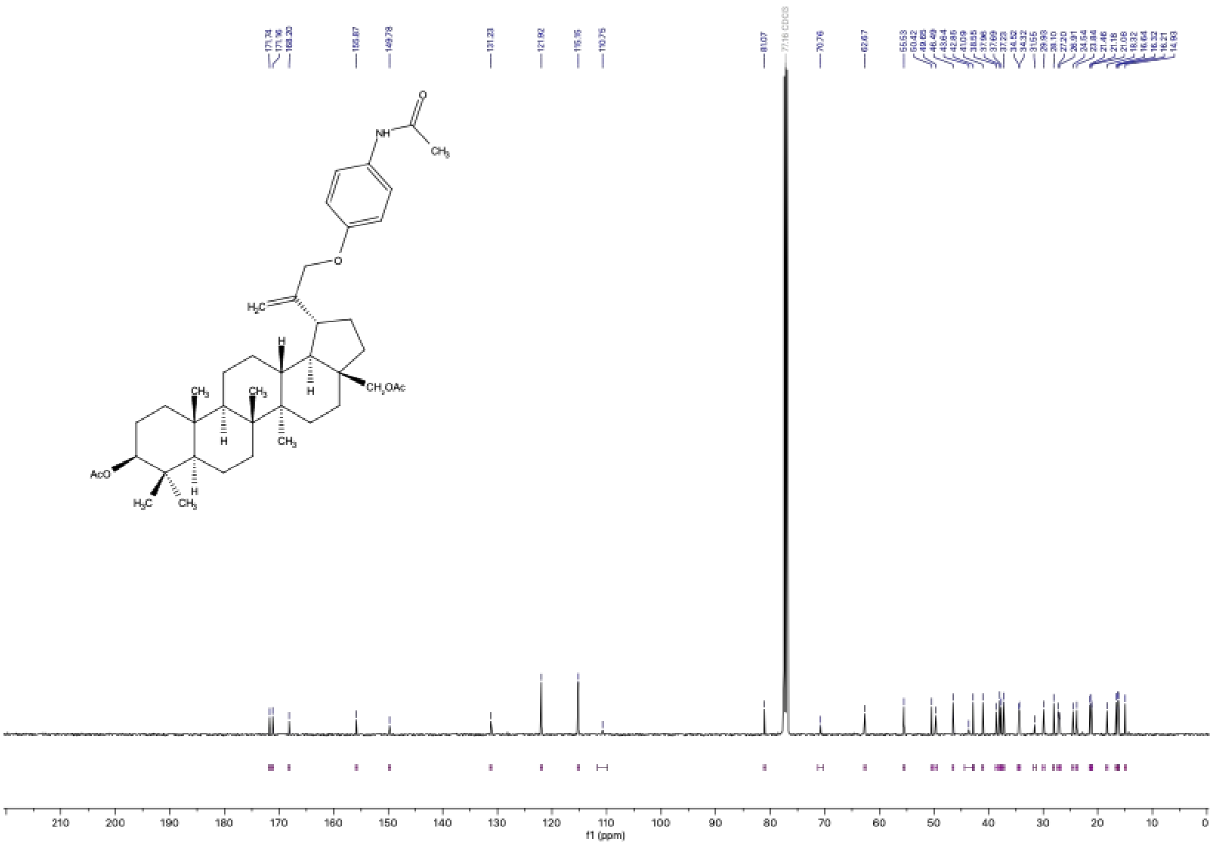

Figure S65. ^{13}C NMR spectrum of the compound **22** ($CDCl_3$, 126 MHz).

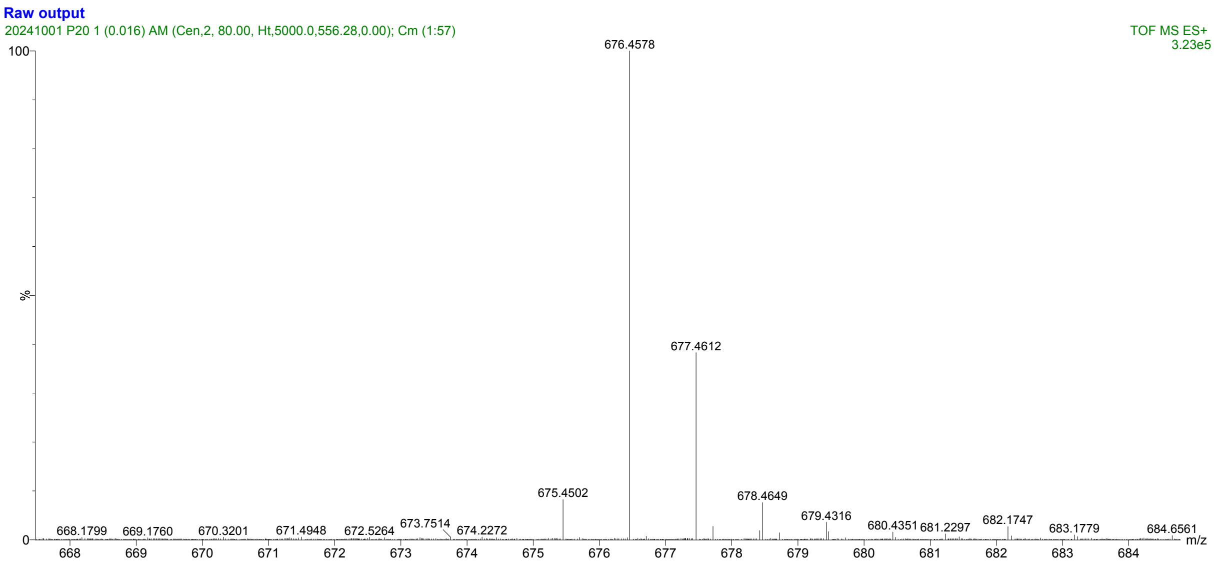

Figure S66. HRMS spectrum of the compound **22**.

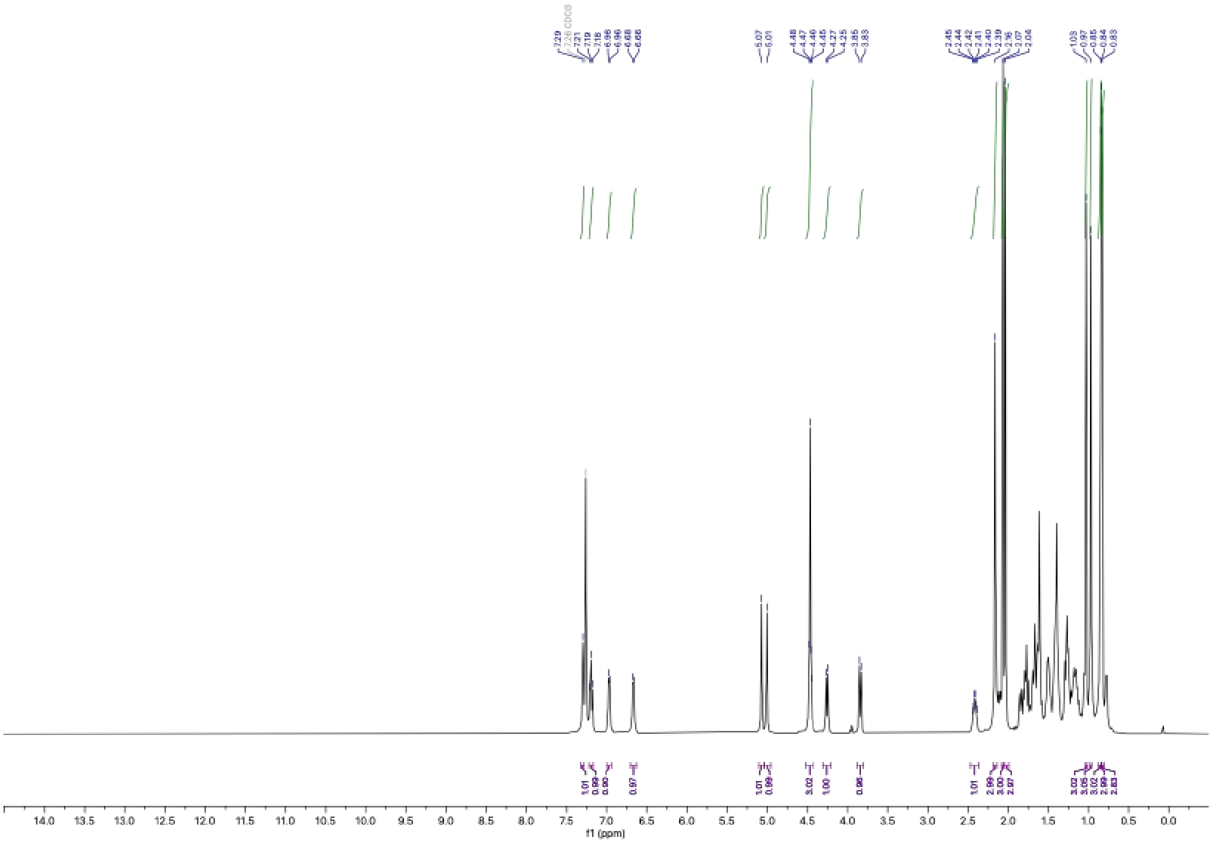

Figure S67. ^{1}H NMR spectrum of the compound **23** ($CDCl_3$, 500 MHz).

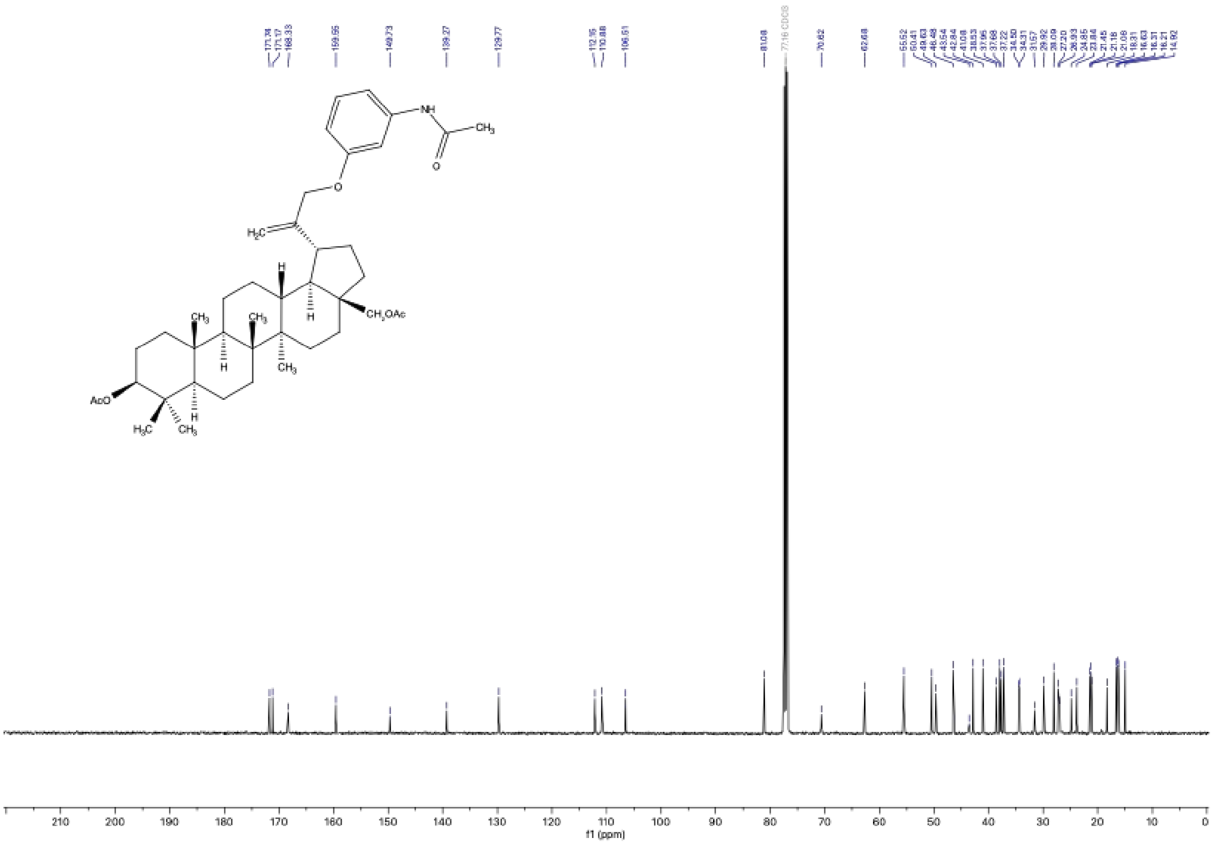

Figure S68. ^{13}C NMR spectrum of the compound **23** ($CDCl_3$, 126 MHz).

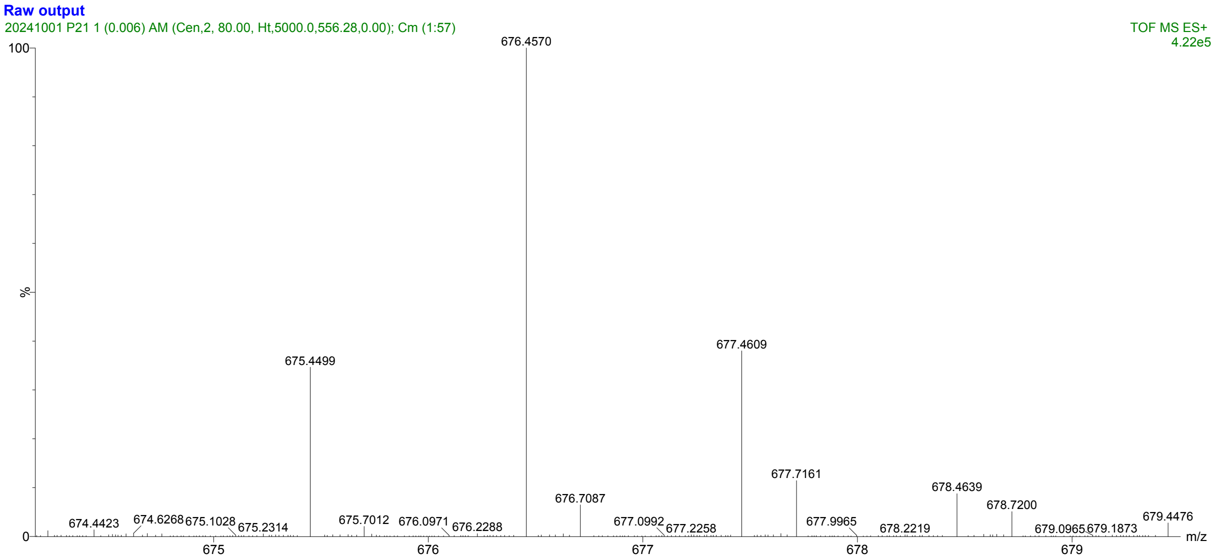

Figure S69. HRMS spectrum of the compound **23**.

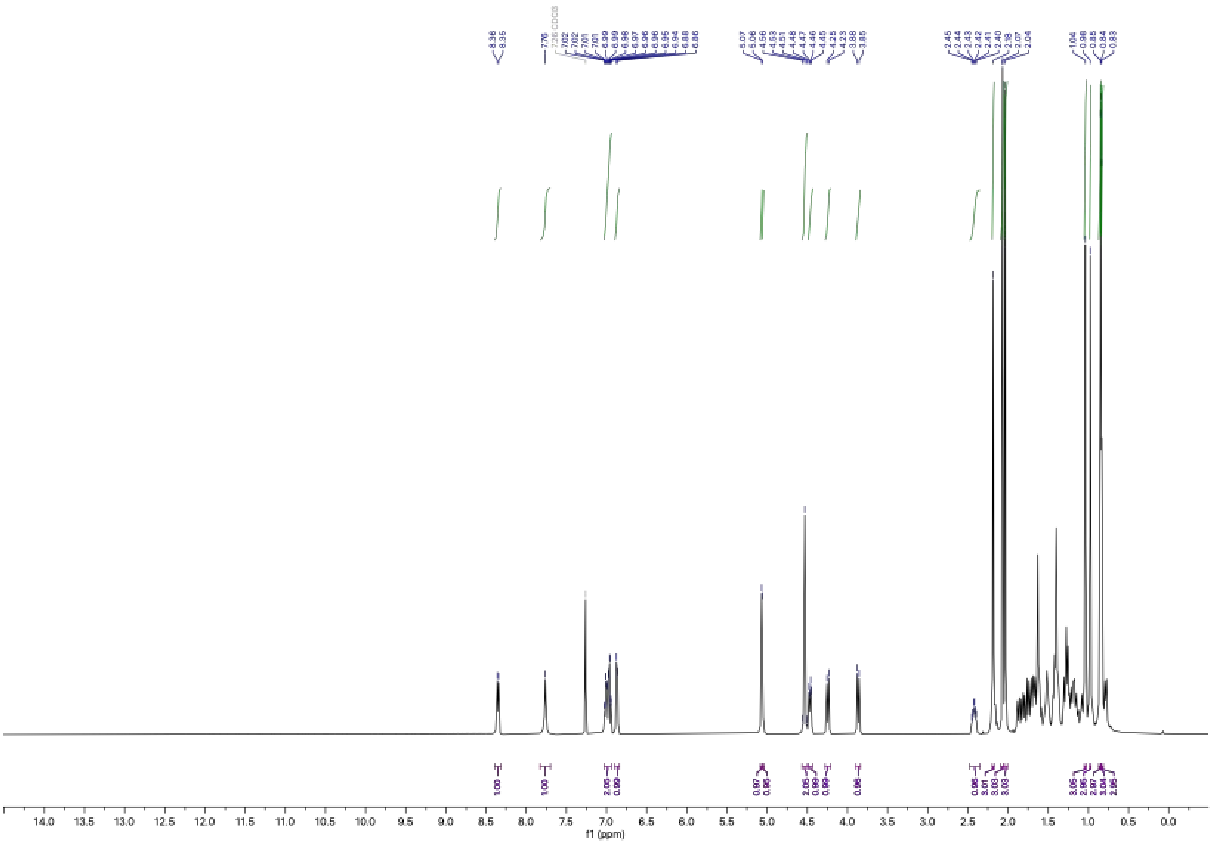

Figure S70. ^{1}H NMR spectrum of the compound **24** ($CDCl_3$, 500 MHz).

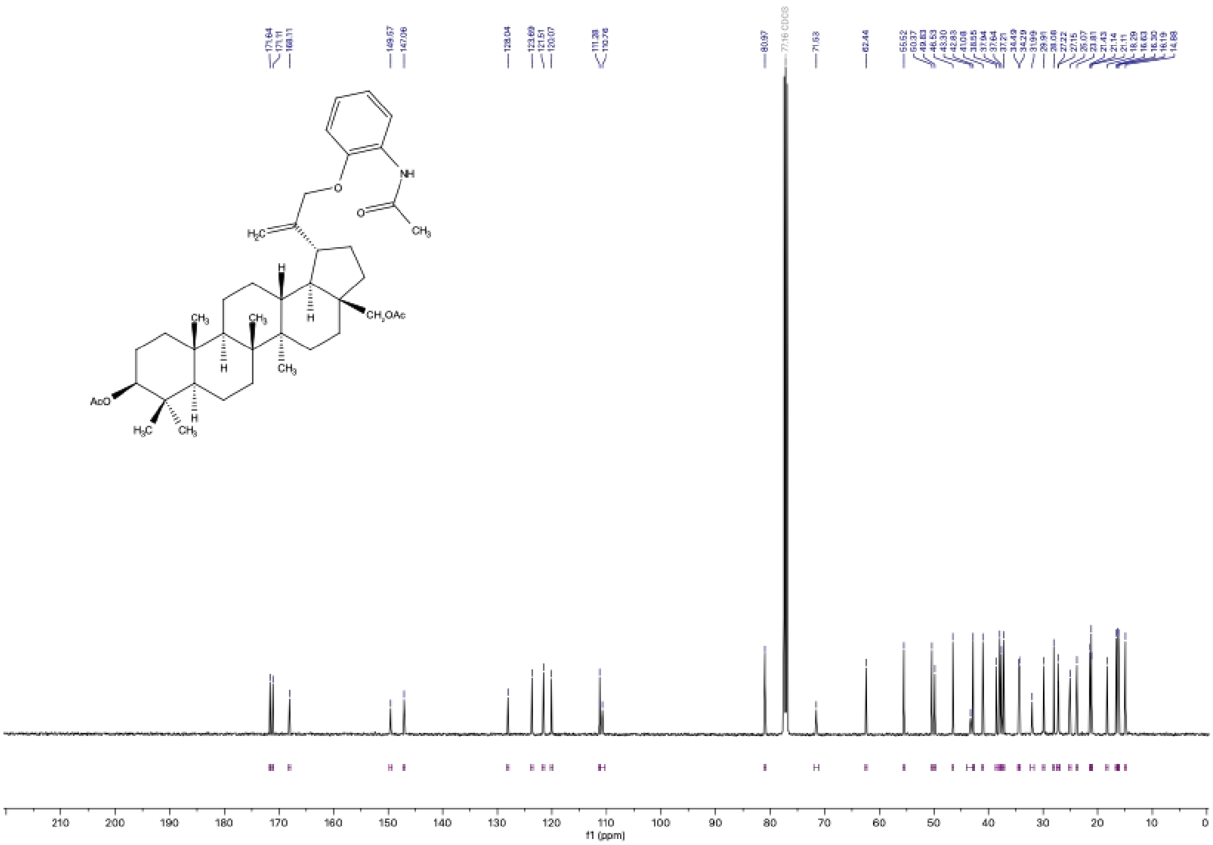

Figure S71. ^{13}C NMR spectrum of the compound **24** ($CDCl_3$, 126 MHz).

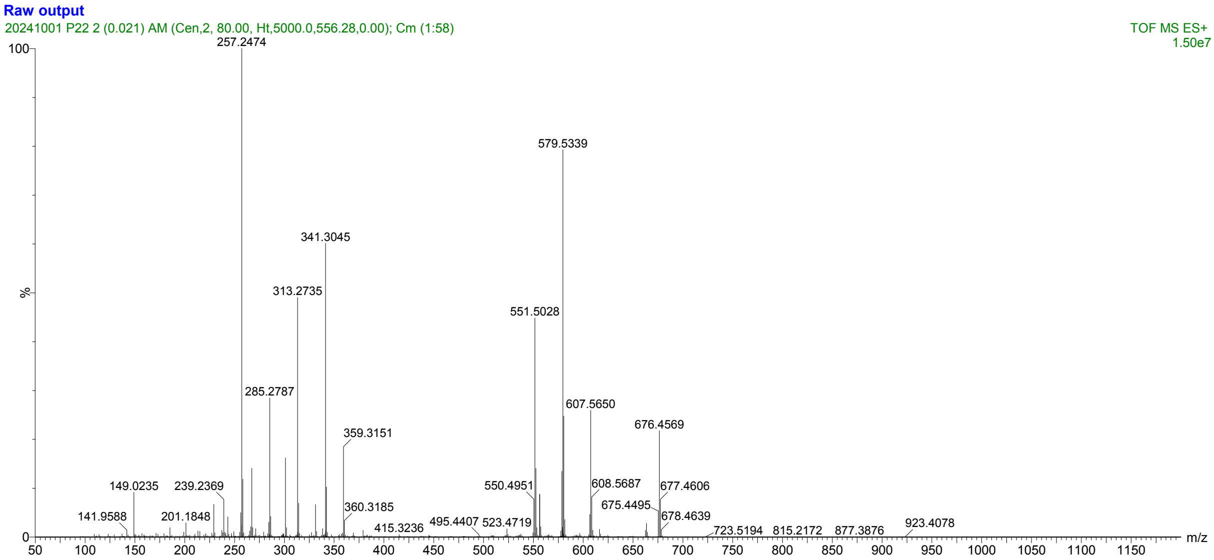

Figure S72. HRMS spectrum of the compound **24**.

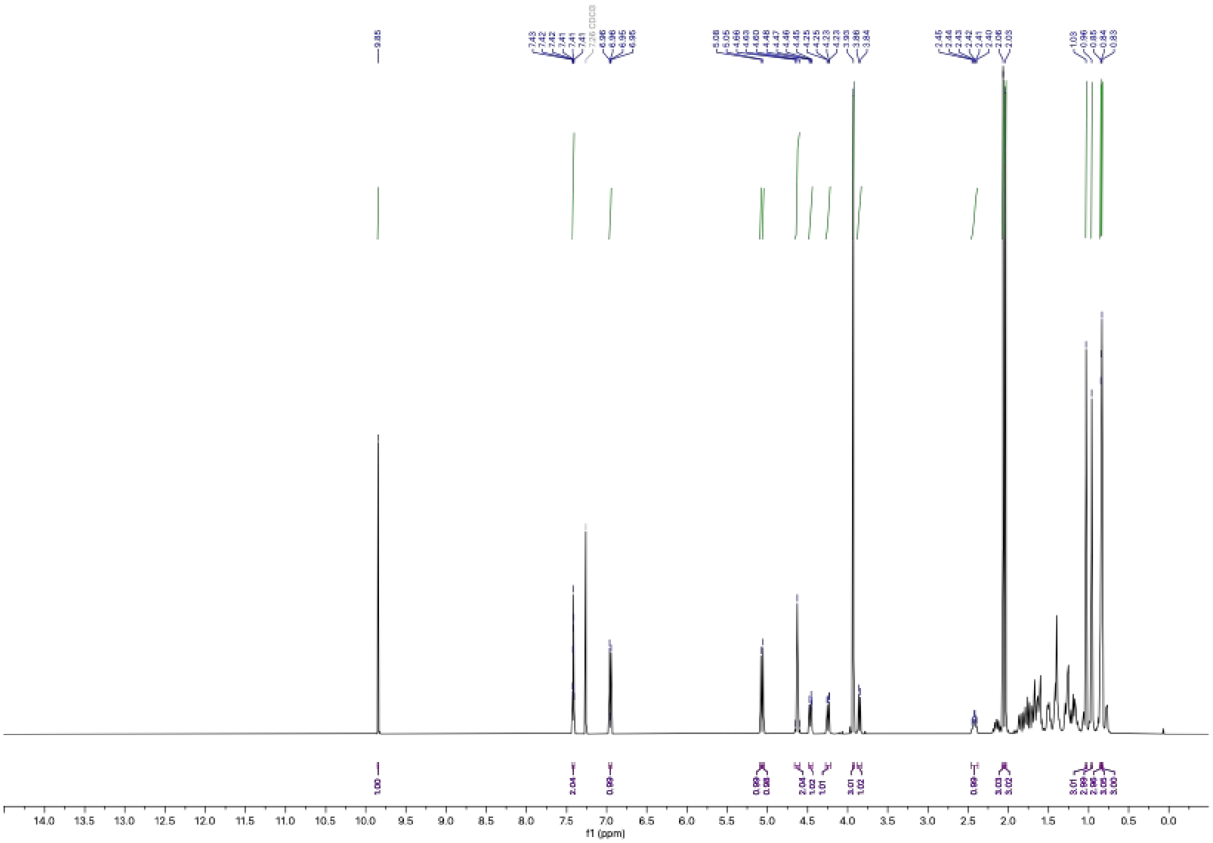

Figure S73. ^{1}H NMR spectrum of the compound **25** ($CDCl_3$, 500 MHz).

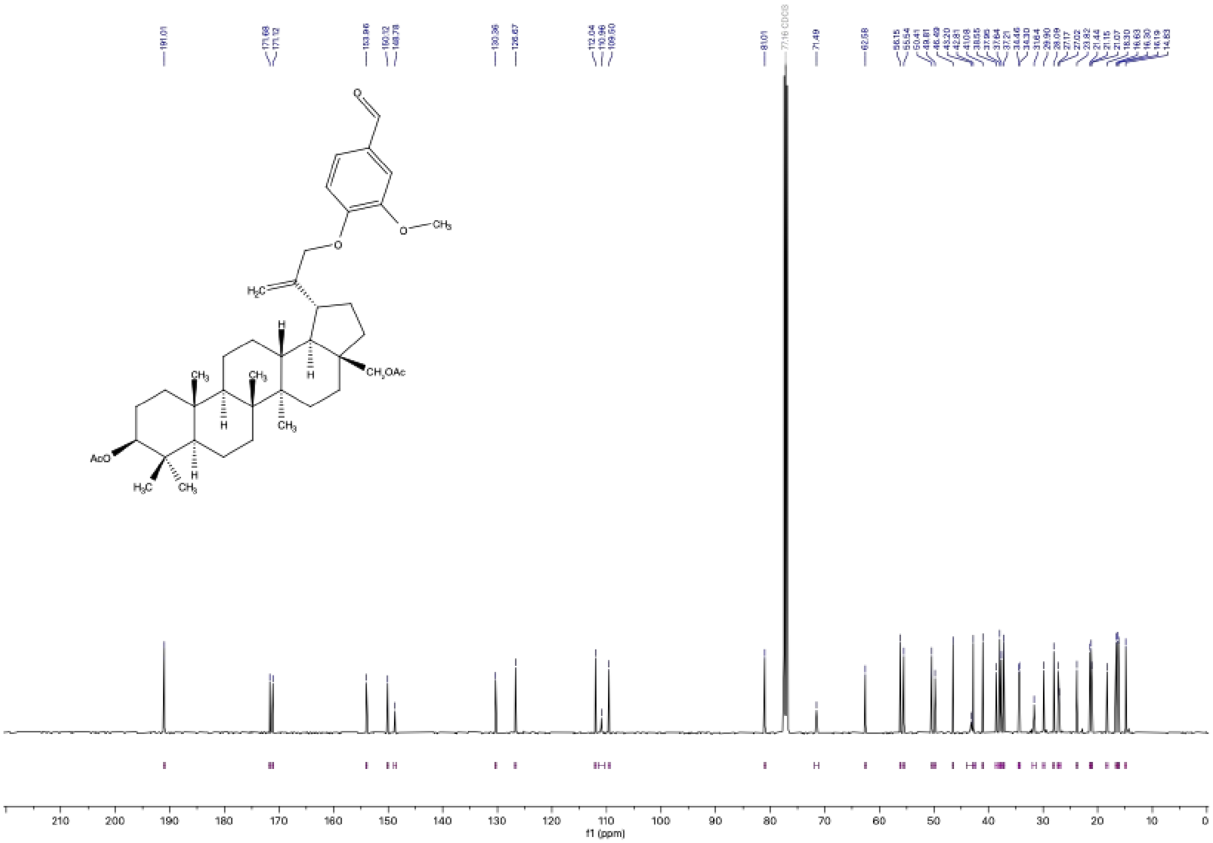

Figure S74. ^{13}C NMR spectrum of the compound **25** ($CDCl_3$, 126 MHz).

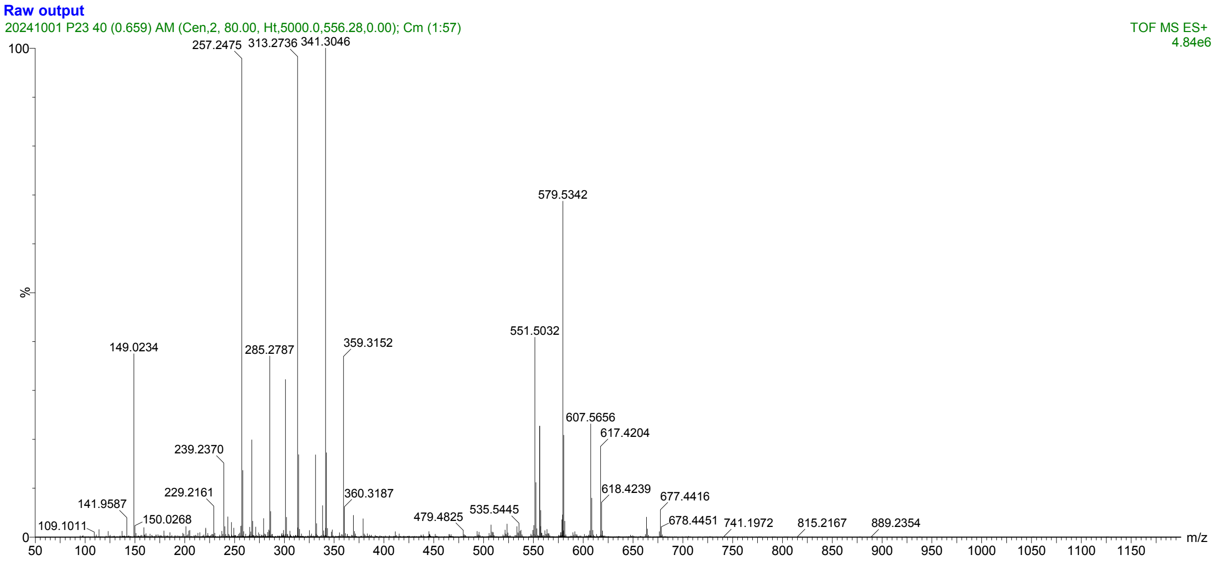

Figure S75. HRMS spectrum of the compound **25**.

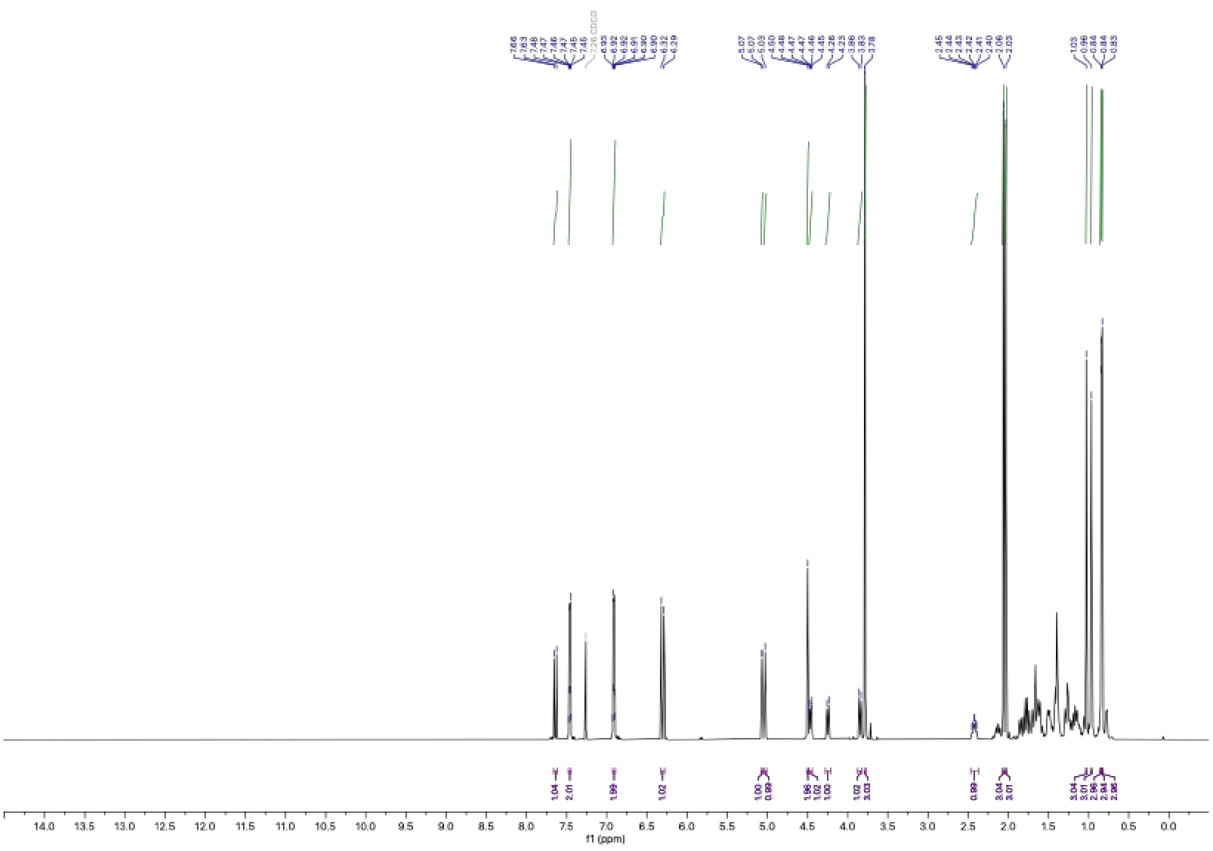

Figure S76. ^{1}H NMR spectrum of the compound **26** ($CDCl_3$, 500 MHz).

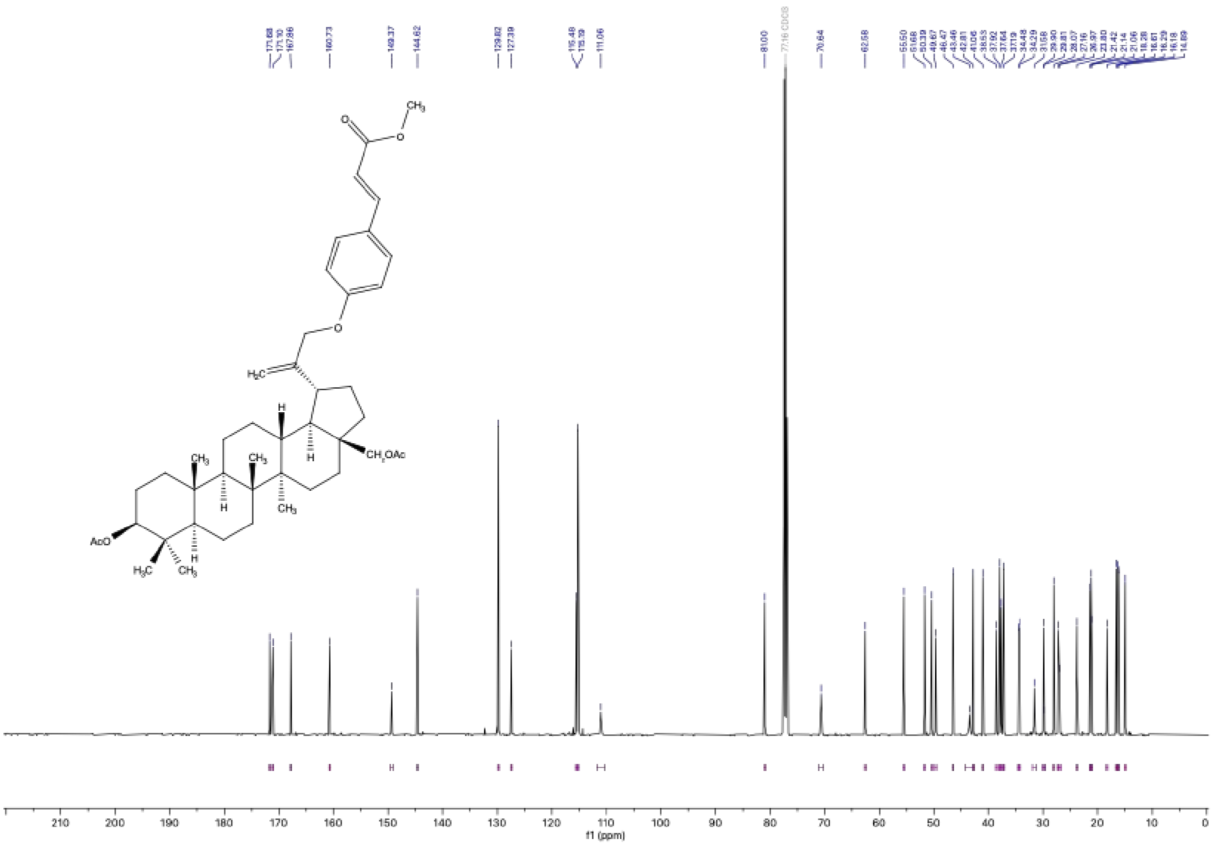

Figure S77. ^{13}C NMR spectrum of the compound **26** ($CDCl_3$, 126 MHz).

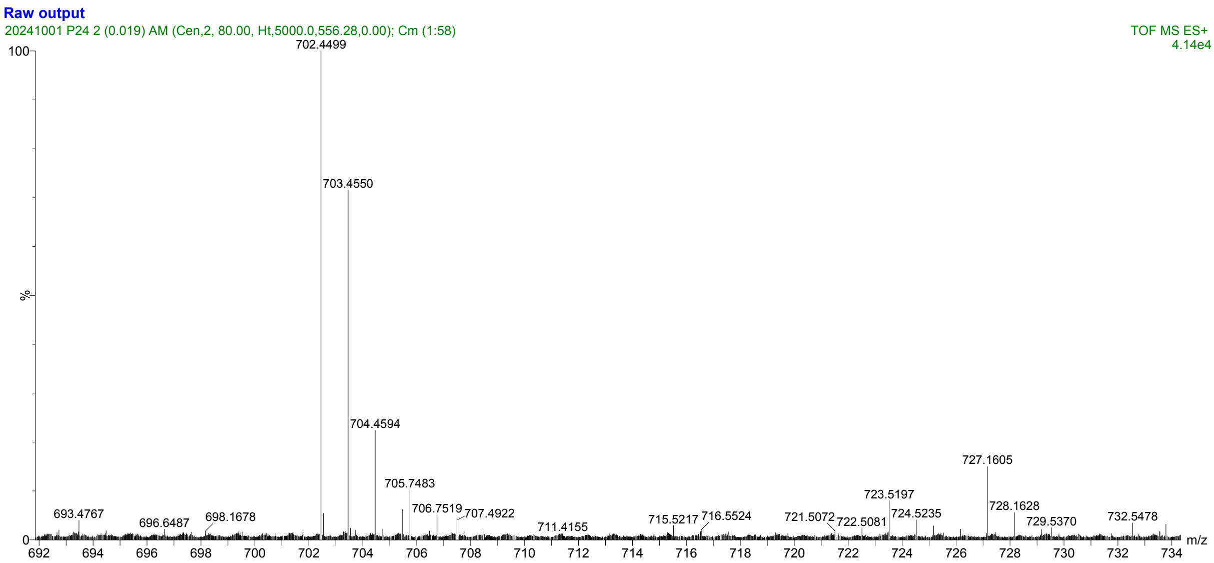

Figure S78. HRMS spectrum of the compound **26**.

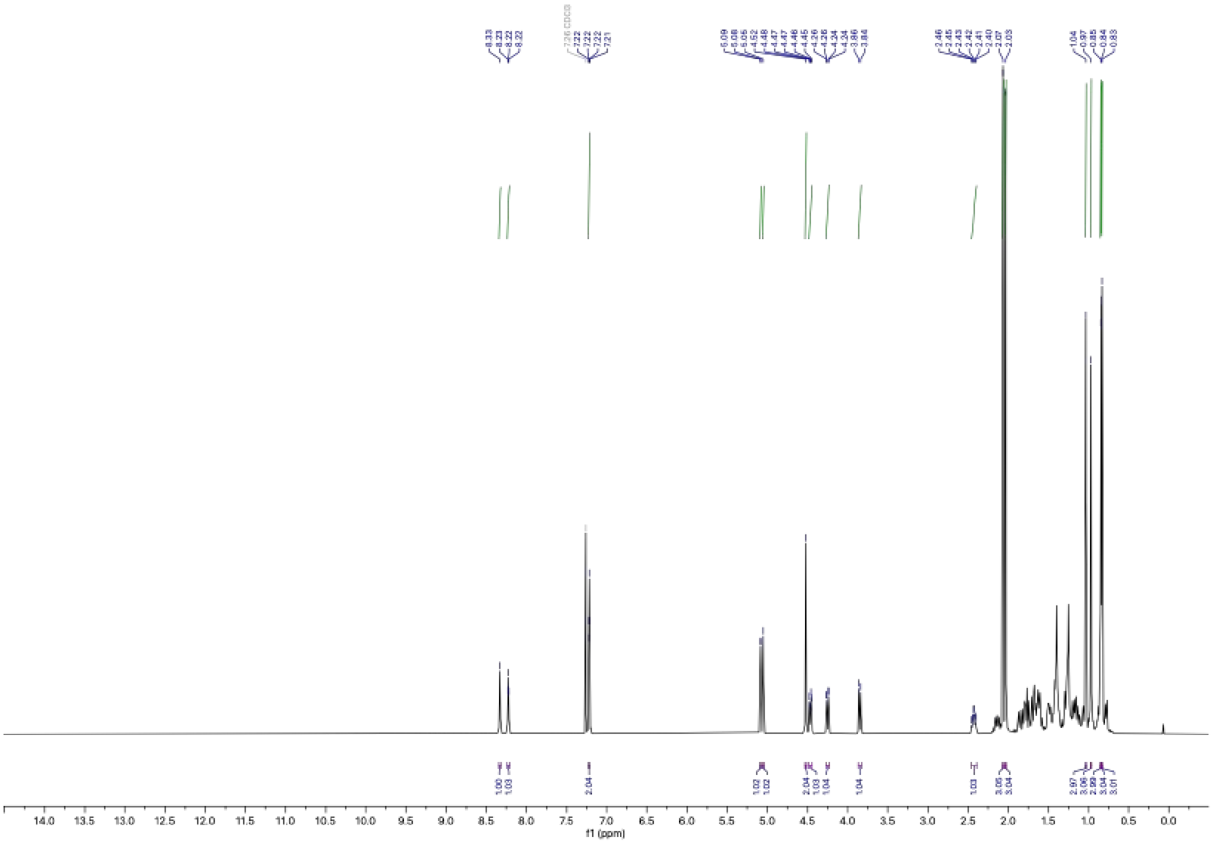

Figure S79. ^{1}H NMR spectrum of the compound **27** ($CDCl_3$, 500 MHz).

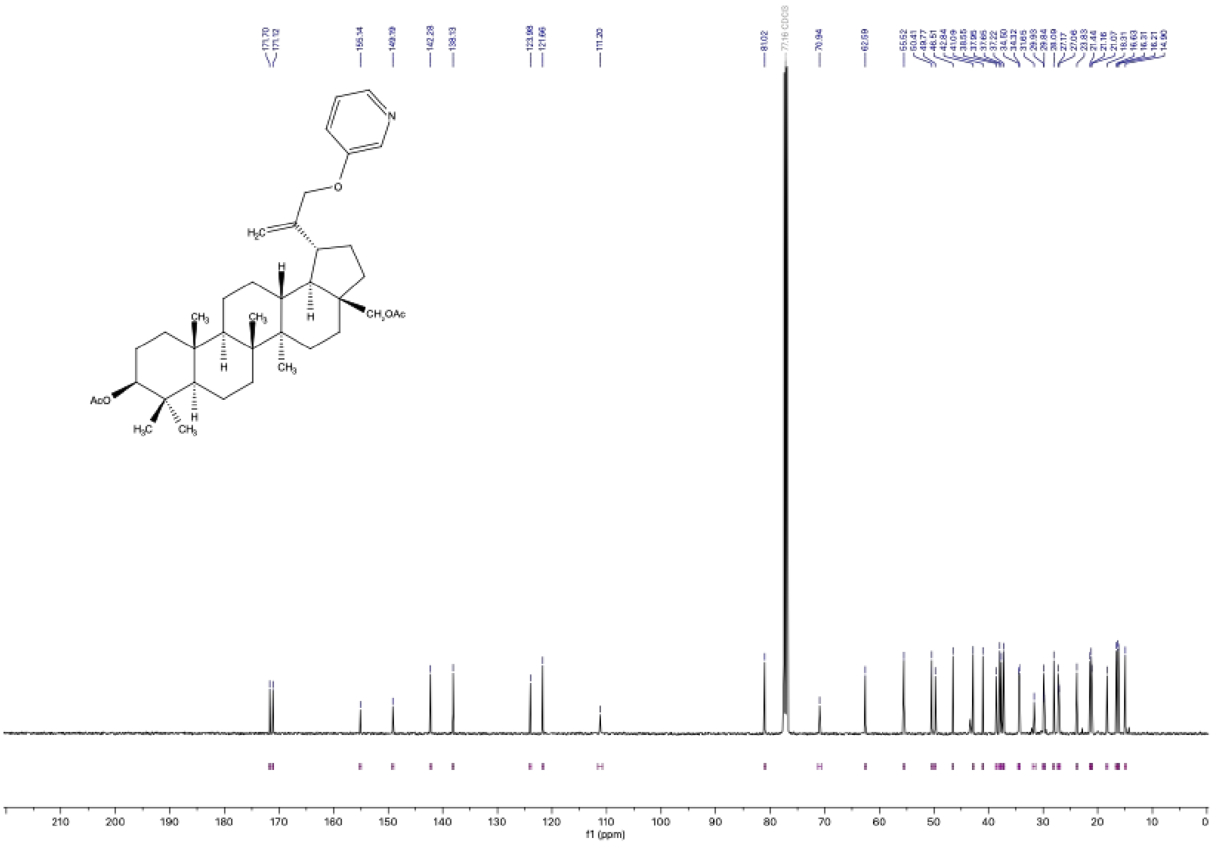

Figure S80. ^{13}C NMR spectrum of the compound **27** ($CDCl_3$, 126 MHz).

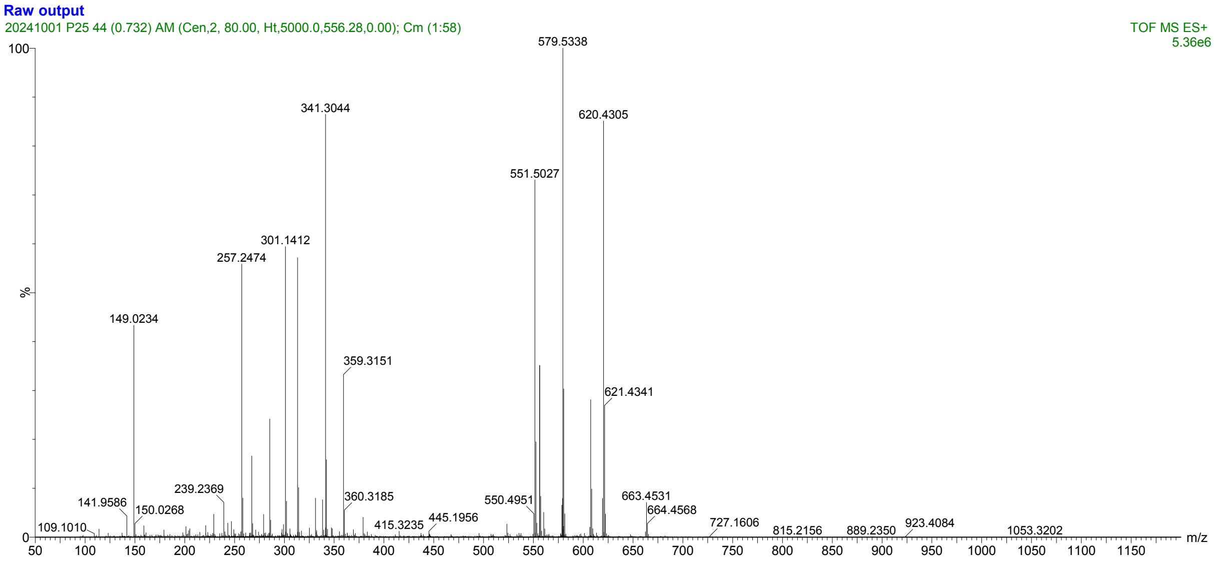

Figure S81. HRMS spectrum of the compound **27**.

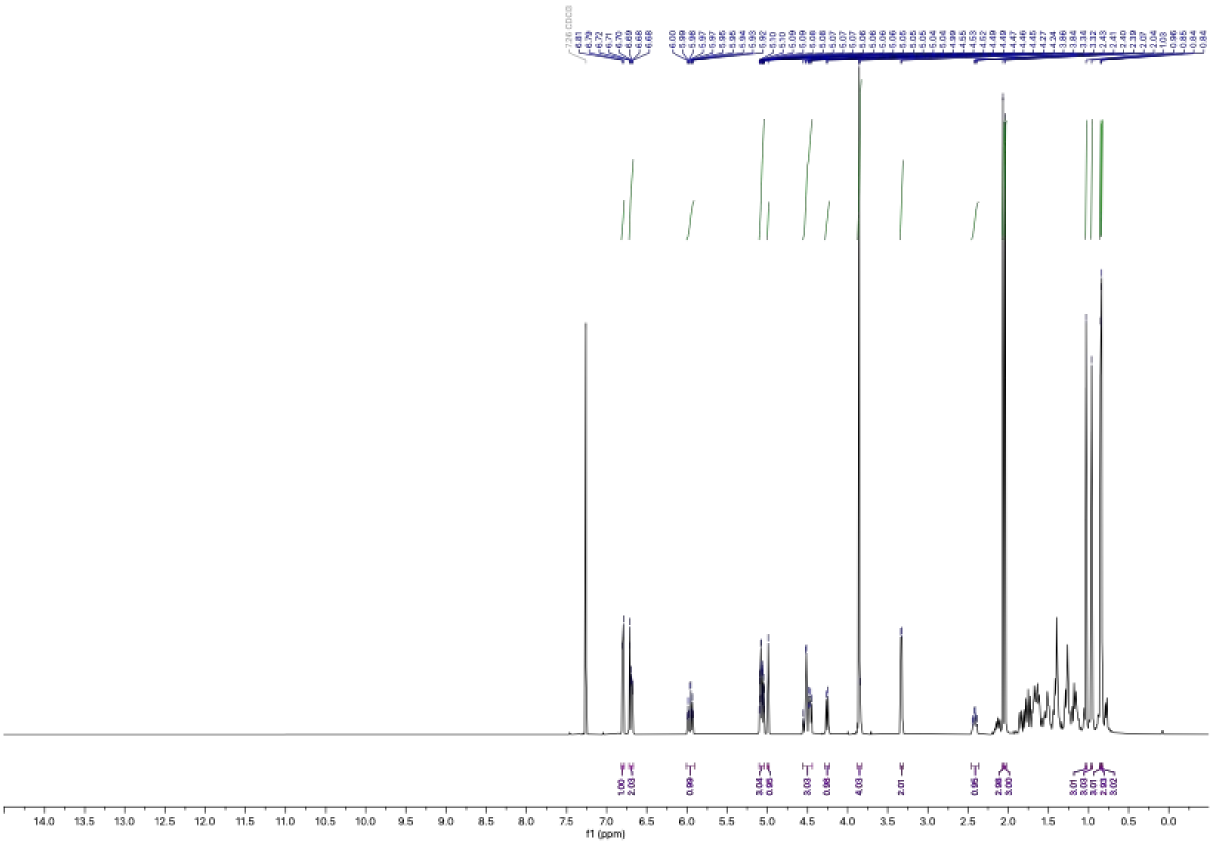

Figure S82. ^{1}H NMR spectrum of the compound **28** ($CDCl_3$, 500 MHz).

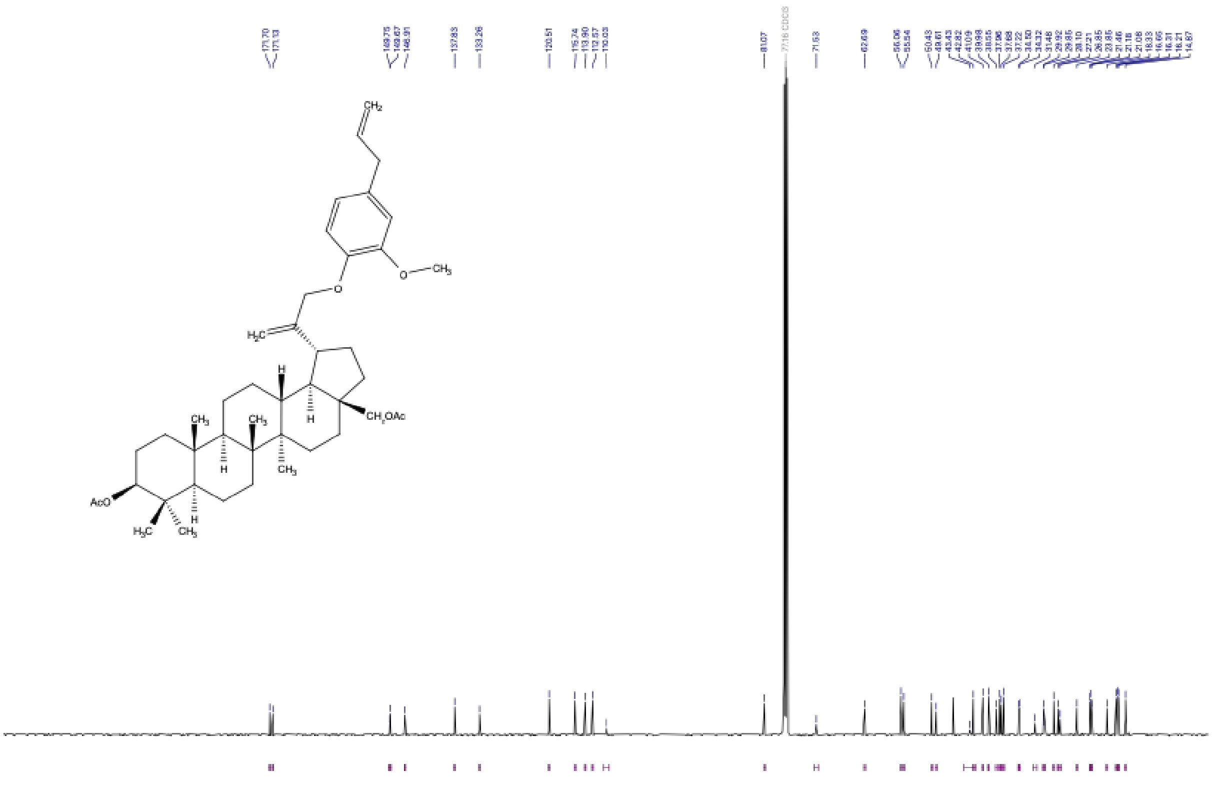

Figure S83. ^{13}C NMR spectrum of the compound **28** ($CDCl_3$, 126 MHz).

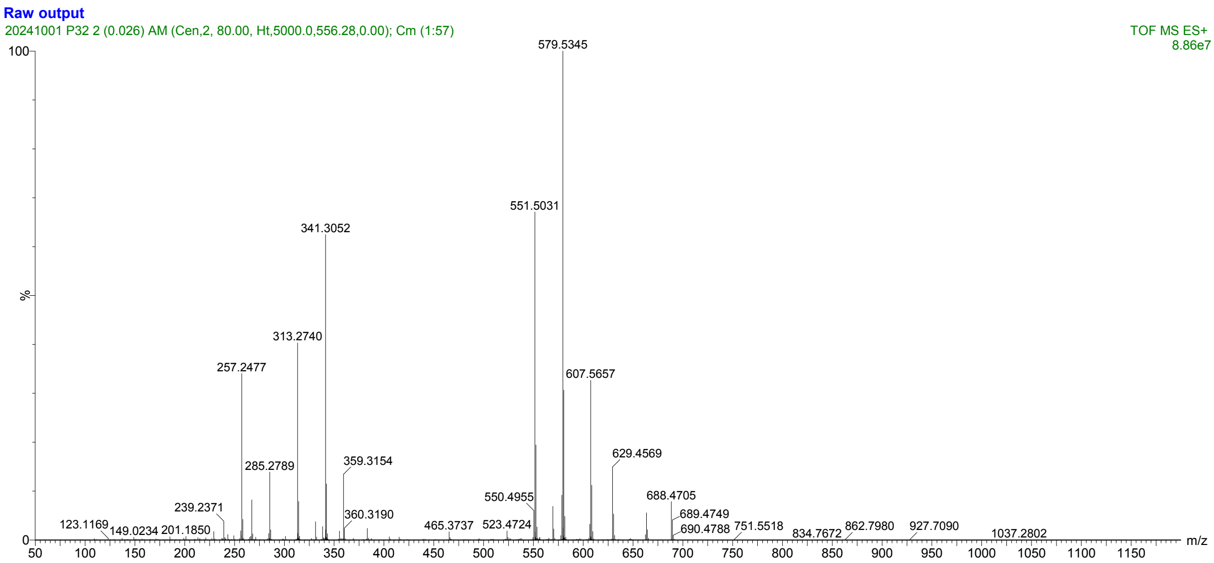

Figure S84. HRMS spectrum of the compound **28**.

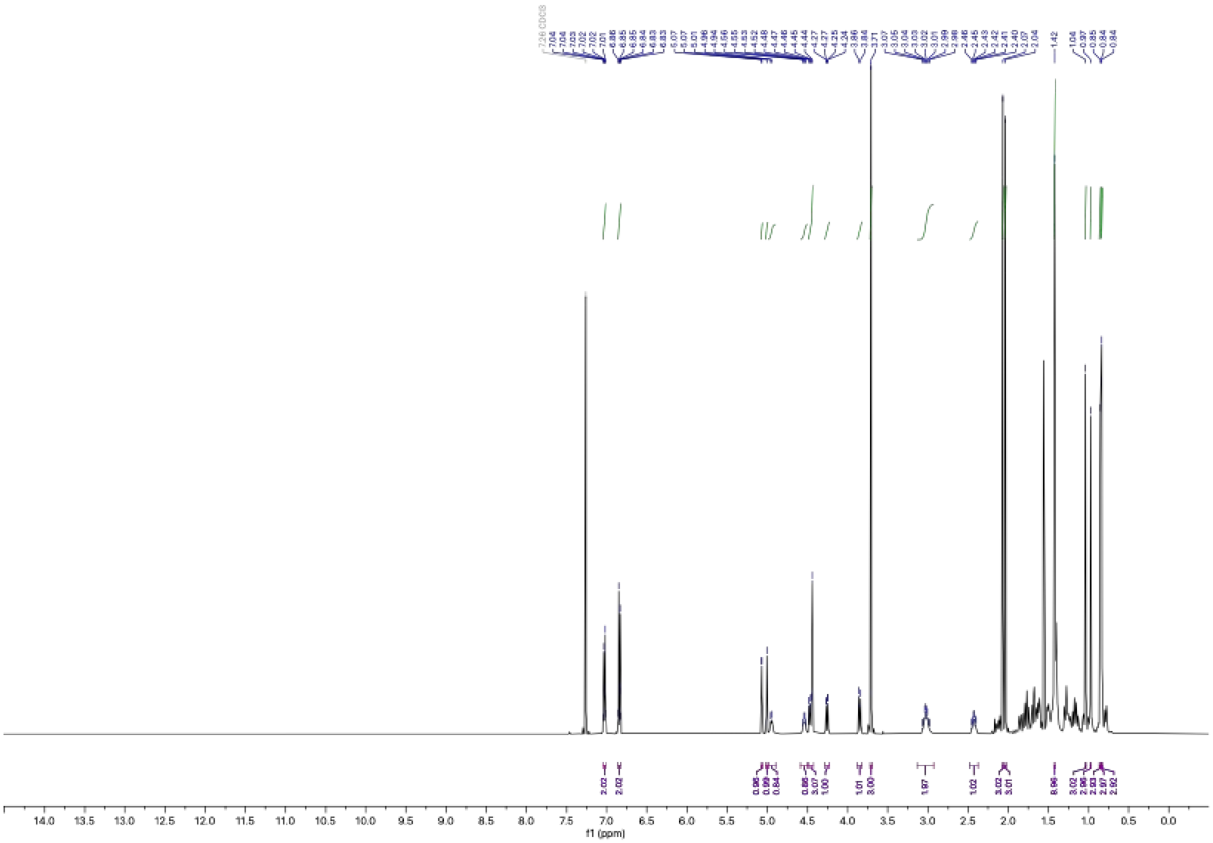

Figure S85. ^{1}H NMR spectrum of the compound **29** ($CDCl_3$, 500 MHz).

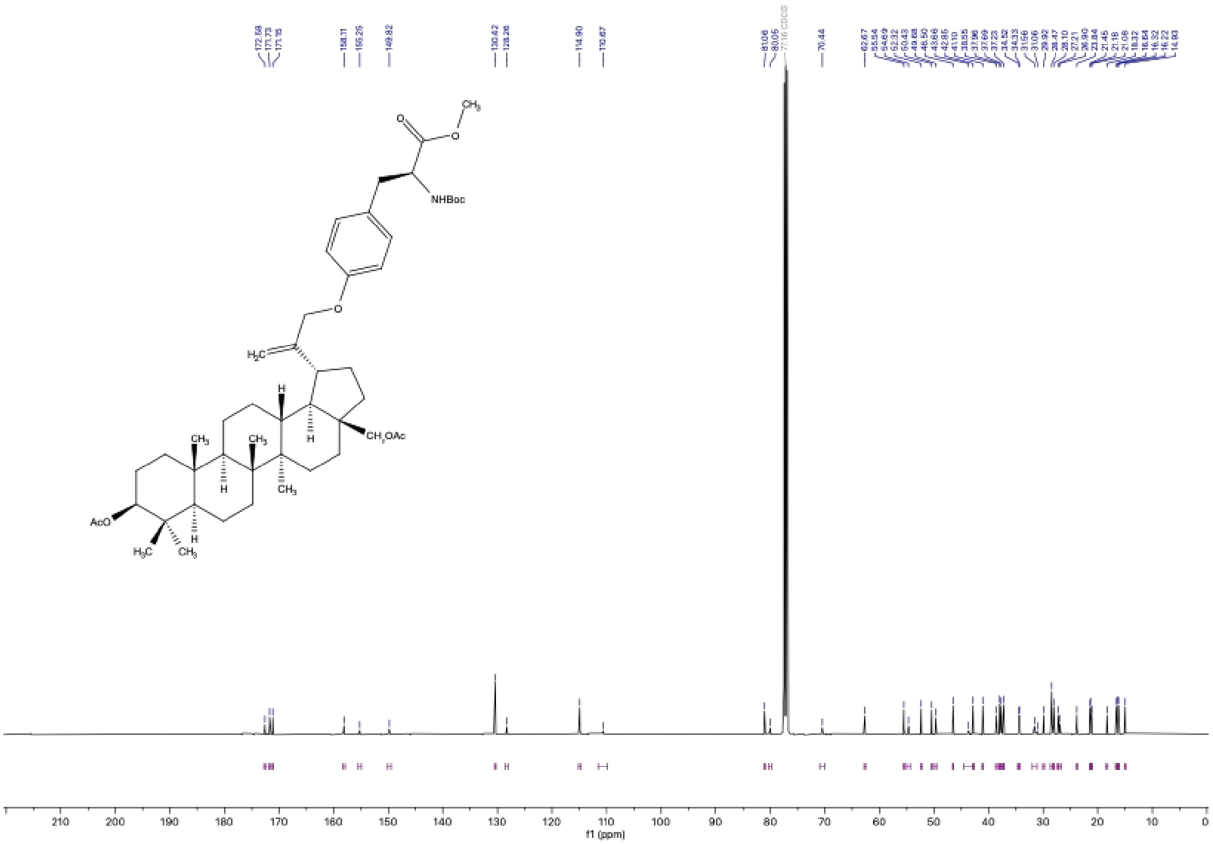

Figure S86. ^{13}C NMR spectrum of the compound **29** ($CDCl_3$, 126 MHz).

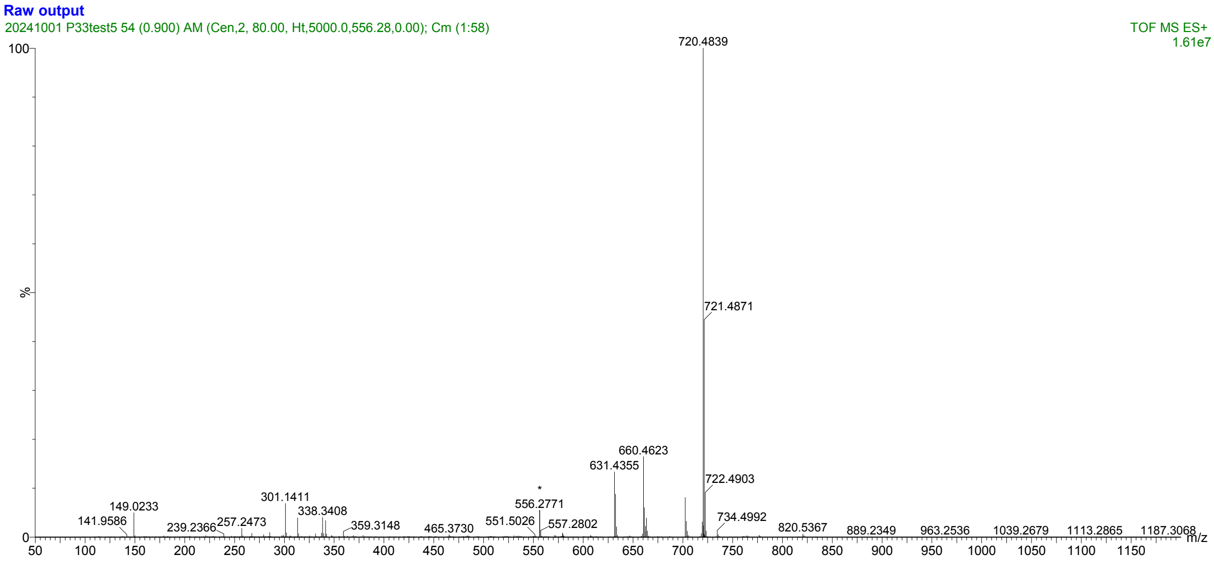

Figure S87. HRMS spectrum of the compound **29**.

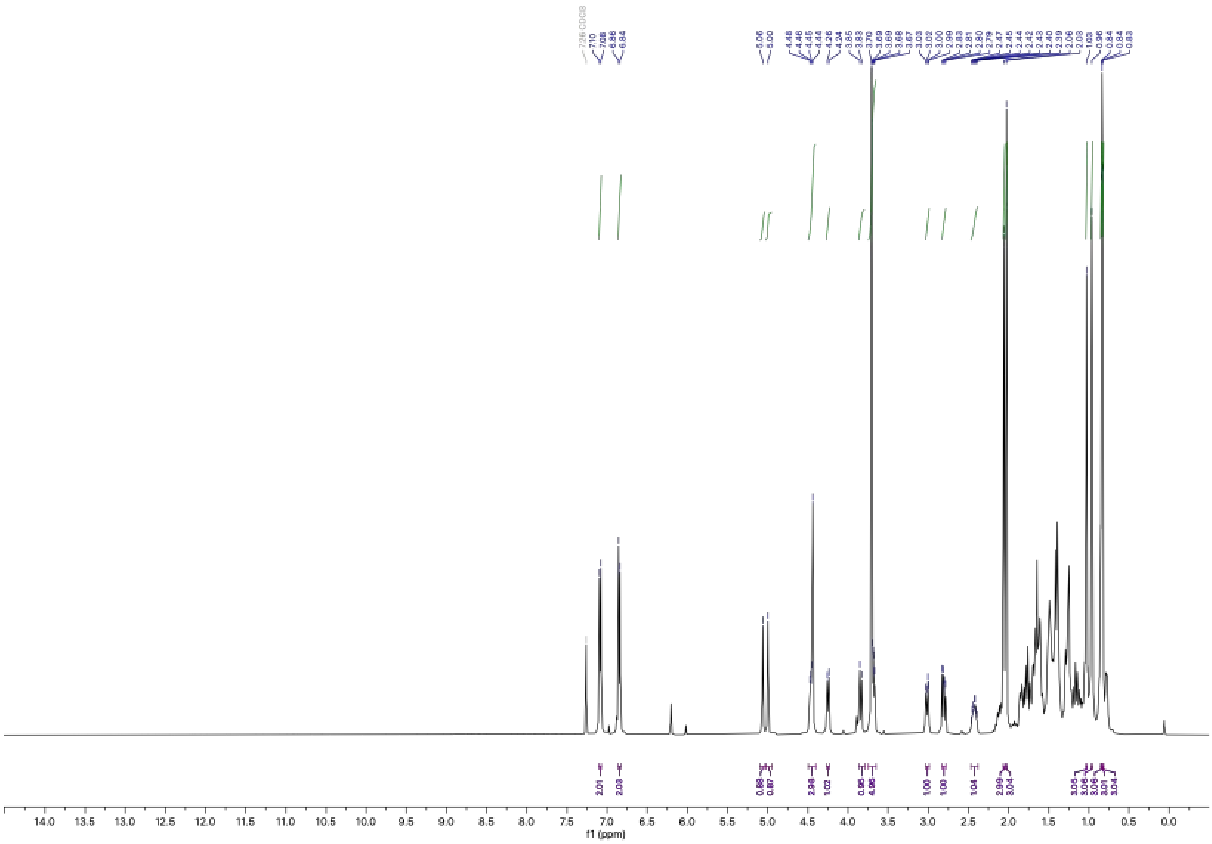

Figure S88. ^{1}H NMR spectrum of the compound **30** ($CDCl_3$, 500 MHz).

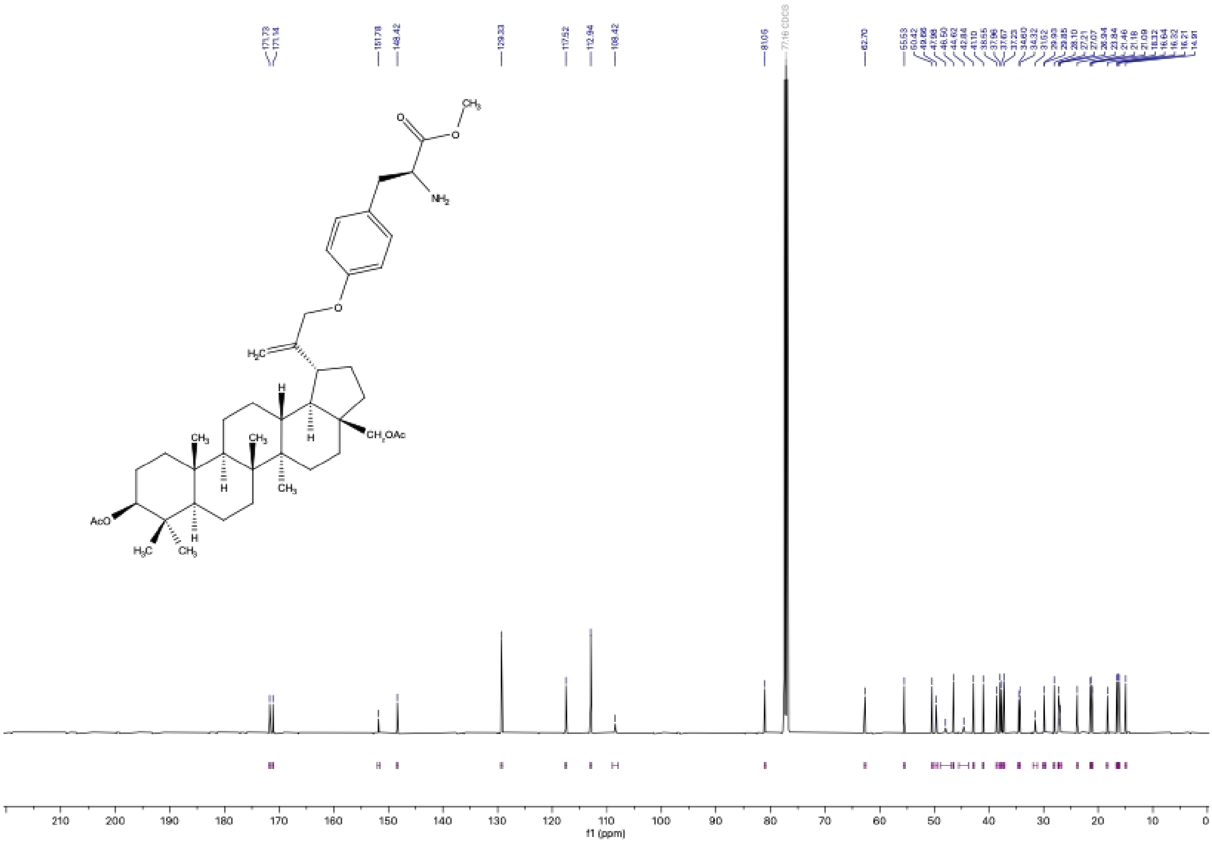

Figure S89. ^{13}C NMR spectrum of the compound **30** ($CDCl_3$, 126 MHz).

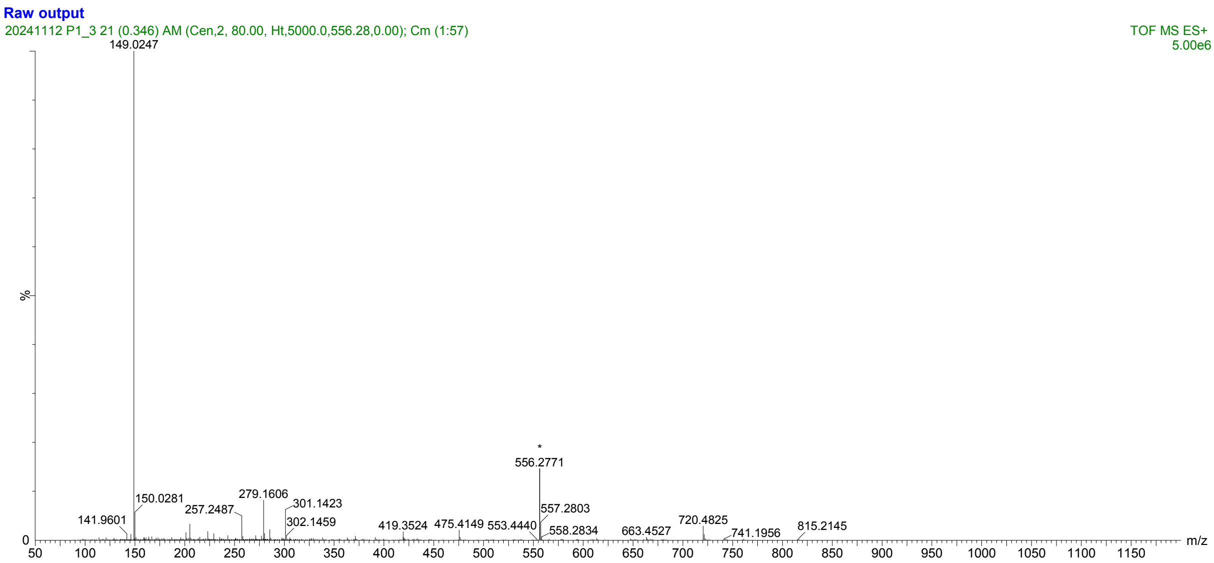

Figure S90. HRMS spectrum of the compound **30**.

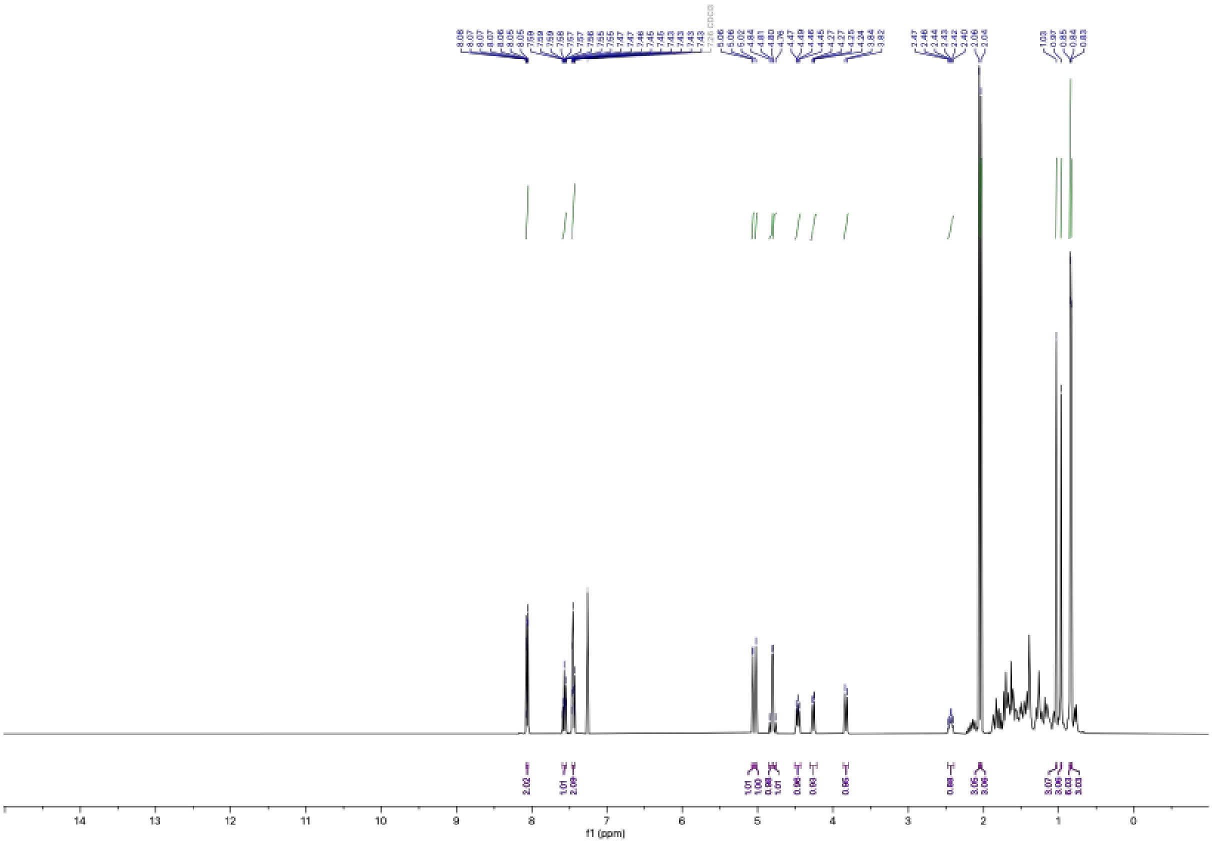

Figure S91. ^{1}H NMR spectrum of the compound **31** ($CDCl_3$, 400 MHz).

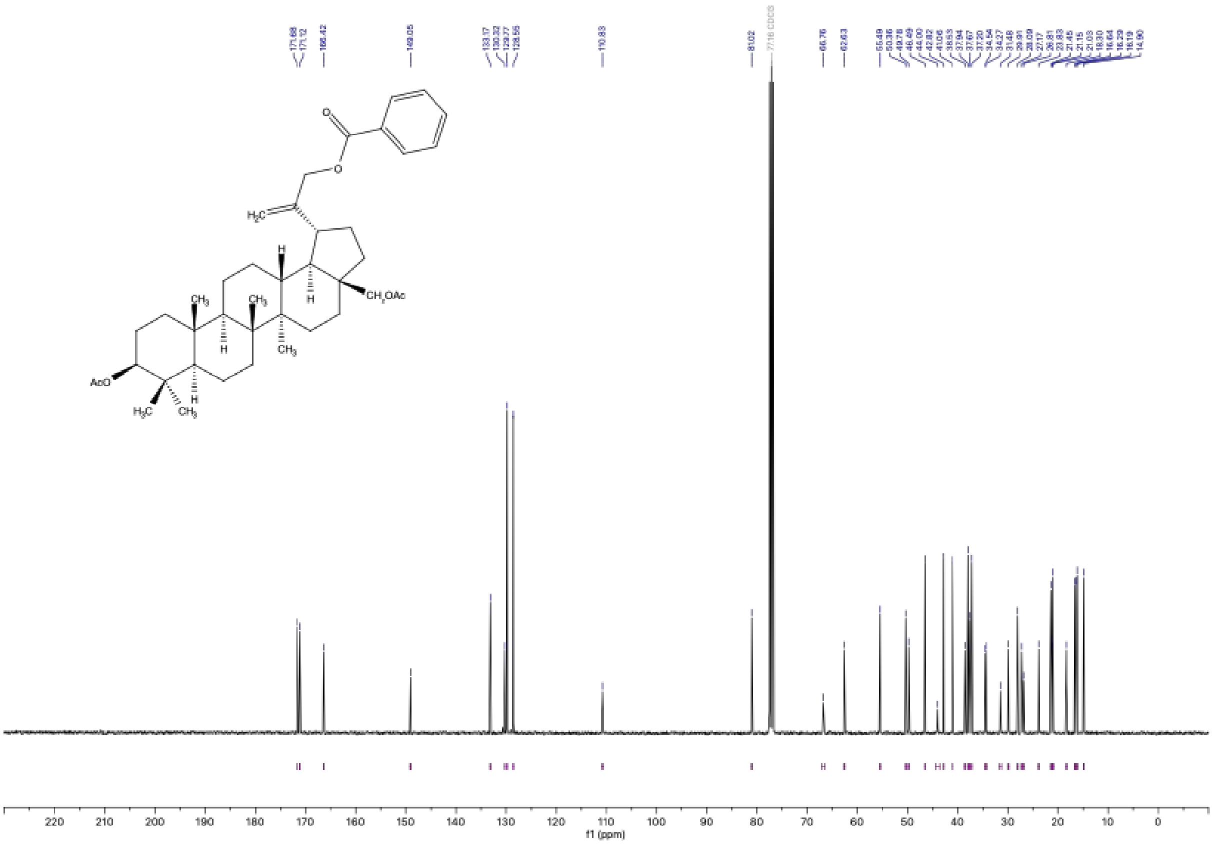

Figure S92. ^{13}C NMR spectrum of the compound **31** ($CDCl_3$, 101 MHz).

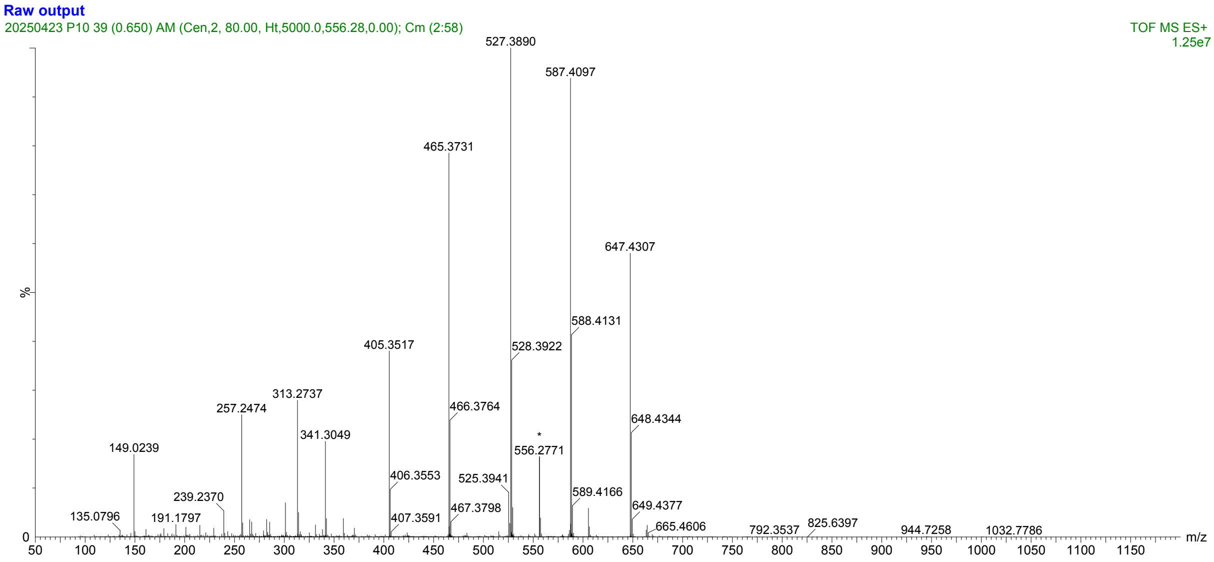

Figure S93. HRMS spectrum of the compound **31**.

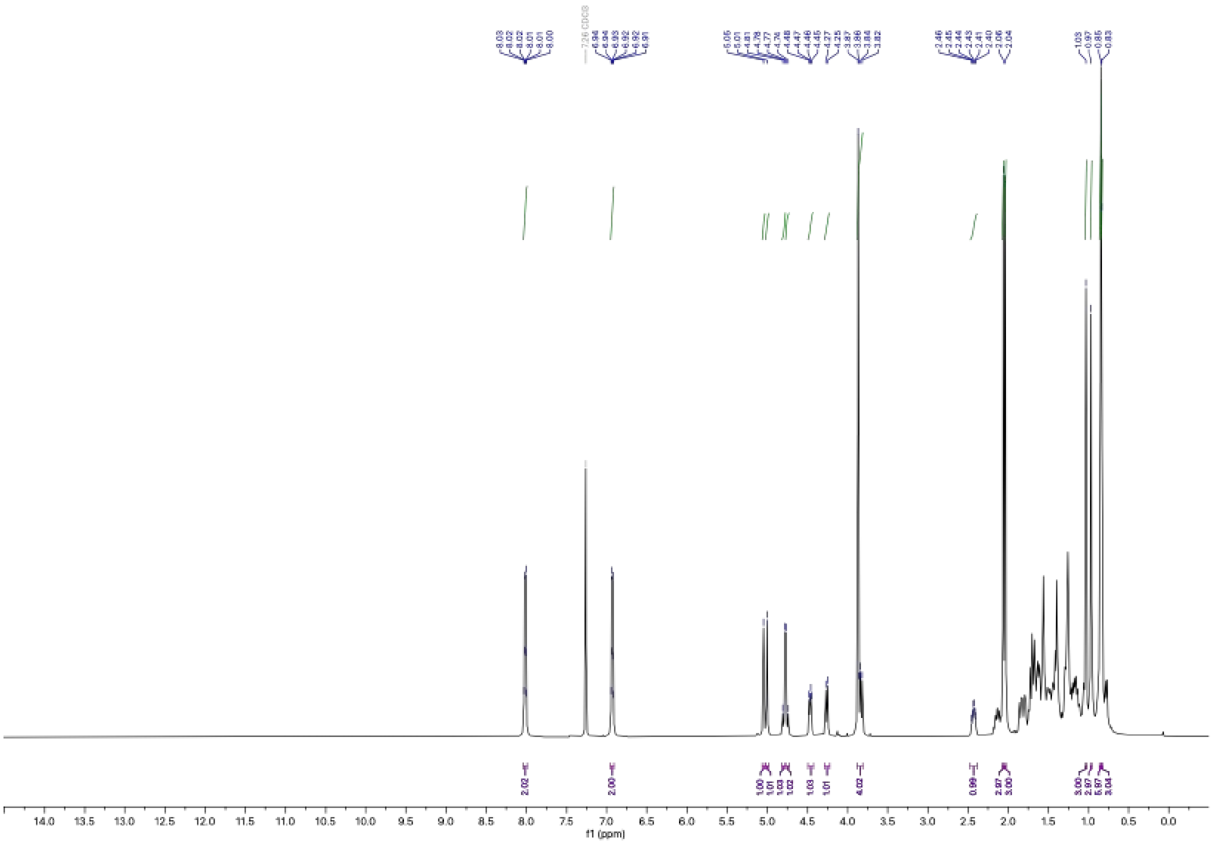

Figure S94. ^{1}H NMR spectrum of the compound **32** ($CDCl_3$, 500 MHz).

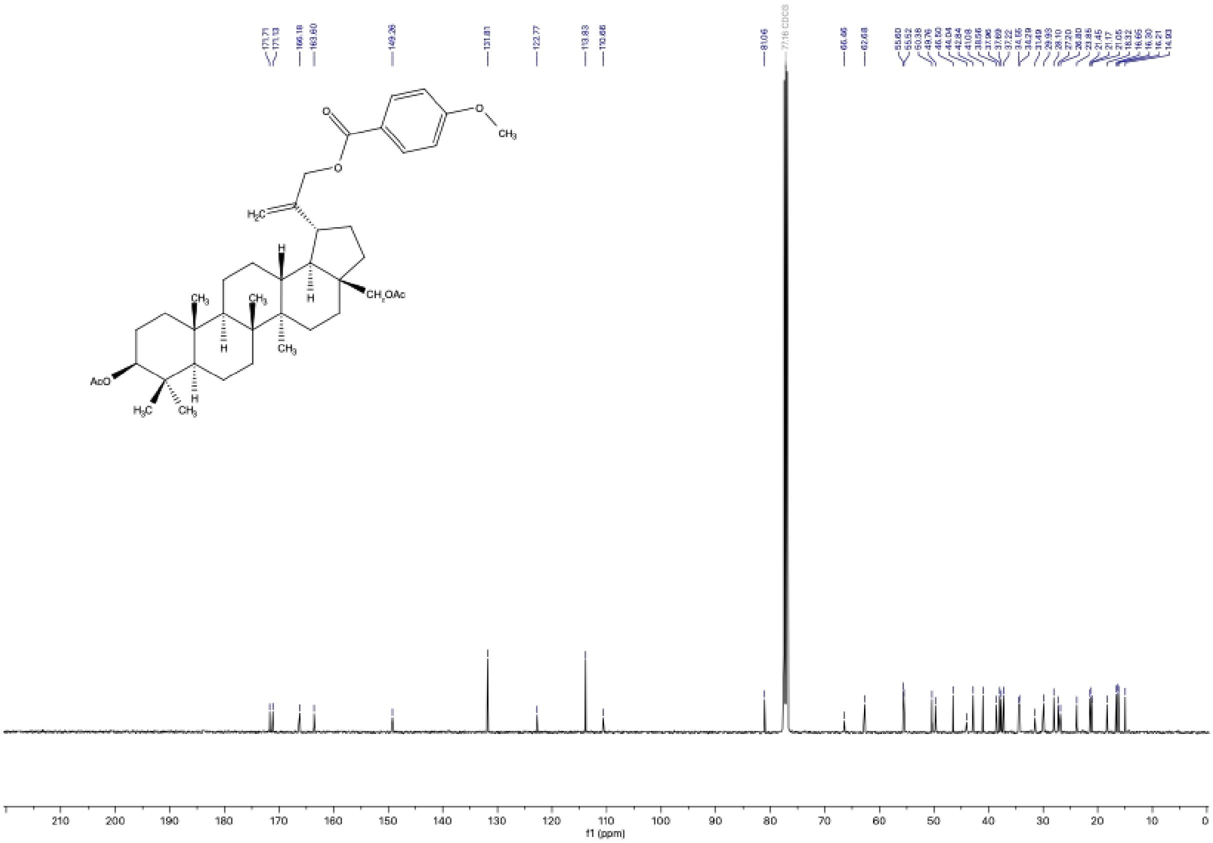

Figure S95. ^{13}C NMR spectrum of the compound **32** ($CDCl_3$, 126 MHz).

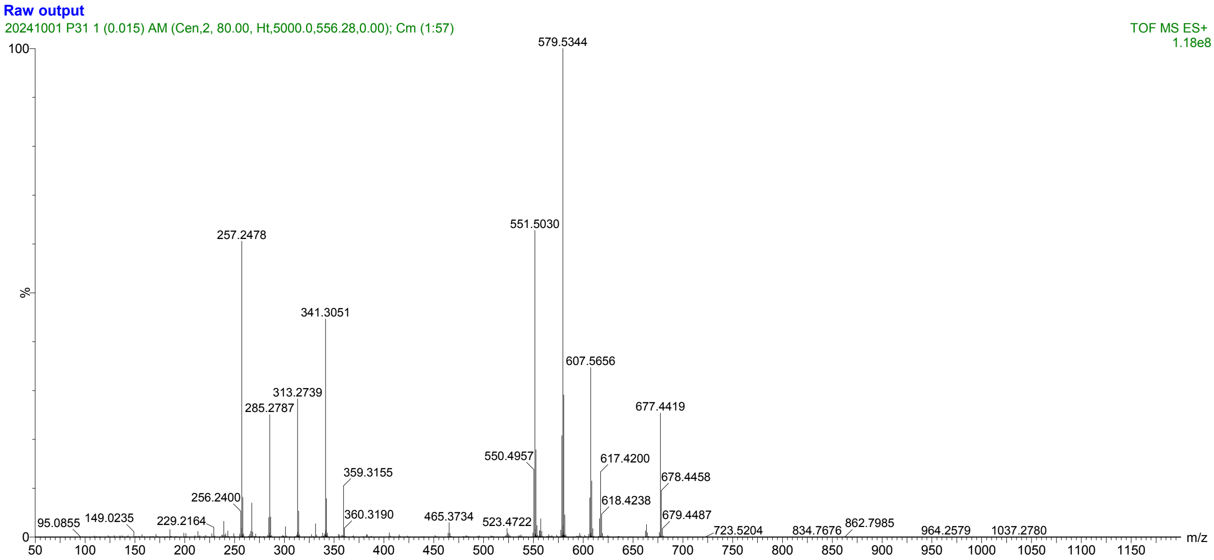

Figure S96. HRMS spectrum of the compound **32**.

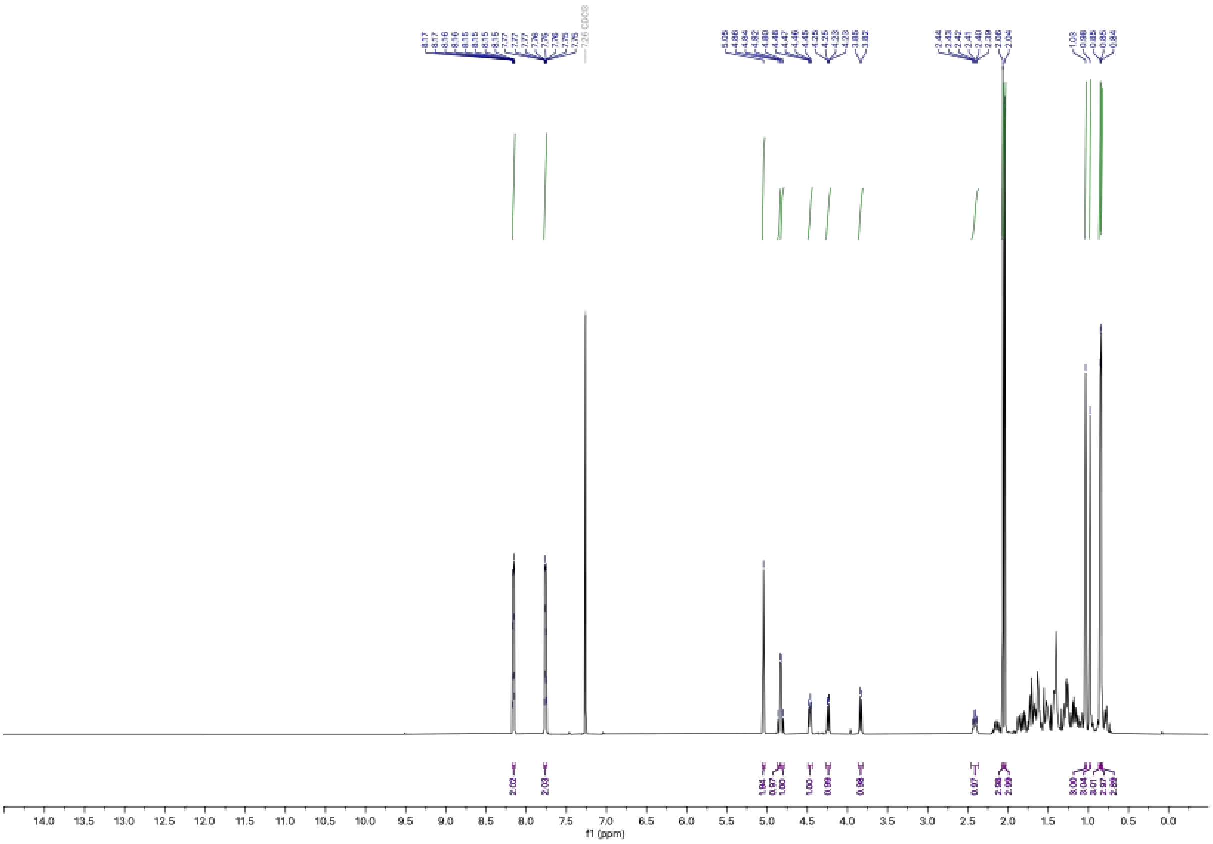

Figure S97. ^{1}H NMR spectrum of the compound **33** ($CDCl_3$, 500 MHz).

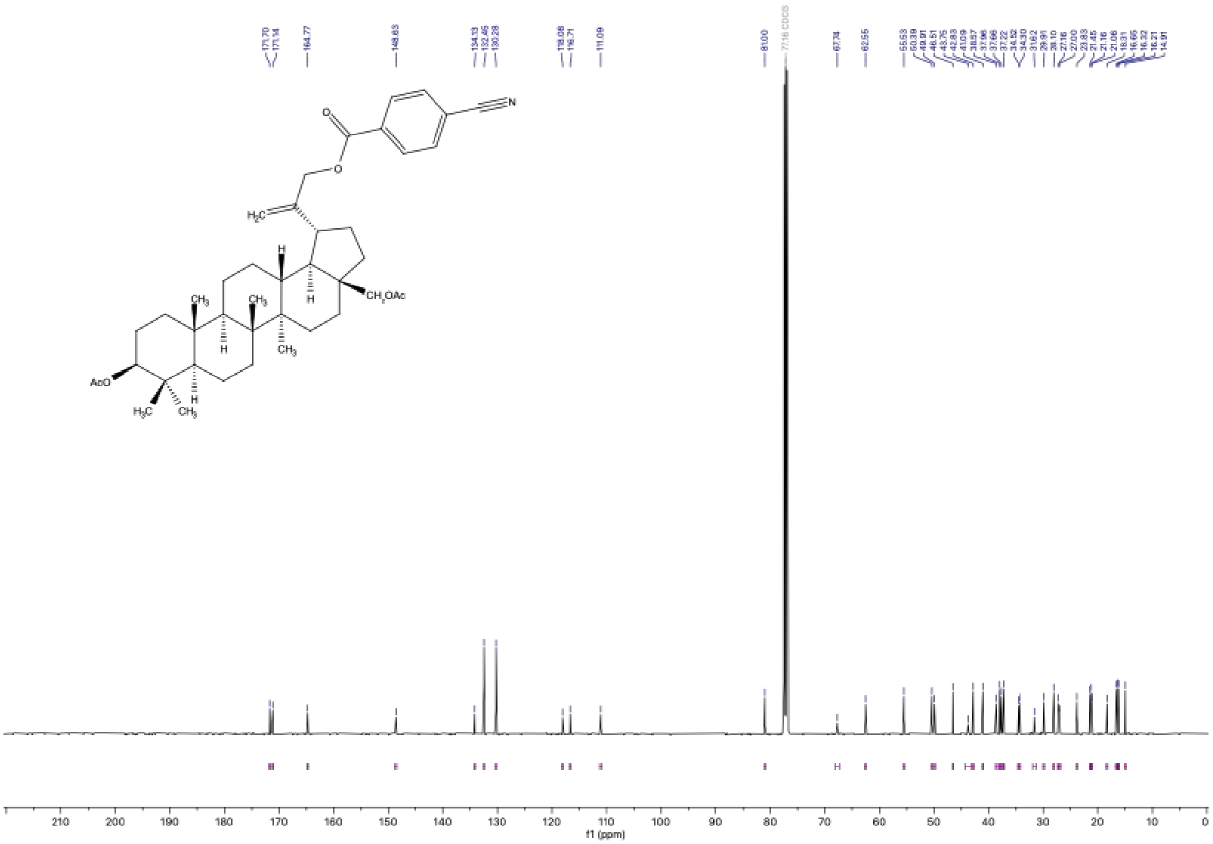

Figure S98. ^{13}C NMR spectrum of the compound **33** ($CDCl_3$, 126 MHz).

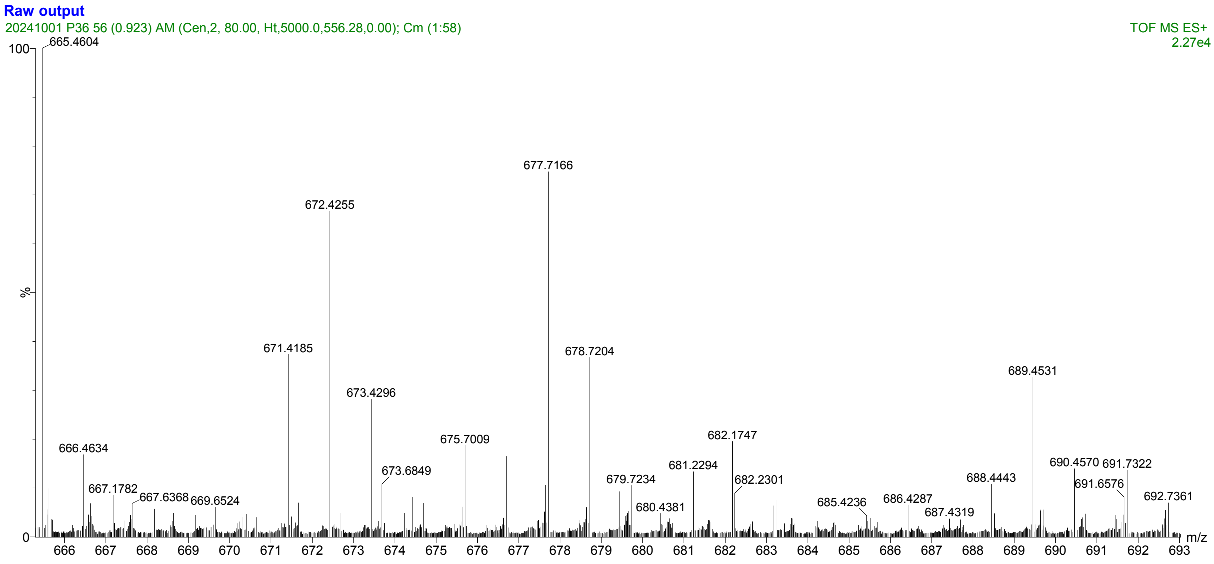

Figure S99. HRMS spectrum of the compound **33**.

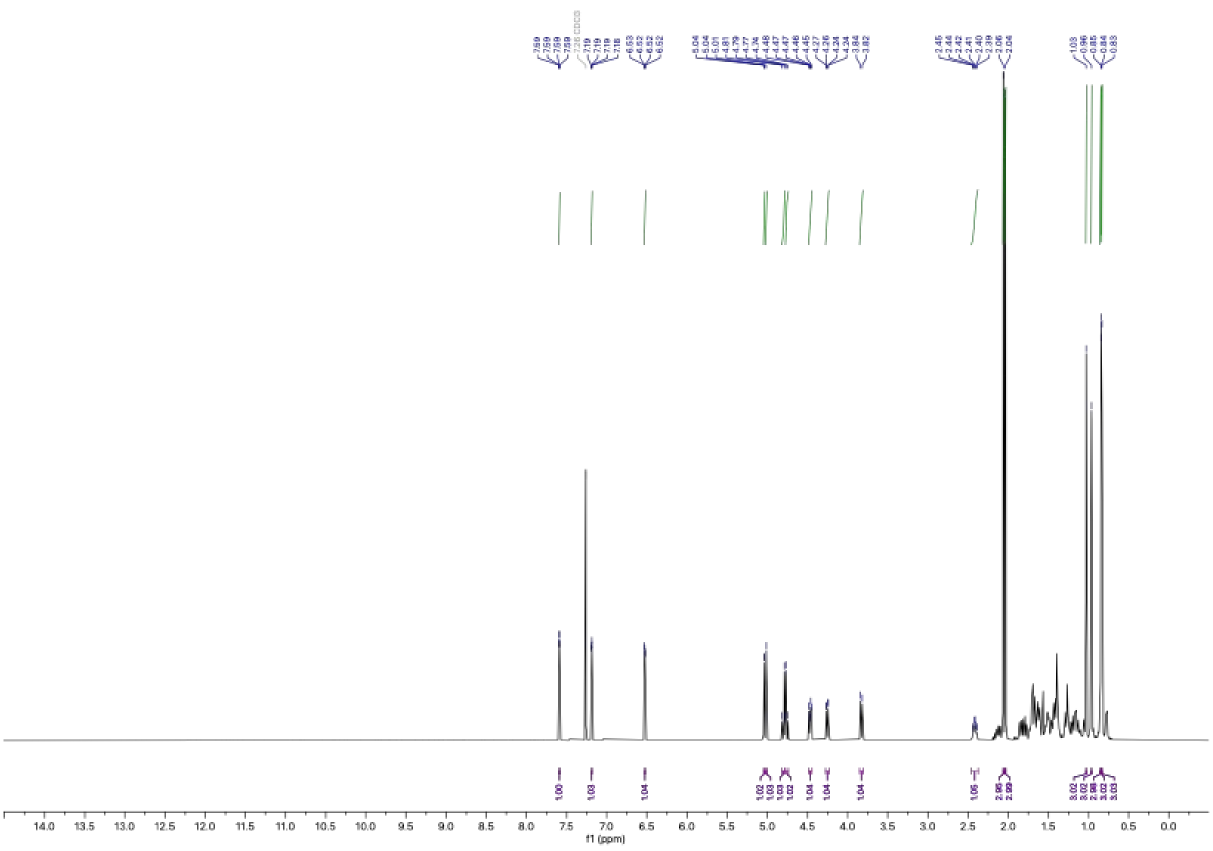

Figure S100. ^{1}H NMR spectrum of the compound **34** ($CDCl_3$, 500 MHz).

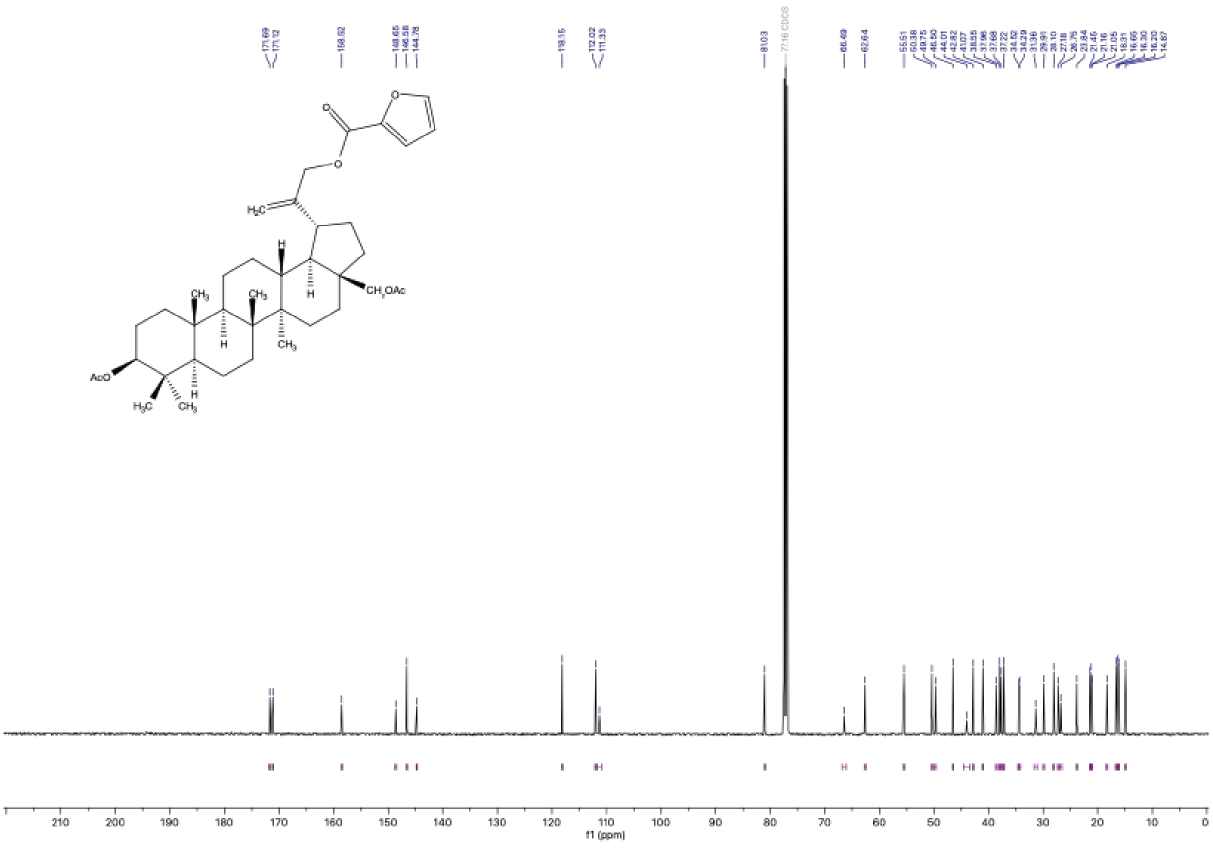

Figure S101. ^{13}C NMR spectrum of the compound **34** ($CDCl_3$, 126 MHz).

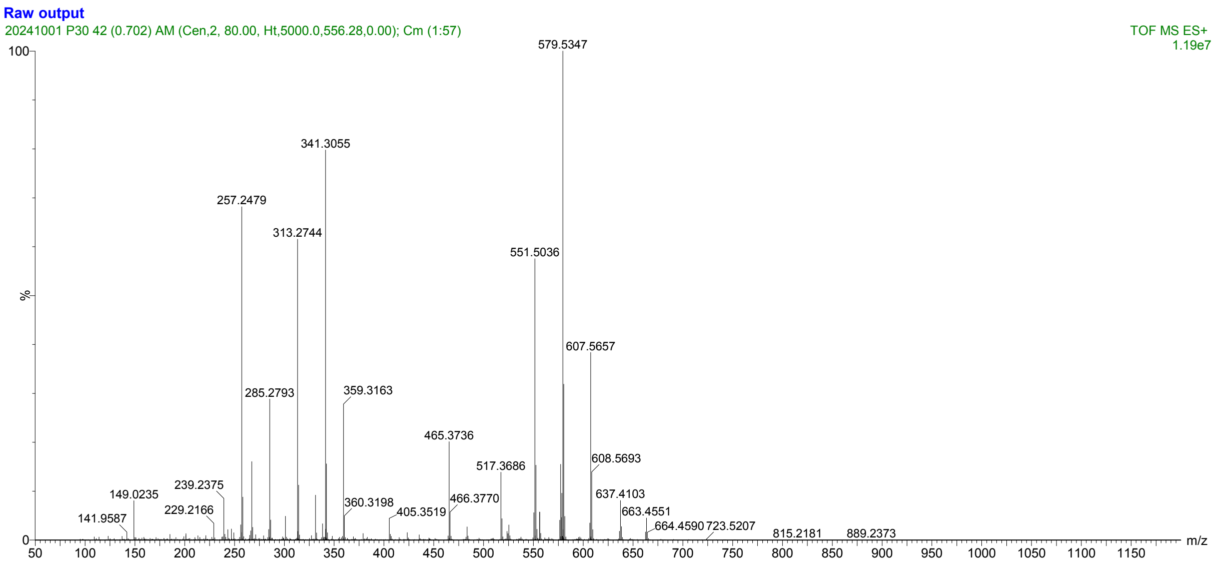

Figure S102. HRMS spectrum of the compound **34**.

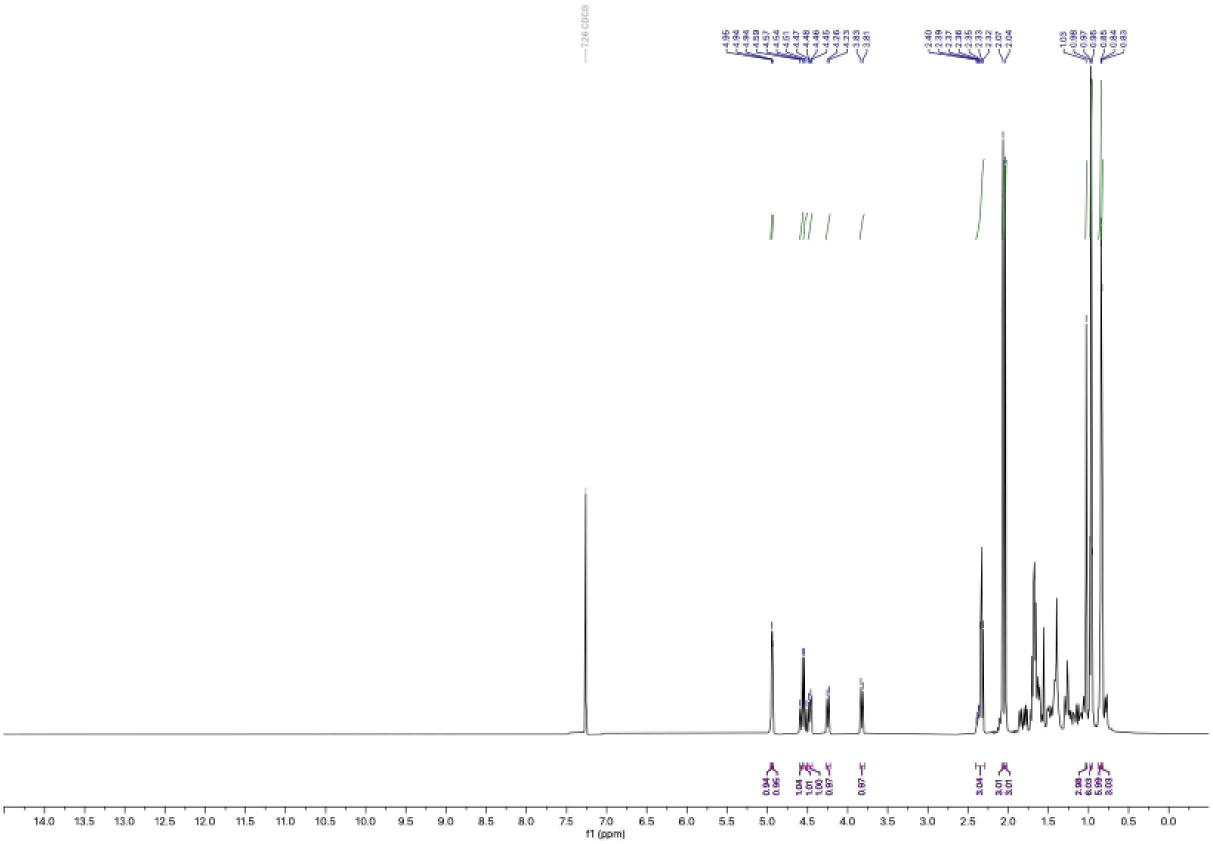

Figure S103. ^{1}H NMR spectrum of the compound **35** ($CDCl_3$, 500 MHz).

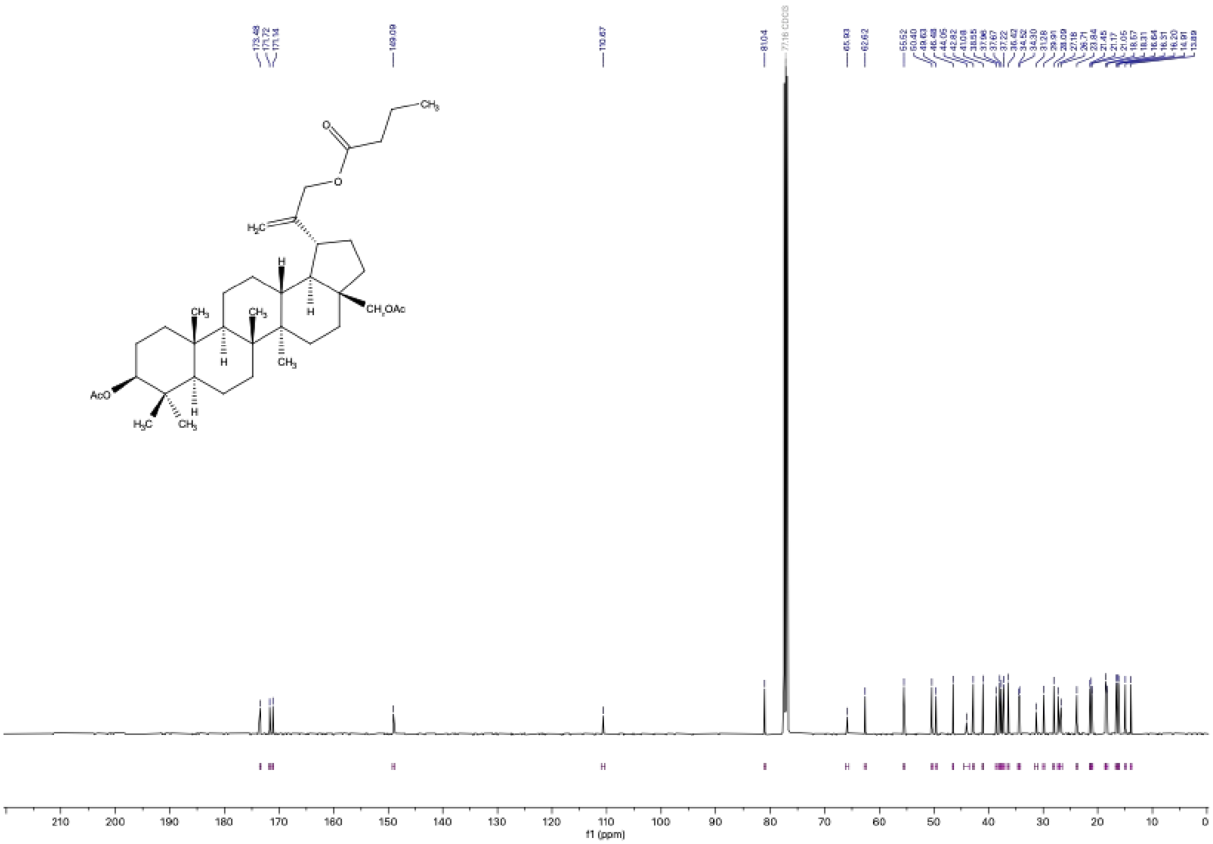

Figure S104. ^{13}C NMR spectrum of the compound **35** ($CDCl_3$, 126 MHz).

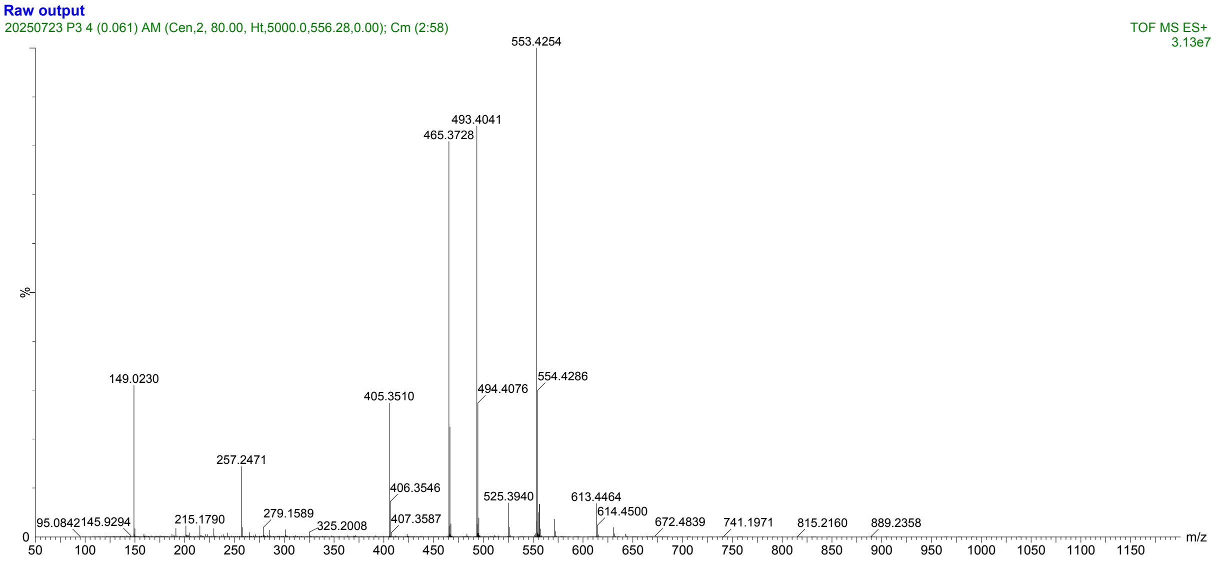

Figure S105. HRMS spectrum of the compound **35**.

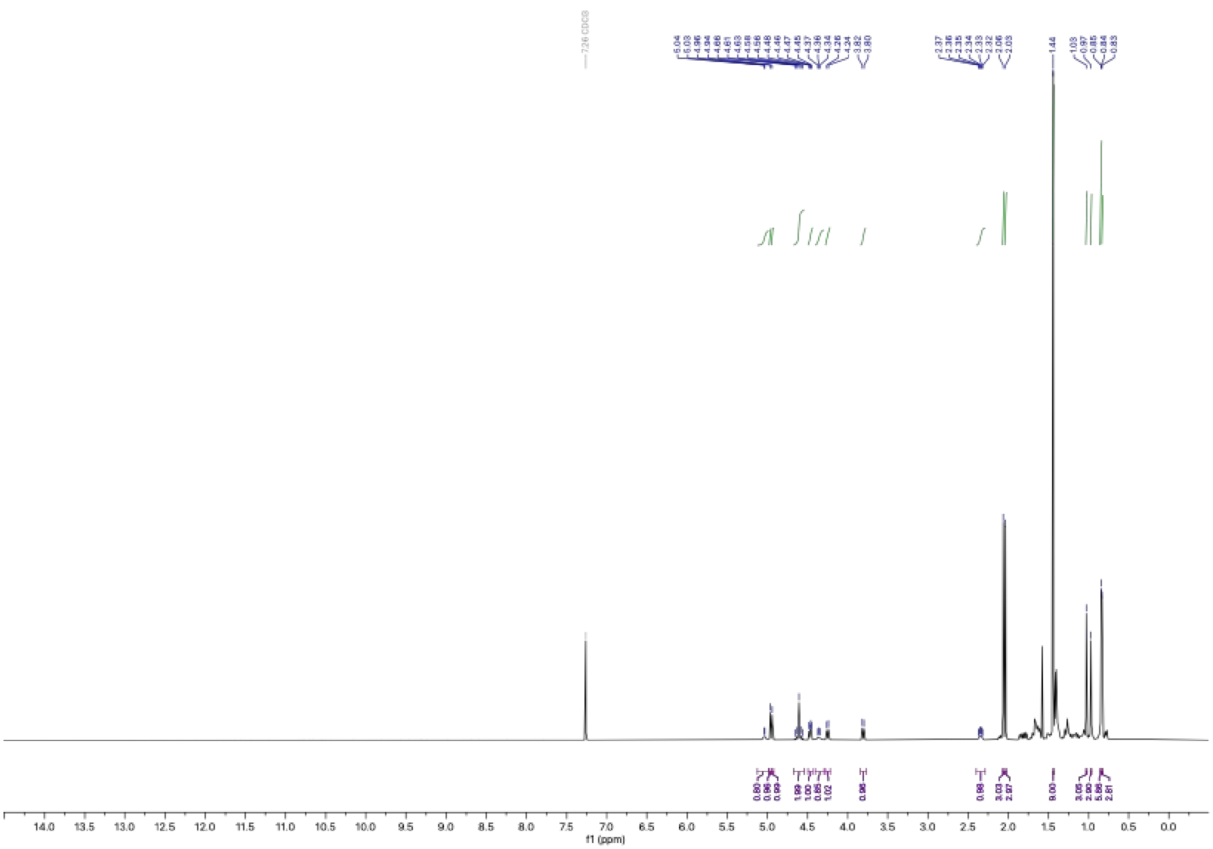

Figure S106. ^{1}H NMR spectrum of the compound **36** ($CDCl_3$, 500 MHz).

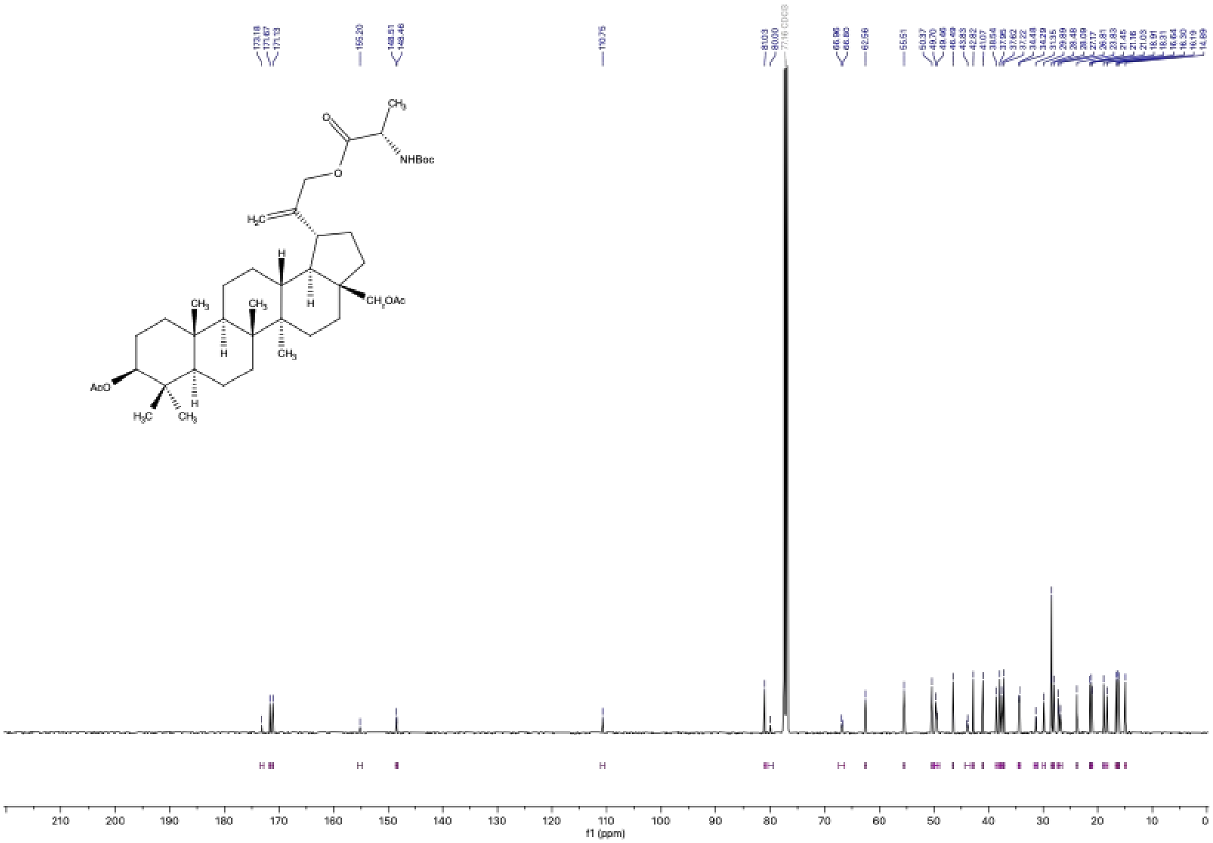

Figure S107. ^{13}C NMR spectrum of the compound **36** ($CDCl_3$, 126 MHz).

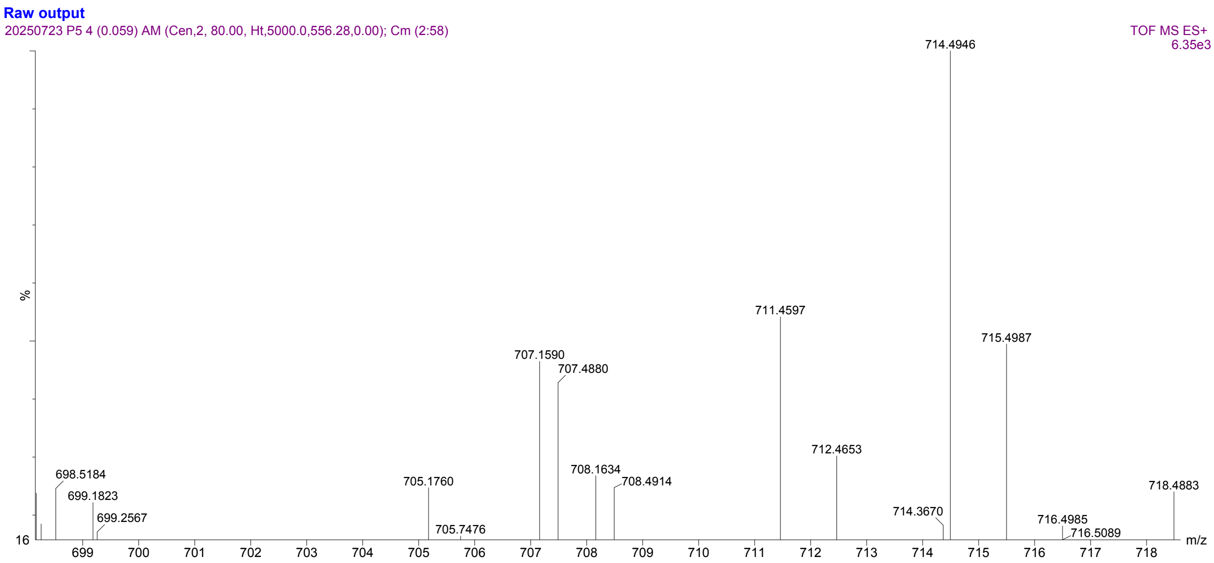

Figure S108. HRMS spectrum of the compound **36**.

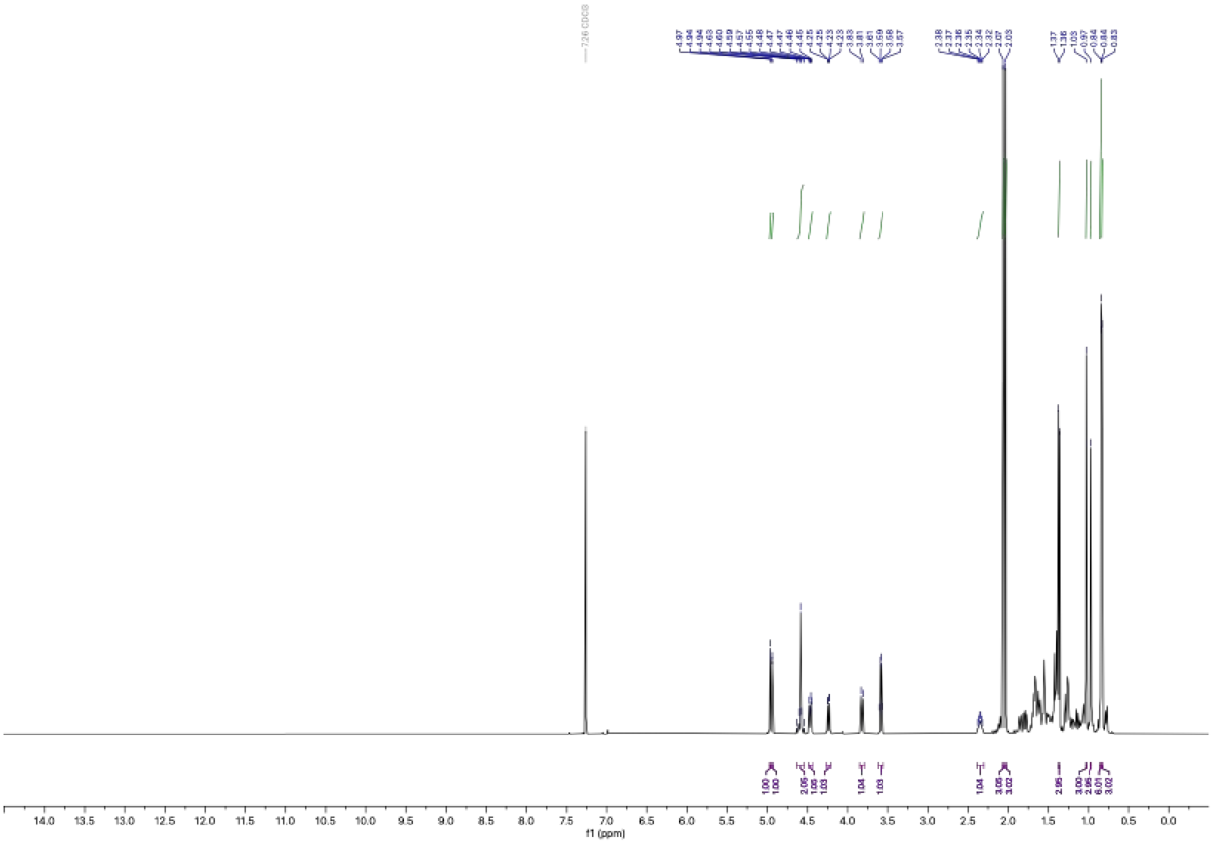

Figure S109. ^{1}H NMR spectrum of the compound **37** ($CDCl_3$, 500 MHz).

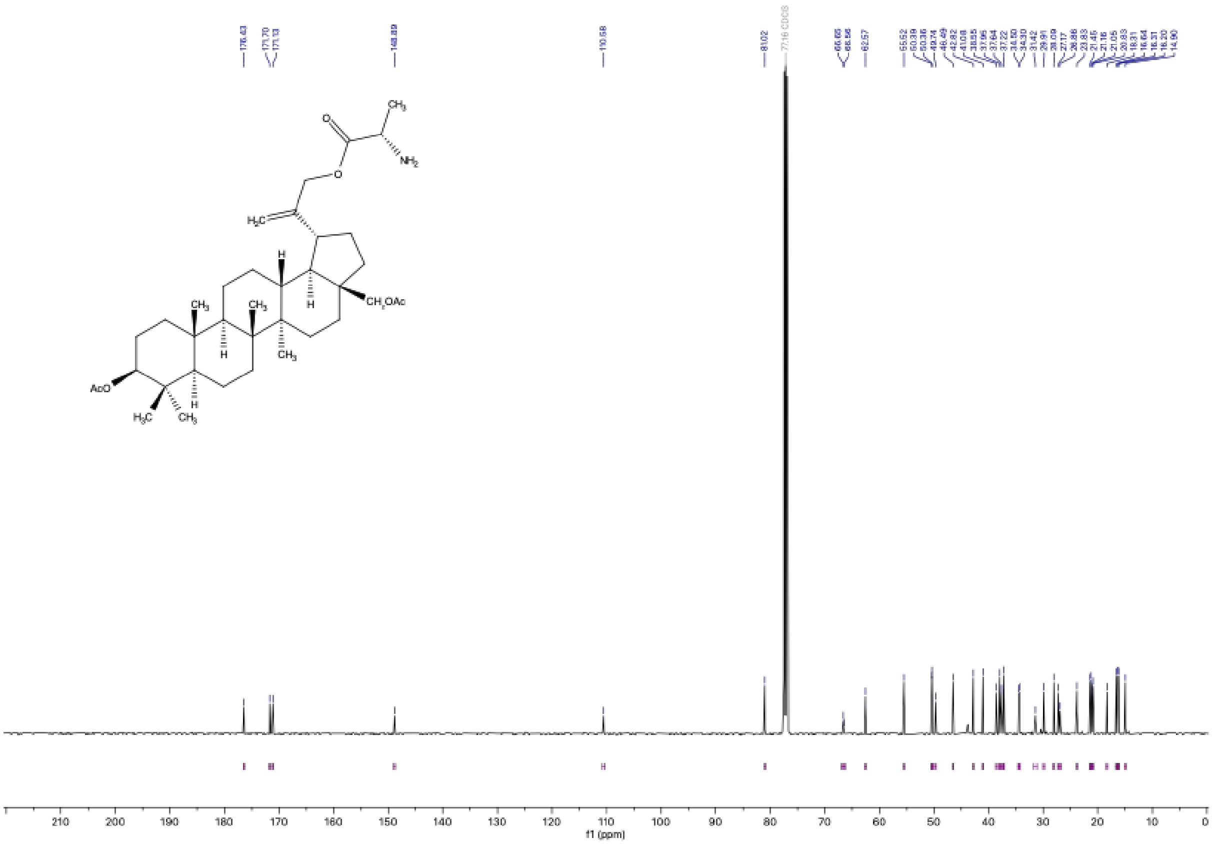

Figure S110. ^{13}C NMR spectrum of the compound **37** ($CDCl_3$, 126 MHz).

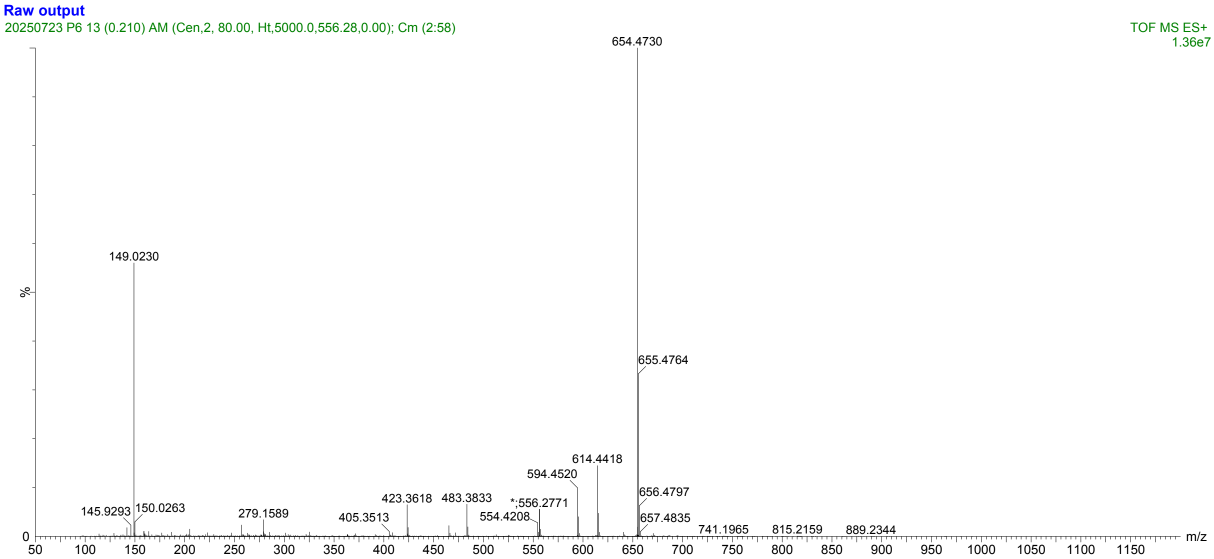

Figure S111. HRMS spectrum of the compound **37**.

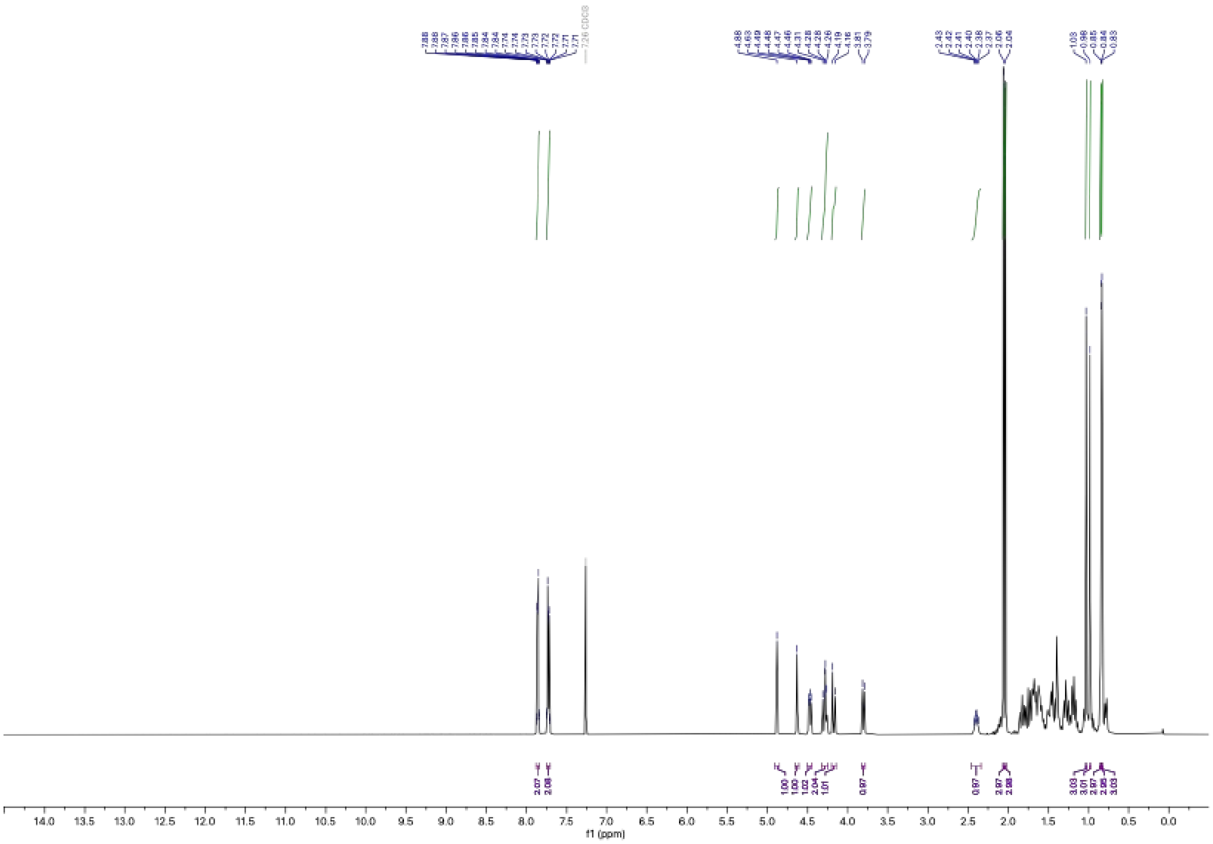

Figure S112. ^{1}H NMR spectrum of the compound **38** ($CDCl_3$, 500 MHz).

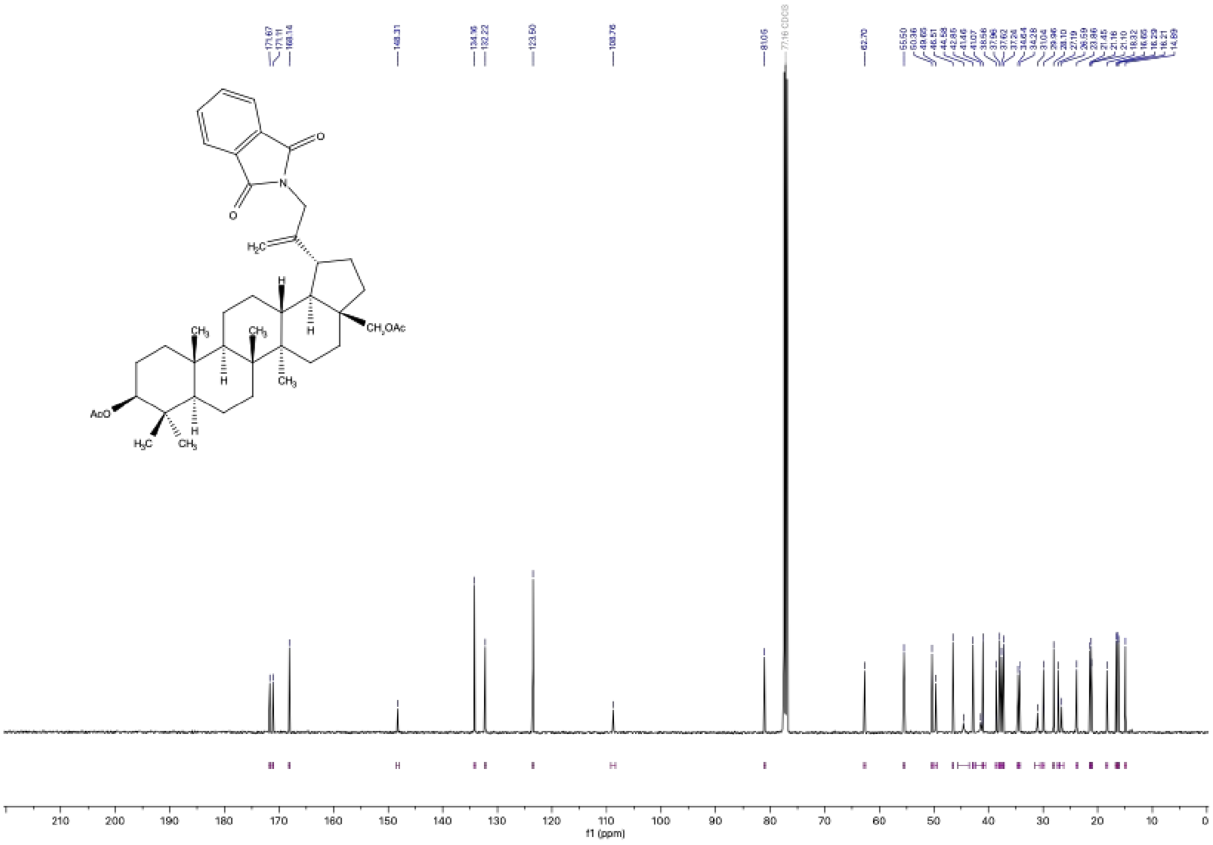

Figure S113. ^{13}C NMR spectrum of the compound **38** ($CDCl_3$, 126 MHz).

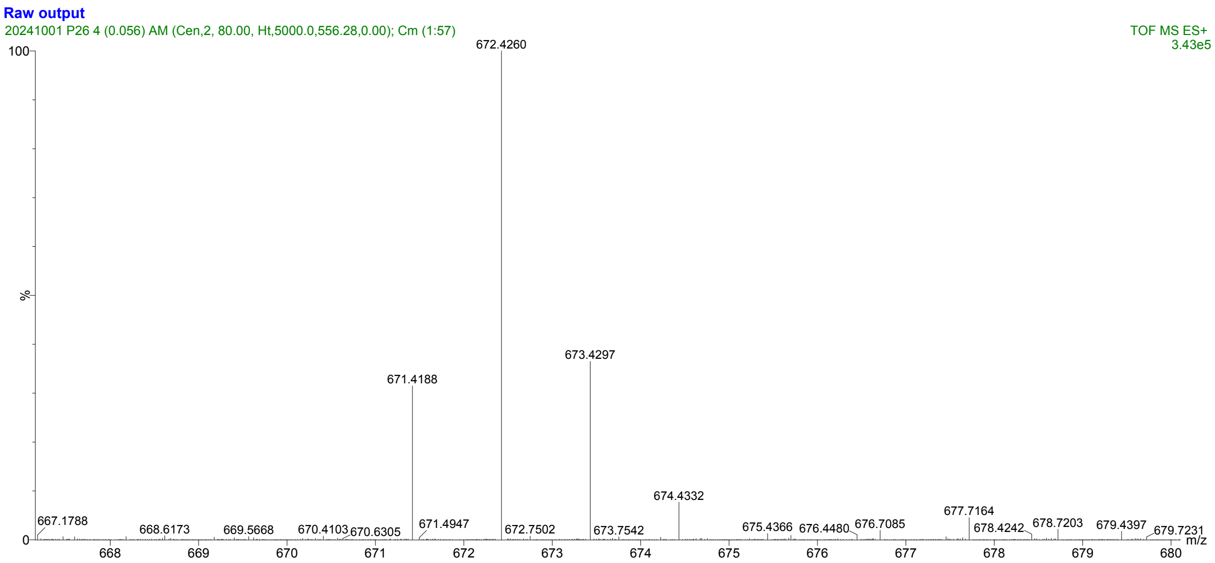

Figure S114. HRMS spectrum of the compound **38**.

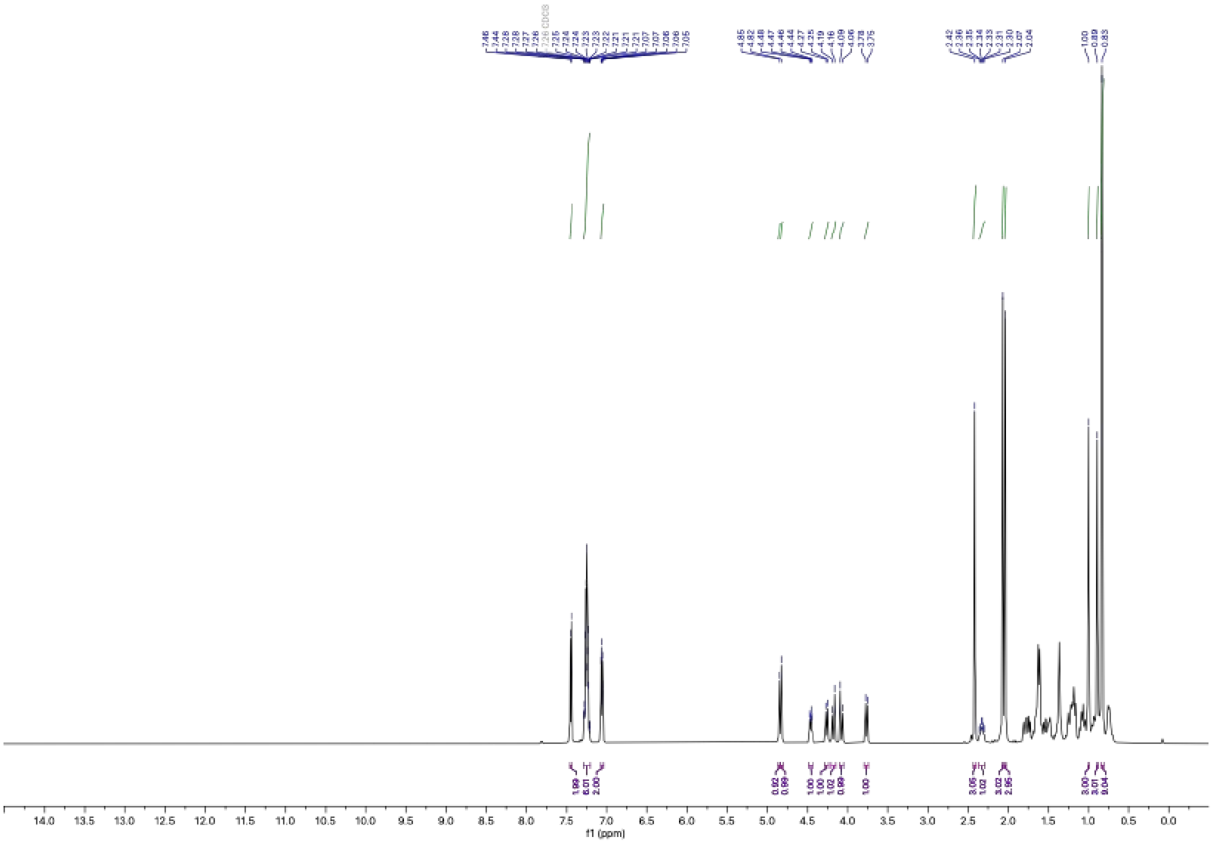

Figure S115. ^{1}H NMR spectrum of the compound **39** ($CDCl_3$, 500 MHz).

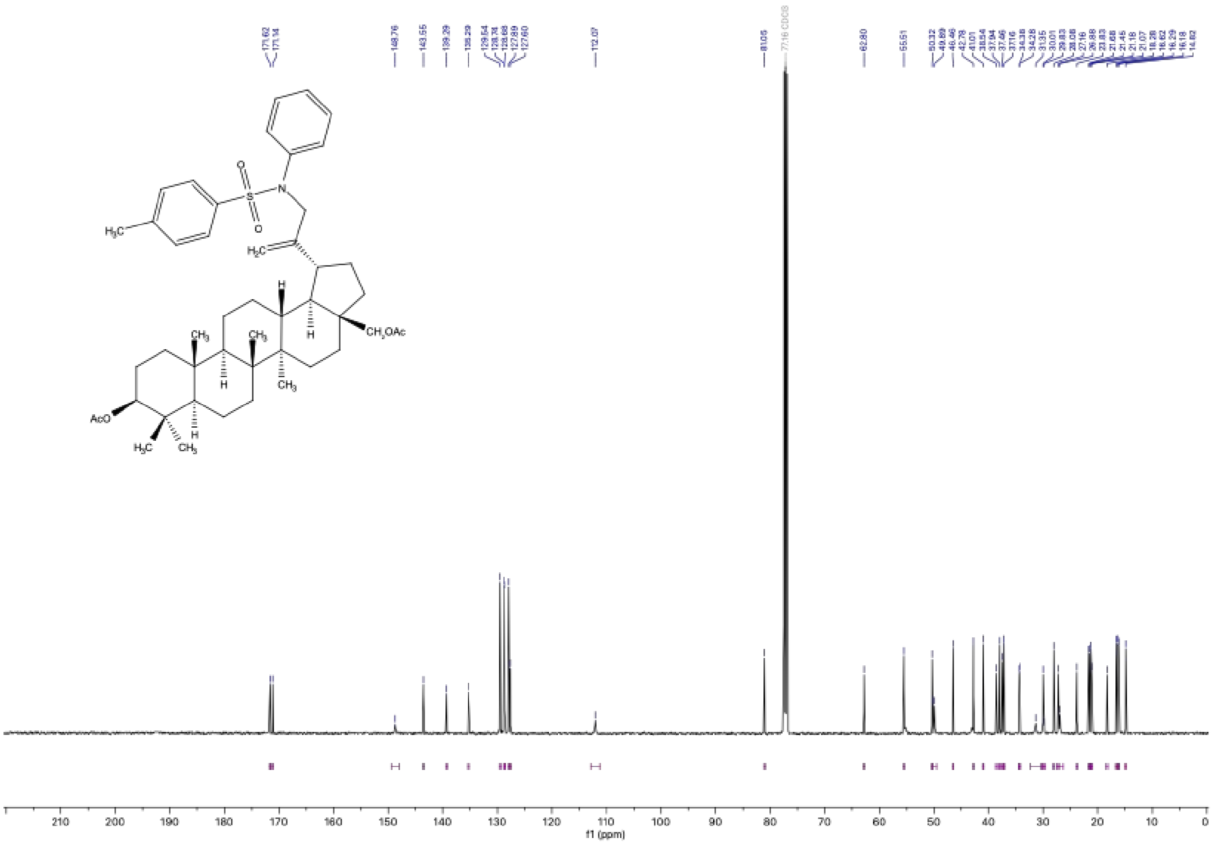

Figure S116. ^{13}C NMR spectrum of the compound **39** ($CDCl_3$, 126 MHz).

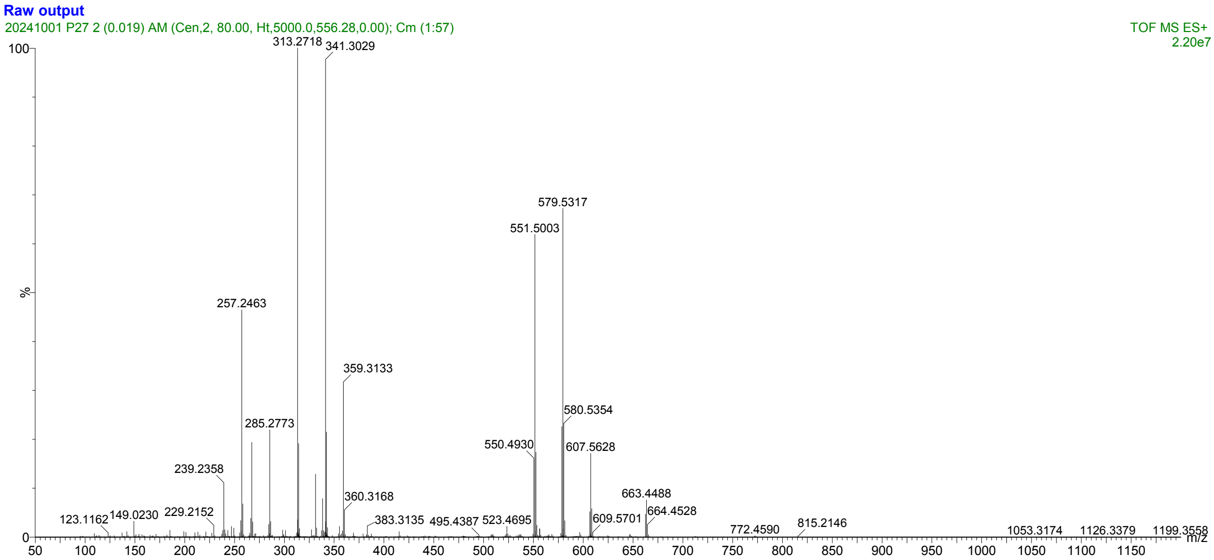

Figure S117. HRMS spectrum of the compound **39**.

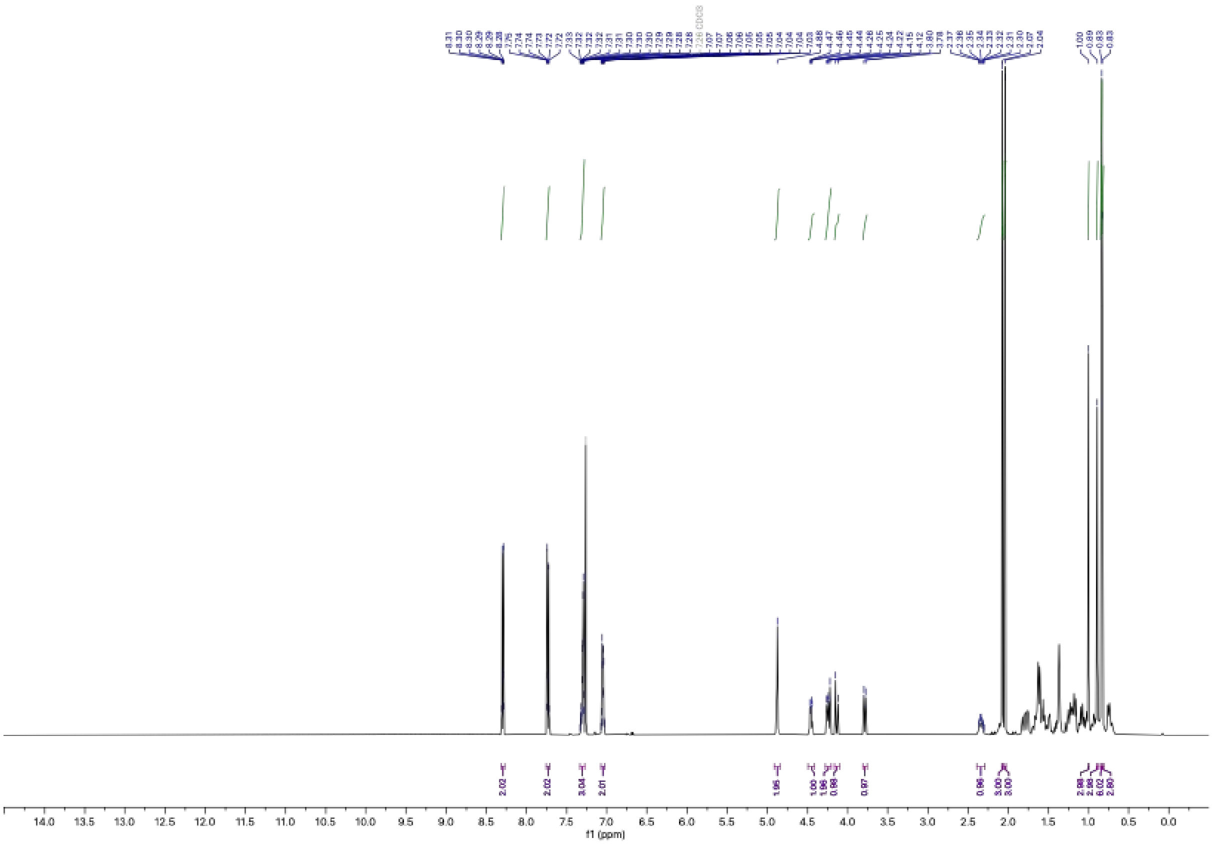

Figure S118. ^{1}H NMR spectrum of the compound **40** ($CDCl_3$, 500 MHz).

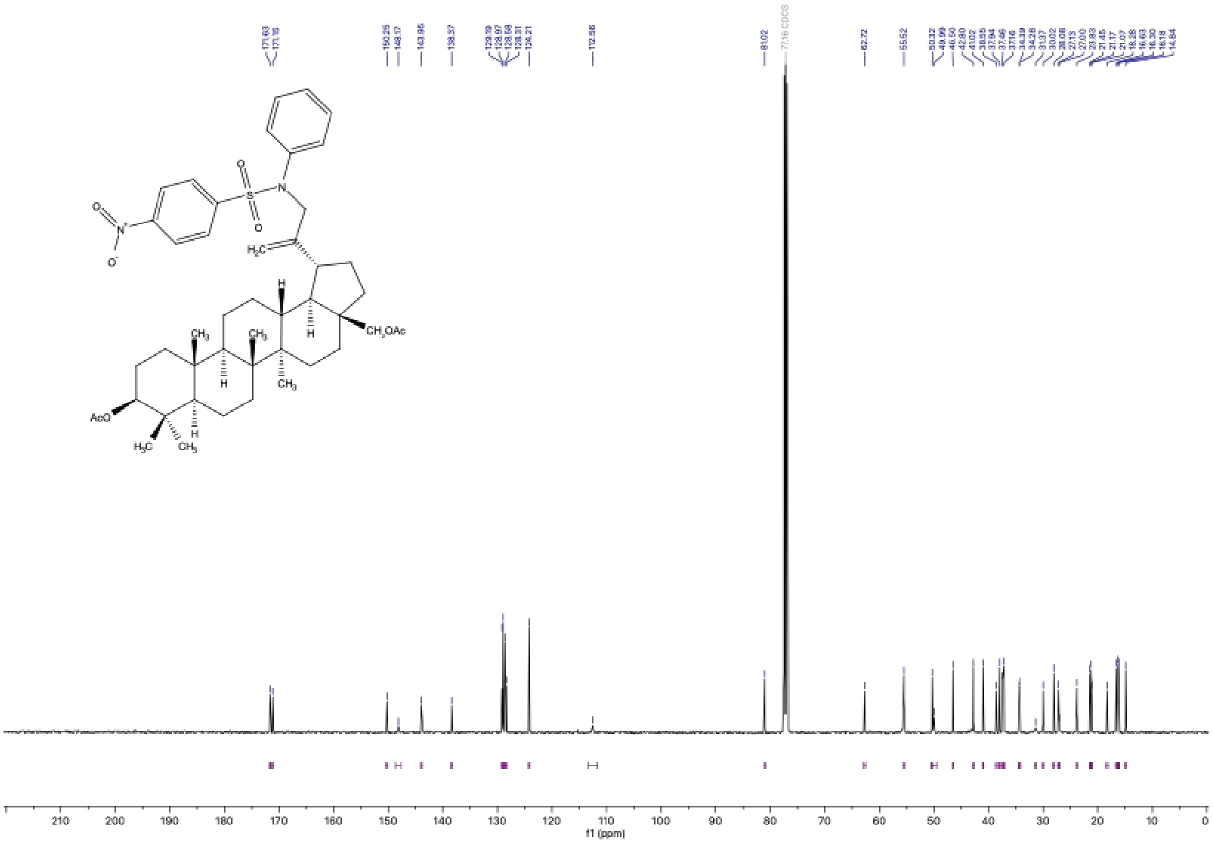

Figure S119. ^{13}C NMR spectrum of the compound **40** ($CDCl_3$, 126 MHz).

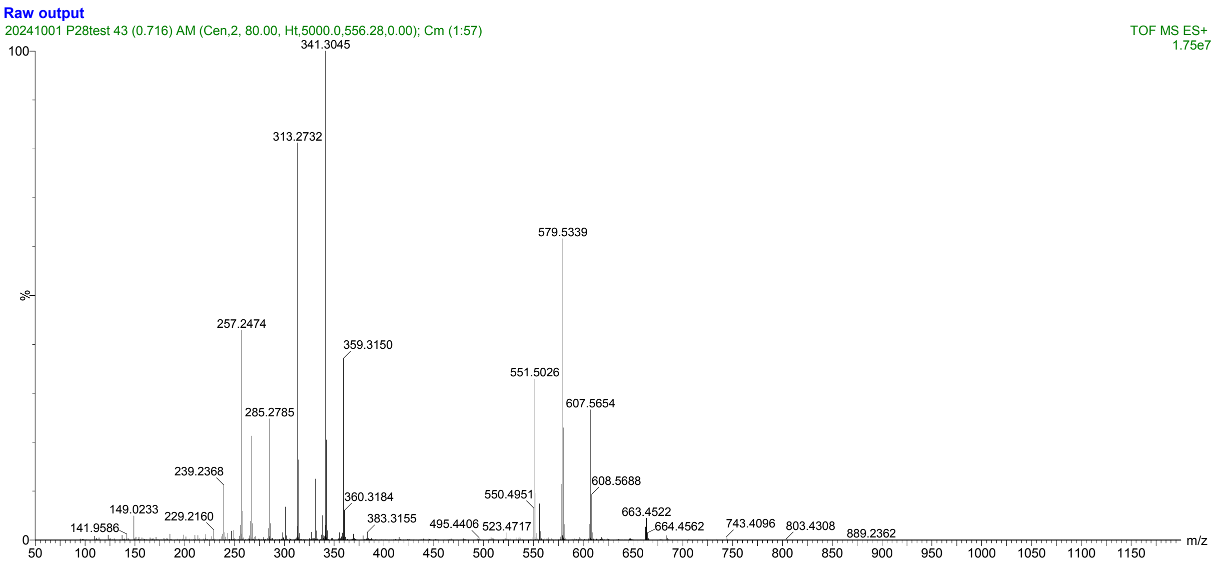

Figure S120. HRMS spectrum of the compound **40**.

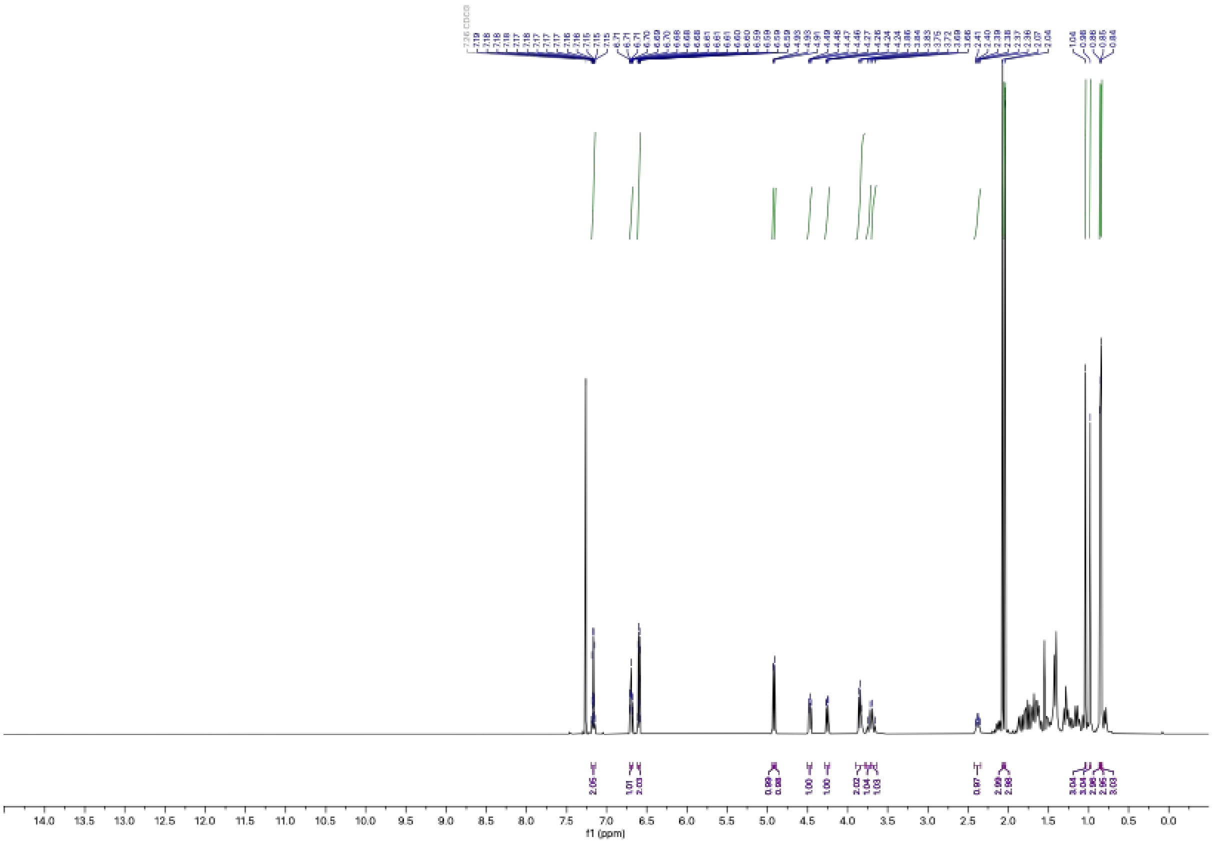

Figure S121. ^{1}H NMR spectrum of the compound **41** ($CDCl_3$, 500 MHz).

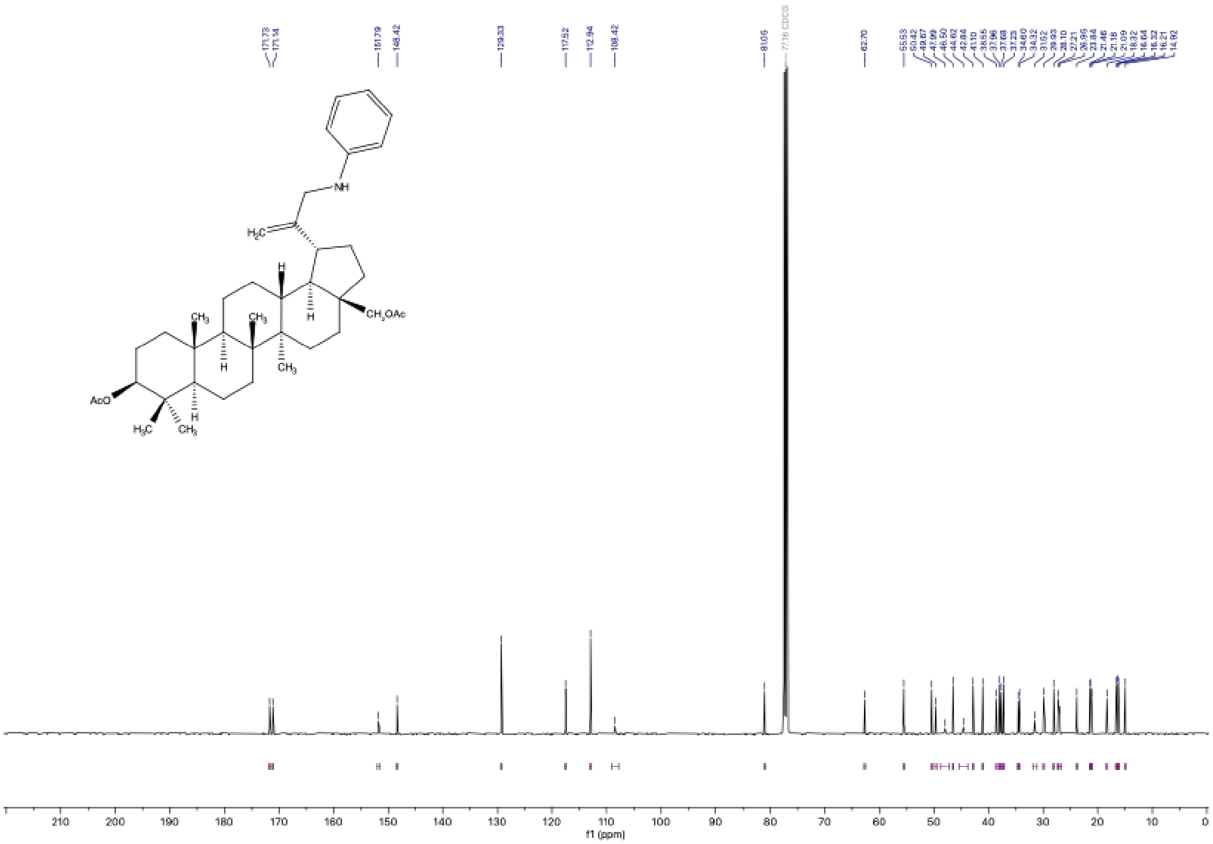

Figure S122. ^{13}C NMR spectrum of the compound **41** ($CDCl_3$, 126 MHz).

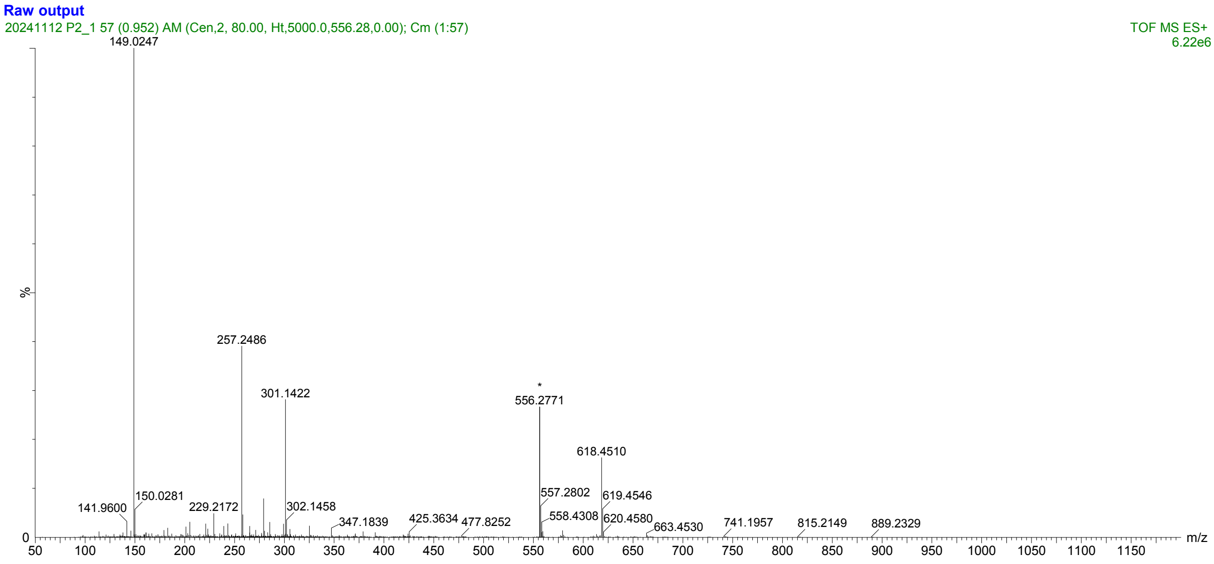

Figure S123. HRMS spectrum of the compound **41**.

Table S1. Full table of all measured cytotoxic activities in all used cancer cells.

Comp.	IC50 (μmol/L)[a]								
	CCRF-CEM	HCT116	HCT116 p53$^{-/-}$	K562	A549	U2OS	BJ	MRC-5	SI[b]
**betulin diacetate**	>50	>50	>50	>50	>50	>50	>50	>50	>1
**2**	16.68±2.14	34.96±1.88	39.94±2.06	39.98±6.42	38.69±2.12	26.27±2.34	>50	>50	>3.00
**3**	>50	>50	>50	>50	>50	>50	>50	>50	>1
**4**	>50	>50	>50	>50	>50	>50	>50	>50	>1
**5**	>50	>50	>50	>50	>50	>50	>50	>50	>1
**6**	>50	>50	>50	>50	>50	>50	>50	>50	>1
**7**	>50	>50	>50	>50	>50	>50	>50	>50	>1
**8**	>50	>50	>50	>50	>50	>50	>50	>50	>1
**9**	>50	>50	>50	>50	>50	>50	>50	>50	>1
**10**	>50	>50	>50	>50	>50	>50	>50	>50	>1
**11**	>50	>50	>50	>50	>50	>50	>50	>50	>1
**12**	>50	>50	>50	>50	>50	>50	>50	>50	>1
**13**	>50	>50	>50	>50	>50	>50	>50	>50	>1
**14**	>50	>50	>50	>50	>50	>50	>50	>50	>1
**15**	>50	>50	>50	>50	>50	>50	>50	>50	>1
**16**	>50	>50	>50	>50	>50	>50	>50	>50	>1
**17**	>50	>50	>50	>50	>50	>50	>50	>50	>1
**18**	>50	>50	>50	>50	>50	>50	>50	>50	>1
**19**	>50	>50	>50	>50	>50	>50	>50	>50	>1
**20**	>50	>50	>50	>50	>50	>50	>50	>50	>1
**21**	>50	>50	>50	>50	>50	>50	>50	>50	>1
**22**	44.28±3.97	>50	>50	>50	>50	>50	>50	>50	>1.13
**23**	>50	>50	>50	>50	>50	>50	>50	>50	>1
**24**	>50	>50	>50	>50	>50	>50	>50	>50	>1
**25**	>50	>50	>50	>50	>50	>50	>50	>50	>1
**26**	>50	>50	>50	>50	>50	>50	>50	>50	>1
**27**	16.90±2.75	>50	>50	>50	>50	>50	>50	>50	>2.96
**28**	>50	>50	>50	>50	>50	>50	>50	>50	>1
**29**	>50	>50	>50	>50	>50	>50	>50	>50	>1
**30**	9.61±2.50	31.02±1.17	30.37±1.70	23.24±3.09	>50	28.28±1.93	40.01±3.31	>50	>4.68
**31**	>50	>50	>50	>50	>50	>50	>50	>50	>1
**32**	>50	>50	>50	>50	>50	>50	>50	>50	>1
**33**	>50	>50	>50	>50	>50	>50	>50	>50	>1
**34**	>50	>50	>50	>50	>50	>50	>50	>50	>1
**35**	>50	>50	>50	>50	>50	>50	>50	>50	>1
**36**	>50	>50	>50	>50	>50	>50	>50	>50	>1
**37**	5.65±0.53	12.30±3.33	8.40±1.53	6.65±0.23	25.91±3.34	5.45±0.65	26.42±2.84	25.00±3.49	4.55
**38**	>50	>50	>50	>50	>50	>50	>50	>50	>1
**39**	>50	>50	>50	>50	>50	>50	>50	>50	>1
**40**	>50	>50	>50	>50	>50	>50	>50	>50	>1
**41**	>50	>50	>50	>50	>50	>50	>50	>50	>1

[a]The IC_{50} represents the concentration of the drug required to inhibit cell growth by 50%. The standard deviation in cytotoxicity assays typically reaches up to 15% of the mean value. [b]The selectivity index is calculated based on the IC_{50} for the CCRF-CEM line versus the average IC_{50} for both fibroblast lines. For IC_{50} values reported as > 50 µM, SI values are expressed as lower bounds.

Table S2. Predicted ADME and Physicochemical Properties of selected compounds using the SwissADME webserver[45].

Property	Compound **2**	Compound **27**	Compound **30**	Compound **37**
**Molecular Weight (g/mol)**	542.79	619.87	719.99	613.87
**Log $P_{o/w}$ (SILICOS-IT)**	6.65	7.70	8.20	6.60
**Log S (SILICOS-IT)**	-6.80	-9.16	-9.56	-7.05
**Solubility Class**	Poorly soluble	Poorly soluble	Poorly soluble	Poorly soluble
**GI Absorption**	Low	Low	Low	Low
**BBB Permeant**	No	No	No	No
**P-gp Substrate**	No	No	No	Yes
**CYP2D6 Inhibitor**	No	No	No	No
**Log K_p**	-3.80 cm/s	-3.36 cm/s	-3.73 cm/s	-4.19 cm/s
**Lipinski Violations**	No; 2 violations: MW>500, MLOGP>4.15	No; 2 violations: MW>500, MLOGP>4.15	No; 2 violations: MW>500, MLOGP>4.15	No; 2 violations: MW>500, MLOGP>4.15
**Bioavailability Score**	0.17	0.17	0.17	0.17

Table S3. Predicted Toxicity Properties of selected compounds using the ProTox 3.0 webserver[49].

	Compound **2**	Compound **27**	Compound **30**	Compound **37**
**Predicted LD50**	5000mg/kg	800mg/kg	1503mg/kg	3424mg/kg
**Predicted Toxicity Class**	5	4	4	5
**Prediction accuracy**	72.9%	67.38%	68.07%	69.26%
**Possible Toxicity Target with Avg Fit**	AOFA (69.18%), PGH1 (65.38%)	No possible targets found	No possible targets found	AOFA (69.87%), PGH1 (66.22%)
